# Inferring branching pathways in genome-scale metabolic networks

**DOI:** 10.1186/1752-0509-3-103

**Published:** 2009-10-29

**Authors:** Esa Pitkänen, Paula Jouhten, Juho Rousu

**Affiliations:** 1 Department of Computer Science, University of Helsinki, Finland; 2 VTT Technical Research Centre of Finland, Espoo, Finland

## Abstract

**Background:**

A central problem in computational metabolic modelling is how to find biochemically plausible pathways between metabolites in a metabolic network. Two general, complementary frameworks have been utilized to find metabolic pathways: constraint-based modelling and graph-theoretical path finding approaches. In constraint-based modelling, one aims to find pathways where metabolites are balanced in a pseudo steady-state. Constraint-based methods, such as elementary flux mode analysis, have typically a high computational cost stemming from a large number of steady-state pathways in a typical metabolic network. On the other hand, graph-theoretical approaches avoid the computational complexity of constraint-based methods by solving a simpler problem of finding shortest paths. However, while scaling well with network size, graph-theoretic methods generally tend to return more false positive pathways than constraint-based methods.

**Results:**

In this paper, we introduce a computational method, ReTrace, for finding biochemically relevant, branching metabolic pathways in an atom-level representation of metabolic networks. The method finds compact pathways which transfer a high fraction of atoms from source to target metabolites by considering combinations of linear shortest paths. In contrast to current steady-state pathway analysis methods, our method scales up well and is able to operate on genome-scale models. Further, we show that the pathways produced are biochemically meaningful by an example involving the biosynthesis of inosine 5'-monophosphate (IMP). In particular, the method is able to avoid typical problems associated with graph-theoretic approaches such as the need to define side metabolites or pathways not carrying any net carbon flux appearing in results. Finally, we discuss an application involving reconstruction of amino acid pathways of a recently sequenced organism demonstrating how measurement data can be easily incorporated into ReTrace analysis. ReTrace is licensed under GPL and is freely available for academic use at http://www.cs.helsinki.fi/group/sysfys/software/retrace/.

**Conclusion:**

ReTrace is a useful method in metabolic path finding tasks, combining some of the best aspects in constraint-based and graph-theoretic methods. It finds use in a multitude of tasks ranging from metabolic engineering to metabolic reconstruction of recently sequenced organisms.

## Background

Genome-scale metabolic reconstructions from a variety of organisms have become available in recent years [1]. At the same time, data from different organism-specific networks has been collected into "universal" metabolic databases such as KEGG [2] and BioCyc [3]. This has enabled comparative analyses of metabolism over multiple organisms [4,5], and proven useful in drug discovery [6], metabolic flux analysis [7] and metabolic engineering [8] tasks.

A typical way to query a metabolic model is to ask whether a biologically realistic connection exists in the model from a metabolite to another. We may ask this question in different contexts, depending on the task at hand. For instance, when reconstructing a metabolic network for a novel organism [9], we are interested in discovering if a previously characterized pathway is present in the organism under study [10]. Further, we may ask whether the organism possesses the ability to produce a substance, for example a particular amino acid, from available nutrients [11]. This is often either to verify that the reconstructed model has the expected structure or to predict a novel phenotype. Unfortunately, genome-scale reconstructions often contain errors, even after manual curation, which need to be taken into account during path finding [12]. In this paper, we introduce a novel method for inferring biologically relevant pathways in metabolic networks. First, we review the current methods for metabolic pathway analysis and describe our contribution. Section Methods introduces methodology, path finding problem, algorithm and its implementation. In section Results, we report the results of computational experiments. Finally, the paper ends in Conclusions.

### Review of methods for metabolic pathway analysis

Two complementary approaches have been used to answer the questions discussed above, constraint-based and graph-theoretical path finding methods. In *constraint-based *methods [11,13], one tries to infer a pathway where the intermediate metabolites are balanced in a (pseudo) steady-state. In a steady-state, the net production of each intermediate metabolite is zero. Pathways satisfying this constraint can be branching, in general consisting of one or more linear paths enabling the production of the target metabolite from sources.

On the other hand, in *graph-theoretical *methods, one typically wants to find a number of shortest paths leading from the source to the target metabolite [14-19]. Methods usually deal only with linear, non-branching pathways. Thus, graph-theoretical methods are often restricted to one source and one target metabolite. Two recent survey articles discuss the relationship between the two approaches [20,21]. Results from graph-theoretical path finding and steady-state pathway analyses complement each other. Graph-theoretical approaches tend to generate a large number of alternative pathways, which need to be filtered and ranked according to some realistic criteria to produce meaningful results. On the other hand, the computational complexity of steady-state analyses hinders the analysis of large metabolic models, though recent studies have improved the efficiency of methods [22]. Particularly, graph-theoretical analyses could be used to produce a moderate-sized representation of a genome-scale model containing only the parts relevant to the task at hand, making the steady-state analysis feasible.

In the ARM method, information on the mapping patterns of carbon atoms in metabolic reactions was utilized [14]. In ARM, a graph is first constructed where nodes corresponded to atoms of metabolites while edges described how the atoms were transferred in reactions from metabolite to metabolite. Then, to answer a path query, an algorithm returning k shortest paths was invoked to return pathways that transfer at least one carbon from source to target metabolite. Such pathways were shown often to correspond to biologically relevant pathways. Moreover, the algorithm scaled up well with the network size. However, ARM was unable to deal with branching pathways and pathways transferring more carbon atoms were not prioritized over those transferring less carbons.

Until more recently, other graph-theoretical approaches have avoided the use of atom mapping information. This is probably due to the fact the obtaining reliable mapping data is a hard problem, both computationally and biochemically. In ARM, atom mappings were computed for a number of KEGG reactions using a heuristic method based on matching of maximum common subgraphs [14]. Later, atom mapping information was added also for a subset of KEGG reactions as the KEGG RPAIR database [23].

Certain metabolites appear in many different reactions in metabolism carrying out tasks such as providing energy for reactions and maintaining redox balance. Such metabolites are commonly called *pool *or *currency metabolites*. Examples include ATP, NAD and water, which indeed have a high degree of connectivity in metabolic networks. If a pool metabolite appears in a reaction in its typical role, the metabolite is usually said to be a *side metabolite *in the particular reaction. However, assignment of metabolites to side metabolites is context-dependent. For instance, ATP appears in side metabolite role in addition to being the end product on the pathway responsible for synthesizing ATP.

In simple shortest path analysis, pool metabolites often cause false positive pathways to be identified, as the shortest paths often traverse via them [24]. A popular method to deal with the problem is to remove pool metabolites from the metabolic network. Then, we are faced with assignment of metabolites as pool metabolites, which is a task that depends on the pathway queries we would like to ask. Taking again ATP as an example, by removing ATP from the metabolic network, we lose the opportunity to obtain results involving pathways which synthesize ATP.

There have been attempts to avoid the problem of assigning a list of side metabolites altogether in pathway analysis tasks. Rahman *et al*. utilized information on metabolite structures in Pathway Hunter Tool (PHT) [16]. In PHT, metabolite structural similarity is first used to identify reactant pairs in reactions which resemble each other most. Path finding is then performed on a graph consisting only of reaction connections between the most similar metabolites. For instance, in reaction glucose + ATP → glucose 6-phosphate + ADP one can find out that glucose and ADP do not resemble each other, and thus no pathway should use the connection glucose → ADP. Of course, the method is dependent on molecular structure data. For some metabolites such data is not available. However, exact atom mappings are not required, and it is possible to approximate molecular similarity with an appropriate heuristic.

A method has been proposed by Croes *et al*. to avoid side metabolites in path finding by weighting the edges of the metabolic network graph by the metabolite degree [25]. In this method, Metabolic PathFinding, highly connected metabolites such as pool metabolites receive a high weight. Therefore, when searching for the lightest paths between the query metabolites, the method tends to generate pathways that avoid pool metabolites. It is still possible for a pool metabolite to appear in a result pathway as it has not been removed from the network. Moreover, some metabolites such as pyruvate have a high degree without appearing as side metabolites in most reactions. As a downside of the method, routes through such metabolites will be penalized.

Blum and Kohlbacher combined atom mapping information with metabolic graphs weighted by metabolite degrees [26]. In their approach, k lightest linear paths are first sought for in a graph corresponding to a metabolic network, where two metabolites are connected if there is a reaction where at least one atom is transferred between the metabolites. To this end, the authors computed atom mappings for a set of reactions using a minimum cut algorithm. The quality of atom mappings was then improved by taking into account the structure of the EC hierarchy. Finally, when a set of pathways had been found between the query metabolites, a check would be made to ensure that at least one atom is actually transferred from source to target. The method has been implemented as the tool MetaRoute [18].

Subsequently, also Metabolic PathFinding was improved by Faust *et al*. by taking advantage of the annotations for reactant pairs in KEGG reactions contained in the RPAIR database [19]. RPAIR describes how the atoms are transferred from a substrate to a product in reactions. Moreover, each reactant (substrate-product) pair has been annotated with the inferred role of the reactant pair in each reaction [27]. Roles include "main", "trans", "cofac", "ligase" and "leave". Pairs assigned as "ligase" and "leave", often indicate a connection irrelevant to typical path finding queries. For instance, in reaction glucose + ATP → glucose 6-phosphate + ADP, reactant pairs (glucose, glucose 6-phosphate) and (ATP, ADP) have been annotated as "main" pairs while (glucose 6-phosphate, ATP) is a "trans" pair. Since no atoms are transferred from glucose to ADP, this reactant pair has not been annotated.

Faust *et al*. evaluated the accuracy of different combinations of path finding parameters in retrieving 55 known reference pathways in three organisms (*E. coli*, *S. cerevisiae*, *H. sapiens*) [19]. Different method variants were constructed, for instance, by choosing whether to remove highly connected metabolites, whether to assign weights to metabolites according to their degree and whether to assign weights to reactions according to their annotated role in RPAIR by favoring "main" pairs. The extensive statistical testing performed by the authors demonstrated that the inclusion of RPAIR annotations together with metabolite weighting improves the path finding results significantly.

The graph-theoretical methods discussed above only find non-branching pathways. As many important pathways are best understood by considering the different branches leading to the target metabolite, it can be argued that a metabolic path finding method should incorporate support also for non-linear pathways. Some methods, such as MetaRoute [18] offer the possibility to view a graphical representation of the combination of the linear paths leading from source to target. However, in such representations unrelated linear paths may be shown together, making drawing conclusions about the branching pathway structure more difficult.

These concerns can be addressed in constraint-based modelling framework. A prominent concept in this framework is *elementary flux mode *(EFM). An elementary flux mode is a minimal set of enzymes capable of operating in a pseudo steady-state, with reactions respecting irreversibility constraints [13]. Elementary flux modes have proven to be useful in analysis of small to medium-scale metabolic models [28]. In particular, EFMs can accurately describe the branching nature of many pathways, such as pentose phosphate pathway.

Without additional constraints, both graph-theoretical and constraint-based methods produce a very large number of pathways for typical queries. For instance, about 500000 linear pathways of length at most nine reactions were found from glucose to pyruvate [29], while roughly the same number of elementary flux modes were found in a metabolic network of 110 reactions connecting glucose, acetyl, glycine and succinate to CO_2_, acetyl, formate, ethanol and lactose [30]. In particular, the large number of EFMs in typical settings has prohibited the analysis of genome-scale models [22]. To analyze a genome-scale model with EFMs in a constraint-based modelling framework, one effectively needs to limit the complexity of the model, either reducing the model size or imposing constraints. A particular problem is how to assign the external metabolites which are excluded from the pseudo steady state constraint. Of course, this task depends on the intended use of the model. Popular tools which can be used to compute elementary flux modes include METATOOL [31] and YANA [32].

A recent case study [33] compared path finding approaches to elementary flux mode analysis in producing sugars from fatty acids. In that work, the system under study was relatively small, consisting of the reactions of the Krebs cycle, glycolysis and gluconeogenesis. The question asked was whether it was possible for the system to produce glucose 6-phosphate (G6P) from acetyl-CoA (AcCoA). Two different models were considered. First, a model with no glyoxylate cycle was demonstrated to not to be able to perform the desired conversion in steady-state. Second, when the glyoxylate cycle was added, a steady-state conversion from AcCoA to G6P was possible. Then, the authors queried two methods, Metabolic PathFinding [15] and Pathway Hunter Tool [16], for paths connecting AcCoA to G6P. The methods failed, according to the authors, to provide realistic pathways corresponding to the steady-state pathways found by elementary flux mode analysis. In particular, many resulting pathways did not carry any carbon net flux, a necessary property of a biosynthetic pathway. However, the authors criticize the pathways found by PathFinding and PHT for not necessarily being balanced at steady-state. This can be argued against in a general path finding setting, as biologically important but unbalanced metabolic pathways exist [20]. Moreover, an unbalanced pathway might be important in its own right, demonstrating a mechanism for the net carbon flow, for instance.

In previous work, we introduced the concept of *feasible pathways *[17]. In this graph-theoretical approach, the metabolic network is viewed as an and-or graph where and nodes correspond to reactions and or nodes correspond to metabolites. A feasible pathway is a set of reactions where each reaction is *reachable *from a set of source metabolites. Two procedural rules define reachability of reactions and metabolites: a reaction can be made reachable if and only if all its substrates have been made reachable, while a metabolite can be made reachable if and only if either at least one of reactions producing has been made reachable or the metabolite is a source metabolite. In other words, a feasible pathway is branching when there is a reaction with two or more substrates. In this approach, pool metabolites are dealt with by removing them from the network before analysis.

### Our contribution

In this paper, we introduce a new graph-theoretical method, *ReTrace*, for finding branching pathways in large-scale metabolic networks. Our method builds on the observation utilized in the previous works of Arita [14] and Blum and Kohlbacher [26] that a biologically interesting pathway should transfer at least one atom from source to target metabolite.

Our method tries to overcome the problem of irrelevant connections faced by most path finding approaches by searching for pathways at atom level instead of reaction-metabolite level. ReTrace searches for pathways in an atom-level representation of the metabolic network in contrast to most other path finding methods discussed above. Particularly, the method improves Arita's ARM method [14] by being able to find branching pathways that transfer as many as possible of the atoms in the target metabolite from precursors. To our best knowledge, this is the first path finding method which explicitly tries to maximize this quantity. Favoring pathways which transfer as many atoms as possible can be justified by considering a pathway that fails to transfer all target metabolite atoms. In order to operate, such pathway necessarily involves reaction or reactions, which bring the missing atoms into the pathway from *dangling *substrates. Specifically, a dangling substrate is a metabolite consumed but not produced by a reaction on the pathway. The number of dangling substrates invariably decreases, as more atoms are transferred to the target metabolite by the pathway. We argue that to find plausible pathways, a path finding method should either minimize the number of dangling substrates or transfer as many atoms as possible to target. Particularly, ReTrace is designed to do both at the same time.

To this end, we introduce a scoring function for pathways taking into account the number of target atoms transferred from source atoms. We then formulate the problem of finding high-scoring branching pathways, study its complexity and give an efficient algorithm to solve the problem. The algorithm operates at the atom-level representation, finding a number of shortest paths [34,35] between source and target metabolites and combining paths into branching, high-scoring pathways. Consequently, we retain the scalability of other graph-theoretical path finding methods and thus enable the analysis of genome-scale metabolic networks without any model restriction done prior to analysis.

In particular, our method avoids the context-dependent problem of defining side metabolites in each reaction. For instance, ATP is a typical side metabolite that participates in a large number of reactions. However, as discussed earlier, by removing ATP from analysis we at the same time lose the possibility to analyze the ATP synthesis pathways. Operating in an atom-level representation of the metabolic network, ReTrace disregards side metabolite connections automatically being unable to transfer atoms from source to target metabolite.

The ability to incorporate measurement data into analysis is vital in discovering condition-specific pathways. In order to achieve this, in addition to the scoring function discussed above, ReTrace allows scores to be assigned to reactions. Pathway search is then guided by the scores to favor pathways which utilize reactions with high scores. Thus, by encoding measurement data into reaction-specific scores, one can obtain pathways which were active in the measured conditions, for instance. In this paper, we describe an application where we incorporated sequence similarity data into ReTrace analysis [36]. In general, one can consider incorporation of data from other sources, such gene or protein expression data, or enzyme function prediction [37] data obtained with machine learning methods.

We have implemented the method in Python [38]. The software is released under GPL and is freely available for academic purposes.

## Methods

We begin by defining some key concepts, a scoring function for pathways and a computational problem of finding branching, high scoring pathways. Then, we describe an algorithm, ReTrace, for solving the path finding problem.

First, we define ℳ to be a collection of metabolites and ℛ to be a collection of reactions which utilize the metabolites in ℳ. A metabolic reaction *r *∈ ℛ is defined by giving its substrate and product metabolite sets *I*(*r*), *O*(*r*) ⊆ ℳ, respectively. For instance, the reaction glucose (GLC) + ATP → glucose 6-phosphate (G6P) + ADP could be given by *I*(*r*) = {GLC, ATP} and *O*(*r*) = {G6P, ADP}. Each metabolite *m *∈ ℳ consists of atoms, *B*(*m*) ⊆ , where  is the set of atom positions over all metabolites. The atoms in different metabolites are distinct. For example, we could assign six (carbon) atoms to both GLC and G6P, and let *B*(GLC) = {*a*_1_,..., *a*_6_} and *B*(G6P) = {*a*_7_,..., *a*_12_}. We deal with bidirectional reactions by considering the reverse direction as a separate reaction , e.g., *I*() = *O*(*r*) and *O*() = *I*(*r*). Unidirectional reactions are then naturally taken into account by leaving the reverse reaction out of the model.

Further, we define the *atom mapping *[39] for a reaction *r *∈ ℛ to be a relation Γ: ℛ →  ×  specifying how substrate atoms are transferred to product atoms. To give an example, let  = {*a*_1_, *a*_2_, *a*_3_, *a*_4_} and ℳ = {*m*_1_, *m*_2_, *m*_3_}, with metabolite structures defined by *B*(*m*_1_) = {*a*_1_, *a*_2_}, *B*(*m*_2_) = {*a*_3_} and *B*(*m*_3_) = {*a*_4_}. We can now define a reaction *r *with *I*(*r*) = {*m*_1_} and *O*(*r*) = {*m*_2_, *m*_3_} and the atom mapping associated with *r *to be Γ_1_(*r*) = {(*a*_1_, *a*_3_), (*a*_2_, *a*_4_)}. Alternatively, atom mapping can be defined as Γ_2_(*r*) = {(*a*_1_, *a*_4_), (*a*_2_, *a*_3_)}. We say that the reaction *r consumes *atoms  (*r*) = {*a*|(*a*, *a'*) ∈ Γ (*r*)} and *produces *atoms  (*r*) = {*a'*|(*a*, *a'*) ∈ Γ (*r*)}.

**Figure 1:**
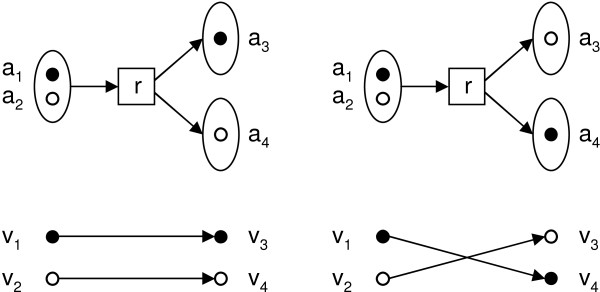
**Example atom mappings**. Example reaction *r*: *m*_1 _→ *m*_2 _+ *m*_3_, two alternative atom mappings Γ_1_(*r*) = {(*a*_1_, *a*_3_), (*a*_2_, *a*_4_)} (left) and Γ_2_(*r*) = {(*a*_1_, *a*_4_), (*a*_2_, *a*_3_)} (right) and corresponding atom graphs *G*({*r*}) for atom mappings Γ_1_, Γ_2_. Atom mappings indicated by shading of atoms. The reaction consumes atoms  (*r*) = {*a*_1_, *a*_2_} and produces atoms  (*r*) = {*a*_3_, *a*_4_}.

For a collection of reactions ℛ with associated atom mappings Γ, we define a directed *atom graph G *(ℛ, ) = (*V*, *E*), induced by atom collection  and atom mappings Γ with vertices *V *and edges *E *as follows. For each atom *a *∈ *A*, we have a vertex *v*_*a *_∈ *V*. Each pair (*a*, *a'*) ∈ Γ (*r*), *r *∈ *R*, corresponds to an edge (*v*_*a*_, *v*_*a'*_) ∈ *E*. Note that if reaction directionality is not explicitly constrained, the atom graph will contain the edge (*v*_*a'*_, *v*_*a*_) for each edge (*v*_*a*_, *v*_*a'*_) due to reverse reactions being treated as separate reactions. The example reaction *r*, atom mappings Γ_1_(*r*), Γ_2_(*r*) and the induced atom graphs are shown in Figure 1.

In this section, we discuss only reactions with *simple *or *0-1 stoichiometry*, that is, reactions where each substrate and product either appears exactly once or does not appear in *I*(*r*) and *O*(*r*), respectively. Briefly, one can relax this assumption by replicating atoms and involved edges in metabolites which appear in a reaction more than once but this topic is not pursued further here. For a reaction *r *involving only simple stoichiometries, we assume the relation Γ(*r*) to be bijective, i.e., there is an one-to-one correspondence between substrate and product atoms. However, if a reaction consumes or produces two units of a metabolite, for example, there is necessarily an atom that is either mapped from or mapped to more than once. As an example, consider the reaction *m*_1 _→ 2*m*_2 _having Γ(*r*) = {(*a*_1_, *a*_3_), (*a*_2_, *a*_3_)} with atom *a*_3 _of metabolite *m*_2 _appearing twice in the relation. Hence, atom mappings of reactions involving stoichiometries different than zero or one, are not bijective.

**Figure 2:**
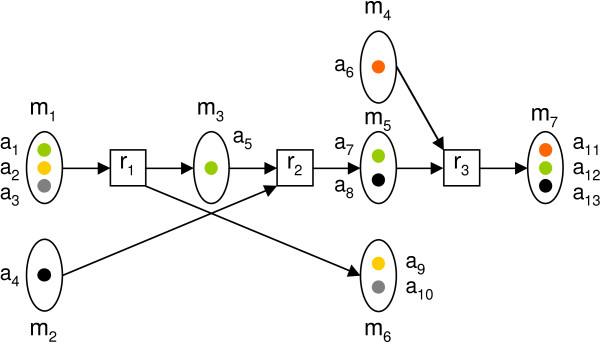
**Example pathway of three reactions**. Example pathway of three reactions *r*_1_, *r*_2 _and *r*_3 _and seven metabolites *m*_1_,..., *m*_7 _containing 13 atoms in total. Atom coloring indicates how the atoms are mapped in reactions. For instance, the pathway transfers atom *a*_1 _to atoms *f*_*P *_({*a*_1_}) = {*a*_5_, *a*_7_, *a*_12_} and atom *a*_8 _to atom *f*_*P *_({*a*_8_}) = {*a*_13_}.

A subset *P *= {*r*_1_,..., *r*_*k*_} ⊆ ℛ is called a *pathway*. A pathway *transfers *an atom *s *∈  to an atom *t *∈  if and only if there is a path *s *→ *t *in the atom graph *G*(*P*). We denote by *f*_*P *_(*S*) the set of atoms to which atoms in *S *are transferred by the pathway *P*. Figure 2 shows examples of *f*_*P *_(*S*) in the context of a small example pathway.

As discussed in the previous section, metabolic pathways which transfer a large fraction of their source atoms to product atoms should be prioritized over pathways transferring a lesser number of atoms. In particular, we are interested specifically in carbon atoms, as their supply is often the limiting factor in cellular functions. Yields of other atoms such as nitrogen or sulfur may also be of importance, depending on the role of the pathway in metabolism.

**Figure 3:**
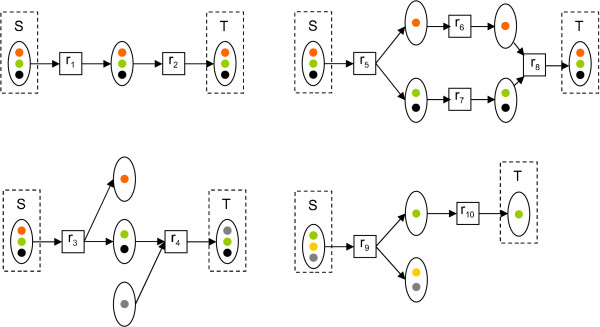
**Pathway examples**. Examples of four pathways and associated *Z*_*O *_scores. Assignment of sets *S *and *T *are indicated with dashed boxes and atom mappings with atom colorings in figure. Top left: pathway *P*_1 _= {*r*_1_, *r*_2_} transfers all three atoms in *S *to *T*, achieving *Z*_*O *_(*P*_1_, *S*, *T*) =  = 1. Bottom left: pathway *P*_2 _= {*r*_3_, *r*_4_} transfers green and black atoms to *T*. Since grey atom is not in *S*, *Z*_*O *_(*P*_2_, *S*, *T*) = . Top right: branching pathway *P*_3 _= {*r*_5_, *r*_6_, *r*_7_, *r*_8_} transfers all atoms in *S*, resulting in *Z*_*O *_(*P*_3_, *S*, *T*) = 1. Bottom right: pathway *P*_4 _= {*r*_9_, *r*_10_} transfers the only target atom from *S*, giving *Z*_*O*_(*P*_4_, *S*, *T*) = 1. However, two atoms of *S *are not transferred to *T*.

To take these considerations into account, we define a scoring criterion for pathways, given source and target atoms, *S*, *T *⊆ , respectively. For instance, source and target atoms can be defined as sets of single atoms, all atoms in some metabolites, or all carbon atoms in some metabolites. If a metabolite contains atoms in the source or target atom set, we denote it as a *source *or *target metabolite*, respectively. We define our pathway scoring criterion as the fraction *Z*_*O *_of target atoms *T *which are transferred by the pathway *P *from source atoms *S*,

A pathway with *Z*_*O*_(*P*, *S*, *T*) = 1 is called a *complete pathway*. Intuitively, a pathway with high *Z*_*O *_is able to produce a large fraction of target atoms from source atoms, and does not require a high contribution from atoms in other than source metabolites. Figure 3 shows four example pathways and *Z*_*O *_scores corresponding to them.

It is easy to see that *Z*_*O *_is maximized on the pathway *P *= ℛ. Instead, we study the following problem where the pathway size is involved.

### Computational Problem 1

(Find-Branching-Pathways). *Given a set of reactions *ℛ, *sets S, T *⊆ , *l *∈ ℤ^+ ^*and w ∈ *ℝ, *find all pathways P ⊆ *ℛ *such that Z*_*O*_(*P*, *S*, *T*) ≥ *w and *|*P*| <*l*.

In Find-Branching-Pathways, we aim to find pathways that transfer enough atoms from sources to targets while using less than some specified amount of reactions. It is possible that a pathway *P *is branching, or non-linear. For instance, the top right pathway in Figure 3 illustrates a simple branching pathway. It is easy to see that Find-Branching-Pathways is computationally hard by considering a related problem, Find-Minimal-Pathway, where we try to find the pathway *P *with minimal size such that *Z*_*O*_(*P*, *S*, *T*) ≥ *w*.

### Computational Problem 2

(Find-Minimal-Pathway). *Given an instance of a Find-Branching-Pathways problem, find a pathway P satisfying the constraints of Find-Branching-Pathways such that *|*P*| ≤ |*P'*| *for any other pathway P' satisfying the constraints*.

#### Lemma 1. *Find-Minimal-Pathway is NP-hard*

*Proof*. We show that Find-Minimal-Pathway is NP-hard [40] by a reduction from the Minimum-Set-Cover problem. Let  be a collection of subsets of a finite set . In Minimum-Set-Cover, we seek the minimal subset  ⊆  such that every element in  belongs to at least one member in . Now, we construct an instance of the Find-Minimal-Pathway problem as follows. For each element *s*_*i *_∈ , we assign two atoms *a*_*i*_,  into *A*. We then assign a reaction *r*_*i *_to *R *for each subset *C*_*i *_∈  such that Γ(*r*_*i*_) = {(*a*_*j*_, )|*s*_*j *_∈ *C*_*i*_}. Finally, we set *S *= {*a*_*j*_|*s*_*j *_∈ *C*_*i*_, *C*_*i *_∈ }, *T *= {|*s*_*j *_∈ *C*_*i*_, *C*_*i *_∈ } and *w *= 1. A solution to Minimum-Set-Cover can be reconstructed in polynomial time from the solution to Find-Minimal-Pathway by assigning *C*_*i *_∈  for each *r*_*i *_∈ *P*. As Minimum-Set-Cover is NP-complete [40], it follows that Find-Minimal-Pathway is NP-hard.   □

**Figure 4:**
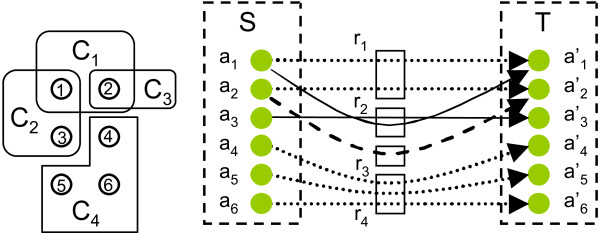
**Reduction of Minimum-Set-Cover to Find-Minimal-Pathway**. Left: a minimal set cover instance with  = {*s*_1_, ..., *s*_6_} (circles) and subset collection  = {*C*_1_, *C*_2_, *C*_3_, *C*_4_}. Right: Find-Minimal-Pathway instance corresponding to the set cover instance with 12 atoms and four reactions. Arrows denote mapping of atoms Γ over reactions. In particular, mappings shown with similarly dashed arrow lines belong to the same reaction. Source and target atom sets indicated with *S *and *T*.

Subsequently, we can conclude that the original problem Find-Branching-Pathways is NP-hard, because we can solve Find-Minimal-Pathway by finding the (potentially exponential number of) solution pathways to Find-Branching-Pathways and choosing the smallest pathway. Figure 4 illustrates how a small minimum set cover instance is reduced into an instance of the Find-Minimal-Pathway problem.

#### Corollary 1. *Find-Branching-Pathways is NP-hard*   □

When solving Find-Branching-Pathways in practice, we would like to benefit from information from other sources to help us evaluate the pathways found. As an important example, we discuss the use of genome-level evidence in the pathway context in section Implementation.

### Algorithm

Due to the computational complexity of the Find-Branching-Pathways problem, we develop next an efficient heuristic algorithm for it.

First, we observe that to maximize *Z*_*O *_we should seek a pathway that is able to transfer an atom to each target atom from source atoms. Further, to achieve a pathway smaller than *l *reactions, we should prefer reactions which are able to transfer many atoms at the same time. To illustrate this, let us first define a *reaction path p *as a sequence of reactions *p *= (*r*_1_, *r*_2_,..., *r*_*n*_), *r*_*i *_∈ *R*, such that there is a path from an atom *s *consumed by the first reaction, *s *∈ (*r*_1_), to an atom *t *produced by the last reaction, *t *∈  (*r*_*n*_), in the atom graph induced by the reactions in *p*. Clearly, such reaction path is able to transfer at least the atom *s *to atom *t *but the path may be to transfer additional atoms as well. Analogously to the definition of a pathway, we denote by *f*_*p*_(*S*) the set of atoms to which atoms in *S *can be transferred by the reaction path *p *in this fashion. As more source atoms are transferred via a reaction path to targets, higher *Z*_*O *_is achieved. Thus, we observe that reaction paths with large *f*_*p*_(*S*) sets should be preferred.

**Figure 5:**
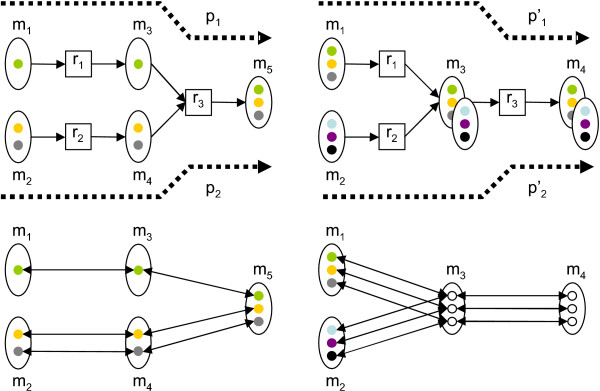
**Reaction and metabolite junctions**. Top left: atoms in metabolites *m*_1 _and *m*_2 _are transferred to atoms in metabolite *m*_5 _via two reaction paths *p*_1 _= (*r*_1_, *r*_3_) and *p*_2 _= (*r*_2_, *r*_3_) (indicated by dashed arrows). The paths merge in reaction *r*_3_. Pathway *P *consisting of reactions *r*_1_, *r*_2_, *r*_3 _has *Z*_*O *_(*P*, *S*, *T*) = 1, when atoms of {*m*_1_, *m*_2_} and {*m*_5_} comprise the source and target sets *S *and *T*, respectively. Top right: pathway *P' *= {*r*_1_, *r*_2_, *r*_3_} achieves *Z*_*O *_(*P'*, *S, T*) = 1 assuming source and target atoms to be all atoms in {*m*_1_, *m*_2_} and {*m*_4_}, respectively. The two reaction paths transferring the atoms,  = (*r*_1_, *r*_3_) and  = (*r*_2_, *r*_3_) merge in metabolite *m*_3_. Subsequently, atoms from *m*_1 _and *m*_2 _are never transferred to the same instance of metabolite *m*_3 _via these paths. Bottom left and right: atom graph representations of pathways *P *(left) and *P' *(right). Hollow circles denote atoms which can originate from atoms in metabolites *m*_1 _or *m*_2_.

Second, we are able to achieve higher *Z*_*O *_with combinations of pathways. Consider a pathway *P *with *Z*_*O*_(*P*, *S*, *T*) < 1. Now, since not all target atoms can be transferred from sources, there must be a target atom *t *∈ *T *which is not connected by a path from any source atom in the atom graph *G*(*P*). We should thus consider an addition of a pathway *P' *such that a path *s *→ *t *exists in pathway *P *∪ *P' *from some *s *∈ *S*. To minimize the size of the combined pathway, we should utilize reactions already on the pathway *P*, and find an addition that is able to provide the target atoms not already transferred. In particular, we should look for branching points on the pathway *P *where an atom *u *in one or more substrates is not already connected to the sources but there is a path *u *→ *t *in *G*(*P*). In such case, connecting a source atom to *u *by an addition of a pathway *P' *such that *u *∈ *f*_*P'*_(*S*) guarantees that *Z*_*O*_(*P *∪ *P'*, *S*, *T*) > *Z*_*O*_(*P*, *S*, *T*). However, it should be noted that certain path combinations yield pathways, where necessarily more than one instance of target molecule is produced. Consider the two pathways illustrated in Figure 5. In the pathway on the left, two reaction paths merge in a *reaction junction*. A reaction junction occurs, when two paths share a reaction (*r*_3 _in Figure 5) but the reactions preceding the shared reaction (*r*_1_, *r*_2_) do not share any product metabolites. The shared reaction is then a reaction junction. In the example presented in Figure 5, atoms in products of *r*_1 _and *r*_2_, *m*_3 _and *m*_4 _respectively, are transferred to the same molecular instance of metabolite *m*_5_.

On the other hand, in the pathway on the right, the two paths meet in a *metabolite junction m*_3_. A metabolite junction is a metabolite shared by two paths such that both paths contain the same reaction consuming the metabolite. In Figure 5, metabolite *m*_3 _is the shared product of *r*_1 _and *r*_2_. The atoms in substrates of *r*_1 _and *r*_2 _are in this case never transferred to the same instance of *m*_3_: Instead, the atoms always end up in different molecules of *m*_4_. Although the pathway is unable to provide all source atoms in the same target molecule, the score *Z*_*O *_does not differentiate between these two cases. We would, however, like to avoid pathways with metabolite junctions, because such pathways in reality can be split into two or more pathways, each transferring source atoms to a single instance of the target metabolite.

These ideas lead into the following recursive algorithm, ReTrace, where we find paths leading to target metabolites from sources in the atom graph, and combine these paths to achieve a higher *Z*_*O *_score. To discover alternative pathways, a number of paths are generated at each recursion level. Furthermore, in order to to reduce occurences of metabolite junctions, ReTrace only considers combinations which add reaction junctions to the pathway.

**Figure 6:**
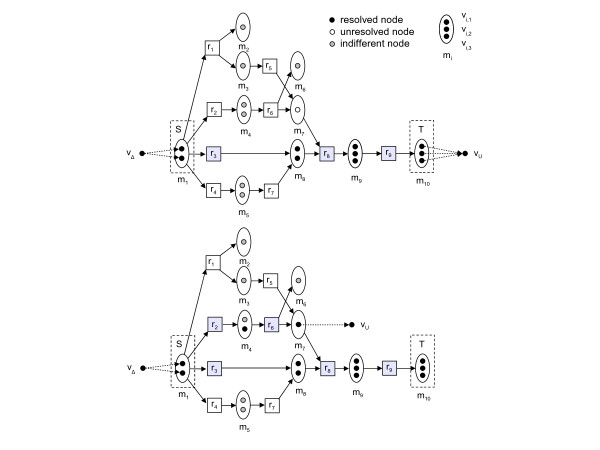
**ReTrace example run**. Example ReTrace run for query *m*_1 _→ *m*_10 _with *k *= 3 in a database of 9 reactions and 10 metabolites. Atoms numbered from top to bottom as shown in figure. Dashed arrows indicate edges connecting *v*_Δ _and *v*_*U *_to atom nodes. Otherwise atom graph edges are not drawn; instead, arrows indicate substrates and products in reactions and atoms are mapped in linear fashion. For example, in reaction *r*_9_, atom nodes *v*_7,1_, *v*_8,1 _and *v*_8,2 _are connected to nodes *v*_9,1_, *v*_9,2 _and *v*_9,3_, respectively. At first, *U *= {*m*_10,1_, *m*_10,2_, *m*_10,3_} are the unresolved nodes. Top: algorithm state after first call to Procedure FindPath. The three shortest atom paths found are  = (*v*_Δ_, *v*_1,1_, *v*_8,1_, *v*_9,2_, *v*_10,2_, *v*_*U*_),  = (*v*_Δ_, *v*_1,2_, *v*_8,2_, *v*_9,3_, *v*_10,3_, *v*_*U*_) and  = (*v*_Δ_, *v*_1,2_, *v*_4,2_, *v*_7,1_, *v*_9,1_, *v*_10,1_, *v*_*U*_), with path length ties broken arbitrarily. Choosing to process  first, the reaction set corresponding to the atom path is *P' *= {*r*_3_, *r*_8_, *r*_9_}. Tracing back from *v*_*U*_, ReTrace finds that *v*_10,2 _and *v*_10,3 _can be traced back to *v*_Δ_, while *v*_7,1 _is added to *U*. Procedure FindPath is then called recursively. Bottom: algorithm state after second call to Procedure FindPath. Edges to *v*_*U *_are updated to reflect *U *= {*v*_7,1_}. Shortest paths from *v*_Δ _to *v*_*U *_are computed. However, only two paths are found:  = (*v*_Δ_, *v*_1,2_, *v*_4,2_, *v*_7,1_, *v*_*U*_) and  = (*v*_Δ_, *v*_1,2_, *v*_3,1_, *v*_7,1_, *v*_*U*_). Choosing arbitrarily  to process next, ReTrace finds out that the reaction set *P' *= {*r*_2_, *r*_6_} resolves the remaining atom in *U *and a complete pathway {*r*_2_, *r*_3_, *r*_6_, *r*_8_, *r*_9_} has been discovered.

The description of the ReTrace method is divided below into Procedures ReTrace, FindPath and FindPathStart, where Procedure ReTrace (Table 1) prepares an atom graph instance, Procedure FindPath (Table 2) solves Find-Branching-Pathways recursively and Procedure FindPathStart (Table 3) finds the first node of the given path. The operation of ReTrace is explained with a small example in Figure 6. Procedure ReTrace first constructs the atom graph *G *induced by reactions *R*. Nodes *v*_Δ _and *v*_*U *_which represent all source and *unresolved *atoms are added to the graph. An atom node *u *is unresolved, if there is no path *v*_Δ _→ *u *on the pathway and addition of a such path would create a path *v*_Δ _→ *t *to some *t ∈ T *for which no path already existed. Each target atom is considered at this step an unresolved node. Subsequently, the algorithm attempts to *resolve *the unresolved nodes by adding paths to the pathway. Additional edges are added to *G *to connect *v*_Δ _to source atoms. Finally, Procedure FindPath is called, which recursively adds paths to the pathway until all nodes have been resolved or there are no more paths to add which would resolve nodes.

**Table 1:** Procedure ReTrace

**ReTrace **(ℛ, *S*, *T*, *w*, *k*, *l*):	
**Input**: reactions *R*, sources *S*, targets *T*, 0 <*w *≤ 1 and *k*, *l *∈ ℤ^+^	
1:	*G ← G*(*R*)
2:	Add nodes *v*_Δ _and *v*_*U *_to *G*
3:	Add edges (*v*_Δ_, *v*_*s*_) for each *s *∈ *S *to *G*
4:	*P *← ∅ % Current pathway
5:	*U *← *T *% Unresolved nodes
6:	FindPath(*G*, *P*, *v*_Δ_, *U*, *w*, *k*, *l*)

**Table 2:** Procedure FindPath

**FindPath**(*G*, *P*, *v*_Δ_, *U*, *w*, *k*, *l*):	
**Input**: atom graph *G*, current pathway *P*, source node *v*_Δ_, unresolved nodes *U*, 0 <*w *≤ 1 and *k*, *l *∈ ℤ^+^	
1:	Add edges (*v*_*u*_, *v*_*U*_) for each *u ∈ U *to *G*
2:	**Q **← FindKShortestSimpleAtomPaths(*G*, *k*, *v*_Δ_, *v*_*U*_)
3:	Remove edges (*v*_*u*_, *v*_*U*_) for each *u ∈ U *from *G*
4:	**for all ***Q *∈ **Q do**
5:	*P' *← {*r *∈ Γ^-1 ^(*q*)|*q *∈ *Q*} % Reactions involved with atom path *Q*
6:	**if ***Z*_*O *_(*P *∪ *P'*, *S*, *T*) ≥ *w *and |*P *∪ *P'*| <*l ***then**
7:	Found a solution pathway *P *∪ *P'*
8:	*U' *← ∅
9:	**for all ***u *∈ *U ***do**
10:	*v *← FindPathStart(*P'*, *u*)
11:	**if ***v *≠ *v*_Δ _**then**
12:	*U' *← *U' *∪ {*v*}
13:	**if ***U' *≠ ∅ **then**
14:	FindPath(*G*, *P *∪ *P'*, *v*_Δ_, *U'*, *w*, *k*, *l*)

**Table 3:** Procedure FindPathStart

**FindPathStart **(*P'*, *u*):	
**Input**: path *P' *= (*p*_1_,..., *p*_*x*_), node *u*	
1:	*i *← *x*
2:	**while ***i *> 0 **do**
3:	*u *← *v *∈ {*v *| (*u*, *v*) ∈ Γ (*p*_*i*_)}
4:	*i *← *i *- 1
5:	Return *u*

In Procedure FindPath, the graph is first set up to reflect the current set of unresolved nodes by adding edges (*v*_*u*_, *v*_*U*_) for each *u *∈ *U *. Then, the algorithm computes *k *shortest *simple paths *from *v*_Δ _to *v*_*U *_in the atom graph *G *by calling the subroutine FindKShortestSimpleAtomPaths. A simple (or acyclic) path is a path where no node is repeated. To implement FindKShortestSimplePaths, any algorithm developed for the *k *shortest simple path problem [35] can be used. For instance, Yen's algorithm takes *O*(*kn*(*m *+ *n *log *n*)) time to compute *k *shortest simple paths in a graph with *n *nodes and *m *edges [34]. After the shortest paths have been computed, the graph is restored by deleting the edges added in step 1. By finding paths from *v*_Δ _to *v*_*U*_, ReTrace aims to discover additions to the current pathway *P *which would increase the *Z*_*O *_score. Further, the additions should be as small as possible because of the maximum pathway size constraint. To this end, ReTrace computes explicitly simple paths in contrast to finding shortest paths where cycles are allowed: a cyclic path can always be transformed into a smaller acyclic path which transfers the same set of atoms from sources to target as the cyclic path.

Note that algorithms for the more general case of computing of *k *shortest paths which are allowed to be cyclic, are considerably faster than algorithms for computing only the simple paths. For instance, the running times of Eppstein's algorithm [35] and REA [41] are *O*(*m *+ *n *log *n *+ *k *log *k*) and *O*(*m *+ *kn *log ()), respectively. However, because the atom graphs considered in this study contain cycles, we would have to remove cyclic paths from the pathways returned by the *k *shortest path algorithm. Furthermore, to compensate for the cyclic pathways generated, we would have to increase the number of shortest paths computed at each step according to the expected number of acyclic paths. Due to these considerations, we have adopted Yen's algorithm in our current implementation of ReTrace.

Then, the reactions involved in the shortest atom path *Q *are assigned as the reaction set *P' *in step 5. Function Γ^-1 ^:  ×  → ℛ gives each atom graph edge the reaction that induced the edge. Each set *P' *is considered as an addition to the current pathway *P *in steps 5-14. If the combined pathway *P *∪ *P' *meets the *Z*_*O *_and pathway size requirements, and it has not been already generated, it is reported as a result. The computation of *Z*_*O *_score of the current pathway in step 6 can be done in *O*(|*P *∪ *P'*|) = *O*(*n*) time by breadth-first search in the atom graph *G*. Next, the algorithm considers the atoms which remain unresolved after addition of path *P'*. In steps 9-12, the algorithm backtracks from each atom *u ∈ U *using the Procedure FindPathStart and checks whether path *P' *transfers a source atom to *u*. If this is not true, the atom *u *is an unresolved atom.

Lastly, in step 14, FindPath is called recursively to find additions to the pathway *P *∪ *P' *by looking for paths from *v*_Δ _to the new set of unresolved nodes *U '*. The recursion depth is at most |*T*|, because during each call at least one unresolved atom is resolved, at most one atom is added to the set *U' *per atom *u *∈ *U *and *U *= *T *at depth 1. As a maximum of *k *paths is generated in each FindPath call, and FindPath is recursively called at most once for each, in total FindPath is called *O*(*k*^|*T*|^) times.

#### Lemma 2

*ReTrace takes O*(*k*^|*T*|^(*n*(*m *+ *n *log *n*) + |*T*|)) *time, assuming Yen's k shortest simple paths algorithm in step 2 of Procedure FindPath*.   □

## Implementation

We have implemented ReTrace in Python [38] as a command-line program. The software is freely available for academic use at http://www.cs.helsinki.fi/group/sysfys/software/retrace. Implementation details and user guide are available in Additional file 1 and in the above web page.

ReTrace requires that Python 2.5 has been installed. In addition, if available, ReTrace calls Graphviz *dot *tool http://www.graphviz.org/ to draw the pathway diagrams. Internet connection is recommended, because an external web program [42] is called to draw molecule structures. Finally, ReTrace assumes that a local install of the KEGG LIGAND database in flatfile format is available.

Our implementation of ReTrace utilizes atom mapping data from the KEGG RPAIR database. During startup, ReTrace parses RPAIR entries and constructs an atom graph corresponding to the data. Details on atom graph construction are given in Additional file 1.

The atom graph constructed is unweighted by default. It is possible to assign weights to atom graph edges by a command line option. Three weighting schemes are currently supported: unweighted, RPAIR size-weighted and reaction score weighted. In RPAIR size-weighting, edges from substrate atoms to RPAIR nodes are assigned weight , where α is the number of atoms mapped by the RPAIR associated with the edge. Edges from RPAIR nodes to product atoms are assigned weight 0. For instance, if an RPAIR entry maps six atoms from a substrate to a product, then the six edges from substrate atoms to RPAIR nodes each receive the weight , while the edges from RPAIR nodes to product atoms receive weights 0. Thus, in RPAIR size-weighted graphs, path finding tends to favor pathways which utilize reactions where a large number of atoms are transferred at the same time.

ReTrace supports also the option of specifying arbitrary scores *C*(*r*) to reactions. The edges are assigned weights , where  is the highest-scoring reaction associated with the edge and *β *is a constant.

Therefore, a reaction having a high score causes atom graph edges corresponding to the reaction to receive a low weight, increasing chances that shortest paths visit its edges, and finally, that the reaction appears in result pathways.

Reaction scores provide a mechanism to easily incorporate measurement data to guide pathway search. In section Results, we discuss the application of ReTrace in reconstructing biosynthesis pathways on basis of genomic evidence.

**Figure 7:**
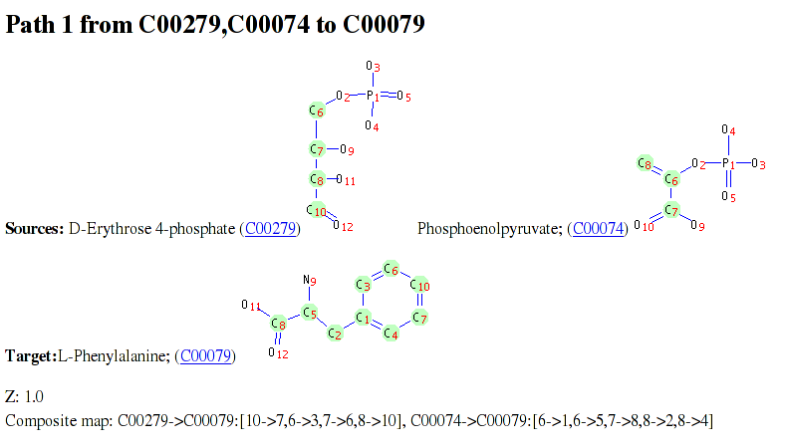
**Excerpt from ReTrace result page**. Excerpt from a html result page showing the first pathway found for the query from erythrose 4-phosphate (E4P) and phosphoenolpyruvate (PEP) to phenylalanine (Phe). Green circles in molecule structures indicate atoms in sources that the pathway transfers to target atoms. Additionally, the *Z*_*O *_score (Z) and the composite map of this pathway are shown.

**Figure 8:**
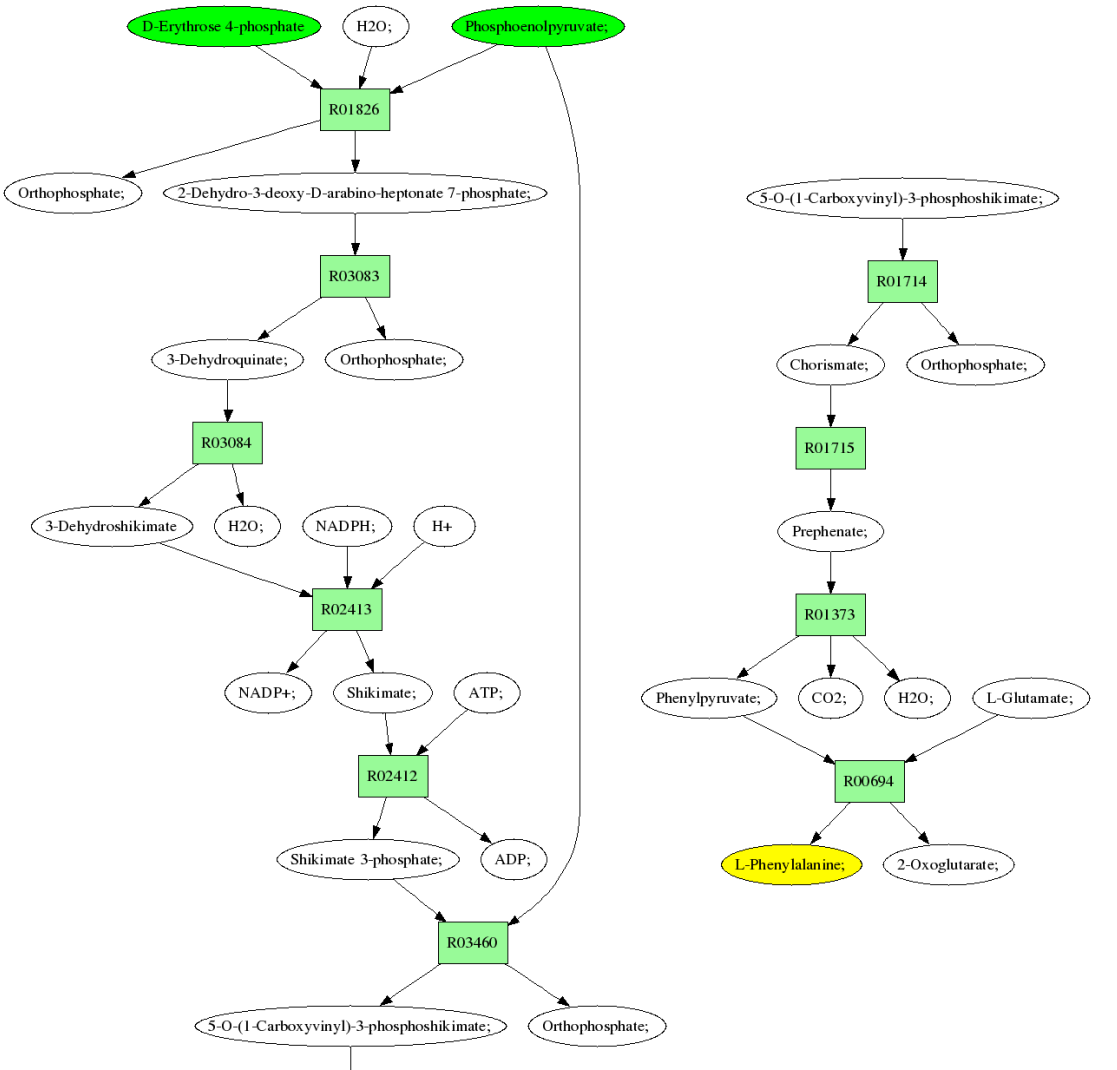
**Result pathway diagram**. Diagram of a result pathway for a query from erythrose 4-phosphate (E4P) and phosphoenolpyruvate (PEP) to phenylalanine (Phe). Source and target metabolites are drawn in green and yellow, respectively. For clarity, pathway has been split into two parts, with 5-O-(1-Carboxyvinyl)-3-phosphoshikimate repeated in both parts.

ReTrace reports query results as a set of generated html files. Figure 7 shows an excerpt from a pathway result file for a query from erythrose 4-phosphate (E4P) and phosphoenolpyruvate (PEP) to Phenylalanine (Phe). The figure shows the carbon atoms involved on the pathway as green circles. Further, results include pathway diagrams for each pathway found (Figure 8). In pathway diagrams, source and target metabolites are colored green and yellow, respectively. If reaction scores have been provided, reactions with zero score and low score are colored red and blue, respectively. Reactions with scores above a user-given score threshold are colored green.

## Results

This section is organized into four parts. First, we describe the properties of an atom graph constructed from data obtained from the KEGG database. The atom graph was used in all experiments reported in this paper. Second, we summarize results obtained in a study where amino acid biosynthesis pathways in *Trichoderma reesei *were reconstructed with ReTrace [36]. In particular, we discuss how experimental data can be incorporated into ReTrace analysis; in [36], sequence similarity data was used to guide ReTrace search. We then report results of a performance evaluation of our method. Finally, we discuss finding pathways for synthesizing inosine 5'-monophosphate (IMP) from glucose.

### Atom graph construction from KEGG RPAIR

We constructed an atom graph corresponding to 7781 reactions in the March 2009 version of KEGG LIGAND [2]. The atom graph was constructed by considering the 11265 entries in the KEGG RPAIR subdatabase. Each RPAIR entry specifies an atom mapping for a reactant pair, or substrate and product, in one or more reactions. For instance, RPAIR entry RP00001 describes the mapping of atoms between NADPH and NADP+ in those 815 reactions, where the mapping for this reactant pair is identical. A total of 140 RPAIR entries where two or more entries were found to refer to the same atom number by different type (e.g., carbon vs nitrogen) were discarded from further analysis. Unfortunately, the RPAIR data fails to map many reactions with non-1-0-stoichiometries correctly, mapping usually only one instance of reactants and leaving others unmapped. Confronted with such cases, ReTrace fails to find pathways utilizing the unmapped portions of the reactions.

**Figure 9:**
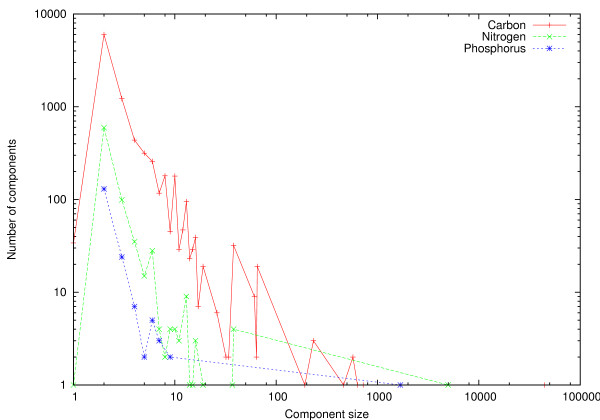
**Component sizes and numbers in atom graph**. Component size vs. the number of components in the atom graph induced by 7781 KEGG reactions. Components of carbon, nitrogen and phosphorus atoms shown separately. Both X- and Y-axes are shown in log-scale.

**Figure 10:**
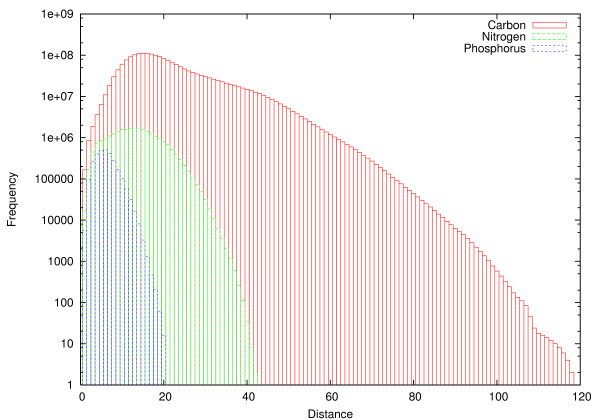
**Pairwise shortest distances in atom graph**. Pairwise distances in three subgraphs corresponding to the carbon, nitrogen and phosphorus specific mappings in the atom graph. Y-axis shown in log-scale.

The atom graph contained 90219 nodes corresponding to 80688 carbon, 7408 nitrogen and 2123 phosphorus atoms. In particular, it consisted of a large number of components, i.e., disconnected subgraphs. Figure 9 shows the distribution of component sizes in terms of number of components. The largest carbon, nitrogen and phosphorus components contained 44378, 4982 and 1684 atoms, respectively. Therefore, a significant amount of atoms were not included in these *giant components *[43], 36310, 2426 and 439 atoms for carbons, nitrogens and phosphorus, respectively. Considering a common pathway query from glucose, which completely resides in the giant carbon component in the graph constructed, a large fraction of the atom graph remains inaccessible. In particular, attempts to find carbon pathways to metabolites which do not reside in the giant carbon component entirely result in incomplete pathways. We assume that a majority of the unconnectivity observed follows from errors in KEGG RPAIR and do not pursue the issue further. We computed the pairwise shortest path distances with Dijkstra's algorithm [44] for atoms in the three subgraphs induced by atom mappings for carbons, nitrogens and phosphorus (Figure 10). Interestingly, the length of the longest shortest path between carbons was nearly 120, while lengths of nitrogen and phosphorus shortest paths were only about 40 and 20, respectively. The mean of the carbon distance distribution, however, was 21.2 (standard deviation 10.4). For nitrogen and phosphorus subgraphs respectively, the average distances were 13.1 and 6.0 with standard deviations 5.6 and 2.3. It should be noted that the data contains pairwise pathways from all components, and not only the largest components. This reduces the average path length considerably compared to the case where we studied only pathways in the giant carbon component, for example. Finally, distance distribution for the three atom types showed markedly different shape than what was observed in a previous study [45]. Particularly, in [45], the mean pairwise distance in a carbon atom graph was only 8.4, in addition to lacking the long tail showing in Figure 10.

**Figure 11:**
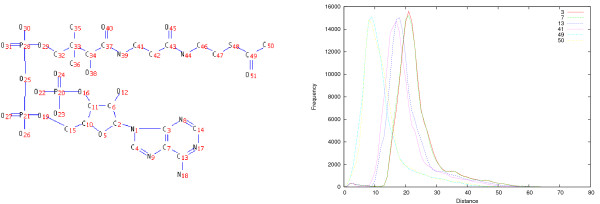
**Distances in atom graph from Acetyl-CoA**. Left: Structure and atom numbering of acetyl-CoA. Right: distances in the atom graph from acetyl-CoA carbon atoms 3, 7, 13, 41, 49 and 50. Acetyl carbons 49 and 50 display significantly shorter graph distances compared to other carbons.

Distances in the atom graph from atoms in the same metabolite can vary significantly. For instance, consider the distribution of distances from some of the carbon atoms in acetyl-CoA shown in Figure 11. As expected, distances from acetyl carbons (49 and 50) are very low compared to other atoms, particularly to carbons 3 and 7, as acetyl participates in a large variety of metabolic functions.

### Reconstruction of Trichoderma reesei amino acid biosynthesis pathways

In [36], amino acid pathways in the filamentous fungus *Trichoderma reesei *were reconstructed with ReTrace and subsequent manual curation. *T. reesei *is a recently sequenced organism [46]. Currently, there is no good-quality curated metabolic network for it. In [36], reconstruction of amino acid synthesis pathways was required, however. To this end, we performed a series of pathway queries to amino acids from their respective precursors. Pathways found were then manually curated.

First, the edges of atom graph were weighted to correspond sequence-level evidence on existence of each reaction derived from Blast [47] alignments. Specifically, we assigned scores to each reaction in KEGG database so that the scores reflect the likelihood that the reaction is catalyzed by some enzyme in the metabolic network. To accomplish this, we performed a pairwise Blast to query the genome sequence *S *of *T. reesei *against the UniProt [48] database *D*, which contained the known enzymatic sequences and their respective functions. Then, we assigned scores to reactions with the formula,

where *E*_*D*(*r*) _is the set of sequences in database *D *which have been annotated to have function (reaction) *r *and *B*(*s, d*) is the Blast score for aligning sequences *s *and *d*. In other words, the score of reaction *r *corresponded to the best Blast hit to the sequence of an enzyme known to catalyze *r*. The higher the Blast best-hit score was for some reaction, the lower weights the corresponding edges received in the atom graph accordingly to the formula described in Implementation. Thus, shortest paths in a graph weighted in this fashion tended to favor plausible reactions. In addition to the study described in [36], a similar scoring scheme has been employed in a recent method for reconstructing metabolic networks with mixed-integer linear optimization [9].

In [36], ReTrace was able to find a plausible biosynthesis pathway supported by sequence similarity evidence for a majority of amino acids. We observed that reaction score weighting in particular proved important in increasing the amount of pathways that existed in *T. reesei *according to manual curation in contrast to the unweighted case.

### Performance testing

We studied the performance of ReTrace and effect of different parameters by querying the atom graph described previously.

First, we evaluated the effect of increasing the number of paths computed at each search level to the total running time and number of pathways found. A total of 13 molecules with different number of carbon atoms were selected for the experiment. Only carbon atoms were utilized in searches and maximum search depth was not constrained. Pathways were computed from each molecule to every other molecule in the query set. Any pathway found with *Z*_*O *_> 0 was accepted. Queries were computed in a cluster of 25 computers running Intel Pentium 4 2.80 GHz dual-core CPUs. Because the current implementation was not parallelized, each query was run on a single core. Full result data is presented in the Additional file 2. Two experiments were performed. First, the number of paths at computed at each search level received values 1, 2, 3 and 4. In the second experiment, the number of paths at first and second search level was varied from 1 to 40 while computing only one path at third and subsequent search levels. Specifically, the option -*k *was given values (1, 1, 1), (2, 2, 1),..., (40, 40, 1).

**Figure 12:**
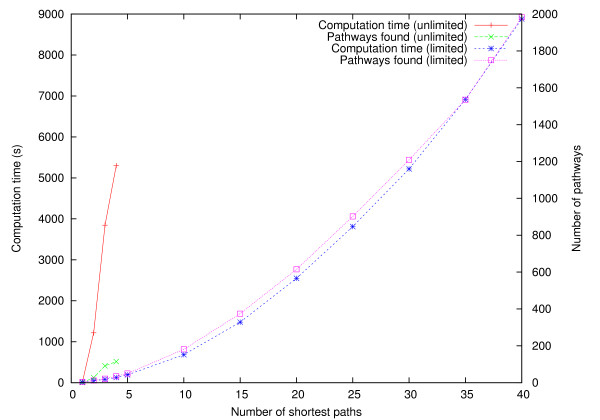
**ReTrace running time and number of pathways found**. Total running time and the number of pathways found in pairwise pathway queries between 13 metabolites. X-axis shows the number of shortest paths searched at each search level. Each point represents averages over 240 pairwise pathway queries.

Figure 12 shows the total running time and the number of pathways found with respect to the number of shortest paths *k *computed in the first and second experiment, labeled *unlimited *and *limited*, respectively. In the first experiment, the computation time grows rapidly with *k*, reaching over 5000 seconds on the average with *k *= 4. In the second experiment, computation takes less time and yields fewer result pathways than in the first experiment when *k *is increased because only one path is computed after the second search level. However, the number of result pathways grows faster than in the first experiment. This is due to a larger fraction of resulting pathways being duplicates in the first experiment. Hence, by limiting the pathway computations in deeper levels we are able to explore more thoroughly the first levels and obtain more unique solutions than when *k *remains constant at every search level.

**Figure 13:**
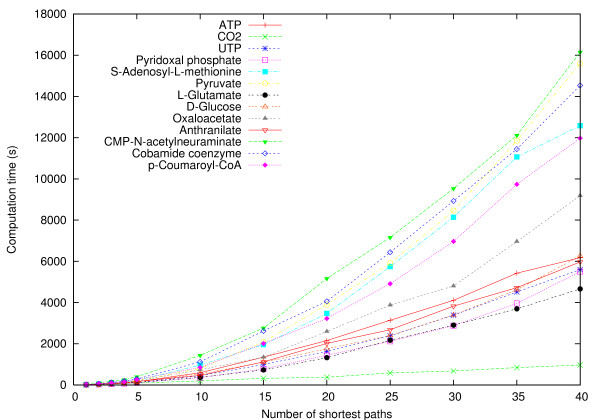
**Metabolite-specific computation time**. Total computation time shown separately for each target metabolite. X-axis shows the number of shortest paths searched at the first and second search level. Each point represents queries from 12 metabolites to the target metabolite.

ReTrace computation time varied significantly with respect to the complexity of the target metabolite and the number of pathways found for each query. As shown in Figure 13, computation of pathways to CMP-N-acetylneuraminate (20 carbons), pyruvate (3 carbons) and cobamide coenzyme (72 carbons) took the longest time.

**Figure 14:**
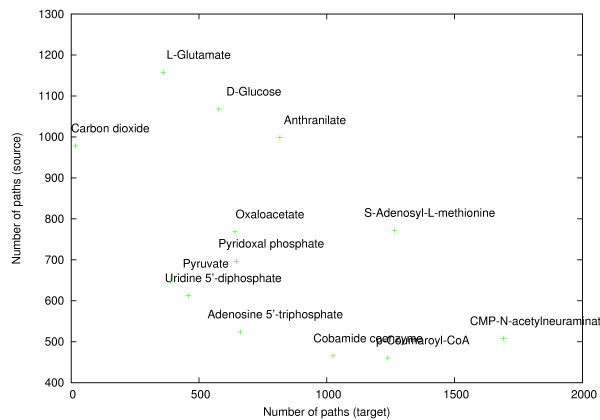
**Number of pathways found**. Number of pathways found on the average for queries where each metabolite in turn was considered as the source (Y-axis) and target (X-axis). Each point corresponds to averages over 12 results.

Figure 14 gives the average number of pathways found from and to each metabolite in the second experiment. The highest number of pathways were found when considering glucose, L-glutamate, CO_2 _or anthranilate as the source metabolite in the query. In contrast, the least number of pathways were found for complex metabolites such as cobamide coenzyme, p-coumaroyl-CoA, CMP-N-acetylneuraminate and ATP. Interestingly, a relatively low number of 564.41 pathways on the average were found for the central carbon metabolite pyruvate. When considering each metabolite as the pathway target, CO_2 _received the smallest amount of pathways. This is due to CO_2 _having only one carbon and being utilized in a large number of pathways. Hence, in most cases, only a simple pathway was necessary to transfer the sole carbon from each source. On the other hand, significantly higher amount of pathways were found for complex metabolites such as CMP-N-acetylneuraminate, S-adenosyl-L-methionine and p-couramoyl-CoA. Particularly, there was a notable difference in the number of pathways found for ATP and S-adenosyl-L-methionine. Although the two resemble each other structurally, only about half as many pathways were found for ATP compared to S-adenosyl-L-methionine.

**Figure 15:**
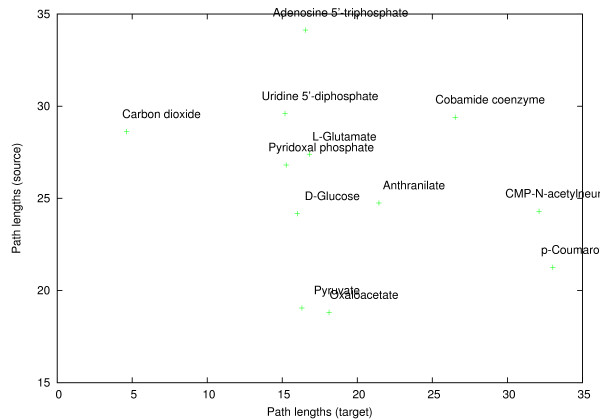
**Pathway sizes**. The average result pathway sizes for queries where each metabolite in turn was considered as the source (Y-axis) and target (X-axis). Each point corresponds to averages over 12 results.

Figure 15 shows sizes of the pathways found when considering each metabolite as source and target. Pathways from pyruvate and oxaloacetate were the smallest on the average. On the other hand, pathways from ATP were the largest. Comparatively to the data shown in Figure 14, pathways to CO_2 _were the smallest, while pathways to complex molecules p-coumaroyl-CoA, CMP-N-acetylneuraminate and cobamine coenzyme were the largest.

### Biosynthesis pathways from glucose to 5'-inosine monophosphate

Lastly, we computed pathways from glucose to 5'-inosine monophosphate (IMP) to validate usefulness of the method in generation of alternative biochemically realistic pathways. On a general IMP biosynthesis pathway as described in [49], IMP receives its carbons from ribose-5-phosphate, glycine, 10-formyl-THF, CO_2 _and aspartate. It is thus interesting to find out whether ReTrace is able to discover alternative, complete pathways to IMP from a single carbon atom source. In such scenario, the pathways found need to contain branches able to produce the different precursors to IMP.

We searched for pathways from glucose to IMP with ReTrace. Maximum search depth was set to 6 and the number of shortest paths computed at each step to 100 and 25 for search depths 1 and 2 and to 1 for depths 3,..., 6. Atom graph edges were given uniform weights. To assess the complexity of this pathway query, we stored also partial pathways in addition to those pathways with *Z*_*O *_= 1 and pathways which could not be extended any further because maximum search depth was encountered. In summary, a total of 4738 pathways were found in 3.5 hours on a Intel Xeon X5355 2.66 GHz CPU. Additional file 2 contains ReTrace html output from the query.

**Figure 16:**
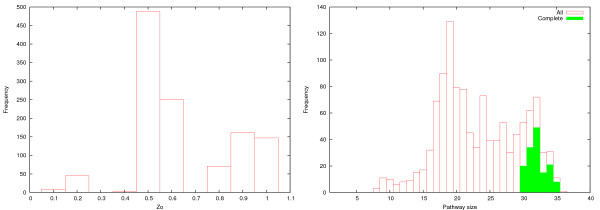
**Z_*O *_score distribution in pathways found**. Left: Distribution of *Z*_*O *_score in pathways found for query glucose → IMP. Right: Distribution of pathway sizes. Green bars show the distribution of complete (*Z*_*O *_= 1) pathways.

Figure 16 shows distributions of *Z*_*O *_scores and sizes of the 1173 pathways found. A total of 147 pathways received *Z*_*O *_= 1 score and were thus complete. Moreover, sizes of complete pathways ranged from 30 to 35 reactions (mean 32.0, standard deviation 1.40), while the average pathway size among all the pathways found was only 22.0 (standard deviation 6.09).

**Figure 17:**
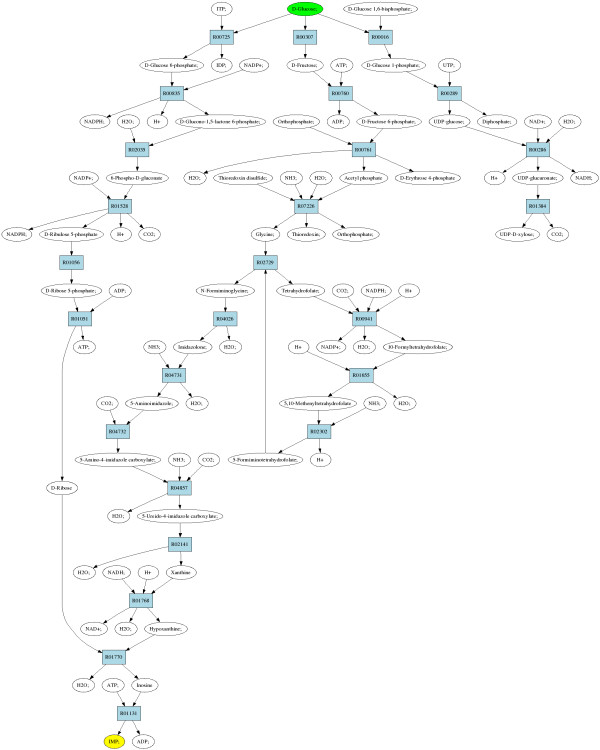
**Representative result pathway for query glucose → IMP**. A representative result pathway for the query glucose → IMP which utilizes reactions commonly used in other result pathways. Glucose and IMP are color-coded green and yellow, respectively.

Result pathways utilized enzymes with 166 distinct EC numbers. Table 4 shows the 28 enzymes which occurred in more than half of the pathways. For instance, three enzymes occurred in more than three fourths of the pathways, namely hexokinase, xanthine oxidase and xanthine dehydrogenase. Figure 17 shows a diagram of a complete result pathway, which utilizes enzymes that were included in other result pathways as well. Specifically, the pathway shown utilized enzymes which were commonly present in other result pathways as well and was thus picked as a representative pathway to demonstrate here.

**Table 4:** Frequently occuring enzymes in query glucose → IMP

Frequent enzymes in query glucose IMP		
**EC**	**Frequency**	**Name**

2.7.1.1	0.827792	hexokinase

1.17.3.2	0.761296	xanthine oxidase

1.17.1.4	0.761296	xanthine dehydrogenase

2.7.1.61	0.759591	acyl-phosphate--hexose phosphotransferase

3.1.3.10	0.665814	glucose-1-phosphatase

2.7.1.62	0.665814	phosphoramidate--hexose phosphotransferase

2.7.1.42	0.665814	riboflavin phosphotransferase

2.7.1.41	0.665814	glucose-1-phosphate phosphodismutase

4.1.1.35	0.663257	UDP-glucuronate decarboxylase

1.1.1.-	0.663257	limonene-1,2-diol dehydrogenase

1.1.1.22	0.663257	UDP-glucose 6-dehydrogenase

5.3.1.9	0.651321	glucose-6-phosphate isomerase

3.6.1.9	0.607843	nucleotide diphosphatase

3.6.1.8	0.607843	ATP diphosphatase

3.6.1.45	0.607843	UDP-sugar diphosphatase

2.7.7.9	0.607843	UTP--glucose-1-phosphate uridylyltransferase

2.7.7.12	0.607843	UDP-glucose--hexose-1-phosphate uridylyltransferase

3.1.3.9	0.586530	glucose-6-phosphatase

3.1.3.58	0.586530	sugar-terminal-phosphatase

2.7.1.63	0.586530	polyphosphate--glucose phosphotransferase

2.7.1.2	0.586530	glucokinase

2.7.1.147	0.586530	ADP-specific glucokinase

2.7.1.142	0.586530	glycerol-3-phosphate--glucose phosphotransferase

5.3.1.5	0.567775	xylose isomerase

2.7.1.4	0.539642	fructokinase

3.5.3.-	0.519182	N-succinylarginine dihydrolase

3.5.2.-	0.519182	enamidase

2.4.2.7	0.508951	adenine phosphoribosyltransferase

Enzymes which occurred in more than half of the pathways found for the query glucose → IMP.

The representative pathway corresponds to a prokaryotic pathway for synthesis of IMP described in [50]. The pathway consists of three "main" branches. First, the branch shown leftmost in Figure 17 produces D-ribose for inosine ribohydrolase, which combines it with hypoxanthine to produce IMP. The second branch first converts glucose to glycine and then further to hypoxanthine. Lastly, the third branch, shown rightmost in the figure, starts from glucose and ends in CO_2_. It should be noted, that the third branch is required to achieve a complete pathway: IMP receives carbons from carbon dioxide and ReTrace explores also branches that produce carbon dioxide from glucose. If such behavior is not required, it is possible to study further only results with *Z*_*O *_< 1.

In addition to the representative pathway, where IMP is synthesized via inosine, ReTrace found complete pathways where IMP is produced from AMP. However, no complete pathway was found which would produce IMP through 1-(5'-Phosphoribosyl)-5-formamido-4-imidazolecarboxamide (FAICAR), although such pathways were among the results with scores *Z*_*O *_< 1. The IMP biosynthesis pathway as described in [49] utilizes FAICAR as an intermediate, in particular. However, most pathways found by ReTrace take the shortcut ribose-5-phosphate → 5-phospho-alpha-d-ribose 1-diphosphate → AICAR → FAICAR → IMP instead of the longer route via GAR.

In summary, a total of 1134, 400 and 206 result pathways utilized inosine, AMP and FAICAR, respectively, as the immediate precursor to IMP.

## Conclusion

Numerous approaches have been developed for pathway analysis in metabolic networks. The two prominent frameworks are constraint-based modelling and graph-theoretic approaches, both having certain advantages over the other. Constraint-based methods have been reported to find biochemically more realistic pathways [33] but are difficult to apply to large-scale models. On the other hand, graph-theoretic path finding methods are applicable to very large networks, but are prone to return a large number of false positive, or irrelevant, pathways. In addition, most graph-theoretic methods do not support branching pathways. In both frameworks, one has to deal with the problem of correctly assigning the list of side metabolites, which is both non-trivial and context-dependent.

The method introduced in this paper, ReTrace, avoids problems with scalability while being able to find biochemically realistic, branching pathways. In contrast to most constraint-based methods, ReTrace is applicable to very large instances, involving genome-scale or larger metabolic networks. In addition, no explicit side metabolite list is required.

Similarly to ARM path finding [14], ReTrace operates on an atom-level representation of the metabolic network. We improve the ARM method by adding a support for branching pathways. Moreover, our method is a generalization of the ARM method as we can simulate ARM by setting ReTrace to find unbranched pathways only. In contrast to MetaRoute [18] and Metabolic Pathfinding [19], ReTrace finds paths in the atom-level metabolic network, instead of using the indirect method of encoding metabolite similarity into graph weights. This allows ReTrace to find pathways with net atom flow from sources to targets.

It should be noted that the method presented here also generalizes our previous work [17], where we studied branching pathways in metabolic network level instead of atom graphs. If we assign the set of target atoms to contain all atoms of a metabolite instead of just atoms traced back from current targets, we effectively arrive at an algorithm similar to our previous feasible pathways algorithm. In contrast to our previous work, the algorithm would operate at atom graph level, however, arguably resulting in more plausible results. We leave pursuing this topic as future work.

In this paper, we have demonstrated that ReTrace is able to discover biochemically feasible alternative pathways for complex metabolites in genome-scale networks. Further, our method has been applied to a biological problem of validation of metabolic pathways in an organism lacking good-quality metabolic reconstruction, namely *Trichoderma reesei*. As demonstrated in [36], the method lends itself naturally to metabolic reconstruction when we utilize reaction scores from genomic evidence. In general, reaction scores provides a mechanism to easily incorporate measurement data to guide the pathway search supported by experimental data. This broadens the applicability of ReTrace to a wide range of tasks. For instance, we could encode gene expression data in scores to find pathways active in different conditions.

We also studied the properties of the atom graph constructed from KEGG data. The pairwise distances between atoms were found to be significantly longer than in a previous study [45]. In particular, we identified a large number of graph components, between which no exchange of atoms is possible. This observation warrants a further study to find out whether the disconnectivity stems from errors in KEGG database, a biological phenomena, or both.

The availability and quality of atom mappings is of great importance to the method. Currently, methods for obtaining high-quality atom mappings are actively being investigated by many groups, including ours. Fortunately for applications demonstrated in this paper, we are mostly able to ignore the problem of deciding between alternative mappings stemming from apparently isomorphic fragments.

## Authors' contributions

EP designed, analyzed and implemented the algorithm, performed the experiments and wrote the manuscript. PJ and JR contributed to the development of the method and writing the manuscript. PJ and EP analyzed the results of the glucose-IMP experiment. All authors have read and approved the final version of the manuscript.

## Supplementary Material

Additional file 1 **ReTrace user guide and implementation notes**. ReTrace implementation details and user guide. A self-contained web site: unpack archive and open index.html in a web browser. Click here for file

Additional file 2 **ReTrace results from experiments**. Summary data and html output from ReTrace runs performed for the queries discussed in the section Results. A self-contained web site: unpack archive and open index.html in a web browser. Click here for file
