# Supplementary material for: Inferring branching pathways in genome-scale metabolic networks
Source: BMC Syst Biol. 2009 Oct 29;3:103. doi: 10.1186/1752-0509-3-103 (PMC2791103; doi:10.1186/1752-0509-3-103)
Supplement: Additional file 1 — ReTrace user guide and implementation notes. ReTrace implementation details and user guide. A self-contained web site: unpack archive and open index.html in a web browser. [file 1752-0509-3-103-S1.zip › retrace-AF1/index.html]

ReTrace user guide and implementation notes


This document is the Additional file 1 of the manuscript

```
Esa Pitk�nen, Paula Jouhten and Juho Rousu:
Inferring branching pathways in genome-scale metabolic networks. 
Submitted, 2009.
```

You can find Additional file 2 here.

ReTrace
is a computational method and software for inferring branching
pathways in genome-scale networks. Below you can find the
User guide and
Implementation notes
of the software.

- User guide
  - Quick guide- Command line options
    - Description of output files
    - Installation
- Implementation notes

# ReTrace user guide

## Quick guide

Provided you have already installed
ReTrace successfully and have a local copy of
KEGG LIGAND database in
directory *kegg*,
you are able to compute branching pathways from
metabolite X to metabolite Y by invoking

```
python retrace.py -d kegg -o results -s X -t Y
```

The results will be written to the directory *results*.
For instance, to compute pathways between glucose (KEGG LIGAND
identifier C00031)
and acetyl-CoA
(C00024),
invoke the command

```
python retrace.py -d kegg -o results -s C00031 -t C00024
```

The main result file, written into
output/pathways-C00031-to-C00024.html, should resemble the
example file you can find here.

## Command line options

| Option | Description | Default value |
| --- | --- | --- |
| -d | KEGG database directory | *Required* |
| -o | Output directory | *Required* |
| -s | Source metabolites | *Required* |
| -t | Target metabolite | *Required* |
| -a | Traced atom types |  |
| -c | Reaction score file | - |
| -e | Atom graph edge weights: (u)niform, (s)cores, (a)toms | Uniform |
| -g | Greedy finish: set k=1 for search levels 2 and beyond | No |
| -i | Report incomplete pathways | No |
| -l | Maximum pathway size | Unbounded |
| -k | Number of shortest paths computed in each step | 50,1 |
| -m | Maximum search depth | 3 |
| -p | Prune atom graph | No pruning |
| -r | Reaction direction constraints file | - |
| -w | Minimum ZO score requirement | 0 |

Command-line options of ReTrace are summarized in above table.
The user is required to give the directory (-d) where the local copy of
KEGG LIGAND database has been installed, the directory where ReTrace
output (-o) is written to, and the source (-s) and target (-t)
metabolites.

### Specifying multiple sources and/or source atom subsets

More than one source metabolites, given as a comma-separated list of
KEGG compound identifiers, are admitted (e.g., "C00024,C00026").
If a particular subset of source atoms are of interest, such often is
the case with AcCoA, for example, the source atoms can be limited to this subset by
giving the atoms as a list after each source atom. The list needs to
be separated by a dash (-) from the source metabolite, and atoms in
the list by a slash (/). For instance, running ReTrace with the
command

```
python retrace.py -d kegg -o result -s C00024-49/50 -t C00047
```

would search for paths from AcCoA (C00024) acetyl group carbons (49 and 50
in the atom numbering of KEGG March 2009 version) to Lysine (C00047).
This search would adopt
the default values for the number of shortest paths computed in each
step and the maximum search depth. Particularly only carbon atoms would
by traced in search.

### Controlling the exhaustiveness of search

Increasing the default number of shortest paths computed with -k
results in more pathways being found and a higher computational cost
at every search level. For a more
fine-tuned control, a comma-separated list of integers can be
specified with -k to set k individually for each search level.
For instance, a query with -k 50,10,1 would search for 50 shortest
paths at the first level, then 10 at the second and 1 at the third and
subsequent levels. Option -g provides a quick way of specifying
a search where -k option determines the number of paths at the first
level but at second and following levels only 1 path is computer per
level. This is particularly useful when the focus is on finding
different linear connections from sources to target and possible
branches can be resolved with any (single) path.

Setting maximum search depth with option -m governs how many branches
at maximum appear in result pathways.
It should be noted, that
with the option -m 1 the method closely corresponds the operation of
the ARM method in the sense that it
searches for k shortest, unbranching pathways in an atom graph.

### Taking advantage of external data

As described in the manuscript,
ReTrace can take advantage of scores computed for any subset of KEGG
reactions. A score file, specified with the -c option, has to contain
one reaction-score pair per line, separated by a tab character. Any
reaction with no score specified is considered to have a zero score.
The -c option should be used in conjunction with the -e option to
reweight the atom graph edges by reaction scores. By default edges are
assigned uniform weights.

Another weighting option is to give each edge
(va, vb) induced by a reaction r
the weight 1/ *alpha*, where *alpha* is the number of edges in total
connecting the metabolites of atoms a and b in reaction r.
Therefore, this weighting scheme favors
pathways traversing reactions which involve a large number of
atoms.

### Changing traced atom types

Currently, KEGG data contains mappings for carbon, nitrogen, oxygen
and phosphorus atoms. By default, however, ReTrace utilizes only
carbon atoms in search. This behavior can be changed with the option
-a by giving a comma-separate list of element symbols, for example
C,N,P. In general, accurate atom mappings for oxygens are hard to
compute because of the typical high degree of symmetry involved.
However, when studying nitrogen metabolism, for instance, it is
necessary to include also nitrogens in search with this option.

### Reducing atom graph size by pruning

In experiments reported in this study, we found it unnecessary to
prune the atom graph induced by KEGG reactions. However, for some
purposes, it may be useful to prune the graph to reduce the
computation time. To accomplish this, ReTrace supports the -p option
which can be supplied an integer n governing the degree of pruning.
Specifically, ReTrace prunes the atom graph by considering
the total distance of reactions from both sources and target and
leaving the n reactions with smallest total distance into the graph
and removing the others. Pruning respects the reweighting scheme
chosen with the option -e.

### Constraining reaction directions

If available, ReTrace is able to incorporate constraints to reaction
directions in search. This is done via the option -r by supplying
the file containing KEGG reaction identifiers and direction constraint
<, > in each line. For instance, the following three lines
would constrain the reactions R00199, R00200 and R00206 involving
PEP -> Pyr so that no edge Pyr -> PEP appears in results
because of these reactions. Note that a KEGG reaction file needs to be
examined to determine the correct reaction direction - in this case
the three reactions have been specified in KEGG in Pyr <- PEP
direction, hence the < constraint.

```
R00199 <
R00200 <
R00206 <
```

## Description of output files

ReTrace generates html results file for the query named according to source
and target metabolite identifiers.
For instance, for a query from Acetyl-CoA to Lysine, a main html
result file named pathways-C00024-to-C00047.html would be generated in
the directory specified with the option -o. In addition, a html file
is generated for each pathway found. These are accessible from the
main html result file, which reports for each summary information
including composite mapping, ZO, average reaction score, number of
RPAIRs and reactions utilized and number of reactions appearing on the
pathways having zero or low reaction score. Currently, low score threshold
can be only specified by changing a constant in the source file
htmlexport.py.

The above figure shows an excerpt from a pathway result file for a
query from E4P and PEP to Phe. In addition to the molecule structures
with transferred atoms indicated, the pathway result file contains
a table detailing each RPAIR and reaction associated with the pathway.
Finally, if Graphviz has been available during ReTrace execution as
discussed above, a pathway diagram is shown (example figure).
In diagram, source and
target metabolites are colored green and yellow, respectively. If
reaction scores have been provided, reactions with zero score and low
score are colored red and blue, respectively. Reactions with scores
above threshold are colored green.

For convience, identifiers in the result tables and pathway diagrams
are hyperlinked to appropriate KEGG web site pages for easier
interpretation of results.

## Installation

### Prerequisites

You need to have the following software installed and available in
path so they can be invoked from command line.

- Python 2.5 (later versions might work too)
- Graphviz dot tool (only to draw
  pathway figures)

### Installation

- Download
  the latest ReTrace version (retrace-X.YY.zip or retrace-X.YY.tar.gz).
- Unzip the archive into a directory (folder). A subdirectory will be
  created named retrace-X.YY where X.YY specifies version number.
- Test your installation by invoking the command

  ```
  python retrace.py
  ```

  You should see a basic summary of command line options.
- Now you can try the example in Quick guide.

# Implementation notes

- The Python program retrace.py implements ReTrace as an iterative
  algorithm, instead as recursion as described in the manuscript.
- The program is divided into following four main-level scripts and
  four packages:

- retrace.py - main program, algorithm implementation
- tracerutil.py - atom graph construction
- htmlexport.py - result file generation
- kspyen.py - implementation of Yen's k shortest simple paths algorithm
- graph/ - graph utility classes
- eppstein/ - David
  Eppstein's implementation of Dijkstra's
  algorithm and priority queue
- metabolism/ - utility classes for metabolic networks, KEGG LIGAND parser
- ds/ - combinatorial functions
