# Supplementary figures and images for: Inferring branching pathways in genome-scale metabolic networks
Source: BMC Syst Biol. 2009 Oct 29;3:103. doi: 10.1186/1752-0509-3-103 (PMC2791103; doi:10.1186/1752-0509-3-103)

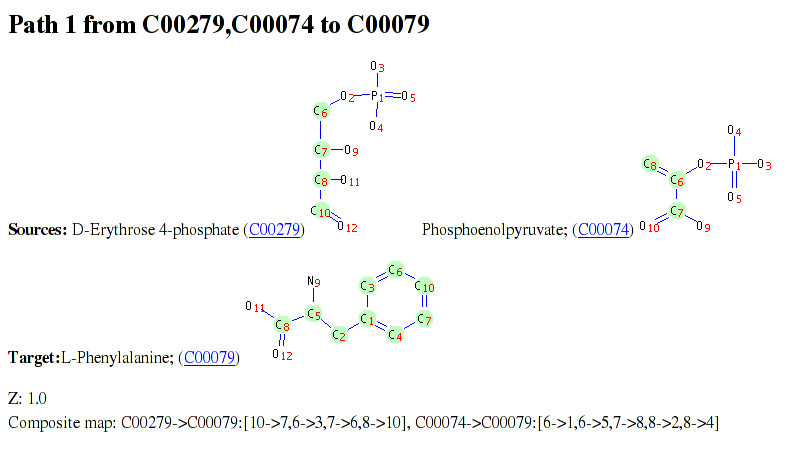

Supplement: Additional file 1 — ReTrace user guide and implementation notes. ReTrace implementation details and user guide. A self-contained web site: unpack archive and open index.html in a web browser. [file 1752-0509-3-103-S1.zip › retrace-AF1/gui1.png]

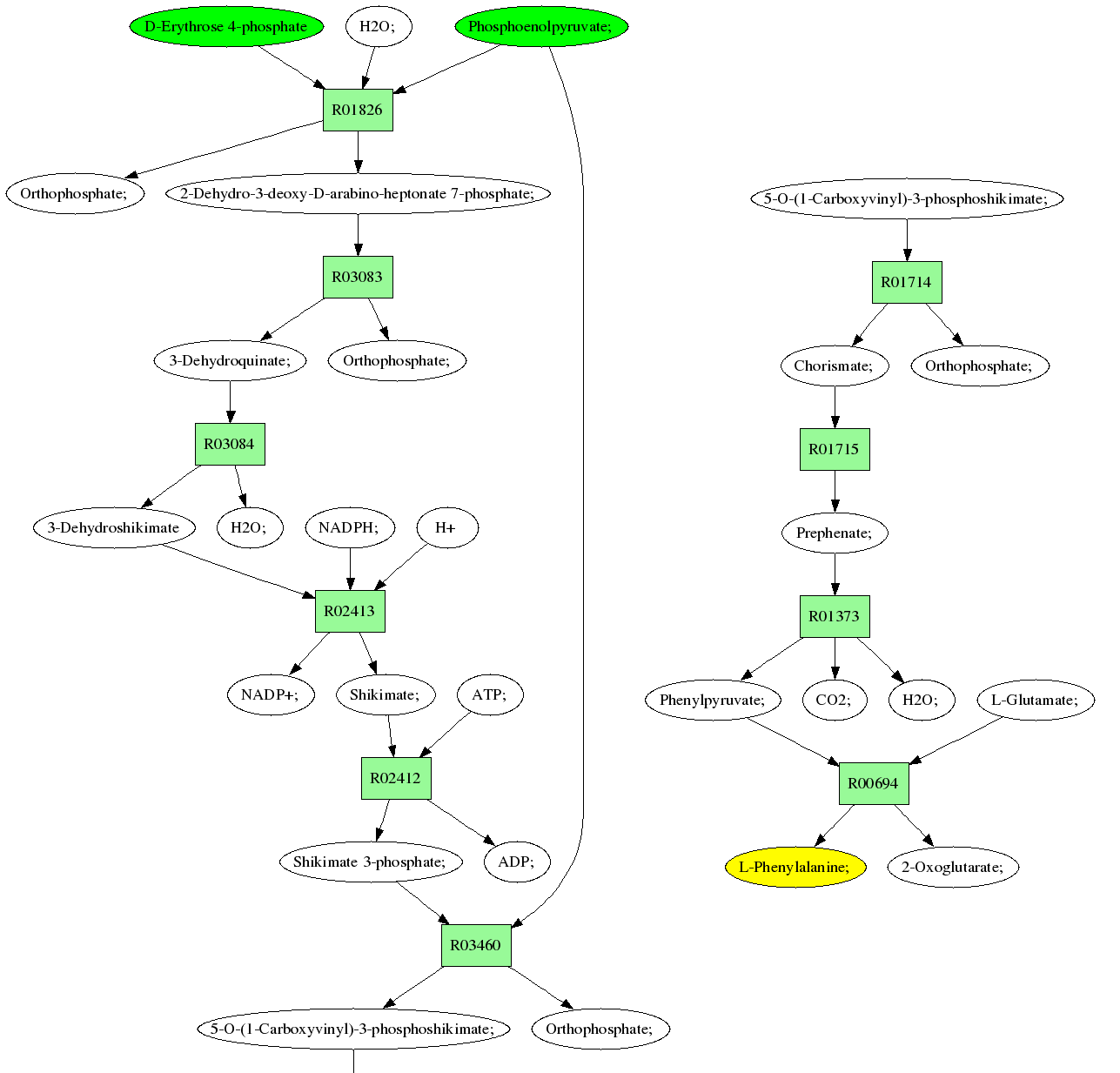

Supplement: Additional file 1 — ReTrace user guide and implementation notes. ReTrace implementation details and user guide. A self-contained web site: unpack archive and open index.html in a web browser. [file 1752-0509-3-103-S1.zip › retrace-AF1/phepath.png]
