# Supplementary material for: Inferring branching pathways in genome-scale metabolic networks
Source: BMC Syst Biol. 2009 Oct 29;3:103. doi: 10.1186/1752-0509-3-103 (PMC2791103; doi:10.1186/1752-0509-3-103)
Supplement: Additional file 2 — ReTrace results from experiments. Summary data and html output from ReTrace runs performed for the queries discussed in the section Results. A self-contained web site: unpack archive and open index.html in a web browser. [file 1752-0509-3-103-S2.zip › retrace-AF2/pathways-C00117-to-C00130.html]

Pathways from C00117 to C00130


**Pathways from C00117 to C00130**

**Sources:** D-Ribose 5-phosphate; (C00117)

**Target:**IMP; (C00130)

|  | Composite mapping | Z | Average score | Rpairs | Reactions | Zero scores | Scores under threshold |
| --- | --- | --- | --- | --- | --- | --- | --- |
| Path 1 | C00117->C00130:[10->8,12->3,6->17,7->10,7->11,7->12,7->4,7->5,8->13] | 0.90 | 0.0 | 24 | 31 | 0 | 24 |
| Path 2 | C00117->C00130:[10->8,12->3,6->17,7->10,7->11,7->12,7->4,7->5,8->13] | 0.90 | 0.0 | 24 | 33 | 0 | 24 |
| Path 3 | C00117->C00130:[10->8,12->3,6->17,7->10,7->11,7->12,7->4,7->5,8->13] | 0.90 | 0.0 | 24 | 33 | 0 | 24 |
| Path 4 | C00117->C00130:[10->8,12->3,6->17,7->10,7->11,7->12,7->4,7->5,8->13] | 0.90 | 0.0 | 22 | 32 | 0 | 22 |
| Path 5 | C00117->C00130:[10->8,12->3,6->17,7->10,7->11,7->12,7->4,7->5,8->13] | 0.90 | 0.0 | 20 | 26 | 0 | 20 |
| Path 6 | C00117->C00130:[10->8,12->3,6->17,7->10,7->11,7->12,7->4,7->5,8->13] | 0.90 | 0.0 | 21 | 27 | 0 | 21 |
| Path 7 | C00117->C00130:[10->8,12->3,6->17,7->10,7->11,7->12,7->4,7->5,8->13] | 0.90 | 0.0 | 23 | 30 | 0 | 23 |
| Path 8 | C00117->C00130:[10->8,12->3,6->17,7->10,7->11,7->12,7->4,7->5,8->13] | 0.90 | 0.0 | 21 | 29 | 0 | 21 |
| Path 9 | C00117->C00130:[10->8,12->3,6->17,7->10,7->11,7->12,7->4,7->5,8->13] | 0.90 | 0.0 | 20 | 28 | 0 | 20 |
| Path 10 | C00117->C00130:[10->8,12->3,6->17,7->10,7->11,7->12,7->4,7->5,8->13] | 0.90 | 0.0 | 24 | 31 | 0 | 24 |
| Path 11 | C00117->C00130:[10->8,12->3,6->17,7->10,7->11,7->12,7->4,7->5,8->13] | 0.90 | 0.0 | 22 | 30 | 0 | 22 |
| Path 12 | C00117->C00130:[10->8,12->3,6->17,7->10,7->11,7->12,7->4,7->5,8->13] | 0.90 | 0.0 | 23 | 30 | 0 | 23 |
| Path 13 | C00117->C00130:[10->8,12->3,6->17,7->10,7->11,7->12,7->4,7->5,8->13] | 0.90 | 0.0 | 20 | 27 | 0 | 20 |
| Path 14 | C00117->C00130:[10->8,12->3,6->17,7->10,7->11,7->12,7->4,7->5,8->13] | 0.90 | 0.0 | 22 | 32 | 0 | 22 |
| Path 15 | C00117->C00130:[10->8,12->3,6->17,7->10,7->11,7->12,7->4,7->5,8->13] | 0.90 | 0.0 | 23 | 30 | 0 | 23 |
| Path 16 | C00117->C00130:[10->8,12->3,6->17,7->10,7->11,7->12,7->4,7->5,8->13] | 0.90 | 0.0 | 20 | 26 | 0 | 20 |
| Path 17 | C00117->C00130:[10->8,12->3,6->17,7->10,7->11,7->12,7->4,7->5,8->13] | 0.90 | 0.0 | 21 | 29 | 0 | 21 |
| Path 18 | C00117->C00130:[10->8,12->3,6->17,7->10,7->11,7->12,7->4,7->5,8->13] | 0.90 | 0.0 | 23 | 31 | 0 | 23 |
| Path 19 | C00117->C00130:[10->8,12->3,6->17,7->10,7->11,7->12,7->4,7->5,8->13] | 0.90 | 0.0 | 20 | 27 | 0 | 20 |
| Path 20 | C00117->C00130:[10->8,12->3,6->17,7->10,7->11,7->12,7->4,7->5,8->13] | 0.90 | 0.0 | 23 | 31 | 0 | 23 |
| Path 21 | C00117->C00130:[10->8,12->3,6->17,7->10,7->11,7->12,7->4,7->5,8->13] | 0.90 | 0.0 | 21 | 27 | 0 | 21 |
| Path 22 | C00117->C00130:[10->8,12->3,6->17,7->10,7->11,7->12,7->4,7->5,8->13] | 0.90 | 0.0 | 20 | 26 | 0 | 20 |
| Path 23 | C00117->C00130:[10->8,12->3,6->10,6->17,7->12,8->11,8->13,8->4] | 0.80 | 0.0 | 31 | 47 | 0 | 31 |
| Path 24 | C00117->C00130:[10->8,12->3,6->17,7->10,7->11,7->12,8->13] | 0.70 | 0.0 | 16 | 23 | 0 | 16 |
| Path 25 | C00117->C00130:[10->8,12->3,6->10,6->17,7->11,7->12,7->4,8->13] | 0.80 | 0.0 | 31 | 37 | 0 | 31 |
| Path 26 | C00117->C00130:[10->8,12->3,6->17,7->12,8->11,8->13] | 0.60 | 0.0 | 13 | 15 | 0 | 13 |
| Path 27 | C00117->C00130:[10->8,12->3,6->17,7->12,8->11,8->13] | 0.60 | 0.0 | 16 | 26 | 0 | 16 |
| Path 28 | C00117->C00130:[10->10,10->8,12->3,6->17,7->12,8->13] | 0.60 | 0.0 | 16 | 21 | 0 | 16 |
| Path 29 | C00117->C00130:[10->8,12->3,6->17,7->12,8->11,8->13] | 0.60 | 0.0 | 13 | 20 | 0 | 13 |
| Path 30 | C00117->C00130:[10->8,12->3,6->17,7->11,7->12,8->13] | 0.60 | 0.0 | 12 | 23 | 0 | 12 |
| Path 31 | C00117->C00130:[10->8,12->3,6->17,7->10,7->11,7->12,7->4,8->10,8->13] | 0.80 | 0.0 | 33 | 39 | 0 | 33 |
| Path 32 | C00117->C00130:[10->8,12->3,6->17,7->12,8->11,8->13] | 0.60 | 0.0 | 20 | 26 | 0 | 20 |
| Path 33 | C00117->C00130:[10->8,12->3,6->17,7->10,7->11,7->12,7->4,7->5,8->13] | 0.90 | 0.0 | 23 | 42 | 0 | 23 |
| Path 34 | C00117->C00130:[10->8,12->3,6->17,7->10,7->11,7->12,7->4,8->13] | 0.80 | 0.0 | 21 | 38 | 0 | 21 |
| Path 35 | C00117->C00130:[10->8,12->3,6->17,7->10,7->11,7->12,7->4,8->13] | 0.80 | 0.0 | 21 | 41 | 0 | 21 |
| Path 36 | C00117->C00130:[10->8,12->3,6->17,7->10,7->11,7->12,7->4,8->13] | 0.80 | 0.0 | 32 | 49 | 0 | 32 |
| Path 37 | C00117->C00130:[10->8,12->3,6->17,7->10,7->11,7->12,8->13] | 0.70 | 0.0 | 18 | 26 | 0 | 18 |
| Path 38 | C00117->C00130:[10->8,12->3,6->17,7->12,8->11,8->13] | 0.60 | 0.0 | 16 | 22 | 0 | 16 |
| Path 39 | C00117->C00130:[8->11] | 0.10 | 0.0 | 11 | 22 | 0 | 11 |
| Path 40 | C00117->C00130:[10->8,12->3,6->17,7->10,7->11,7->12,7->4,8->13] | 0.80 | 0.0 | 31 | 36 | 0 | 31 |
| Path 41 | C00117->C00130:[10->8,12->3,6->17,7->10,7->11,7->12,7->4,7->5,8->13] | 0.90 | 0.0 | 26 | 35 | 0 | 26 |
| Path 42 | C00117->C00130:[10->8,12->3,6->17,7->10,7->11,7->12,7->2,7->5,8->13] | 0.90 | 0.0 | 26 | 32 | 0 | 26 |
| Path 43 | C00117->C00130:[10->8,12->3,6->17,7->12,8->10,8->13] | 0.60 | 0.0 | 14 | 20 | 0 | 14 |
| Path 44 | C00117->C00130:[10->8,12->3,6->17,7->12,8->11,8->13] | 0.60 | 0.0 | 16 | 20 | 0 | 16 |
| Path 45 | C00117->C00130:[10->8,12->3,6->10,6->17,7->12,8->11,8->13,8->4] | 0.80 | 0.0 | 32 | 36 | 0 | 32 |
| Path 46 | C00117->C00130:[10->8,12->3,6->10,6->17,7->12,8->11,8->13,8->4] | 0.80 | 0.0 | 31 | 39 | 0 | 31 |
| Path 47 | C00117->C00130:[10->8,12->3,6->17,7->12,8->11,8->13] | 0.60 | 0.0 | 21 | 26 | 0 | 21 |
| Path 48 | C00117->C00130:[10->8,12->3,6->17,7->12,8->11,8->13] | 0.60 | 0.0 | 19 | 33 | 0 | 19 |
| Path 49 | C00117->C00130:[10->8,12->3,6->17,7->12,8->13] | 0.50 | 0.0 | 5 | 6 | 0 | 5 |
| Path 50 | C00117->C00130:[10->8,12->3,6->17,7->10,7->11,7->12,8->13] | 0.70 | 0.0 | 13 | 16 | 0 | 13 |
| Path 51 | C00117->C00130:[10->8,12->3,6->17,7->10,7->12,7->4,8->13] | 0.70 | 0.0 | 17 | 26 | 0 | 17 |
| Path 52 | C00117->C00130:[10->8,12->3,6->10,6->17,7->11,7->12,7->4,8->13] | 0.80 | 0.0 | 30 | 47 | 0 | 30 |
| Path 53 | C00117->C00130:[10->8,12->3,6->17,7->10,7->11,7->12,7->2,7->5,8->13] | 0.90 | 0.0 | 29 | 36 | 0 | 29 |
| Path 54 | C00117->C00130:[10->8,12->3,6->17,7->12,8->11,8->13] | 0.60 | 0.0 | 16 | 27 | 0 | 16 |
| Path 55 | C00117->C00130:[10->8,12->3,6->17,7->11,7->12,8->13] | 0.60 | 0.0 | 12 | 15 | 0 | 12 |
| Path 56 | C00117->C00130:[10->8,12->3,6->17,7->12,8->11,8->13] | 0.60 | 0.0 | 19 | 33 | 0 | 19 |
| Path 57 | C00117->C00130:[10->8,12->3,6->17,7->10,7->11,7->12,7->2,8->13] | 0.80 | 0.0 | 25 | 31 | 0 | 25 |
| Path 58 | C00117->C00130:[10->8,12->3,6->17,7->10,7->12,8->13] | 0.60 | 0.0 | 15 | 24 | 0 | 15 |
| Path 59 | C00117->C00130:[10->8,12->3,6->17,7->12,8->11,8->13] | 0.60 | 0.0 | 19 | 23 | 0 | 19 |
| Path 60 | C00117->C00130:[10->8,12->3,6->10,6->17,7->11,7->12,7->4,8->13] | 0.80 | 0.0 | 32 | 42 | 0 | 32 |
| Path 61 | C00117->C00130:[10->8,12->3,6->17,7->10,7->11,7->12,7->4,8->13] | 0.80 | 0.0 | 18 | 25 | 0 | 18 |
| Path 62 | C00117->C00130:[10->8,12->3,6->17,7->12,8->11,8->13] | 0.60 | 0.0 | 23 | 31 | 0 | 23 |
| Path 63 | C00117->C00130:[10->8,12->3,6->17,7->10,7->11,7->12,7->4,8->13] | 0.80 | 0.0 | 20 | 30 | 0 | 20 |
| Path 64 | C00117->C00130:[10->8,12->3,6->17,7->10,7->12,7->4,8->13] | 0.70 | 0.0 | 20 | 29 | 0 | 20 |
| Path 65 | C00117->C00130:[10->8,12->3,6->17,7->10,7->11,7->12,7->4,7->5,8->13] | 0.90 | 0.0 | 27 | 40 | 0 | 27 |
| Path 66 | C00117->C00130:[10->8,12->3,6->17,7->12,8->11,8->13] | 0.60 | 0.0 | 14 | 21 | 0 | 14 |
| Path 67 | C00117->C00130:[10->8,12->3,6->17,7->10,7->11,7->12,7->4,8->13] | 0.80 | 0.0 | 18 | 24 | 0 | 18 |
| Path 68 | C00117->C00130:[10->8,12->3,6->17,7->10,7->11,7->12,7->2,7->5,8->13] | 0.90 | 0.0 | 25 | 43 | 0 | 25 |
| Path 69 | C00117->C00130:[6->10,6->11,8->10,8->11] | 0.20 | 0.0 | 22 | 34 | 0 | 22 |
| Path 70 | C00117->C00130:[10->8,12->3,6->17,7->12,8->11,8->13] | 0.60 | 0.0 | 14 | 22 | 0 | 14 |
| Path 71 | C00117->C00130:[10->8,12->3,6->17,7->10,7->12,7->4,8->13] | 0.70 | 0.0 | 19 | 27 | 0 | 19 |
| Path 72 | C00117->C00130:[10->8,12->3,6->17,7->10,7->11,7->12,7->4,8->13] | 0.80 | 0.0 | 34 | 46 | 0 | 34 |
| Path 73 | C00117->C00130:[10->8,12->3,6->17,7->12,8->11,8->13] | 0.60 | 0.0 | 15 | 23 | 0 | 15 |
| Path 74 | C00117->C00130:[10->8,12->3,6->17,7->10,7->12,7->4,8->13] | 0.70 | 0.0 | 22 | 45 | 0 | 22 |
| Path 75 | C00117->C00130:[10->8,12->3,6->17,7->11,7->12,8->13] | 0.60 | 0.0 | 12 | 14 | 0 | 12 |
| Path 76 | C00117->C00130:[6->10,6->11] | 0.20 | 0.0 | 24 | 29 | 0 | 24 |
| Path 77 | C00117->C00130:[10->8,12->3,6->17,7->10,7->12,7->4,8->13] | 0.70 | 0.0 | 18 | 25 | 0 | 18 |
| Path 78 | C00117->C00130:[10->8,12->3,6->17,7->10,7->11,7->12,7->2,8->13] | 0.80 | 0.0 | 23 | 40 | 0 | 23 |
| Path 79 | C00117->C00130:[10->8,12->3,6->17,7->12,8->11,8->13] | 0.60 | 0.0 | 19 | 23 | 0 | 19 |
| Path 80 | C00117->C00130:[8->11] | 0.10 | 0.0 | 12 | 16 | 0 | 12 |
| Path 81 | C00117->C00130:[10->8,12->3,6->17,7->12,8->11,8->13] | 0.60 | 0.0 | 20 | 27 | 0 | 20 |
| Path 82 | C00117->C00130:[10->8,12->3,6->17,7->12,8->11,8->13] | 0.60 | 0.0 | 14 | 16 | 0 | 14 |
| Path 83 | C00117->C00130:[10->8,12->3,6->17,7->12,8->11,8->13] | 0.60 | 0.0 | 25 | 36 | 0 | 25 |
| Path 84 | C00117->C00130:[10->8,12->3,6->17,7->10,7->11,7->12,8->13] | 0.70 | 0.0 | 18 | 25 | 0 | 18 |
| Path 85 | C00117->C00130:[8->11] | 0.10 | 0.0 | 10 | 12 | 0 | 10 |
| Path 86 | C00117->C00130:[10->8,12->3,6->17,7->10,7->11,7->12,7->4,7->5,8->13] | 0.90 | 0.0 | 23 | 40 | 0 | 23 |
| Path 87 | C00117->C00130:[10->8,12->3,6->17,7->12,8->11,8->13] | 0.60 | 0.0 | 23 | 31 | 0 | 23 |
| Path 88 | C00117->C00130:[10->8,12->3,6->17,7->12,8->10,8->11,8->13] | 0.70 | 0.0 | 16 | 19 | 0 | 16 |
| Path 89 | C00117->C00130:[6->10,6->11,8->10,8->11] | 0.20 | 0.0 | 22 | 26 | 0 | 22 |
| Path 90 | C00117->C00130:[10->8,12->3,6->17,7->12,8->11,8->13] | 0.60 | 0.0 | 17 | 23 | 0 | 17 |
| Path 91 | C00117->C00130:[10->8,12->3,6->17,7->12,8->10,8->11,8->13] | 0.70 | 0.0 | 16 | 21 | 0 | 16 |
| Path 92 | C00117->C00130:[10->8,12->3,6->17,7->12,8->11,8->13] | 0.60 | 0.0 | 17 | 22 | 0 | 17 |
| Path 93 | C00117->C00130:[10->8,12->3,6->17,7->10,7->12,7->4,8->13] | 0.70 | 0.0 | 17 | 24 | 0 | 17 |
| Path 94 | C00117->C00130:[10->8,12->3,6->17,7->10,7->12,8->13] | 0.60 | 0.0 | 14 | 27 | 0 | 14 |
| Path 95 | C00117->C00130:[10->11,10->8,12->3,6->17,7->12,8->13] | 0.60 | 0.0 | 13 | 16 | 0 | 13 |
| Path 96 | C00117->C00130:[10->8,12->3,6->17,7->10,7->11,7->12,7->2,8->13] | 0.80 | 0.0 | 24 | 34 | 0 | 24 |
| Path 97 | C00117->C00130:[10->8,12->3,6->17,7->12,8->11,8->13] | 0.60 | 0.0 | 15 | 26 | 0 | 15 |
| Path 98 | C00117->C00130:[8->11] | 0.10 | 0.0 | 11 | 22 | 0 | 11 |
| Path 99 | C00117->C00130:[10->8,12->3,6->17,7->10,7->12,7->4,8->13] | 0.70 | 0.0 | 21 | 29 | 0 | 21 |
| Path 100 | C00117->C00130:[10->8,12->3,6->17,7->12,8->10,8->11,8->13] | 0.70 | 0.0 | 15 | 16 | 0 | 15 |
| Path 101 | C00117->C00130:[10->8,12->3,6->17,7->12,8->11,8->13] | 0.60 | 0.0 | 19 | 25 | 0 | 19 |
| Path 102 | C00117->C00130:[10->8,12->3,6->17,7->12,8->11,8->13] | 0.60 | 0.0 | 12 | 13 | 0 | 12 |
| Path 103 | C00117->C00130:[10->8,12->3,6->17,7->12,8->11,8->13] | 0.60 | 0.0 | 19 | 33 | 0 | 19 |
| Path 104 | C00117->C00130:[10->8,12->3,6->17,7->10,7->11,7->12,8->13] | 0.70 | 0.0 | 16 | 30 | 0 | 16 |
| Path 105 | C00117->C00130:[10->8,12->3,6->17,7->10,7->11,7->12,8->13] | 0.70 | 0.0 | 19 | 29 | 0 | 19 |
| Path 106 | C00117->C00130:[10->8,12->3,6->17,7->10,7->11,7->12,7->4,8->13] | 0.80 | 0.0 | 31 | 39 | 0 | 31 |
| Path 107 | C00117->C00130:[10->8,12->3,6->17,7->10,7->11,7->12,7->4,8->13] | 0.80 | 0.0 | 32 | 46 | 0 | 32 |
| Path 108 | C00117->C00130:[10->8,12->3,6->17,7->12,8->11,8->13] | 0.60 | 0.0 | 20 | 27 | 0 | 20 |
| Path 109 | C00117->C00130:[10->8,12->3,6->17,7->12,8->11,8->13] | 0.60 | 0.0 | 24 | 29 | 0 | 24 |
| Path 110 | C00117->C00130:[10->8,12->3,6->17,7->12,8->11,8->13] | 0.60 | 0.0 | 16 | 24 | 0 | 16 |
| Path 111 | C00117->C00130:[10->8,12->3,6->17,7->10,7->11,7->12,8->13] | 0.70 | 0.0 | 17 | 20 | 0 | 17 |
| Path 112 | C00117->C00130:[10->8,12->3,6->17,7->12,8->13] | 0.50 | 0.0 | 5 | 5 | 0 | 5 |
| Path 113 | C00117->C00130:[10->8,12->3,6->17,7->12,8->11,8->13] | 0.60 | 0.0 | 21 | 32 | 0 | 21 |
| Path 114 | C00117->C00130:[10->8,12->3,6->17,7->10,7->11,7->12,7->4,7->5,8->13] | 0.90 | 0.0 | 27 | 51 | 0 | 27 |
| Path 115 | C00117->C00130:[10->8,12->3,6->10,6->11,6->17,6->4,7->12,8->10,8->11,8->13,8->4] | 0.80 | 0.0 | 29 | 35 | 0 | 29 |
| Path 116 | C00117->C00130:[8->10,8->11] | 0.20 | 0.0 | 18 | 21 | 0 | 18 |
| Path 117 | C00117->C00130:[10->8,12->3,6->17,7->12,8->11,8->13] | 0.60 | 0.0 | 15 | 18 | 0 | 15 |
| Path 118 | C00117->C00130:[10->8,12->3,6->17,7->10,7->11,7->12,7->2,7->5,8->13] | 0.90 | 0.0 | 23 | 29 | 0 | 23 |
| Path 119 | C00117->C00130:[10->8,12->3,6->17,7->10,7->11,7->12,7->2,8->13] | 0.80 | 0.0 | 21 | 30 | 0 | 21 |
| Path 120 | C00117->C00130:[10->8,12->3,6->17,7->10,7->12,7->4,8->13] | 0.70 | 0.0 | 17 | 22 | 0 | 17 |
| Path 121 | C00117->C00130:[10->12,12->17,6->3,7->8,8->11,8->13] | 0.60 | 0.0 | 19 | 39 | 0 | 19 |
| Path 122 | C00117->C00130:[10->8,12->3,6->17,7->12,8->10,8->11,8->13] | 0.70 | 0.0 | 15 | 16 | 0 | 15 |
| Path 123 | C00117->C00130:[10->8,12->3,6->17,7->10,7->11,7->12,7->4,8->13] | 0.80 | 0.0 | 21 | 28 | 0 | 21 |
| Path 124 | C00117->C00130:[10->8,12->3,6->17,7->12,8->11,8->13] | 0.60 | 0.0 | 20 | 30 | 0 | 20 |
| Path 125 | C00117->C00130:[10->8,12->3,6->17,7->10,7->11,7->12,7->4,8->13] | 0.80 | 0.0 | 21 | 40 | 0 | 21 |
| Path 126 | C00117->C00130:[10->8,12->3,6->17,7->12,8->11,8->13] | 0.60 | 0.0 | 16 | 30 | 0 | 16 |
| Path 127 | C00117->C00130:[10->8,12->3,6->17,7->10,7->11,7->12,7->4,8->13] | 0.80 | 0.0 | 21 | 38 | 0 | 21 |
| Path 128 | C00117->C00130:[10->8,12->3,6->17,7->12,8->11,8->13] | 0.60 | 0.0 | 16 | 18 | 0 | 16 |
| Path 129 | C00117->C00130:[10->8,12->3,6->17,7->12,8->11,8->13] | 0.60 | 0.0 | 20 | 29 | 0 | 20 |
| Path 130 | C00117->C00130:[10->8,12->3,6->11,6->17,7->12,8->13] | 0.60 | 0.0 | 15 | 19 | 0 | 15 |
| Path 131 | C00117->C00130:[10->8,12->3,6->17,7->10,7->11,7->12,7->4,8->13] | 0.80 | 0.0 | 22 | 41 | 0 | 22 |
| Path 132 | C00117->C00130:[10->8,12->3,6->17,7->10,7->11,7->12,7->2,7->5,8->13] | 0.90 | 0.0 | 25 | 32 | 0 | 25 |
| Path 133 | C00117->C00130:[10->8,12->3,6->17,7->12,8->11,8->13] | 0.60 | 0.0 | 15 | 22 | 0 | 15 |
| Path 134 | C00117->C00130:[10->8,12->3,6->17,7->10,7->12,8->13] | 0.60 | 0.0 | 14 | 19 | 0 | 14 |
| Path 135 | C00117->C00130:[10->8,12->3,6->11,6->17,7->12,8->13] | 0.60 | 0.0 | 16 | 28 | 0 | 16 |
| Path 136 | C00117->C00130:[10->8,12->3,6->17,7->12,8->11,8->13] | 0.60 | 0.0 | 24 | 34 | 0 | 24 |
| Path 137 | C00117->C00130:[8->11] | 0.10 | 0.0 | 11 | 15 | 0 | 11 |
| Path 138 | C00117->C00130:[10->8,12->3,6->17,7->12,8->11,8->13] | 0.60 | 0.0 | 15 | 21 | 0 | 15 |
| Path 139 | C00117->C00130:[10->8,12->3,6->17,7->12,8->11,8->13] | 0.60 | 0.0 | 21 | 29 | 0 | 21 |
| Path 140 | C00117->C00130:[10->8,12->3,6->17,7->12,8->11,8->13] | 0.60 | 0.0 | 19 | 25 | 0 | 19 |
| Path 141 | C00117->C00130:[10->8,12->3,6->17,7->12,8->11,8->13] | 0.60 | 0.0 | 23 | 33 | 0 | 23 |
| Path 142 | C00117->C00130:[10->8,12->3,6->17,7->12,8->11,8->13] | 0.60 | 0.0 | 28 | 36 | 0 | 28 |
| Path 143 | C00117->C00130:[10->8,12->3,6->17,7->10,7->11,7->12,7->4,8->13] | 0.80 | 0.0 | 25 | 38 | 0 | 25 |
| Path 144 | C00117->C00130:[10->8,12->3,6->17,7->12,8->11,8->13] | 0.60 | 0.0 | 13 | 23 | 0 | 13 |
| Path 145 | C00117->C00130:[10->8,12->3,6->17,7->10,7->12,8->13] | 0.60 | 0.0 | 15 | 30 | 0 | 15 |
| Path 146 | C00117->C00130:[10->8,12->3,6->17,7->12,8->11,8->13] | 0.60 | 0.0 | 22 | 30 | 0 | 22 |
| Path 147 | C00117->C00130:[8->11] | 0.10 | 0.0 | 13 | 15 | 0 | 13 |
| Path 148 | C00117->C00130:[10->8,12->3,6->17,7->10,7->11,7->12,7->2,8->13] | 0.80 | 0.0 | 27 | 48 | 0 | 27 |
| Path 149 | C00117->C00130:[10->8,12->3,6->17,7->12,8->11,8->13] | 0.60 | 0.0 | 22 | 31 | 0 | 22 |
| Path 150 | C00117->C00130:[10->8,12->3,6->17,7->10,7->11,7->12,7->2,8->13] | 0.80 | 0.0 | 22 | 30 | 0 | 22 |
| Path 151 | C00117->C00130:[10->8,12->3,6->17,7->12,8->13] | 0.50 | 0.0 | 5 | 8 | 0 | 5 |
| Path 152 | C00117->C00130:[10->8,12->3,6->17,7->10,7->12,7->4,8->13] | 0.70 | 0.0 | 19 | 37 | 0 | 19 |
| Path 153 | C00117->C00130:[8->11] | 0.10 | 0.0 | 13 | 18 | 0 | 13 |
| Path 154 | C00117->C00130:[10->8,12->3,6->17,7->11,7->12,8->13] | 0.60 | 0.0 | 12 | 16 | 0 | 12 |
| Path 155 | C00117->C00130:[10->8,12->3,6->17,7->10,7->11,7->12,7->4,8->13] | 0.80 | 0.0 | 30 | 35 | 0 | 30 |
| Path 156 | C00117->C00130:[10->8,12->3,6->17,7->12,8->11,8->13] | 0.60 | 0.0 | 19 | 24 | 0 | 19 |
| Path 157 | C00117->C00130:[10->8,12->3,6->17,7->12,8->11,8->13] | 0.60 | 0.0 | 28 | 34 | 0 | 28 |
| Path 158 | C00117->C00130:[10->8,12->3,6->17,7->12,8->11,8->13] | 0.60 | 0.0 | 20 | 22 | 0 | 20 |
| Path 159 | C00117->C00130:[10->8,12->3,6->17,7->12,8->11,8->13] | 0.60 | 0.0 | 23 | 33 | 0 | 23 |
| Path 160 | C00117->C00130:[10->8,12->3,6->17,7->10,7->11,7->12,8->13] | 0.70 | 0.0 | 19 | 40 | 0 | 19 |
| Path 161 | C00117->C00130:[10->8,12->3,6->17,7->12,8->11,8->13] | 0.60 | 0.0 | 12 | 14 | 0 | 12 |
| Path 162 | C00117->C00130:[10->8,12->3,6->17,7->10,7->11,7->12,7->2,7->5,8->13] | 0.90 | 0.0 | 30 | 53 | 0 | 30 |
| Path 163 | C00117->C00130:[6->10,8->11] | 0.20 | 0.0 | 23 | 32 | 0 | 23 |
| Path 164 | C00117->C00130:[10->8,12->3,6->17,7->12,8->13] | 0.50 | 0.0 | 5 | 6 | 0 | 5 |
| Path 165 | C00117->C00130:[10->8,12->3,6->17,7->10,7->11,7->12,8->13] | 0.70 | 0.0 | 14 | 19 | 0 | 14 |
| Path 166 | C00117->C00130:[10->8,12->3,6->17,7->12,8->13] | 0.50 | 0.0 | 5 | 6 | 0 | 5 |
| Path 167 | C00117->C00130:[10->8,12->11,12->3,6->17,7->12,8->13] | 0.60 | 0.0 | 14 | 20 | 0 | 14 |
| Path 168 | C00117->C00130:[10->8,12->3,6->17,7->12,8->11,8->13] | 0.60 | 0.0 | 16 | 21 | 0 | 16 |
| Path 169 | C00117->C00130:[10->8,12->3,6->17,7->12,8->10,8->11,8->13] | 0.70 | 0.0 | 14 | 15 | 0 | 14 |
| Path 170 | C00117->C00130:[10->8,12->3,6->17,7->12,8->11,8->13] | 0.60 | 0.0 | 25 | 33 | 0 | 25 |
| Path 171 | C00117->C00130:[10->8,12->3,6->17,7->12,8->11,8->13] | 0.60 | 0.0 | 17 | 22 | 0 | 17 |
| Path 172 | C00117->C00130:[10->8,12->3,6->17,7->10,7->11,7->12,7->4,8->13] | 0.80 | 0.0 | 25 | 38 | 0 | 25 |
| Path 173 | C00117->C00130:[10->8,12->3,6->17,7->12,8->11,8->13] | 0.60 | 0.0 | 27 | 35 | 0 | 27 |
| Path 174 | C00117->C00130:[10->8,12->3,6->17,7->11,7->12,8->13] | 0.60 | 0.0 | 11 | 17 | 0 | 11 |
| Path 175 | C00117->C00130:[10->8,12->3,6->11,6->17,7->12,8->13] | 0.60 | 0.0 | 16 | 17 | 0 | 16 |
| Path 176 | C00117->C00130:[10->8,12->3,6->17,7->10,7->12,8->13] | 0.60 | 0.0 | 14 | 19 | 0 | 14 |
| Path 177 | C00117->C00130:[10->8,12->3,6->17,7->12,8->11,8->13] | 0.60 | 0.0 | 19 | 23 | 0 | 19 |
| Path 178 | C00117->C00130:[6->10,6->11,8->10,8->11] | 0.20 | 0.0 | 24 | 34 | 0 | 24 |
| Path 179 | C00117->C00130:[10->8,12->3,6->17,7->12,8->10,8->11,8->13,8->4] | 0.80 | 0.0 | 29 | 33 | 0 | 29 |
| Path 180 | C00117->C00130:[10->8,12->3,6->17,7->10,7->12,7->4,8->13] | 0.70 | 0.0 | 22 | 34 | 0 | 22 |
| Path 181 | C00117->C00130:[10->8,12->3,6->17,7->12,8->11,8->13] | 0.60 | 0.0 | 20 | 24 | 0 | 20 |
| Path 182 | C00117->C00130:[10->11,10->8,12->3,6->17,7->12,8->13] | 0.60 | 0.0 | 13 | 19 | 0 | 13 |
| Path 183 | C00117->C00130:[10->8,12->3,6->17,7->12,8->11,8->13] | 0.60 | 0.0 | 16 | 22 | 0 | 16 |
| Path 184 | C00117->C00130:[10->8,12->3,6->17,7->12,8->11,8->13] | 0.60 | 0.0 | 15 | 18 | 0 | 15 |
| Path 185 | C00117->C00130:[10->11,10->8,12->3,6->17,7->12,8->13] | 0.60 | 0.0 | 13 | 17 | 0 | 13 |
| Path 186 | C00117->C00130:[10->8,12->3,6->17,7->12,8->11,8->13] | 0.60 | 0.0 | 20 | 34 | 0 | 20 |
| Path 187 | C00117->C00130:[10->8,12->3,6->17,7->10,7->11,7->12,7->2,7->5,8->13] | 0.90 | 0.0 | 25 | 45 | 0 | 25 |
| Path 188 | C00117->C00130:[10->8,12->3,6->17,7->10,7->11,7->12,7->2,8->13] | 0.80 | 0.0 | 27 | 35 | 0 | 27 |
| Path 189 | C00117->C00130:[10->8,12->3,6->17,7->11,7->12,8->13] | 0.60 | 0.0 | 11 | 16 | 0 | 11 |
| Path 190 | C00117->C00130:[10->8,12->3,6->17,7->10,7->11,7->12,7->4,7->5,8->13] | 0.90 | 0.0 | 24 | 41 | 0 | 24 |
| Path 191 | C00117->C00130:[10->8,12->3,6->17,7->10,7->11,7->12,8->13] | 0.70 | 0.0 | 19 | 38 | 0 | 19 |
| Path 192 | C00117->C00130:[10->8,12->11,12->3,6->17,7->12,8->11,8->13] | 0.60 | 0.0 | 26 | 37 | 0 | 26 |
| Path 193 | C00117->C00130:[10->8,12->3,6->17,7->10,7->11,7->12,8->13] | 0.70 | 0.0 | 19 | 29 | 0 | 19 |
| Path 194 | C00117->C00130:[10->8,12->3,6->17,7->12,8->11,8->13] | 0.60 | 0.0 | 21 | 26 | 0 | 21 |
| Path 195 | C00117->C00130:[10->8,12->3,6->17,7->10,7->12,7->4,8->13] | 0.70 | 0.0 | 19 | 37 | 0 | 19 |
| Path 196 | C00117->C00130:[10->8,12->3,6->17,7->12,8->11,8->13] | 0.60 | 0.0 | 23 | 29 | 0 | 23 |
| Path 197 | C00117->C00130:[10->8,12->3,6->17,7->10,7->11,7->12,7->4,7->5,8->13] | 0.90 | 0.0 | 21 | 28 | 0 | 21 |
| Path 198 | C00117->C00130:[10->8,12->3,6->17,7->10,7->11,7->12,7->4,8->13] | 0.80 | 0.0 | 19 | 26 | 0 | 19 |
| Path 199 | C00117->C00130:[10->8,12->3,6->17,7->12,8->11,8->13] | 0.60 | 0.0 | 20 | 31 | 0 | 20 |
| Path 200 | C00117->C00130:[10->8,12->3,6->17,7->10,7->12,7->4,8->13] | 0.70 | 0.0 | 21 | 29 | 0 | 21 |
| Path 201 | C00117->C00130:[10->8,12->3,6->17,7->12,8->11,8->13] | 0.60 | 0.0 | 25 | 30 | 0 | 25 |
| Path 202 | C00117->C00130:[10->8,12->3,6->17,7->12,8->10,8->13] | 0.60 | 0.0 | 16 | 23 | 0 | 16 |
| Path 203 | C00117->C00130:[10->8,12->3,6->17,7->10,7->11,7->12,7->4,8->13] | 0.80 | 0.0 | 33 | 38 | 0 | 33 |
| Path 204 | C00117->C00130:[10->8,12->3,6->17,7->12,8->11,8->13] | 0.60 | 0.0 | 25 | 34 | 0 | 25 |
| Path 205 | C00117->C00130:[10->11,10->8,12->3,6->17,7->12,8->13] | 0.60 | 0.0 | 15 | 19 | 0 | 15 |
| Path 206 | C00117->C00130:[6->10,6->11,8->10,8->11] | 0.20 | 0.0 | 22 | 34 | 0 | 22 |
| Path 207 | C00117->C00130:[10->8,12->3,6->10,6->17,7->11,7->12,7->4,8->13] | 0.80 | 0.0 | 34 | 42 | 0 | 34 |
| Path 208 | C00117->C00130:[10->8,12->3,6->17,7->12,8->11,8->13] | 0.60 | 0.0 | 13 | 18 | 0 | 13 |
| Path 209 | C00117->C00130:[10->8,12->3,6->17,7->12,8->11,8->13] | 0.60 | 0.0 | 15 | 18 | 0 | 15 |
| Path 210 | C00117->C00130:[10->8,12->3,6->17,7->10,7->11,7->12,7->4,8->13] | 0.80 | 0.0 | 23 | 42 | 0 | 23 |
| Path 211 | C00117->C00130:[10->8,12->3,6->17,7->10,7->11,7->12,7->2,8->13] | 0.80 | 0.0 | 24 | 31 | 0 | 24 |
| Path 212 | C00117->C00130:[10->8,12->3,6->17,7->10,7->12,8->13] | 0.60 | 0.0 | 14 | 21 | 0 | 14 |
| Path 213 | C00117->C00130:[10->8,12->10,12->3,6->17,7->12,8->13] | 0.60 | 0.0 | 16 | 36 | 0 | 16 |
| Path 214 | C00117->C00130:[8->10,8->11] | 0.20 | 0.0 | 23 | 27 | 0 | 23 |
| Path 215 | C00117->C00130:[10->8,12->3,6->17,7->12,8->11,8->13] | 0.60 | 0.0 | 19 | 23 | 0 | 19 |
| Path 216 | C00117->C00130:[10->8,12->3,6->17,7->10,7->11,7->12,8->13] | 0.70 | 0.0 | 17 | 21 | 0 | 17 |
| Path 217 | C00117->C00130:[10->8,12->3,6->17,7->10,7->11,7->12,7->4,7->5,8->13] | 0.90 | 0.0 | 23 | 43 | 0 | 23 |
| Path 218 | C00117->C00130:[10->8,12->3,6->17,7->12,8->11,8->13] | 0.60 | 0.0 | 25 | 34 | 0 | 25 |
| Path 219 | C00117->C00130:[10->8,12->3,6->17,7->12,8->11,8->13] | 0.60 | 0.0 | 14 | 19 | 0 | 14 |
| Path 220 | C00117->C00130:[10->8,12->3,6->17,7->10,7->12,8->13] | 0.60 | 0.0 | 16 | 31 | 0 | 16 |
| Path 221 | C00117->C00130:[10->8,12->3,6->17,7->10,7->11,7->12,7->2,7->5,8->13] | 0.90 | 0.0 | 29 | 53 | 0 | 29 |
| Path 222 | C00117->C00130:[10->8,12->3,6->17,7->12,8->11,8->13] | 0.60 | 0.0 | 15 | 23 | 0 | 15 |
| Path 223 | C00117->C00130:[10->8,12->3,6->17,7->12,8->11,8->13] | 0.60 | 0.0 | 27 | 37 | 0 | 27 |
| Path 224 | C00117->C00130:[10->8,12->3,6->17,7->10,7->12,7->4,8->13] | 0.70 | 0.0 | 19 | 35 | 0 | 19 |
| Path 225 | C00117->C00130:[10->8,12->3,6->17,7->12,8->11,8->13] | 0.60 | 0.0 | 14 | 24 | 0 | 14 |
| Path 226 | C00117->C00130:[10->8,12->3,6->17,7->12,8->11,8->13] | 0.60 | 0.0 | 17 | 22 | 0 | 17 |
| Path 227 | C00117->C00130:[10->8,12->3,6->17,7->12,8->11,8->13] | 0.60 | 0.0 | 19 | 26 | 0 | 19 |
| Path 228 | C00117->C00130:[10->8,12->3,6->17,7->11,7->12,8->13] | 0.60 | 0.0 | 11 | 14 | 0 | 11 |
| Path 229 | C00117->C00130:[10->8,12->3,6->17,7->12,8->11,8->13] | 0.60 | 0.0 | 16 | 26 | 0 | 16 |
| Path 230 | C00117->C00130:[10->8,12->3,6->17,7->12,8->11,8->13] | 0.60 | 0.0 | 21 | 27 | 0 | 21 |
| Path 231 | C00117->C00130:[10->11,10->8,12->3,6->17,7->12,8->13] | 0.60 | 0.0 | 13 | 16 | 0 | 13 |
| Path 232 | C00117->C00130:[10->8,12->3,6->17,7->10,7->12,7->4,8->13] | 0.70 | 0.0 | 18 | 24 | 0 | 18 |
| Path 233 | C00117->C00130:[10->8,12->3,6->10,6->11,6->17,6->4,7->12,8->10,8->11,8->13,8->4] | 0.80 | 0.0 | 28 | 35 | 0 | 28 |
| Path 234 | C00117->C00130:[10->8,12->3,6->10,6->11,6->17,6->4,7->12,8->13] | 0.80 | 0.0 | 32 | 45 | 0 | 32 |
| Path 235 | C00117->C00130:[10->8,12->3,6->17,7->10,7->11,7->12,7->4,7->5,8->13] | 0.90 | 0.0 | 22 | 30 | 0 | 22 |
| Path 236 | C00117->C00130:[10->8,12->3,6->17,7->10,7->11,7->12,7->2,7->5,8->13] | 0.90 | 0.0 | 25 | 32 | 0 | 25 |
| Path 237 | C00117->C00130:[10->8,12->3,6->17,7->10,7->11,7->12,7->4,8->13] | 0.80 | 0.0 | 22 | 29 | 0 | 22 |
| Path 238 | C00117->C00130:[10->8,12->3,6->17,7->11,7->12,8->13] | 0.60 | 0.0 | 11 | 13 | 0 | 11 |
| Path 239 | C00117->C00130:[10->8,12->3,6->17,7->10,7->11,7->12,7->2,7->5,8->13] | 0.90 | 0.0 | 26 | 43 | 0 | 26 |
| Path 240 | C00117->C00130:[10->8,12->3,6->17,7->12,8->11,8->13] | 0.60 | 0.0 | 20 | 25 | 0 | 20 |
| Path 241 | C00117->C00130:[10->8,12->3,6->17,7->12,8->11,8->13] | 0.60 | 0.0 | 21 | 32 | 0 | 21 |
| Path 242 | C00117->C00130:[10->8,12->3,6->17,7->10,7->12,7->4,8->13] | 0.70 | 0.0 | 19 | 25 | 0 | 19 |
| Path 243 | C00117->C00130:[10->8,12->3,6->17,7->10,7->11,7->12,7->4,8->13] | 0.80 | 0.0 | 32 | 36 | 0 | 32 |
| Path 244 | C00117->C00130:[10->8,12->3,6->17,7->12,8->11,8->13] | 0.60 | 0.0 | 26 | 33 | 0 | 26 |
| Path 245 | C00117->C00130:[8->11] | 0.10 | 0.0 | 13 | 24 | 0 | 13 |
| Path 246 | C00117->C00130:[10->8,12->3,6->17,7->12,8->11,8->13] | 0.60 | 0.0 | 17 | 26 | 0 | 17 |
| Path 247 | C00117->C00130:[10->8,12->3,6->10,6->17,7->12,8->11,8->13,8->4] | 0.80 | 0.0 | 31 | 41 | 0 | 31 |
| Path 248 | C00117->C00130:[10->8,12->3,6->17,7->12,8->11,8->13] | 0.60 | 0.0 | 25 | 33 | 0 | 25 |
| Path 249 | C00117->C00130:[10->12,12->17,6->3,7->8,8->11,8->13] | 0.60 | 0.0 | 20 | 37 | 0 | 20 |
| Path 250 | C00117->C00130:[10->8,12->3,6->17,7->12,8->11,8->13] | 0.60 | 0.0 | 16 | 23 | 0 | 16 |
| Path 251 | C00117->C00130:[10->8,12->3,6->17,7->10,7->11,7->12,7->2,7->5,8->13] | 0.90 | 0.0 | 25 | 42 | 0 | 25 |
| Path 252 | C00117->C00130:[10->8,12->3,6->17,7->10,7->11,7->12,7->4,8->13] | 0.80 | 0.0 | 22 | 28 | 0 | 22 |
| Path 253 | C00117->C00130:[10->8,12->3,6->17,7->12,8->11,8->13] | 0.60 | 0.0 | 26 | 34 | 0 | 26 |
| Path 254 | C00117->C00130:[10->8,12->3,6->17,7->12,8->11,8->13] | 0.60 | 0.0 | 14 | 28 | 0 | 14 |
| Path 255 | C00117->C00130:[10->8,12->3,6->17,7->10,7->11,7->12,7->4,7->5,8->13] | 0.90 | 0.0 | 24 | 31 | 0 | 24 |
| Path 256 | C00117->C00130:[10->8,12->3,6->17,7->12,8->11,8->13] | 0.60 | 0.0 | 28 | 33 | 0 | 28 |
| Path 257 | C00117->C00130:[10->8,12->3,6->17,7->12,8->11,8->13] | 0.60 | 0.0 | 20 | 22 | 0 | 20 |
| Path 258 | C00117->C00130:[10->8,12->3,6->17,7->12,8->11,8->13] | 0.60 | 0.0 | 15 | 20 | 0 | 15 |
| Path 259 | C00117->C00130:[10->8,12->3,6->17,7->12,8->11,8->13] | 0.60 | 0.0 | 13 | 15 | 0 | 13 |
| Path 260 | C00117->C00130:[10->8,12->3,6->17,7->10,7->12,7->4,8->13] | 0.70 | 0.0 | 19 | 28 | 0 | 19 |
| Path 261 | C00117->C00130:[10->8,12->3,6->17,7->12,8->11,8->13] | 0.60 | 0.0 | 19 | 21 | 0 | 19 |
| Path 262 | C00117->C00130:[10->8,12->3,6->17,7->12,8->11,8->13] | 0.60 | 0.0 | 21 | 23 | 0 | 21 |
| Path 263 | C00117->C00130:[10->8,12->3,6->17,7->10,7->11,7->12,7->4,8->13] | 0.80 | 0.0 | 22 | 28 | 0 | 22 |
| Path 264 | C00117->C00130:[10->8,12->3,6->17,7->12,8->11,8->13] | 0.60 | 0.0 | 21 | 36 | 0 | 21 |
| Path 265 | C00117->C00130:[10->8,12->3,6->17,7->12,8->11,8->13] | 0.60 | 0.0 | 13 | 14 | 0 | 13 |
| Path 266 | C00117->C00130:[10->8,12->3,6->17,7->12,8->11,8->13] | 0.60 | 0.0 | 21 | 25 | 0 | 21 |
| Path 267 | C00117->C00130:[10->8,12->3,6->17,7->10,7->11,7->12,7->2,7->5,8->13] | 0.90 | 0.0 | 23 | 29 | 0 | 23 |
| Path 268 | C00117->C00130:[10->8,12->3,6->17,7->12,8->11,8->13] | 0.60 | 0.0 | 16 | 21 | 0 | 16 |
| Path 269 | C00117->C00130:[10->8,12->3,6->10,6->11,6->17,6->4,7->12,8->13] | 0.80 | 0.0 | 31 | 35 | 0 | 31 |
| Path 270 | C00117->C00130:[10->8,12->3,6->17,7->12,8->11,8->13] | 0.60 | 0.0 | 23 | 28 | 0 | 23 |
| Path 271 | C00117->C00130:[10->8,12->3,6->17,7->12,8->11,8->13] | 0.60 | 0.0 | 13 | 23 | 0 | 13 |
| Path 272 | C00117->C00130:[10->8,12->3,6->17,7->12,8->11,8->13] | 0.60 | 0.0 | 15 | 20 | 0 | 15 |
| Path 273 | C00117->C00130:[10->8,12->3,6->17,7->10,7->11,7->12,7->2,8->13] | 0.80 | 0.0 | 23 | 31 | 0 | 23 |
| Path 274 | C00117->C00130:[10->8,12->3,6->17,7->10,7->12,8->13] | 0.60 | 0.0 | 14 | 18 | 0 | 14 |
| Path 275 | C00117->C00130:[10->8,12->3,6->17,7->12,8->11,8->13] | 0.60 | 0.0 | 18 | 26 | 0 | 18 |
| Path 276 | C00117->C00130:[10->8,12->3,6->17,7->12,8->11,8->13] | 0.60 | 0.0 | 25 | 37 | 0 | 25 |
| Path 277 | C00117->C00130:[10->8,12->3,6->17,7->10,7->12,7->4,8->13] | 0.70 | 0.0 | 18 | 25 | 0 | 18 |
| Path 278 | C00117->C00130:[10->8,12->3,6->10,6->11,6->17,6->4,7->12,8->10,8->11,8->13,8->4] | 0.80 | 0.0 | 28 | 37 | 0 | 28 |
| Path 279 | C00117->C00130:[10->8,12->3,6->17,7->12,8->11,8->13] | 0.60 | 0.0 | 16 | 31 | 0 | 16 |
| Path 280 | C00117->C00130:[10->8,12->3,6->17,7->10,7->11,7->12,7->4,7->5,8->13] | 0.90 | 0.0 | 22 | 30 | 0 | 22 |
| Path 281 | C00117->C00130:[10->8,12->3,6->17,7->12,8->11,8->13] | 0.60 | 0.0 | 26 | 35 | 0 | 26 |
| Path 282 | C00117->C00130:[10->8,12->3,6->17,7->12,8->11,8->13] | 0.60 | 0.0 | 20 | 31 | 0 | 20 |
| Path 283 | C00117->C00130:[10->8,12->3,6->17,7->10,7->11,7->12,7->4,8->13] | 0.80 | 0.0 | 33 | 43 | 0 | 33 |
| Path 284 | C00117->C00130:[10->8,12->3,6->17,7->10,7->11,7->12,7->4,8->13] | 0.80 | 0.0 | 22 | 29 | 0 | 22 |
| Path 285 | C00117->C00130:[10->8,12->3,6->17,7->12,8->11,8->13] | 0.60 | 0.0 | 14 | 19 | 0 | 14 |
| Path 286 | C00117->C00130:[6->10,6->11] | 0.20 | 0.0 | 23 | 31 | 0 | 23 |
| Path 287 | C00117->C00130:[10->8,12->3,6->17,7->12,8->11,8->13] | 0.60 | 0.0 | 19 | 34 | 0 | 19 |
| Path 288 | C00117->C00130:[10->8,12->3,6->10,6->17,7->12,8->11,8->13,8->4] | 0.80 | 0.0 | 32 | 41 | 0 | 32 |
| Path 289 | C00117->C00130:[10->8,12->3,6->17,7->12,8->11,8->13] | 0.60 | 0.0 | 12 | 22 | 0 | 12 |
| Path 290 | C00117->C00130:[10->8,12->3,6->17,7->12,8->11,8->13] | 0.60 | 0.0 | 24 | 32 | 0 | 24 |
| Path 291 | C00117->C00130:[10->8,12->3,6->17,7->12,8->11,8->13] | 0.60 | 0.0 | 29 | 37 | 0 | 29 |
| Path 292 | C00117->C00130:[10->8,12->3,6->11,6->17,7->12,8->13] | 0.60 | 0.0 | 15 | 16 | 0 | 15 |
| Path 293 | C00117->C00130:[10->8,12->3,6->17,7->10,7->11,7->12,7->4,8->13] | 0.80 | 0.0 | 21 | 29 | 0 | 21 |
| Path 294 | C00117->C00130:[10->8,12->3,6->17,7->10,7->11,7->12,7->4,8->13] | 0.80 | 0.0 | 31 | 44 | 0 | 31 |
| Path 295 | C00117->C00130:[10->8,12->3,6->17,7->12,8->11,8->13] | 0.60 | 0.0 | 15 | 20 | 0 | 15 |
| Path 296 | C00117->C00130:[10->8,12->3,6->17,7->12,8->11,8->13] | 0.60 | 0.0 | 20 | 31 | 0 | 20 |
| Path 297 | C00117->C00130:[10->8,12->3,6->17,7->12,8->11,8->13] | 0.60 | 0.0 | 13 | 15 | 0 | 13 |
| Path 298 | C00117->C00130:[10->12,12->17,6->3,7->8,8->11,8->13] | 0.60 | 0.0 | 18 | 36 | 0 | 18 |
| Path 299 | C00117->C00130:[10->8,12->3,6->17,7->10,7->12,8->13] | 0.60 | 0.0 | 14 | 17 | 0 | 14 |
| Path 300 | C00117->C00130:[8->11] | 0.10 | 0.0 | 12 | 19 | 0 | 12 |
| Path 301 | C00117->C00130:[10->8,12->3,6->17,7->10,7->11,7->12,7->4,8->13] | 0.80 | 0.0 | 33 | 46 | 0 | 33 |
| Path 302 | C00117->C00130:[10->8,12->3,6->17,7->12,8->11,8->13] | 0.60 | 0.0 | 26 | 42 | 0 | 26 |
| Path 303 | C00117->C00130:[10->8,12->3,6->17,7->12,8->11,8->13] | 0.60 | 0.0 | 20 | 30 | 0 | 20 |
| Path 304 | C00117->C00130:[10->8,12->3,6->17,7->12,8->11,8->13] | 0.60 | 0.0 | 25 | 32 | 0 | 25 |
| Path 305 | C00117->C00130:[8->11] | 0.10 | 0.0 | 8 | 10 | 0 | 8 |
| Path 306 | C00117->C00130:[10->8,12->3,6->17,7->12,8->11,8->13] | 0.60 | 0.0 | 25 | 30 | 0 | 25 |
| Path 307 | C00117->C00130:[10->8,12->3,6->17,7->12,8->11,8->13] | 0.60 | 0.0 | 16 | 30 | 0 | 16 |
| Path 308 | C00117->C00130:[10->8,12->3,6->17,7->12,8->10,8->11,8->13,8->4] | 0.80 | 0.0 | 27 | 36 | 0 | 27 |
| Path 309 | C00117->C00130:[10->8,12->3,6->17,7->12,8->11,8->13] | 0.60 | 0.0 | 21 | 34 | 0 | 21 |
| Path 310 | C00117->C00130:[10->8,12->3,6->17,7->10,7->11,7->12,7->2,7->5,8->13] | 0.90 | 0.0 | 26 | 34 | 0 | 26 |
| Path 311 | C00117->C00130:[10->8,12->3,6->17,7->11,7->12,8->13] | 0.60 | 0.0 | 12 | 26 | 0 | 12 |
| Path 312 | C00117->C00130:[10->8,12->3,6->17,7->10,7->11,7->12,7->2,8->13] | 0.80 | 0.0 | 22 | 30 | 0 | 22 |
| Path 313 | C00117->C00130:[10->8,12->3,6->17,7->12,8->10,8->11,8->13] | 0.70 | 0.0 | 14 | 17 | 0 | 14 |
| Path 314 | C00117->C00130:[10->8,12->3,6->17,7->12,8->11,8->13] | 0.60 | 0.0 | 15 | 22 | 0 | 15 |
| Path 315 | C00117->C00130:[10->8,12->3,6->10,6->17,7->11,7->12,7->4,8->13] | 0.80 | 0.0 | 31 | 40 | 0 | 31 |
| Path 316 | C00117->C00130:[10->8,12->3,6->17,7->10,7->11,7->12,7->4,8->13] | 0.80 | 0.0 | 22 | 32 | 0 | 22 |
| Path 317 | C00117->C00130:[10->8,12->3,6->17,7->12,8->11,8->13] | 0.60 | 0.0 | 17 | 24 | 0 | 17 |
| Path 318 | C00117->C00130:[10->8,12->3,6->17,7->10,7->11,7->12,8->13] | 0.70 | 0.0 | 17 | 23 | 0 | 17 |
| Path 319 | C00117->C00130:[10->8,12->3,6->17,7->12,8->10,8->13] | 0.60 | 0.0 | 14 | 18 | 0 | 14 |
| Path 320 | C00117->C00130:[10->8,12->3,6->17,7->10,7->11,7->12,8->13] | 0.70 | 0.0 | 15 | 29 | 0 | 15 |
| Path 321 | C00117->C00130:[10->8,12->3,6->10,6->17,7->11,7->12,7->4,8->13] | 0.80 | 0.0 | 32 | 39 | 0 | 32 |
| Path 322 | C00117->C00130:[10->8,12->3,6->17,7->10,7->11,7->12,7->2,7->5,8->13] | 0.90 | 0.0 | 27 | 34 | 0 | 27 |
| Path 323 | C00117->C00130:[10->10,10->8,12->3,6->17,7->12,8->13] | 0.60 | 0.0 | 16 | 23 | 0 | 16 |
| Path 324 | C00117->C00130:[10->8,12->3,6->17,7->12,8->11,8->13] | 0.60 | 0.0 | 18 | 26 | 0 | 18 |
| Path 325 | C00117->C00130:[10->8,12->3,6->17,7->12,8->11,8->13] | 0.60 | 0.0 | 22 | 27 | 0 | 22 |
| Path 326 | C00117->C00130:[10->8,12->3,6->17,7->12,8->11,8->13] | 0.60 | 0.0 | 26 | 31 | 0 | 26 |
| Path 327 | C00117->C00130:[10->8,12->3,6->17,7->12,8->11,8->13] | 0.60 | 0.0 | 14 | 25 | 0 | 14 |
| Path 328 | C00117->C00130:[10->8,12->3,6->17,7->12,8->11,8->13] | 0.60 | 0.0 | 24 | 33 | 0 | 24 |
| Path 329 | C00117->C00130:[10->8,12->3,6->17,7->10,7->11,7->12,7->2,8->13] | 0.80 | 0.0 | 27 | 37 | 0 | 27 |
| Path 330 | C00117->C00130:[10->8,12->3,6->17,7->10,7->11,7->12,7->2,7->5,8->13] | 0.90 | 0.0 | 29 | 53 | 0 | 29 |
| Path 331 | C00117->C00130:[10->8,12->3,6->17,7->12,8->10,8->11,8->13] | 0.70 | 0.0 | 14 | 15 | 0 | 14 |
| Path 332 | C00117->C00130:[10->8,12->3,6->17,7->10,7->11,7->12,7->4,7->5,8->13] | 0.90 | 0.0 | 22 | 28 | 0 | 22 |
| Path 333 | C00117->C00130:[10->8,12->3,6->17,7->12,8->11,8->13] | 0.60 | 0.0 | 19 | 22 | 0 | 19 |
| Path 334 | C00117->C00130:[10->8,12->3,6->17,7->10,7->11,7->12,7->2,7->5,8->13] | 0.90 | 0.0 | 25 | 45 | 0 | 25 |
| Path 335 | C00117->C00130:[10->8,12->3,6->17,7->10,7->11,7->12,8->13] | 0.70 | 0.0 | 19 | 27 | 0 | 19 |
| Path 336 | C00117->C00130:[10->8,12->3,6->17,7->12,8->11,8->13] | 0.60 | 0.0 | 15 | 19 | 0 | 15 |
| Path 337 | C00117->C00130:[10->8,12->3,6->17,7->10,7->11,7->12,7->4,8->13] | 0.80 | 0.0 | 20 | 28 | 0 | 20 |
| Path 338 | C00117->C00130:[10->10,10->8,12->3,6->17,7->12,8->13] | 0.60 | 0.0 | 16 | 21 | 0 | 16 |
| Path 339 | C00117->C00130:[10->8,12->3,6->17,7->12,8->11,8->13] | 0.60 | 0.0 | 14 | 28 | 0 | 14 |
| Path 340 | C00117->C00130:[8->11] | 0.10 | 0.0 | 13 | 20 | 0 | 13 |
| Path 341 | C00117->C00130:[10->8,12->11,12->3,6->17,7->12,8->13] | 0.60 | 0.0 | 14 | 30 | 0 | 14 |
| Path 342 | C00117->C00130:[10->8,12->3,6->17,7->12,8->11,8->13] | 0.60 | 0.0 | 19 | 21 | 0 | 19 |
| Path 343 | C00117->C00130:[10->8,12->3,6->10,6->17,7->11,7->12,7->4,8->13] | 0.80 | 0.0 | 33 | 50 | 0 | 33 |
| Path 344 | C00117->C00130:[10->8,12->3,6->17,7->10,7->12,7->4,8->13] | 0.70 | 0.0 | 20 | 28 | 0 | 20 |
| Path 345 | C00117->C00130:[10->8,12->3,6->17,7->12,8->11,8->13] | 0.60 | 0.0 | 18 | 23 | 0 | 18 |
| Path 346 | C00117->C00130:[10->8,12->3,6->17,7->12,8->11,8->13] | 0.60 | 0.0 | 20 | 27 | 0 | 20 |
| Path 347 | C00117->C00130:[10->8,12->3,6->17,7->10,7->11,7->12,7->4,8->13] | 0.80 | 0.0 | 29 | 33 | 0 | 29 |
| Path 348 | C00117->C00130:[10->8,12->3,6->17,7->10,7->12,7->5,8->13] | 0.70 | 0.0 | 18 | 23 | 0 | 18 |
| Path 349 | C00117->C00130:[10->8,12->3,6->17,7->10,7->12,7->4,8->13] | 0.70 | 0.0 | 16 | 21 | 0 | 16 |
| Path 350 | C00117->C00130:[10->8,12->3,6->17,7->12,8->11,8->13] | 0.60 | 0.0 | 16 | 21 | 0 | 16 |
| Path 351 | C00117->C00130:[10->8,12->3,6->17,7->10,7->11,7->12,7->4,8->13] | 0.80 | 0.0 | 20 | 26 | 0 | 20 |
| Path 352 | C00117->C00130:[10->8,12->3,6->17,7->12,8->11,8->13] | 0.60 | 0.0 | 24 | 37 | 0 | 24 |
| Path 353 | C00117->C00130:[10->8,12->3,6->17,7->12,8->11,8->13] | 0.60 | 0.0 | 17 | 21 | 0 | 17 |
| Path 354 | C00117->C00130:[10->8,12->3,6->17,7->10,7->11,7->12,7->2,8->13] | 0.80 | 0.0 | 27 | 34 | 0 | 27 |
| Path 355 | C00117->C00130:[8->11] | 0.10 | 0.0 | 10 | 17 | 0 | 10 |
| Path 356 | C00117->C00130:[10->8,12->3,6->17,7->12,8->11,8->13] | 0.60 | 0.0 | 18 | 25 | 0 | 18 |
| Path 357 | C00117->C00130:[10->8,12->3,6->17,7->10,7->11,7->12,7->4,8->13] | 0.80 | 0.0 | 31 | 47 | 0 | 31 |
| Path 358 | C00117->C00130:[10->8,12->3,6->17,7->12,8->11,8->13] | 0.60 | 0.0 | 18 | 24 | 0 | 18 |
| Path 359 | C00117->C00130:[10->8,12->3,6->17,7->12,8->11,8->13] | 0.60 | 0.0 | 15 | 26 | 0 | 15 |
| Path 360 | C00117->C00130:[10->8,12->3,6->17,7->10,7->12,8->13] | 0.60 | 0.0 | 14 | 19 | 0 | 14 |
| Path 361 | C00117->C00130:[10->8,12->3,6->17,7->10,7->12,7->4,8->13] | 0.70 | 0.0 | 16 | 24 | 0 | 16 |
| Path 362 | C00117->C00130:[10->8,12->3,6->17,7->10,7->11,7->12,7->4,8->13] | 0.80 | 0.0 | 30 | 36 | 0 | 30 |
| Path 363 | C00117->C00130:[10->11,10->8,12->3,6->17,7->12,8->13] | 0.60 | 0.0 | 12 | 13 | 0 | 12 |
| Path 364 | C00117->C00130:[10->8,12->3,6->17,7->12,8->11,8->13] | 0.60 | 0.0 | 14 | 16 | 0 | 14 |
| Path 365 | C00117->C00130:[10->8,12->3,6->17,7->12,8->11,8->13] | 0.60 | 0.0 | 13 | 18 | 0 | 13 |
| Path 366 | C00117->C00130:[8->11] | 0.10 | 0.0 | 9 | 16 | 0 | 9 |
| Path 367 | C00117->C00130:[6->10,6->11,8->10,8->11] | 0.20 | 0.0 | 22 | 29 | 0 | 22 |
| Path 368 | C00117->C00130:[10->8,12->3,6->17,7->10,7->11,7->12,7->4,7->5,8->13] | 0.90 | 0.0 | 22 | 29 | 0 | 22 |
| Path 369 | C00117->C00130:[10->8,12->3,6->17,7->10,7->11,7->12,8->13] | 0.70 | 0.0 | 18 | 24 | 0 | 18 |
| Path 370 | C00117->C00130:[10->8,12->3,6->17,7->10,7->11,7->12,7->2,8->13] | 0.80 | 0.0 | 27 | 49 | 0 | 27 |
| Path 371 | C00117->C00130:[10->8,12->3,6->17,7->12,8->11,8->13] | 0.60 | 0.0 | 25 | 35 | 0 | 25 |
| Path 372 | C00117->C00130:[10->8,12->3,6->17,7->12,8->11,8->13] | 0.60 | 0.0 | 14 | 21 | 0 | 14 |
| Path 373 | C00117->C00130:[10->11,10->8,12->3,6->17,7->12,8->13] | 0.60 | 0.0 | 12 | 18 | 0 | 12 |
| Path 374 | C00117->C00130:[10->8,12->3,6->17,7->12,8->11,8->13] | 0.60 | 0.0 | 24 | 34 | 0 | 24 |
| Path 375 | C00117->C00130:[10->8,12->3,6->17,7->10,7->11,7->12,7->2,7->5,8->13] | 0.90 | 0.0 | 26 | 34 | 0 | 26 |
| Path 376 | C00117->C00130:[10->8,12->11,12->3,6->17,7->12,8->13] | 0.60 | 0.0 | 15 | 18 | 0 | 15 |
| Path 377 | C00117->C00130:[10->8,12->3,6->17,7->11,7->12,8->13] | 0.60 | 0.0 | 12 | 23 | 0 | 12 |
| Path 378 | C00117->C00130:[10->8,12->3,6->17,7->10,7->11,7->12,7->4,7->5,8->13] | 0.90 | 0.0 | 24 | 33 | 0 | 24 |
| Path 379 | C00117->C00130:[10->8,12->3,6->17,7->12,8->11,8->13] | 0.60 | 0.0 | 23 | 28 | 0 | 23 |
| Path 380 | C00117->C00130:[10->8,12->11,12->3,6->17,7->12,8->13] | 0.60 | 0.0 | 14 | 20 | 0 | 14 |
| Path 381 | C00117->C00130:[10->8,12->3,6->17,7->12,8->11,8->13] | 0.60 | 0.0 | 28 | 37 | 0 | 28 |
| Path 382 | C00117->C00130:[10->8,12->3,6->17,7->12,8->10,8->11,8->13] | 0.70 | 0.0 | 15 | 18 | 0 | 15 |
| Path 383 | C00117->C00130:[10->11,10->8,12->3,6->17,7->12,8->13] | 0.60 | 0.0 | 13 | 17 | 0 | 13 |
| Path 384 | C00117->C00130:[10->8,12->3,6->17,7->10,7->11,7->12,8->13] | 0.70 | 0.0 | 20 | 24 | 0 | 20 |
| Path 385 | C00117->C00130:[10->8,12->3,6->17,7->10,7->11,7->12,7->2,7->5,8->13] | 0.90 | 0.0 | 27 | 36 | 0 | 27 |
| Path 386 | C00117->C00130:[10->8,12->3,6->17,7->12,8->11,8->13] | 0.60 | 0.0 | 20 | 22 | 0 | 20 |
| Path 387 | C00117->C00130:[10->8,12->3,6->17,7->10,7->11,7->12,8->13] | 0.70 | 0.0 | 20 | 24 | 0 | 20 |
| Path 388 | C00117->C00130:[10->8,12->3,6->17,7->12,8->11,8->13] | 0.60 | 0.0 | 20 | 30 | 0 | 20 |
| Path 389 | C00117->C00130:[10->8,12->3,6->17,7->10,7->12,8->13] | 0.60 | 0.0 | 13 | 21 | 0 | 13 |
| Path 390 | C00117->C00130:[10->8,12->3,6->17,7->10,7->11,7->12,7->2,7->5,8->13] | 0.90 | 0.0 | 24 | 32 | 0 | 24 |
| Path 391 | C00117->C00130:[10->8,12->3,6->17,7->10,7->11,7->12,7->4,7->5,8->13] | 0.90 | 0.0 | 24 | 31 | 0 | 24 |
| Path 392 | C00117->C00130:[10->8,12->3,6->17,7->10,7->12,8->13] | 0.60 | 0.0 | 15 | 28 | 0 | 15 |
| Path 393 | C00117->C00130:[10->8,12->3,6->17,7->11,7->12,8->13] | 0.60 | 0.0 | 13 | 20 | 0 | 13 |
| Path 394 | C00117->C00130:[10->8,12->3,6->17,7->12,8->11,8->13] | 0.60 | 0.0 | 21 | 26 | 0 | 21 |
| Path 395 | C00117->C00130:[10->8,12->3,6->17,7->10,7->11,7->12,8->13] | 0.70 | 0.0 | 18 | 24 | 0 | 18 |
| Path 396 | C00117->C00130:[10->8,12->3,6->17,7->11,7->12,8->13] | 0.60 | 0.0 | 17 | 22 | 0 | 17 |
| Path 397 | C00117->C00130:[10->8,12->3,6->17,7->10,7->11,7->12,7->4,8->13] | 0.80 | 0.0 | 33 | 40 | 0 | 33 |
| Path 398 | C00117->C00130:[10->8,12->3,6->17,7->12,8->11,8->13] | 0.60 | 0.0 | 20 | 25 | 0 | 20 |
| Path 399 | C00117->C00130:[10->8,12->3,6->17,7->10,7->11,7->12,8->13] | 0.70 | 0.0 | 17 | 20 | 0 | 17 |
| Path 400 | C00117->C00130:[10->8,12->3,6->17,7->12,7->4,8->13] | 0.60 | 0.0 | 13 | 13 | 0 | 13 |
| Path 401 | C00117->C00130:[10->8,12->3,6->17,7->12,8->11,8->13] | 0.60 | 0.0 | 20 | 22 | 0 | 20 |
| Path 402 | C00117->C00130:[10->8,12->3,6->17,7->12,8->11,8->13] | 0.60 | 0.0 | 16 | 21 | 0 | 16 |
| Path 403 | C00117->C00130:[10->8,12->3,6->17,7->10,7->12,8->13] | 0.60 | 0.0 | 13 | 21 | 0 | 13 |
| Path 404 | C00117->C00130:[6->10,6->11] | 0.20 | 0.0 | 23 | 30 | 0 | 23 |
| Path 405 | C00117->C00130:[10->8,12->3,6->17,7->12,8->11,8->13] | 0.60 | 0.0 | 20 | 39 | 0 | 20 |
| Path 406 | C00117->C00130:[10->8,12->3,6->17,7->10,7->11,7->12,7->2,8->13] | 0.80 | 0.0 | 24 | 41 | 0 | 24 |
| Path 407 | C00117->C00130:[10->8,12->3,6->17,7->12,8->11,8->13] | 0.60 | 0.0 | 22 | 34 | 0 | 22 |
| Path 408 | C00117->C00130:[10->8,12->3,6->17,7->12,8->11,8->13] | 0.60 | 0.0 | 22 | 31 | 0 | 22 |
| Path 409 | C00117->C00130:[10->8,12->3,6->10,6->11,6->17,6->4,7->12,8->10,8->11,8->13,8->4] | 0.80 | 0.0 | 32 | 43 | 0 | 32 |
| Path 410 | C00117->C00130:[10->8,12->3,6->10,6->17,7->12,8->11,8->13,8->4] | 0.80 | 0.0 | 28 | 44 | 0 | 28 |
| Path 411 | C00117->C00130:[10->8,12->3,6->17,7->11,7->12,8->13] | 0.60 | 0.0 | 12 | 15 | 0 | 12 |
| Path 412 | C00117->C00130:[10->8,12->3,6->17,7->10,7->11,7->12,7->4,8->13] | 0.80 | 0.0 | 32 | 43 | 0 | 32 |
| Path 413 | C00117->C00130:[10->8,12->3,6->17,7->12,8->11,8->13] | 0.60 | 0.0 | 23 | 34 | 0 | 23 |
| Path 414 | C00117->C00130:[10->8,12->3,6->17,7->10,7->12,7->4,8->13] | 0.70 | 0.0 | 15 | 20 | 0 | 15 |
| Path 415 | C00117->C00130:[10->11,10->8,12->3,6->17,7->12,8->13] | 0.60 | 0.0 | 14 | 19 | 0 | 14 |
| Path 416 | C00117->C00130:[10->8,12->3,6->17,7->10,7->11,7->12,7->2,8->13] | 0.80 | 0.0 | 23 | 33 | 0 | 23 |
| Path 417 | C00117->C00130:[10->8,12->3,6->17,7->10,7->12,7->4,8->13] | 0.70 | 0.0 | 16 | 23 | 0 | 16 |
| Path 418 | C00117->C00130:[10->8,12->3,6->17,7->10,7->11,7->12,7->2,7->5,8->13] | 0.90 | 0.0 | 26 | 34 | 0 | 26 |
| Path 419 | C00117->C00130:[10->8,12->3,6->17,7->12,8->11,8->13] | 0.60 | 0.0 | 14 | 18 | 0 | 14 |
| Path 420 | C00117->C00130:[10->8,12->3,6->17,7->10,7->11,7->12,7->2,8->13] | 0.80 | 0.0 | 28 | 49 | 0 | 28 |
| Path 421 | C00117->C00130:[10->8,12->3,6->17,7->12,8->11,8->13] | 0.60 | 0.0 | 19 | 24 | 0 | 19 |
| Path 422 | C00117->C00130:[10->8,12->3,6->17,7->10,7->11,7->12,7->4,8->13] | 0.80 | 0.0 | 23 | 31 | 0 | 23 |
| Path 423 | C00117->C00130:[10->8,12->3,6->17,7->10,7->11,7->12,7->2,7->5,8->13] | 0.90 | 0.0 | 29 | 39 | 0 | 29 |
| Path 424 | C00117->C00130:[6->10,6->11,8->10,8->11] | 0.20 | 0.0 | 21 | 31 | 0 | 21 |
| Path 425 | C00117->C00130:[10->8,12->3,6->17,7->10,7->11,7->12,8->13] | 0.70 | 0.0 | 15 | 22 | 0 | 15 |
| Path 426 | C00117->C00130:[10->8,12->3,6->17,7->12,8->11,8->13] | 0.60 | 0.0 | 29 | 38 | 0 | 29 |
| Path 427 | C00117->C00130:[10->8,12->3,6->17,7->12,8->11,8->13] | 0.60 | 0.0 | 19 | 24 | 0 | 19 |
| Path 428 | C00117->C00130:[10->8,12->3,6->17,7->10,7->12,7->4,8->13] | 0.70 | 0.0 | 18 | 24 | 0 | 18 |
| Path 429 | C00117->C00130:[10->8,12->3,6->17,7->10,7->12,7->4,8->13] | 0.70 | 0.0 | 16 | 21 | 0 | 16 |
| Path 430 | C00117->C00130:[10->8,12->3,6->17,7->12,8->11,8->13] | 0.60 | 0.0 | 25 | 34 | 0 | 25 |
| Path 431 | C00117->C00130:[10->8,12->3,6->17,7->12,8->11,8->13] | 0.60 | 0.0 | 15 | 17 | 0 | 15 |
| Path 432 | C00117->C00130:[10->8,12->3,6->17,7->12,8->13] | 0.50 | 0.0 | 5 | 7 | 0 | 5 |
| Path 433 | C00117->C00130:[10->8,12->3,6->17,7->12,8->11,8->13] | 0.60 | 0.0 | 28 | 36 | 0 | 28 |
| Path 434 | C00117->C00130:[10->8,12->3,6->17,7->10,7->11,7->12,8->13] | 0.70 | 0.0 | 13 | 19 | 0 | 13 |
| Path 435 | C00117->C00130:[10->8,12->3,6->17,7->12,8->11,8->13] | 0.60 | 0.0 | 18 | 24 | 0 | 18 |
| Path 436 | C00117->C00130:[10->8,12->3,6->17,7->10,7->11,7->12,7->4,8->13] | 0.80 | 0.0 | 32 | 38 | 0 | 32 |
| Path 437 | C00117->C00130:[10->8,12->3,6->17,7->12,8->10,8->13,8->4] | 0.70 | 0.0 | 17 | 20 | 0 | 17 |
| Path 438 | C00117->C00130:[10->8,12->3,6->17,7->12,8->11,8->13] | 0.60 | 0.0 | 21 | 26 | 0 | 21 |
| Path 439 | C00117->C00130:[10->8,12->3,6->17,7->12,8->11,8->13] | 0.60 | 0.0 | 17 | 19 | 0 | 17 |
| Path 440 | C00117->C00130:[10->8,12->3,6->17,7->10,7->12,7->4,8->13] | 0.70 | 0.0 | 18 | 35 | 0 | 18 |
| Path 441 | C00117->C00130:[10->8,12->3,6->17,7->10,7->12,8->13] | 0.60 | 0.0 | 14 | 19 | 0 | 14 |
| Path 442 | C00117->C00130:[10->8,12->3,6->17,7->12,8->11,8->13] | 0.60 | 0.0 | 26 | 35 | 0 | 26 |
| Path 443 | C00117->C00130:[10->8,12->3,6->17,7->10,7->11,7->12,7->4,7->5,8->13] | 0.90 | 0.0 | 26 | 35 | 0 | 26 |
| Path 444 | C00117->C00130:[10->8,12->3,6->17,7->12,8->11,8->13] | 0.60 | 0.0 | 18 | 22 | 0 | 18 |
| Path 445 | C00117->C00130:[10->8,12->3,6->17,7->10,7->11,7->12,7->4,8->13] | 0.80 | 0.0 | 18 | 26 | 0 | 18 |
| Path 446 | C00117->C00130:[10->8,12->3,6->17,7->12,8->11,8->13] | 0.60 | 0.0 | 15 | 22 | 0 | 15 |
| Path 447 | C00117->C00130:[6->10,6->11] | 0.20 | 0.0 | 23 | 34 | 0 | 23 |
| Path 448 | C00117->C00130:[10->8,12->3,6->17,7->12,8->11,8->13] | 0.60 | 0.0 | 23 | 39 | 0 | 23 |
| Path 449 | C00117->C00130:[8->11] | 0.10 | 0.0 | 13 | 22 | 0 | 13 |
| Path 450 | C00117->C00130:[10->8,12->3,6->17,7->12,8->11,8->13] | 0.60 | 0.0 | 16 | 24 | 0 | 16 |
| Path 451 | C00117->C00130:[10->8,12->3,6->17,7->10,7->11,7->12,7->4,7->5,8->13] | 0.90 | 0.0 | 23 | 31 | 0 | 23 |
| Path 452 | C00117->C00130:[10->8,12->3,6->17,7->10,7->11,7->12,7->2,7->5,8->13] | 0.90 | 0.0 | 25 | 33 | 0 | 25 |
| Path 453 | C00117->C00130:[10->8,12->3,6->17,7->10,7->12,7->4,8->13] | 0.70 | 0.0 | 16 | 21 | 0 | 16 |
| Path 454 | C00117->C00130:[10->8,12->11,12->3,6->17,7->12,8->13] | 0.60 | 0.0 | 14 | 29 | 0 | 14 |
| Path 455 | C00117->C00130:[10->8,12->3,6->17,7->12,8->11,8->13] | 0.60 | 0.0 | 18 | 22 | 0 | 18 |
| Path 456 | C00117->C00130:[10->8,12->11,12->3,6->17,7->12,8->13] | 0.60 | 0.0 | 15 | 32 | 0 | 15 |
| Path 457 | C00117->C00130:[10->8,12->3,6->17,7->12,8->11,8->13] | 0.60 | 0.0 | 14 | 16 | 0 | 14 |
| Path 458 | C00117->C00130:[10->8,12->3,6->17,7->10,7->11,7->12,7->4,8->13] | 0.80 | 0.0 | 20 | 26 | 0 | 20 |
| Path 459 | C00117->C00130:[10->8,12->3,6->17,7->12,8->11,8->13] | 0.60 | 0.0 | 28 | 37 | 0 | 28 |
| Path 460 | C00117->C00130:[10->8,12->3,6->17,7->12,8->11,8->13] | 0.60 | 0.0 | 16 | 21 | 0 | 16 |
| Path 461 | C00117->C00130:[10->8,12->3,6->17,7->12,8->11,8->13] | 0.60 | 0.0 | 21 | 26 | 0 | 21 |
| Path 462 | C00117->C00130:[10->8,12->3,6->17,7->12,8->11,8->13] | 0.60 | 0.0 | 19 | 24 | 0 | 19 |
| Path 463 | C00117->C00130:[10->8,12->3,6->17,7->12,8->11,8->13] | 0.60 | 0.0 | 24 | 32 | 0 | 24 |
| Path 464 | C00117->C00130:[10->8,12->3,6->17,6->3,7->12,7->8,8->11,8->13] | 0.60 | 0.0 | 21 | 41 | 0 | 21 |
| Path 465 | C00117->C00130:[10->8,12->3,6->17,7->10,7->11,7->12,7->2,8->13] | 0.80 | 0.0 | 23 | 30 | 0 | 23 |
| Path 466 | C00117->C00130:[10->10,10->8,12->3,6->17,7->12,8->13] | 0.60 | 0.0 | 15 | 23 | 0 | 15 |
| Path 467 | C00117->C00130:[10->8,12->3,6->17,7->10,7->11,7->12,8->13] | 0.70 | 0.0 | 19 | 23 | 0 | 19 |
| Path 468 | C00117->C00130:[10->8,12->3,6->17,7->12,8->11,8->13] | 0.60 | 0.0 | 13 | 20 | 0 | 13 |
| Path 469 | C00117->C00130:[10->8,12->3,6->17,7->10,7->12,8->13] | 0.60 | 0.0 | 14 | 21 | 0 | 14 |
| Path 470 | C00117->C00130:[10->8,12->3,6->17,7->12,8->11,8->13] | 0.60 | 0.0 | 14 | 18 | 0 | 14 |
| Path 471 | C00117->C00130:[10->8,12->3,6->17,7->12,8->11,8->13] | 0.60 | 0.0 | 12 | 16 | 0 | 12 |
| Path 472 | C00117->C00130:[10->8,12->3,6->17,7->12,8->11,8->13] | 0.60 | 0.0 | 19 | 22 | 0 | 19 |
| Path 473 | C00117->C00130:[10->8,12->3,6->17,7->11,7->12,8->13] | 0.60 | 0.0 | 12 | 17 | 0 | 12 |
| Path 474 | C00117->C00130:[10->8,12->3,6->10,6->11,6->17,6->4,7->12,8->10,8->11,8->13,8->4] | 0.80 | 0.0 | 33 | 39 | 0 | 33 |
| Path 475 | C00117->C00130:[10->8,12->3,6->17,7->10,7->11,7->12,7->2,8->13] | 0.80 | 0.0 | 22 | 28 | 0 | 22 |
| Path 476 | C00117->C00130:[10->8,12->3,6->17,7->12,8->11,8->13] | 0.60 | 0.0 | 18 | 29 | 0 | 18 |
| Path 477 | C00117->C00130:[10->8,12->3,6->17,7->12,8->11,8->13] | 0.60 | 0.0 | 15 | 20 | 0 | 15 |
| Path 478 | C00117->C00130:[10->8,12->3,6->17,7->12,8->11,8->13] | 0.60 | 0.0 | 12 | 13 | 0 | 12 |
| Path 479 | C00117->C00130:[10->8,12->3,6->17,7->10,7->11,7->12,7->2,8->13] | 0.80 | 0.0 | 23 | 43 | 0 | 23 |
| Path 480 | C00117->C00130:[10->8,12->3,6->17,7->10,7->11,7->12,7->4,8->13] | 0.80 | 0.0 | 20 | 26 | 0 | 20 |
| Path 481 | C00117->C00130:[10->8,12->3,6->17,7->10,7->11,7->12,7->2,7->5,8->13] | 0.90 | 0.0 | 27 | 33 | 0 | 27 |
| Path 482 | C00117->C00130:[10->8,12->3,6->17,7->10,7->12,7->4,8->13] | 0.70 | 0.0 | 18 | 37 | 0 | 18 |
| Path 483 | C00117->C00130:[10->8,12->3,6->17,7->12,8->11,8->13] | 0.60 | 0.0 | 19 | 30 | 0 | 19 |
| Path 484 | C00117->C00130:[10->8,12->3,6->17,7->12,8->11,8->13] | 0.60 | 0.0 | 15 | 17 | 0 | 15 |
| Path 485 | C00117->C00130:[10->8,12->3,6->17,7->12,8->11,8->13] | 0.60 | 0.0 | 14 | 19 | 0 | 14 |
| Path 486 | C00117->C00130:[10->8,12->3,6->17,7->10,7->11,7->12,7->2,8->13] | 0.80 | 0.0 | 28 | 37 | 0 | 28 |
| Path 487 | C00117->C00130:[10->8,12->3,6->17,7->10,7->11,7->12,7->2,7->5,8->13] | 0.90 | 0.0 | 25 | 34 | 0 | 25 |
| Path 488 | C00117->C00130:[10->8,12->3,6->17,7->10,7->11,7->12,7->4,8->13] | 0.80 | 0.0 | 25 | 36 | 0 | 25 |
| Path 489 | C00117->C00130:[10->8,12->3,6->17,7->10,7->12,7->4,8->13] | 0.70 | 0.0 | 20 | 26 | 0 | 20 |
| Path 490 | C00117->C00130:[10->8,12->3,6->17,7->10,7->12,7->4,8->13] | 0.70 | 0.0 | 15 | 21 | 0 | 15 |
| Path 491 | C00117->C00130:[10->8,12->3,6->17,7->12,8->11,8->13] | 0.60 | 0.0 | 13 | 20 | 0 | 13 |
| Path 492 | C00117->C00130:[8->11] | 0.10 | 0.0 | 12 | 17 | 0 | 12 |
| Path 493 | C00117->C00130:[10->8,12->3,6->17,7->12,8->11,8->13] | 0.60 | 0.0 | 18 | 22 | 0 | 18 |
| Path 494 | C00117->C00130:[8->11] | 0.10 | 0.0 | 13 | 17 | 0 | 13 |
| Path 495 | C00117->C00130:[10->8,12->3,6->17,7->12,8->11,8->13] | 0.60 | 0.0 | 15 | 21 | 0 | 15 |
| Path 496 | C00117->C00130:[10->8,12->3,6->17,7->12,8->11,8->13] | 0.60 | 0.0 | 24 | 38 | 0 | 24 |
| Path 497 | C00117->C00130:[10->8,12->3,6->17,7->11,7->12,8->13] | 0.60 | 0.0 | 18 | 25 | 0 | 18 |
| Path 498 | C00117->C00130:[10->8,12->3,6->17,7->10,7->11,7->12,7->4,7->5,8->13] | 0.90 | 0.0 | 24 | 32 | 0 | 24 |
| Path 499 | C00117->C00130:[10->8,12->3,6->11,6->17,7->12,8->13] | 0.60 | 0.0 | 15 | 16 | 0 | 15 |
| Path 500 | C00117->C00130:[8->11] | 0.10 | 0.0 | 11 | 17 | 0 | 11 |
| Path 501 | C00117->C00130:[10->8,12->3,6->17,7->10,7->11,7->12,7->2,7->5,8->13] | 0.90 | 0.0 | 25 | 33 | 0 | 25 |
| Path 502 | C00117->C00130:[10->8,12->3,6->17,7->12,8->11,8->13] | 0.60 | 0.0 | 21 | 24 | 0 | 21 |
| Path 503 | C00117->C00130:[10->8,12->3,6->17,7->10,7->12,7->4,8->13] | 0.70 | 0.0 | 19 | 35 | 0 | 19 |
| Path 504 | C00117->C00130:[10->8,12->3,6->17,7->12,8->11,8->13] | 0.60 | 0.0 | 15 | 18 | 0 | 15 |
| Path 505 | C00117->C00130:[10->8,12->3,6->17,7->12,8->11,8->13] | 0.60 | 0.0 | 22 | 27 | 0 | 22 |
| Path 506 | C00117->C00130:[10->8,12->3,6->17,7->12,8->11,8->13] | 0.60 | 0.0 | 20 | 34 | 0 | 20 |
| Path 507 | C00117->C00130:[10->8,12->11,12->3,6->17,7->12,8->11,8->13] | 0.60 | 0.0 | 25 | 38 | 0 | 25 |
| Path 508 | C00117->C00130:[10->8,12->3,6->17,7->10,7->11,7->12,7->4,8->13] | 0.80 | 0.0 | 18 | 24 | 0 | 18 |
| Path 509 | C00117->C00130:[10->8,12->3,6->17,7->10,7->12,8->13] | 0.60 | 0.0 | 15 | 28 | 0 | 15 |
| Path 510 | C00117->C00130:[10->8,12->3,6->17,7->10,7->11,7->12,7->2,7->5,8->13] | 0.90 | 0.0 | 30 | 39 | 0 | 30 |
| Path 511 | C00117->C00130:[10->8,12->3,6->17,7->10,7->11,7->12,7->2,7->5,8->13] | 0.90 | 0.0 | 30 | 51 | 0 | 30 |
| Path 512 | C00117->C00130:[10->8,12->3,6->17,7->12,8->11,8->13] | 0.60 | 0.0 | 19 | 30 | 0 | 19 |
| Path 513 | C00117->C00130:[10->8,12->3,6->17,7->12,8->11,8->13] | 0.60 | 0.0 | 20 | 25 | 0 | 20 |
| Path 514 | C00117->C00130:[10->8,12->3,6->17,7->10,7->11,7->12,8->13] | 0.70 | 0.0 | 19 | 24 | 0 | 19 |
| Path 515 | C00117->C00130:[10->8,12->3,6->17,7->12,8->11,8->13] | 0.60 | 0.0 | 21 | 25 | 0 | 21 |
| Path 516 | C00117->C00130:[10->8,12->3,6->10,6->11,6->17,6->4,7->12,8->10,8->11,8->13,8->4] | 0.80 | 0.0 | 28 | 34 | 0 | 28 |
| Path 517 | C00117->C00130:[10->8,12->3,6->17,7->12,8->11,8->13] | 0.60 | 0.0 | 16 | 23 | 0 | 16 |
| Path 518 | C00117->C00130:[10->8,12->3,6->17,7->10,7->11,7->12,7->4,8->13] | 0.80 | 0.0 | 21 | 29 | 0 | 21 |
| Path 519 | C00117->C00130:[10->8,12->3,6->17,7->10,7->11,7->12,7->4,7->5,8->13] | 0.90 | 0.0 | 23 | 41 | 0 | 23 |
| Path 520 | C00117->C00130:[10->8,12->3,6->17,7->12,8->13] | 0.50 | 0.0 | 8 | 9 | 0 | 8 |
| Path 521 | C00117->C00130:[10->8,12->3,6->17,7->12,8->11,8->13] | 0.60 | 0.0 | 27 | 34 | 0 | 27 |
| Path 522 | C00117->C00130:[10->8,12->3,6->17,7->10,7->12,7->4,8->13] | 0.70 | 0.0 | 18 | 34 | 0 | 18 |
| Path 523 | C00117->C00130:[10->8,12->3,6->17,7->10,7->12,7->4,8->13] | 0.70 | 0.0 | 15 | 22 | 0 | 15 |
| Path 524 | C00117->C00130:[10->8,12->3,6->17,7->10,7->11,7->12,7->2,7->5,8->13] | 0.90 | 0.0 | 30 | 37 | 0 | 30 |
| Path 525 | C00117->C00130:[10->8,12->3,6->17,7->12,8->11,8->13] | 0.60 | 0.0 | 17 | 20 | 0 | 17 |
| Path 526 | C00117->C00130:[10->8,12->3,6->17,7->12,8->11,8->13] | 0.60 | 0.0 | 17 | 23 | 0 | 17 |
| Path 527 | C00117->C00130:[10->8,12->3,6->17,7->10,7->11,7->12,7->4,7->5,8->13] | 0.90 | 0.0 | 23 | 43 | 0 | 23 |
| Path 528 | C00117->C00130:[10->8,12->3,6->17,7->12,8->11,8->13] | 0.60 | 0.0 | 16 | 22 | 0 | 16 |
| Path 529 | C00117->C00130:[10->12,12->17,6->3,7->8,8->11,8->13] | 0.60 | 0.0 | 15 | 26 | 0 | 15 |
| Path 530 | C00117->C00130:[10->8,12->3,6->17,7->10,7->11,7->12,7->4,7->5,8->13] | 0.90 | 0.0 | 22 | 28 | 0 | 22 |
| Path 531 | C00117->C00130:[10->8,12->3,6->17,7->10,7->11,7->12,8->13] | 0.70 | 0.0 | 16 | 20 | 0 | 16 |
| Path 532 | C00117->C00130:[10->8,12->3,6->17,7->10,7->11,7->12,8->13] | 0.70 | 0.0 | 17 | 23 | 0 | 17 |
| Path 533 | C00117->C00130:[10->8,12->3,6->17,7->10,7->11,7->12,7->4,7->5,8->13] | 0.90 | 0.0 | 21 | 28 | 0 | 21 |
| Path 534 | C00117->C00130:[8->10,8->11] | 0.20 | 0.0 | 23 | 26 | 0 | 23 |
| Path 535 | C00117->C00130:[10->8,12->3,6->17,7->10,7->11,7->12,7->4,7->5,8->13] | 0.90 | 0.0 | 26 | 37 | 0 | 26 |
| Path 536 | C00117->C00130:[10->8,12->3,6->11,6->17,7->12,8->13] | 0.60 | 0.0 | 15 | 16 | 0 | 15 |
| Path 537 | C00117->C00130:[10->8,12->3,6->17,7->12,8->10,8->13,8->4] | 0.70 | 0.0 | 17 | 22 | 0 | 17 |
| Path 538 | C00117->C00130:[10->8,12->3,6->17,7->10,7->11,7->12,8->13] | 0.70 | 0.0 | 20 | 26 | 0 | 20 |
| Path 539 | C00117->C00130:[10->8,12->3,6->17,7->12,8->11,8->13] | 0.60 | 0.0 | 21 | 26 | 0 | 21 |
| Path 540 | C00117->C00130:[10->8,12->3,6->17,7->10,7->11,7->12,7->2,7->5,8->13] | 0.90 | 0.0 | 26 | 33 | 0 | 26 |
| Path 541 | C00117->C00130:[10->8,12->3,6->17,7->10,7->11,7->12,7->4,8->10,8->13] | 0.80 | 0.0 | 31 | 36 | 0 | 31 |
| Path 542 | C00117->C00130:[10->8,12->3,6->11,6->17,7->12,8->13] | 0.60 | 0.0 | 16 | 17 | 0 | 16 |
| Path 543 | C00117->C00130:[10->8,12->3,6->17,7->12,8->11,8->13] | 0.60 | 0.0 | 25 | 34 | 0 | 25 |
| Path 544 | C00117->C00130:[10->8,12->3,6->17,7->12,8->10,8->11,8->13,8->4] | 0.80 | 0.0 | 26 | 33 | 0 | 26 |
| Path 545 | C00117->C00130:[10->8,12->3,6->17,7->12,8->11,8->13] | 0.60 | 0.0 | 16 | 26 | 0 | 16 |
| Path 546 | C00117->C00130:[10->8,12->3,6->17,7->10,7->12,8->13] | 0.60 | 0.0 | 16 | 33 | 0 | 16 |
| Path 547 | C00117->C00130:[10->8,12->3,6->17,7->12,8->11,8->13] | 0.60 | 0.0 | 25 | 35 | 0 | 25 |
| Path 548 | C00117->C00130:[10->8,12->3,6->17,7->10,7->11,7->12,8->13] | 0.70 | 0.0 | 15 | 30 | 0 | 15 |
| Path 549 | C00117->C00130:[10->8,12->3,6->17,7->10,7->11,7->12,8->13] | 0.70 | 0.0 | 15 | 29 | 0 | 15 |
| Path 550 | C00117->C00130:[10->8,12->3,6->17,7->12,8->11,8->13] | 0.60 | 0.0 | 16 | 20 | 0 | 16 |
| Path 551 | C00117->C00130:[10->12,12->17,6->3,7->8,8->11,8->13] | 0.60 | 0.0 | 17 | 28 | 0 | 17 |
| Path 552 | C00117->C00130:[10->8,12->3,6->17,7->12,8->10,8->11,8->13] | 0.70 | 0.0 | 14 | 16 | 0 | 14 |
| Path 553 | C00117->C00130:[10->8,12->3,6->11,6->17,7->12,8->13] | 0.60 | 0.0 | 16 | 19 | 0 | 16 |
| Path 554 | C00117->C00130:[10->8,12->3,6->17,7->10,7->11,7->12,7->2,8->13] | 0.80 | 0.0 | 26 | 37 | 0 | 26 |
| Path 555 | C00117->C00130:[10->8,12->3,6->17,7->12,8->11,8->13] | 0.60 | 0.0 | 18 | 29 | 0 | 18 |
| Path 556 | C00117->C00130:[10->8,12->3,6->17,7->12,8->11,8->13] | 0.60 | 0.0 | 29 | 34 | 0 | 29 |
| Path 557 | C00117->C00130:[10->8,12->3,6->17,7->12,8->11,8->13] | 0.60 | 0.0 | 18 | 25 | 0 | 18 |
| Path 558 | C00117->C00130:[10->8,12->3,6->17,7->12,8->11,8->13] | 0.60 | 0.0 | 14 | 16 | 0 | 14 |
| Path 559 | C00117->C00130:[8->11] | 0.10 | 0.0 | 12 | 27 | 0 | 12 |
| Path 560 | C00117->C00130:[10->8,12->3,6->17,7->12,8->11,8->13] | 0.60 | 0.0 | 25 | 31 | 0 | 25 |
| Path 561 | C00117->C00130:[10->8,12->3,6->17,7->12,8->11,8->13] | 0.60 | 0.0 | 16 | 18 | 0 | 16 |
| Path 562 | C00117->C00130:[10->8,12->3,6->17,7->12,8->10,8->13] | 0.60 | 0.0 | 17 | 22 | 0 | 17 |
| Path 563 | C00117->C00130:[10->8,12->3,6->17,7->10,7->11,7->12,7->4,8->13] | 0.80 | 0.0 | 31 | 43 | 0 | 31 |
| Path 564 | C00117->C00130:[10->8,12->3,6->10,6->11,6->17,6->4,7->12,8->10,8->11,8->13,8->4] | 0.80 | 0.0 | 29 | 38 | 0 | 29 |
| Path 565 | C00117->C00130:[10->8,12->3,6->17,7->10,7->11,7->12,7->4,8->13] | 0.80 | 0.0 | 31 | 38 | 0 | 31 |
| Path 566 | C00117->C00130:[10->8,12->3,6->17,7->10,7->11,7->12,7->2,7->5,8->13] | 0.90 | 0.0 | 26 | 33 | 0 | 26 |
| Path 567 | C00117->C00130:[10->8,12->3,6->17,7->10,7->11,7->12,8->13] | 0.70 | 0.0 | 14 | 17 | 0 | 14 |
| Path 568 | C00117->C00130:[10->8,12->3,6->17,7->12,8->11,8->13] | 0.60 | 0.0 | 19 | 30 | 0 | 19 |
| Path 569 | C00117->C00130:[10->8,12->3,6->17,7->10,7->11,7->12,8->13] | 0.70 | 0.0 | 13 | 16 | 0 | 13 |
