# Supplementary material for: Inferring branching pathways in genome-scale metabolic networks
Source: BMC Syst Biol. 2009 Oct 29;3:103. doi: 10.1186/1752-0509-3-103 (PMC2791103; doi:10.1186/1752-0509-3-103)
Supplement: Additional file 2 — ReTrace results from experiments. Summary data and html output from ReTrace runs performed for the queries discussed in the section Results. A self-contained web site: unpack archive and open index.html in a web browser. [file 1752-0509-3-103-S2.zip › retrace-AF2/imp.html]

ReTrace results: glucose - IMP


## ReTrace results: glucose - IMP

This document contains ReTrace result files from glucose - IMP query and
additional related queries.

Back to index.

| Source(s) | Target | Pathways | Summary | NumPathways | Zo | BestZo | BestAvgScore | AvgScores | #RPAIRs | #Reactions | MinZeroScores | ZeroScores | MinScoresUnderThr | ScoresUnderThr |
| --- | --- | --- | --- | --- | --- | --- | --- | --- | --- | --- | --- | --- | --- | --- |
| D-Glucose | IMP | html | text | 1173 | 0.70 (0.19) | 1.0 | 0.0 | 0.00 (0.00) | 23.26 (6.09) | 41.06 (12.17) | 0 | 0.00 (0.00) | 8 | 23.26 (6.09) |
| D-Ribose 5-phosphate | IMP | html | text | 568 | 0.66 (0.17) | 0.9 | 0.0 | 0.00 (0.00) | 19.91 (5.74) | 27.95 (8.57) | 0 | 0.00 (0.00) | 5 | 19.91 (5.74) |
| D-Glucose | ATP | html | text | 1563 | 0.67 (0.17) | 1.0 | 0.0 | 0.00 (0.00) | 23.56 (6.45) | 147.94 (89.80) | 0 | 0.00 (0.00) | 8 | 23.56 (6.45) |
| D-Ribose 5-phosphate | ATP | html | text | 1543 | 0.72 (0.14) | 1.0 | 0.0 | 0.00 (0.00) | 20.41 (5.39) | 130.15 (118.10) | 0 | 0.00 (0.00) | 3 | 20.41 (5.39) |
