# Supplementary material for: Inferring branching pathways in genome-scale metabolic networks
Source: BMC Syst Biol. 2009 Oct 29;3:103. doi: 10.1186/1752-0509-3-103 (PMC2791103; doi:10.1186/1752-0509-3-103)
Supplement: Additional file 2 — ReTrace results from experiments. Summary data and html output from ReTrace runs performed for the queries discussed in the section Results. A self-contained web site: unpack archive and open index.html in a web browser. [file 1752-0509-3-103-S2.zip › retrace-AF2/pathways-C00031-to-C00002.html]

Pathways from C00031 to C00002


**Pathways from C00031 to C00002**

**Sources:** D-Glucose; (C00031)

**Target:**ATP; (C00002)

|  | Composite mapping | Z | Average score | Rpairs | Reactions | Zero scores | Scores under threshold |
| --- | --- | --- | --- | --- | --- | --- | --- |
| Path 1 | C00031->C00002:[1->12,2->13,4->10,4->11,4->17,4->4,5->8,7->10,7->11,7->4,7->5,9->2,9->3] | 1.00 | 0.0 | 33 | 164 | 0 | 33 |
| Path 2 | C00031->C00002:[1->12,2->13,4->10,4->11,4->17,4->4,5->8,7->5,9->2,9->3] | 1.00 | 0.0 | 30 | 157 | 0 | 30 |
| Path 3 | C00031->C00002:[1->12,2->13,4->10,4->11,4->17,4->4,5->8,7->10,7->11,7->4,7->5,9->2,9->3] | 1.00 | 0.0 | 34 | 169 | 0 | 34 |
| Path 4 | C00031->C00002:[1->12,2->13,4->10,4->11,4->17,4->4,5->8,7->10,7->11,7->4,7->5,9->2,9->3] | 1.00 | 0.0 | 34 | 169 | 0 | 34 |
| Path 5 | C00031->C00002:[1->12,2->13,4->10,4->11,4->17,4->2,4->4,5->8,7->5,9->2,9->3] | 1.00 | 0.0 | 31 | 165 | 0 | 31 |
| Path 6 | C00031->C00002:[1->12,2->13,4->10,4->11,4->17,4->4,5->8,7->5,9->2,9->3] | 1.00 | 0.0 | 30 | 161 | 0 | 30 |
| Path 7 | C00031->C00002:[1->12,2->13,4->10,4->11,4->17,4->2,4->4,5->8,7->5,9->2,9->3] | 1.00 | 0.0 | 29 | 156 | 0 | 29 |
| Path 8 | C00031->C00002:[1->12,2->13,4->10,4->11,4->17,4->4,5->8,7->5,9->2,9->3] | 1.00 | 0.0 | 28 | 152 | 0 | 28 |
| Path 9 | C00031->C00002:[1->12,2->13,4->10,4->11,4->17,4->2,4->4,5->8,7->5,9->2,9->3] | 1.00 | 0.0 | 31 | 162 | 0 | 31 |
| Path 10 | C00031->C00002:[1->12,2->13,4->10,4->11,4->17,4->2,4->4,5->8,7->5,9->2,9->3] | 1.00 | 0.0 | 31 | 165 | 0 | 31 |
| Path 11 | C00031->C00002:[1->12,2->13,4->10,4->11,4->17,4->4,5->8,7->5,9->2,9->3] | 1.00 | 0.0 | 32 | 164 | 0 | 32 |
| Path 12 | C00031->C00002:[1->12,2->13,4->10,4->11,4->17,4->4,5->8,7->5,9->2,9->3] | 1.00 | 0.0 | 32 | 166 | 0 | 32 |
| Path 13 | C00031->C00002:[1->12,2->13,4->10,4->11,4->17,4->4,5->8,7->10,7->11,7->4,7->5,9->2,9->3] | 1.00 | 0.0 | 31 | 159 | 0 | 31 |
| Path 14 | C00031->C00002:[1->12,2->13,4->10,4->11,4->17,4->4,5->8,7->5,9->2,9->3] | 1.00 | 0.0 | 32 | 161 | 0 | 32 |
| Path 15 | C00031->C00002:[1->12,2->13,4->10,4->11,4->17,4->4,5->8,7->5,9->2,9->3] | 1.00 | 0.0 | 32 | 164 | 0 | 32 |
| Path 16 | C00031->C00002:[1->12,2->13,4->10,4->11,4->17,4->4,5->8,7->10,7->11,7->4,7->5,9->2,9->3] | 1.00 | 0.0 | 32 | 160 | 0 | 32 |
| Path 17 | C00031->C00002:[1->12,2->13,4->10,4->11,4->17,4->4,5->8,7->5,9->2,9->3] | 1.00 | 0.0 | 32 | 163 | 0 | 32 |
| Path 18 | C00031->C00002:[1->12,2->13,4->10,4->11,4->17,4->4,5->8,7->5,9->2,9->3] | 1.00 | 0.0 | 30 | 158 | 0 | 30 |
| Path 19 | C00031->C00002:[1->12,2->13,4->10,4->11,4->17,4->4,5->8,7->5,9->2,9->3] | 1.00 | 0.0 | 30 | 161 | 0 | 30 |
| Path 20 | C00031->C00002:[1->12,2->13,4->10,4->11,4->17,4->4,5->8,7->5,9->2,9->3] | 1.00 | 0.0 | 32 | 166 | 0 | 32 |
| Path 21 | C00031->C00002:[1->12,2->13,4->10,4->11,4->17,4->4,5->8,7->10,7->11,7->4,7->5,9->2,9->3] | 1.00 | 0.0 | 31 | 162 | 0 | 31 |
| Path 22 | C00031->C00002:[1->12,2->13,4->10,4->11,4->17,4->4,5->8,7->5,9->2,9->3] | 1.00 | 0.0 | 30 | 155 | 0 | 30 |
| Path 23 | C00031->C00002:[1->12,2->13,4->10,4->11,4->17,4->4,5->8,7->10,7->11,7->4,7->5,9->2,9->3] | 1.00 | 0.0 | 32 | 163 | 0 | 32 |
| Path 24 | C00031->C00002:[2->8,5->13,5->3,7->17,9->12] | 0.50 | 0.0 | 13 | 132 | 0 | 13 |
| Path 25 | C00031->C00002:[4->11,5->13,7->17,7->3,9->12,9->8] | 0.60 | 0.0 | 23 | 152 | 0 | 23 |
| Path 26 | C00031->C00002:[5->13,7->17,7->3,9->12,9->8] | 0.50 | 0.0 | 14 | 36 | 0 | 14 |
| Path 27 | C00031->C00002:[4->10,4->11,5->17,7->13,7->3,7->5,9->12,9->2,9->8] | 0.90 | 0.0 | 34 | 182 | 0 | 34 |
| Path 28 | C00031->C00002:[4->10,4->11,5->13,7->17,7->3,7->5,9->12,9->2,9->8] | 0.90 | 0.0 | 31 | 61 | 0 | 31 |
| Path 29 | C00031->C00002:[4->11,5->13,7->17,7->3,9->12,9->8] | 0.60 | 0.0 | 24 | 376 | 0 | 24 |
| Path 30 | C00031->C00002:[4->11,5->17,7->13,7->3,9->12,9->8] | 0.60 | 0.0 | 26 | 161 | 0 | 26 |
| Path 31 | C00031->C00002:[1->12,2->13,4->10,4->11,4->17,7->3,9->8] | 0.70 | 0.0 | 23 | 152 | 0 | 23 |
| Path 32 | C00031->C00002:[1->12,2->13,4->17,5->3,5->8,7->11] | 0.60 | 0.0 | 19 | 138 | 0 | 19 |
| Path 33 | C00031->C00002:[4->10,4->11,5->13,7->17,7->3,9->12,9->8] | 0.70 | 0.0 | 26 | 56 | 0 | 26 |
| Path 34 | C00031->C00002:[1->12,2->13,4->11,4->17,7->3,9->8] | 0.60 | 0.0 | 22 | 148 | 0 | 22 |
| Path 35 | C00031->C00002:[1->12,2->13,4->17,7->3,9->8] | 0.50 | 0.0 | 12 | 32 | 0 | 12 |
| Path 36 | C00031->C00002:[4->10,4->11,5->13,7->17,7->3,9->12,9->8] | 0.70 | 0.0 | 26 | 49 | 0 | 26 |
| Path 37 | C00031->C00002:[1->12,2->13,4->17,5->8,7->11,9->3] | 0.60 | 0.0 | 17 | 141 | 0 | 17 |
| Path 38 | C00031->C00002:[5->13,7->17,7->3,9->12,9->8] | 0.50 | 0.0 | 16 | 353 | 0 | 16 |
| Path 39 | C00031->C00002:[4->10,4->11,5->13,7->17,7->3,7->5,9->12,9->2,9->8] | 0.90 | 0.0 | 34 | 172 | 0 | 34 |
| Path 40 | C00031->C00002:[2->8,5->13,5->3,7->17,9->12] | 0.50 | 0.0 | 13 | 131 | 0 | 13 |
| Path 41 | C00031->C00002:[4->10,4->11,5->13,7->17,7->3,7->5,9->12,9->2,9->8] | 0.90 | 0.0 | 32 | 50 | 0 | 32 |
| Path 42 | C00031->C00002:[4->11,5->13,7->17,7->3,9->12,9->8] | 0.60 | 0.0 | 22 | 150 | 0 | 22 |
| Path 43 | C00031->C00002:[1->12,2->13,4->11,4->17,5->3,5->8] | 0.60 | 0.0 | 24 | 153 | 0 | 24 |
| Path 44 | C00031->C00002:[1->12,2->13,4->11,4->17,7->3,9->8] | 0.60 | 0.0 | 21 | 35 | 0 | 21 |
| Path 45 | C00031->C00002:[1->12,2->13,4->10,4->11,4->17,5->3,5->8] | 0.70 | 0.0 | 26 | 155 | 0 | 26 |
| Path 46 | C00031->C00002:[1->12,2->13,4->11,4->17,5->3,5->8] | 0.60 | 0.0 | 21 | 369 | 0 | 21 |
| Path 47 | C00031->C00002:[4->10,4->11,5->13,7->17,7->3,9->12,9->8] | 0.70 | 0.0 | 27 | 379 | 0 | 27 |
| Path 48 | C00031->C00002:[4->11,5->17,7->13,7->3,9->12,9->8] | 0.60 | 0.0 | 27 | 389 | 0 | 27 |
| Path 49 | C00031->C00002:[4->10,4->11,5->17,7->13,7->3,7->5,9->12,9->2,9->8] | 0.90 | 0.0 | 32 | 58 | 0 | 32 |
| Path 50 | C00031->C00002:[4->10,4->11,5->13,7->17,7->3,7->5,9->12,9->2,9->8] | 0.90 | 0.0 | 31 | 62 | 0 | 31 |
| Path 51 | C00031->C00002:[1->12,2->13,4->10,4->11,4->17,5->3,5->8,7->10,7->11,7->5,9->2] | 0.90 | 0.0 | 33 | 163 | 0 | 33 |
| Path 52 | C00031->C00002:[4->10,4->11,5->13,7->17,7->3,7->5,9->12,9->2,9->8] | 0.90 | 0.0 | 31 | 380 | 0 | 31 |
| Path 53 | C00031->C00002:[1->12,2->13,4->10,4->11,4->17,7->3,7->5,9->2,9->8] | 0.90 | 0.0 | 30 | 166 | 0 | 30 |
| Path 54 | C00031->C00002:[1->12,2->13,4->10,4->11,4->17,5->8,7->10,7->11,7->5,9->2,9->3] | 0.90 | 0.0 | 28 | 156 | 0 | 28 |
| Path 55 | C00031->C00002:[4->10,4->11,5->13,7->17,7->3,7->5,9->12,9->2,9->8] | 0.90 | 0.0 | 30 | 162 | 0 | 30 |
| Path 56 | C00031->C00002:[1->12,2->13,4->17,5->8,7->11,9->3] | 0.60 | 0.0 | 18 | 142 | 0 | 18 |
| Path 57 | C00031->C00002:[1->12,2->13,4->10,4->17,4->4,5->8,9->3] | 0.70 | 0.0 | 24 | 152 | 0 | 24 |
| Path 58 | C00031->C00002:[4->10,4->11,5->13,7->17,7->3,7->5,9->12,9->2,9->8] | 0.90 | 0.0 | 34 | 165 | 0 | 34 |
| Path 59 | C00031->C00002:[4->11,5->13,7->17,7->3,9->12,9->8] | 0.60 | 0.0 | 23 | 151 | 0 | 23 |
| Path 60 | C00031->C00002:[4->10,4->11,5->13,7->17,7->3,9->12,9->8] | 0.70 | 0.0 | 26 | 68 | 0 | 26 |
| Path 61 | C00031->C00002:[5->13,7->17,7->3,9->12,9->8] | 0.50 | 0.0 | 15 | 359 | 0 | 15 |
| Path 62 | C00031->C00002:[1->12,2->13,4->11,4->17,5->3,5->8] | 0.60 | 0.0 | 24 | 60 | 0 | 24 |
| Path 63 | C00031->C00002:[4->11,5->17,7->13,7->3,9->12,9->8] | 0.60 | 0.0 | 25 | 156 | 0 | 25 |
| Path 64 | C00031->C00002:[1->12,2->13,2->3,4->10,4->11,4->17,5->8] | 0.70 | 0.0 | 25 | 159 | 0 | 25 |
| Path 65 | C00031->C00002:[4->10,4->11,5->17,7->13,7->3,9->12,9->8] | 0.70 | 0.0 | 28 | 50 | 0 | 28 |
| Path 66 | C00031->C00002:[1->12,2->13,4->17,7->3,9->8] | 0.50 | 0.0 | 11 | 129 | 0 | 11 |
| Path 67 | C00031->C00002:[4->11,5->13,7->17,7->3,9->12,9->8] | 0.60 | 0.0 | 24 | 372 | 0 | 24 |
| Path 68 | C00031->C00002:[1->12,2->13,4->17,5->3,5->8] | 0.50 | 0.0 | 15 | 43 | 0 | 15 |
| Path 69 | C00031->C00002:[5->13,7->17,7->3,9->12,9->8] | 0.50 | 0.0 | 15 | 150 | 0 | 15 |
| Path 70 | C00031->C00002:[5->13,7->17,7->3,9->12,9->8] | 0.50 | 0.0 | 15 | 143 | 0 | 15 |
| Path 71 | C00031->C00002:[4->11,5->13,7->17,7->3,9->12,9->8] | 0.60 | 0.0 | 23 | 151 | 0 | 23 |
| Path 72 | C00031->C00002:[1->12,2->13,4->10,4->11,4->17,7->3,7->5,9->2,9->8] | 0.90 | 0.0 | 30 | 166 | 0 | 30 |
| Path 73 | C00031->C00002:[5->13,7->17,7->3,9->12,9->8] | 0.50 | 0.0 | 16 | 136 | 0 | 16 |
| Path 74 | C00031->C00002:[4->11,5->13,7->17,7->3,9->12,9->8] | 0.60 | 0.0 | 24 | 151 | 0 | 24 |
| Path 75 | C00031->C00002:[5->17,7->13,7->3,9->12,9->8] | 0.50 | 0.0 | 16 | 358 | 0 | 16 |
| Path 76 | C00031->C00002:[5->13,7->17,7->3,9->12,9->8] | 0.50 | 0.0 | 14 | 358 | 0 | 14 |
| Path 77 | C00031->C00002:[4->10,4->11,5->13,7->17,7->3,9->12,9->8] | 0.70 | 0.0 | 25 | 161 | 0 | 25 |
| Path 78 | C00031->C00002:[4->11,5->17,7->13,7->3,9->12,9->8] | 0.60 | 0.0 | 25 | 62 | 0 | 25 |
| Path 79 | C00031->C00002:[1->12,2->13,4->17,7->11,7->3,9->8] | 0.60 | 0.0 | 22 | 151 | 0 | 22 |
| Path 80 | C00031->C00002:[4->10,4->11,5->13,7->17,7->3,9->12,9->8] | 0.70 | 0.0 | 26 | 154 | 0 | 26 |
| Path 81 | C00031->C00002:[1->12,2->13,4->10,4->11,4->17,7->3,9->8] | 0.70 | 0.0 | 23 | 152 | 0 | 23 |
| Path 82 | C00031->C00002:[1->12,2->13,4->11,4->17,5->3,5->8] | 0.60 | 0.0 | 24 | 366 | 0 | 24 |
| Path 83 | C00031->C00002:[1->12,2->13,4->11,4->17,7->3,9->8] | 0.60 | 0.0 | 21 | 42 | 0 | 21 |
| Path 84 | C00031->C00002:[1->12,2->13,4->10,4->11,4->17,5->3,5->8] | 0.70 | 0.0 | 27 | 381 | 0 | 27 |
| Path 85 | C00031->C00002:[5->13,7->17,7->3,9->12,9->8] | 0.50 | 0.0 | 18 | 142 | 0 | 18 |
| Path 86 | C00031->C00002:[4->10,4->11,5->13,7->17,7->3,9->12,9->8] | 0.70 | 0.0 | 27 | 159 | 0 | 27 |
| Path 87 | C00031->C00002:[1->12,2->13,4->10,4->11,4->17,5->8,7->5,9->2,9->3] | 0.90 | 0.0 | 27 | 155 | 0 | 27 |
| Path 88 | C00031->C00002:[4->11,5->13,7->17,7->3,9->12,9->8] | 0.60 | 0.0 | 24 | 376 | 0 | 24 |
| Path 89 | C00031->C00002:[1->12,2->13,2->3,4->10,4->11,4->17,5->8] | 0.70 | 0.0 | 24 | 158 | 0 | 24 |
| Path 90 | C00031->C00002:[4->11,5->13,7->17,7->3,9->12,9->8] | 0.60 | 0.0 | 23 | 165 | 0 | 23 |
| Path 91 | C00031->C00002:[1->12,2->13,4->17,5->3,5->8,7->11] | 0.60 | 0.0 | 19 | 139 | 0 | 19 |
| Path 92 | C00031->C00002:[4->11,5->17,7->13,7->3,9->12,9->8] | 0.60 | 0.0 | 26 | 168 | 0 | 26 |
| Path 93 | C00031->C00002:[4->10,4->11,5->13,7->17,7->3,9->12,9->8] | 0.70 | 0.0 | 26 | 162 | 0 | 26 |
| Path 94 | C00031->C00002:[1->12,2->13,4->10,4->11,4->17,5->3,5->8,7->5,9->2] | 0.90 | 0.0 | 31 | 378 | 0 | 31 |
| Path 95 | C00031->C00002:[4->10,4->11,5->13,7->17,7->3,9->12,9->8] | 0.70 | 0.0 | 23 | 43 | 0 | 23 |
| Path 96 | C00031->C00002:[1->12,2->13,4->10,4->11,4->17,5->3,5->8,7->5,9->2] | 0.90 | 0.0 | 31 | 160 | 0 | 31 |
| Path 97 | C00031->C00002:[2->8,5->13,5->3,7->17,9->12] | 0.50 | 0.0 | 12 | 30 | 0 | 12 |
| Path 98 | C00031->C00002:[1->12,2->13,4->10,4->11,4->17,7->3,7->5,9->2,9->8] | 0.90 | 0.0 | 32 | 381 | 0 | 32 |
| Path 99 | C00031->C00002:[4->10,4->11,5->17,7->13,7->3,7->5,9->12,9->2,9->8] | 0.90 | 0.0 | 33 | 167 | 0 | 33 |
| Path 100 | C00031->C00002:[4->10,4->11,5->13,7->17,7->3,7->5,9->12,9->2,9->8] | 0.90 | 0.0 | 32 | 171 | 0 | 32 |
| Path 101 | C00031->C00002:[1->12,2->13,4->17,7->3,9->8] | 0.50 | 0.0 | 12 | 32 | 0 | 12 |
| Path 102 | C00031->C00002:[4->11,5->13,7->17,7->3,9->12,9->8] | 0.60 | 0.0 | 23 | 154 | 0 | 23 |
| Path 103 | C00031->C00002:[5->13,7->17,7->3,9->12,9->8] | 0.50 | 0.0 | 14 | 36 | 0 | 14 |
| Path 104 | C00031->C00002:[4->11,5->17,7->13,7->3,9->12,9->8] | 0.60 | 0.0 | 25 | 163 | 0 | 25 |
| Path 105 | C00031->C00002:[4->10,4->11,5->13,7->17,7->3,9->12,9->8] | 0.70 | 0.0 | 26 | 56 | 0 | 26 |
| Path 106 | C00031->C00002:[5->13,7->17,7->3,9->12,9->8] | 0.50 | 0.0 | 13 | 136 | 0 | 13 |
| Path 107 | C00031->C00002:[1->12,2->13,4->10,4->11,4->17,7->3,9->8] | 0.70 | 0.0 | 25 | 154 | 0 | 25 |
| Path 108 | C00031->C00002:[4->10,4->11,5->13,7->17,7->3,7->5,9->12,9->2,9->8] | 0.90 | 0.0 | 29 | 161 | 0 | 29 |
| Path 109 | C00031->C00002:[4->11,5->17,7->13,7->3,9->12,9->8] | 0.60 | 0.0 | 25 | 55 | 0 | 25 |
| Path 110 | C00031->C00002:[5->17,7->13,7->3,9->12,9->8] | 0.50 | 0.0 | 16 | 139 | 0 | 16 |
| Path 111 | C00031->C00002:[4->10,4->11,5->13,7->17,7->3,9->12,9->8] | 0.70 | 0.0 | 26 | 56 | 0 | 26 |
| Path 112 | C00031->C00002:[1->12,2->13,4->11,4->17,5->8] | 0.50 | 0.0 | 18 | 145 | 0 | 18 |
| Path 113 | C00031->C00002:[2->8,4->11,5->13,5->3,7->17,9->12] | 0.60 | 0.0 | 23 | 149 | 0 | 23 |
| Path 114 | C00031->C00002:[4->10,4->11,5->17,7->13,7->3,9->12,9->8] | 0.70 | 0.0 | 26 | 171 | 0 | 26 |
| Path 115 | C00031->C00002:[7->3,9->8] | 0.20 | 0.0 | 10 | 30 | 0 | 10 |
| Path 116 | C00031->C00002:[4->11,5->13,7->17,7->3,9->12,9->8] | 0.60 | 0.0 | 22 | 154 | 0 | 22 |
| Path 117 | C00031->C00002:[1->12,2->13,4->10,4->11,4->17,5->8,9->3] | 0.70 | 0.0 | 21 | 147 | 0 | 21 |
| Path 118 | C00031->C00002:[4->10,4->11,5->13,7->17,7->3,9->12,9->8] | 0.70 | 0.0 | 24 | 152 | 0 | 24 |
| Path 119 | C00031->C00002:[4->10,4->11,5->13,7->17,7->3,7->5,9->12,9->2,9->8] | 0.90 | 0.0 | 32 | 61 | 0 | 32 |
| Path 120 | C00031->C00002:[4->10,4->11,5->13,7->17,7->3,9->12,9->8] | 0.70 | 0.0 | 26 | 161 | 0 | 26 |
| Path 121 | C00031->C00002:[7->11,7->3,9->8] | 0.30 | 0.0 | 18 | 140 | 0 | 18 |
| Path 122 | C00031->C00002:[1->12,2->13,4->17,7->3,9->8] | 0.50 | 0.0 | 11 | 129 | 0 | 11 |
| Path 123 | C00031->C00002:[4->10,4->11,5->13,7->17,7->3,9->12,9->8] | 0.70 | 0.0 | 23 | 51 | 0 | 23 |
| Path 124 | C00031->C00002:[4->10,4->11,5->17,7->13,7->3,9->12,9->8] | 0.70 | 0.0 | 28 | 75 | 0 | 28 |
| Path 125 | C00031->C00002:[4->10,4->11,5->13,7->17,7->3,9->12,9->8] | 0.70 | 0.0 | 24 | 44 | 0 | 24 |
| Path 126 | C00031->C00002:[1->12,2->13,4->11,4->17,7->3,9->8] | 0.60 | 0.0 | 20 | 363 | 0 | 20 |
| Path 127 | C00031->C00002:[4->10,4->11,5->13,7->17,7->3,7->5,9->12,9->2,9->8] | 0.90 | 0.0 | 31 | 170 | 0 | 31 |
| Path 128 | C00031->C00002:[7->3,9->8] | 0.20 | 0.0 | 9 | 130 | 0 | 9 |
| Path 129 | C00031->C00002:[2->8,5->13,5->3,7->17,9->12] | 0.50 | 0.0 | 12 | 131 | 0 | 12 |
| Path 130 | C00031->C00002:[5->13,7->17,7->3,9->12,9->8] | 0.50 | 0.0 | 15 | 41 | 0 | 15 |
| Path 131 | C00031->C00002:[4->11,5->13,7->17,7->3,9->12,9->8] | 0.60 | 0.0 | 25 | 377 | 0 | 25 |
| Path 132 | C00031->C00002:[5->17,7->13,7->3,9->12,9->8] | 0.50 | 0.0 | 18 | 155 | 0 | 18 |
| Path 133 | C00031->C00002:[4->10,4->11,5->13,7->17,7->3,9->12,9->8] | 0.70 | 0.0 | 24 | 153 | 0 | 24 |
| Path 134 | C00031->C00002:[4->10,4->11,5->17,7->13,7->3,9->12,9->8] | 0.70 | 0.0 | 28 | 173 | 0 | 28 |
| Path 135 | C00031->C00002:[4->11,5->13,7->17,7->3,9->12,9->8] | 0.60 | 0.0 | 23 | 39 | 0 | 23 |
| Path 136 | C00031->C00002:[4->10,4->11,5->13,7->17,7->3,9->12,9->8] | 0.70 | 0.0 | 25 | 153 | 0 | 25 |
| Path 137 | C00031->C00002:[4->10,4->11,5->13,7->17,7->3,7->5,9->12,9->2,9->8] | 0.90 | 0.0 | 30 | 162 | 0 | 30 |
| Path 138 | C00031->C00002:[1->12,2->13,4->17,5->3,5->8,7->11] | 0.60 | 0.0 | 21 | 144 | 0 | 21 |
| Path 139 | C00031->C00002:[4->10,4->11,5->13,7->17,7->3,7->5,9->12,9->2,9->8] | 0.90 | 0.0 | 30 | 50 | 0 | 30 |
| Path 140 | C00031->C00002:[1->12,2->13,4->17,5->3,5->8] | 0.50 | 0.0 | 14 | 30 | 0 | 14 |
| Path 141 | C00031->C00002:[1->12,2->13,4->17,5->3,5->8] | 0.50 | 0.0 | 16 | 362 | 0 | 16 |
| Path 142 | C00031->C00002:[5->17,7->13,7->3,9->12,9->8] | 0.50 | 0.0 | 16 | 144 | 0 | 16 |
| Path 143 | C00031->C00002:[7->3,9->8] | 0.20 | 0.0 | 10 | 30 | 0 | 10 |
| Path 144 | C00031->C00002:[5->13,7->17,7->3,9->12,9->8] | 0.50 | 0.0 | 15 | 139 | 0 | 15 |
| Path 145 | C00031->C00002:[4->10,4->11,5->13,7->17,7->3,9->12,9->8] | 0.70 | 0.0 | 27 | 50 | 0 | 27 |
| Path 146 | C00031->C00002:[1->12,2->13,4->17,7->3,9->8] | 0.50 | 0.0 | 13 | 351 | 0 | 13 |
| Path 147 | C00031->C00002:[5->13,7->17,7->3,9->12,9->8] | 0.50 | 0.0 | 14 | 30 | 0 | 14 |
| Path 148 | C00031->C00002:[4->10,4->11,5->13,7->17,7->3,7->5,9->12,9->2,9->8] | 0.90 | 0.0 | 32 | 163 | 0 | 32 |
| Path 149 | C00031->C00002:[5->13,7->17,7->3,9->12,9->8] | 0.50 | 0.0 | 15 | 138 | 0 | 15 |
| Path 150 | C00031->C00002:[1->12,2->13,4->10,4->11,4->17,7->3,9->8] | 0.70 | 0.0 | 23 | 152 | 0 | 23 |
| Path 151 | C00031->C00002:[5->13,7->17,7->3,9->12,9->8] | 0.50 | 0.0 | 16 | 31 | 0 | 16 |
| Path 152 | C00031->C00002:[1->12,2->13,4->10,4->11,4->17,7->3,7->5,9->2,9->8] | 0.90 | 0.0 | 31 | 163 | 0 | 31 |
| Path 153 | C00031->C00002:[5->13,7->17,7->3,9->12,9->8] | 0.50 | 0.0 | 13 | 144 | 0 | 13 |
| Path 154 | C00031->C00002:[5->13,7->17,7->3,9->12,9->8] | 0.50 | 0.0 | 14 | 30 | 0 | 14 |
| Path 155 | C00031->C00002:[1->12,2->13,4->10,4->11,4->17,5->8,9->3] | 0.70 | 0.0 | 21 | 149 | 0 | 21 |
| Path 156 | C00031->C00002:[1->12,2->13,4->17,7->3,9->8] | 0.50 | 0.0 | 14 | 135 | 0 | 14 |
| Path 157 | C00031->C00002:[4->10,4->11,5->13,7->17,7->3,9->12,9->8] | 0.70 | 0.0 | 26 | 161 | 0 | 26 |
| Path 158 | C00031->C00002:[4->11,5->13,7->17,7->3,9->12,9->8] | 0.60 | 0.0 | 24 | 55 | 0 | 24 |
| Path 159 | C00031->C00002:[4->10,4->11,5->13,7->17,7->3,7->5,9->12,9->2,9->8] | 0.90 | 0.0 | 31 | 62 | 0 | 31 |
| Path 160 | C00031->C00002:[5->13,5->3,5->8,7->17,9->12] | 0.50 | 0.0 | 17 | 359 | 0 | 17 |
| Path 161 | C00031->C00002:[4->11,5->17,7->13,7->3,9->12,9->8] | 0.60 | 0.0 | 25 | 160 | 0 | 25 |
| Path 162 | C00031->C00002:[4->10,4->11,5->13,7->17,7->3,9->12,9->8] | 0.70 | 0.0 | 25 | 156 | 0 | 25 |
| Path 163 | C00031->C00002:[4->10,4->11,5->13,7->17,7->3,7->5,9->12,9->2,9->8] | 0.90 | 0.0 | 34 | 386 | 0 | 34 |
| Path 164 | C00031->C00002:[5->17,7->13,7->3,9->12,9->8] | 0.50 | 0.0 | 16 | 45 | 0 | 16 |
| Path 165 | C00031->C00002:[1->12,2->13,4->10,4->17,5->8,9->3] | 0.60 | 0.0 | 19 | 149 | 0 | 19 |
| Path 166 | C00031->C00002:[1->12,1->8,2->13,4->10,4->11,4->17,4->3,7->3,9->8] | 0.70 | 0.0 | 25 | 154 | 0 | 25 |
| Path 167 | C00031->C00002:[4->10,4->11,5->13,7->17,7->3,7->5,9->12,9->2,9->8] | 0.90 | 0.0 | 33 | 169 | 0 | 33 |
| Path 168 | C00031->C00002:[4->10,4->11,5->13,7->17,7->3,7->5,9->12,9->2,9->8] | 0.90 | 0.0 | 30 | 54 | 0 | 30 |
| Path 169 | C00031->C00002:[5->13,7->17,7->3,9->12,9->8] | 0.50 | 0.0 | 16 | 360 | 0 | 16 |
| Path 170 | C00031->C00002:[4->10,4->11,5->13,7->17,7->3,7->5,9->12,9->2,9->8] | 0.90 | 0.0 | 33 | 51 | 0 | 33 |
| Path 171 | C00031->C00002:[4->11,5->13,7->17,7->3,9->12,9->8] | 0.60 | 0.0 | 23 | 158 | 0 | 23 |
| Path 172 | C00031->C00002:[1->12,2->13,4->10,4->11,4->17,5->3,5->8] | 0.70 | 0.0 | 24 | 148 | 0 | 24 |
| Path 173 | C00031->C00002:[1->12,2->13,4->10,4->11,4->17,5->3,5->8] | 0.70 | 0.0 | 23 | 151 | 0 | 23 |
| Path 174 | C00031->C00002:[1->12,2->13,4->10,4->11,4->17,5->3,5->8] | 0.70 | 0.0 | 27 | 159 | 0 | 27 |
| Path 175 | C00031->C00002:[1->12,2->13,4->10,4->11,4->17,5->3,5->8,7->10,7->11,7->5,9->2] | 0.90 | 0.0 | 31 | 161 | 0 | 31 |
| Path 176 | C00031->C00002:[2->8,4->10,4->11,5->13,5->3,7->17,9->12] | 0.70 | 0.0 | 25 | 369 | 0 | 25 |
| Path 177 | C00031->C00002:[1->12,2->13,4->11,4->17,5->8] | 0.50 | 0.0 | 18 | 145 | 0 | 18 |
| Path 178 | C00031->C00002:[4->11,5->17,7->13,7->3,9->12,9->8] | 0.60 | 0.0 | 24 | 160 | 0 | 24 |
| Path 179 | C00031->C00002:[1->12,2->13,4->10,4->17,5->8,9->3] | 0.60 | 0.0 | 19 | 146 | 0 | 19 |
| Path 180 | C00031->C00002:[4->11,5->13,7->17,7->3,9->12,9->8] | 0.60 | 0.0 | 23 | 61 | 0 | 23 |
| Path 181 | C00031->C00002:[4->10,4->11,5->13,7->17,7->3,7->5,9->12,9->2,9->8] | 0.90 | 0.0 | 32 | 77 | 0 | 32 |
| Path 182 | C00031->C00002:[1->12,2->13,4->11,4->17,7->3,9->8] | 0.60 | 0.0 | 20 | 146 | 0 | 20 |
| Path 183 | C00031->C00002:[5->13,7->17,7->3,9->12,9->8] | 0.50 | 0.0 | 23 | 150 | 0 | 23 |
| Path 184 | C00031->C00002:[5->3,7->11] | 0.20 | 0.0 | 18 | 137 | 0 | 18 |
| Path 185 | C00031->C00002:[1->12,2->13,2->3,4->11,4->17,5->8] | 0.60 | 0.0 | 22 | 151 | 0 | 22 |
| Path 186 | C00031->C00002:[1->12,2->13,2->3,4->10,4->11,4->17,5->8,7->5,9->2] | 0.90 | 0.0 | 30 | 160 | 0 | 30 |
| Path 187 | C00031->C00002:[1->12,2->13,4->10,4->11,4->17,4->4,5->8,7->10,7->11,7->2,7->4,9->3,9->5] | 1.00 | 0.0 | 32 | 172 | 0 | 32 |
| Path 188 | C00031->C00002:[4->10,4->11,5->17,7->13,7->3,9->12,9->8] | 0.70 | 0.0 | 28 | 176 | 0 | 28 |
| Path 189 | C00031->C00002:[1->12,2->13,4->10,4->11,4->17,5->8,9->3] | 0.70 | 0.0 | 21 | 150 | 0 | 21 |
| Path 190 | C00031->C00002:[1->12,2->13,4->11,4->17,5->3,5->8] | 0.60 | 0.0 | 21 | 361 | 0 | 21 |
| Path 191 | C00031->C00002:[5->13,7->17,7->3,9->12,9->8] | 0.50 | 0.0 | 15 | 140 | 0 | 15 |
| Path 192 | C00031->C00002:[4->11,5->13,7->17,7->3,9->12,9->8] | 0.60 | 0.0 | 23 | 155 | 0 | 23 |
| Path 193 | C00031->C00002:[5->13,7->17,7->3,9->12,9->8] | 0.50 | 0.0 | 13 | 137 | 0 | 13 |
| Path 194 | C00031->C00002:[1->12,2->13,4->10,4->11,4->17,4->4,5->8,9->3] | 0.80 | 0.0 | 27 | 160 | 0 | 27 |
| Path 195 | C00031->C00002:[5->13,7->17,7->3,9->12,9->8] | 0.50 | 0.0 | 12 | 139 | 0 | 12 |
| Path 196 | C00031->C00002:[4->10,4->11,5->13,7->17,7->3,9->12,9->8] | 0.70 | 0.0 | 26 | 157 | 0 | 26 |
| Path 197 | C00031->C00002:[5->13,7->17,7->3,9->12,9->8] | 0.50 | 0.0 | 22 | 149 | 0 | 22 |
| Path 198 | C00031->C00002:[1->12,2->13,4->10,4->11,4->17,5->3,5->8,7->5,9->2] | 0.90 | 0.0 | 31 | 161 | 0 | 31 |
| Path 199 | C00031->C00002:[1->12,2->13,4->10,4->11,4->17,4->4,5->8,9->3] | 0.80 | 0.0 | 28 | 158 | 0 | 28 |
| Path 200 | C00031->C00002:[2->8,5->13,5->3,7->17,9->12] | 0.50 | 0.0 | 15 | 350 | 0 | 15 |
| Path 201 | C00031->C00002:[4->11,5->13,5->3,5->8,7->17,9->12] | 0.60 | 0.0 | 24 | 56 | 0 | 24 |
| Path 202 | C00031->C00002:[4->11,5->17,7->13,7->3,9->12,9->8] | 0.60 | 0.0 | 24 | 160 | 0 | 24 |
| Path 203 | C00031->C00002:[1->12,2->13,4->17,5->8,7->11,9->3] | 0.60 | 0.0 | 18 | 143 | 0 | 18 |
| Path 204 | C00031->C00002:[1->12,2->13,4->10,4->11,4->17,7->3,7->5,9->2,9->8] | 0.90 | 0.0 | 28 | 56 | 0 | 28 |
| Path 205 | C00031->C00002:[5->13,7->17,7->3,9->12,9->8] | 0.50 | 0.0 | 22 | 153 | 0 | 22 |
| Path 206 | C00031->C00002:[4->10,4->11,5->13,7->17,7->3,7->5,9->12,9->2,9->8] | 0.90 | 0.0 | 31 | 162 | 0 | 31 |
| Path 207 | C00031->C00002:[5->13,7->17,7->3,9->12,9->8] | 0.50 | 0.0 | 16 | 41 | 0 | 16 |
| Path 208 | C00031->C00002:[1->12,2->13,4->10,4->11,4->17,5->3,5->8,7->5,9->2] | 0.90 | 0.0 | 30 | 59 | 0 | 30 |
| Path 209 | C00031->C00002:[1->12,2->13,4->11,4->17,7->3,9->8] | 0.60 | 0.0 | 21 | 154 | 0 | 21 |
| Path 210 | C00031->C00002:[4->10,4->11,5->17,7->13,7->3,7->5,9->12,9->2,9->8] | 0.90 | 0.0 | 35 | 176 | 0 | 35 |
| Path 211 | C00031->C00002:[5->13,7->17,7->3,9->12,9->8] | 0.50 | 0.0 | 15 | 40 | 0 | 15 |
| Path 212 | C00031->C00002:[5->13,7->17,7->3,9->12,9->8] | 0.50 | 0.0 | 19 | 145 | 0 | 19 |
| Path 213 | C00031->C00002:[4->11,5->17,7->13,7->3,9->12,9->8] | 0.60 | 0.0 | 25 | 375 | 0 | 25 |
| Path 214 | C00031->C00002:[4->10,4->11,5->17,7->13,7->3,7->5,9->12,9->2,9->8] | 0.90 | 0.0 | 35 | 400 | 0 | 35 |
| Path 215 | C00031->C00002:[5->13,7->17,7->3,9->12,9->8] | 0.50 | 0.0 | 13 | 133 | 0 | 13 |
| Path 216 | C00031->C00002:[1->12,2->13,4->11,4->17,5->3,5->8] | 0.60 | 0.0 | 18 | 141 | 0 | 18 |
| Path 217 | C00031->C00002:[4->10,4->11,5->13,7->17,7->3,9->12,9->8] | 0.70 | 0.0 | 25 | 56 | 0 | 25 |
| Path 218 | C00031->C00002:[4->10,4->11,5->17,7->13,7->3,7->5,9->12,9->2,9->8] | 0.90 | 0.0 | 35 | 171 | 0 | 35 |
| Path 219 | C00031->C00002:[1->12,2->13,4->10,4->11,4->17,4->4,5->8,7->10,7->11,7->2,7->4,9->3,9->5] | 1.00 | 0.0 | 32 | 175 | 0 | 32 |
| Path 220 | C00031->C00002:[5->13,7->17,7->3,9->12,9->8] | 0.50 | 0.0 | 15 | 143 | 0 | 15 |
| Path 221 | C00031->C00002:[1->12,2->13,4->10,4->11,4->17,5->3,5->8,7->5,9->2] | 0.90 | 0.0 | 31 | 159 | 0 | 31 |
| Path 222 | C00031->C00002:[5->13,7->17,7->3,9->12,9->8] | 0.50 | 0.0 | 15 | 37 | 0 | 15 |
| Path 223 | C00031->C00002:[4->11,5->13,7->17,7->3,9->12,9->8] | 0.60 | 0.0 | 24 | 46 | 0 | 24 |
| Path 224 | C00031->C00002:[1->12,2->13,4->10,4->11,4->17,5->3,5->8] | 0.70 | 0.0 | 21 | 144 | 0 | 21 |
| Path 225 | C00031->C00002:[1->12,2->13,4->11,4->17,5->3,5->8] | 0.60 | 0.0 | 24 | 60 | 0 | 24 |
| Path 226 | C00031->C00002:[1->12,2->13,4->17,5->3,5->8] | 0.50 | 0.0 | 16 | 140 | 0 | 16 |
| Path 227 | C00031->C00002:[5->17,7->13,7->3,9->12,9->8] | 0.50 | 0.0 | 17 | 144 | 0 | 17 |
| Path 228 | C00031->C00002:[1->12,2->13,4->17,7->3,9->8] | 0.50 | 0.0 | 12 | 133 | 0 | 12 |
| Path 229 | C00031->C00002:[4->10,4->11,5->13,7->17,7->3,9->12,9->8] | 0.70 | 0.0 | 23 | 57 | 0 | 23 |
| Path 230 | C00031->C00002:[5->3,7->11] | 0.20 | 0.0 | 19 | 141 | 0 | 19 |
| Path 231 | C00031->C00002:[1->12,2->13,4->17,7->3,9->8] | 0.50 | 0.0 | 12 | 133 | 0 | 12 |
| Path 232 | C00031->C00002:[5->13,7->17,7->3,9->12,9->8] | 0.50 | 0.0 | 14 | 33 | 0 | 14 |
| Path 233 | C00031->C00002:[1->12,2->13,2->3,4->10,4->11,4->17,5->8,7->5,9->2] | 0.90 | 0.0 | 31 | 168 | 0 | 31 |
| Path 234 | C00031->C00002:[4->10,4->11,5->17,7->13,7->3,7->5,9->12,9->2,9->8] | 0.90 | 0.0 | 35 | 169 | 0 | 35 |
| Path 235 | C00031->C00002:[1->12,2->13,4->11,4->17,5->3,5->8] | 0.60 | 0.0 | 22 | 147 | 0 | 22 |
| Path 236 | C00031->C00002:[1->12,2->13,4->10,4->11,4->17,5->3,5->8,7->5,9->2] | 0.90 | 0.0 | 35 | 170 | 0 | 35 |
| Path 237 | C00031->C00002:[4->11,5->17,7->13,7->3,9->12,9->8] | 0.60 | 0.0 | 25 | 159 | 0 | 25 |
| Path 238 | C00031->C00002:[1->12,2->13,4->10,4->11,4->17,5->3,5->8] | 0.70 | 0.0 | 26 | 376 | 0 | 26 |
| Path 239 | C00031->C00002:[5->13,7->17,7->3,9->12,9->8] | 0.50 | 0.0 | 13 | 40 | 0 | 13 |
| Path 240 | C00031->C00002:[4->10,4->11,5->17,7->13,7->3,7->5,9->12,9->2,9->8] | 0.90 | 0.0 | 35 | 173 | 0 | 35 |
| Path 241 | C00031->C00002:[4->10,4->11,5->17,7->13,7->3,7->5,9->12,9->2,9->8] | 0.90 | 0.0 | 34 | 70 | 0 | 34 |
| Path 242 | C00031->C00002:[4->11,5->17,7->13,7->3,9->12,9->8] | 0.60 | 0.0 | 24 | 162 | 0 | 24 |
| Path 243 | C00031->C00002:[5->13,7->17,7->3,9->12,9->8] | 0.50 | 0.0 | 14 | 149 | 0 | 14 |
| Path 244 | C00031->C00002:[1->12,2->13,4->10,4->11,4->17,5->3,5->8,7->10,7->11] | 0.70 | 0.0 | 25 | 156 | 0 | 25 |
| Path 245 | C00031->C00002:[4->10,4->11,5->13,7->17,7->3,7->5,9->12,9->2,9->8] | 0.90 | 0.0 | 34 | 165 | 0 | 34 |
| Path 246 | C00031->C00002:[5->17,7->13,7->3,9->12,9->8] | 0.50 | 0.0 | 15 | 143 | 0 | 15 |
| Path 247 | C00031->C00002:[1->12,2->13,4->17,5->3,5->8,7->11] | 0.60 | 0.0 | 21 | 148 | 0 | 21 |
| Path 248 | C00031->C00002:[4->10,4->11,5->13,7->17,7->3,9->12,9->8] | 0.70 | 0.0 | 26 | 172 | 0 | 26 |
| Path 249 | C00031->C00002:[4->10,4->11,5->17,7->13,7->3,7->5,9->12,9->2,9->8] | 0.90 | 0.0 | 37 | 185 | 0 | 37 |
| Path 250 | C00031->C00002:[1->12,2->13,4->10,4->11,4->17,5->3,5->8] | 0.70 | 0.0 | 25 | 154 | 0 | 25 |
| Path 251 | C00031->C00002:[1->12,2->13,4->17,5->3,5->8] | 0.50 | 0.0 | 13 | 348 | 0 | 13 |
| Path 252 | C00031->C00002:[5->13,7->17,7->3,9->12,9->8] | 0.50 | 0.0 | 15 | 134 | 0 | 15 |
| Path 253 | C00031->C00002:[4->11,5->13,7->17,7->3,9->12,9->8] | 0.60 | 0.0 | 23 | 156 | 0 | 23 |
| Path 254 | C00031->C00002:[4->11,5->13,5->3,5->8,7->17,9->12] | 0.60 | 0.0 | 26 | 376 | 0 | 26 |
| Path 255 | C00031->C00002:[4->11,5->13,7->17,7->3,9->12,9->8] | 0.60 | 0.0 | 25 | 160 | 0 | 25 |
| Path 256 | C00031->C00002:[1->12,2->13,4->10,4->11,4->17,5->3,5->8,7->5,9->2] | 0.90 | 0.0 | 31 | 59 | 0 | 31 |
| Path 257 | C00031->C00002:[1->12,2->13,4->10,4->11,4->17,5->3,5->8] | 0.70 | 0.0 | 20 | 143 | 0 | 20 |
| Path 258 | C00031->C00002:[4->10,4->11,5->17,7->13,7->3,9->12,9->8] | 0.70 | 0.0 | 26 | 171 | 0 | 26 |
| Path 259 | C00031->C00002:[5->13,7->17,7->3,9->12,9->8] | 0.50 | 0.0 | 13 | 36 | 0 | 13 |
| Path 260 | C00031->C00002:[4->10,4->11,5->13,7->17,7->3,9->12,9->8] | 0.70 | 0.0 | 26 | 45 | 0 | 26 |
| Path 261 | C00031->C00002:[4->11,5->13,7->17,7->3,9->12,9->8] | 0.60 | 0.0 | 25 | 160 | 0 | 25 |
| Path 262 | C00031->C00002:[4->10,4->11,5->13,7->17,7->3,7->5,9->12,9->2,9->8] | 0.90 | 0.0 | 34 | 176 | 0 | 34 |
| Path 263 | C00031->C00002:[5->13,7->17,7->3,9->12,9->8] | 0.50 | 0.0 | 16 | 42 | 0 | 16 |
| Path 264 | C00031->C00002:[1->12,2->13,4->10,4->11,4->17,5->8,9->3] | 0.70 | 0.0 | 24 | 158 | 0 | 24 |
| Path 265 | C00031->C00002:[1->12,2->13,4->17,5->3,5->8] | 0.50 | 0.0 | 13 | 131 | 0 | 13 |
| Path 266 | C00031->C00002:[4->10,4->11,5->17,7->13,7->3,9->12,9->8] | 0.70 | 0.0 | 27 | 158 | 0 | 27 |
| Path 267 | C00031->C00002:[1->12,2->13,4->17,5->3,5->8] | 0.50 | 0.0 | 11 | 126 | 0 | 11 |
| Path 268 | C00031->C00002:[1->12,2->13,4->10,4->11,4->17,7->3,7->5,9->2,9->8] | 0.90 | 0.0 | 29 | 57 | 0 | 29 |
| Path 269 | C00031->C00002:[4->10,4->11,5->17,7->13,7->3,7->5,9->12,9->2,9->8] | 0.90 | 0.0 | 34 | 179 | 0 | 34 |
| Path 270 | C00031->C00002:[4->10,4->11,5->13,7->17,7->3,9->12,9->8] | 0.70 | 0.0 | 26 | 158 | 0 | 26 |
| Path 271 | C00031->C00002:[4->10,4->11,5->13,7->17,7->3,7->5,9->12,9->2,9->8] | 0.90 | 0.0 | 32 | 59 | 0 | 32 |
| Path 272 | C00031->C00002:[1->12,2->13,4->17,5->3,5->8] | 0.50 | 0.0 | 11 | 347 | 0 | 11 |
| Path 273 | C00031->C00002:[4->10,4->11,5->17,7->13,7->3,9->12,9->8] | 0.70 | 0.0 | 29 | 174 | 0 | 29 |
| Path 274 | C00031->C00002:[5->17,7->13,7->3,9->12,9->8] | 0.50 | 0.0 | 17 | 144 | 0 | 17 |
| Path 275 | C00031->C00002:[1->12,2->13,4->11,4->17,5->3,5->8] | 0.60 | 0.0 | 25 | 375 | 0 | 25 |
| Path 276 | C00031->C00002:[1->12,2->13,4->10,4->11,4->17,5->3,5->8,7->10,7->11] | 0.70 | 0.0 | 26 | 156 | 0 | 26 |
| Path 277 | C00031->C00002:[4->10,4->11,5->13,7->17,7->3,7->5,9->12,9->2,9->8] | 0.90 | 0.0 | 30 | 162 | 0 | 30 |
| Path 278 | C00031->C00002:[1->12,2->13,4->17,7->3,9->8] | 0.50 | 0.0 | 13 | 134 | 0 | 13 |
| Path 279 | C00031->C00002:[4->10,4->11,5->13,7->17,7->3,7->5,9->12,9->2,9->8] | 0.90 | 0.0 | 32 | 73 | 0 | 32 |
| Path 280 | C00031->C00002:[4->10,4->11,5->17,7->13,7->3,9->12,9->8] | 0.70 | 0.0 | 26 | 59 | 0 | 26 |
| Path 281 | C00031->C00002:[5->3,7->11] | 0.20 | 0.0 | 19 | 142 | 0 | 19 |
| Path 282 | C00031->C00002:[1->12,2->13,4->10,4->11,4->17,4->2,5->8,7->5,9->2,9->3] | 0.90 | 0.0 | 28 | 159 | 0 | 28 |
| Path 283 | C00031->C00002:[4->10,4->11,5->17,7->13,7->3,9->12,9->8] | 0.70 | 0.0 | 26 | 61 | 0 | 26 |
| Path 284 | C00031->C00002:[4->10,4->11,5->13,7->17,7->3,7->5,9->12,9->2,9->8] | 0.90 | 0.0 | 29 | 164 | 0 | 29 |
| Path 285 | C00031->C00002:[5->13,7->17,7->3,9->12,9->8] | 0.50 | 0.0 | 14 | 29 | 0 | 14 |
| Path 286 | C00031->C00002:[1->12,2->13,4->17,5->8,7->10,9->3] | 0.60 | 0.0 | 19 | 145 | 0 | 19 |
| Path 287 | C00031->C00002:[4->10,4->11,5->13,7->17,7->3,9->12,9->8] | 0.70 | 0.0 | 25 | 59 | 0 | 25 |
| Path 288 | C00031->C00002:[4->10,4->11,5->13,7->17,7->3,7->5,9->12,9->2,9->8] | 0.90 | 0.0 | 33 | 179 | 0 | 33 |
| Path 289 | C00031->C00002:[4->11,5->13,7->17,7->3,9->12,9->8] | 0.60 | 0.0 | 24 | 43 | 0 | 24 |
| Path 290 | C00031->C00002:[4->10,4->11,5->17,7->13,7->3,7->5,9->12,9->2,9->8] | 0.90 | 0.0 | 36 | 184 | 0 | 36 |
| Path 291 | C00031->C00002:[1->12,2->13,4->17,5->3,5->8] | 0.50 | 0.0 | 11 | 25 | 0 | 11 |
| Path 292 | C00031->C00002:[4->10,4->11,5->13,7->17,7->3,7->5,9->12,9->2,9->8] | 0.90 | 0.0 | 31 | 379 | 0 | 31 |
| Path 293 | C00031->C00002:[4->10,4->11,5->13,7->17,7->3,9->12,9->8] | 0.70 | 0.0 | 25 | 153 | 0 | 25 |
| Path 294 | C00031->C00002:[4->10,4->11,5->13,7->17,7->3,7->5,9->12,9->2,9->8] | 0.90 | 0.0 | 35 | 181 | 0 | 35 |
| Path 295 | C00031->C00002:[5->13,7->17,7->3,9->12,9->8] | 0.50 | 0.0 | 15 | 30 | 0 | 15 |
| Path 296 | C00031->C00002:[4->10,4->11,5->13,7->17,7->3,9->12,9->8] | 0.70 | 0.0 | 26 | 169 | 0 | 26 |
| Path 297 | C00031->C00002:[2->8,4->10,4->11,5->13,5->3,7->17,9->12] | 0.70 | 0.0 | 24 | 53 | 0 | 24 |
| Path 298 | C00031->C00002:[1->12,2->13,4->10,4->11,4->17,7->3,7->5,9->2,9->8] | 0.90 | 0.0 | 29 | 46 | 0 | 29 |
| Path 299 | C00031->C00002:[4->10,4->11,5->13,7->17,7->3,9->12,9->8] | 0.70 | 0.0 | 24 | 58 | 0 | 24 |
| Path 300 | C00031->C00002:[4->11,5->13,7->17,7->3,9->12,9->8] | 0.60 | 0.0 | 23 | 47 | 0 | 23 |
| Path 301 | C00031->C00002:[2->8,4->10,4->11,5->13,5->3,7->17,9->12] | 0.70 | 0.0 | 23 | 150 | 0 | 23 |
| Path 302 | C00031->C00002:[4->10,4->11,5->13,7->17,7->3,9->12,9->8] | 0.70 | 0.0 | 26 | 45 | 0 | 26 |
| Path 303 | C00031->C00002:[4->10,4->11,5->13,7->17,7->3,7->5,9->12,9->2,9->8] | 0.90 | 0.0 | 34 | 176 | 0 | 34 |
| Path 304 | C00031->C00002:[1->12,2->13,4->10,4->11,4->17,5->3,5->8,7->5,9->2] | 0.90 | 0.0 | 35 | 388 | 0 | 35 |
| Path 305 | C00031->C00002:[1->12,2->13,4->17,5->3,5->8] | 0.50 | 0.0 | 11 | 134 | 0 | 11 |
| Path 306 | C00031->C00002:[1->12,2->13,2->3,4->10,4->11,4->17,5->8,7->5,9->2] | 0.90 | 0.0 | 32 | 168 | 0 | 32 |
| Path 307 | C00031->C00002:[4->10,4->11,5->13,7->17,7->3,7->5,9->12,9->2,9->8] | 0.90 | 0.0 | 30 | 162 | 0 | 30 |
| Path 308 | C00031->C00002:[4->10,4->11,5->17,7->13,7->3,9->12,9->8] | 0.70 | 0.0 | 26 | 164 | 0 | 26 |
| Path 309 | C00031->C00002:[5->3] | 0.10 | 0.0 | 8 | 22 | 0 | 8 |
| Path 310 | C00031->C00002:[1->12,2->13,4->10,4->11,4->17,7->3,7->5,9->2,9->8] | 0.90 | 0.0 | 30 | 47 | 0 | 30 |
| Path 311 | C00031->C00002:[4->10,4->11,5->17,7->13,7->3,7->5,9->12,9->2,9->8] | 0.90 | 0.0 | 32 | 67 | 0 | 32 |
| Path 312 | C00031->C00002:[4->10,4->11,5->13,7->17,7->3,9->12,9->8] | 0.70 | 0.0 | 25 | 52 | 0 | 25 |
| Path 313 | C00031->C00002:[1->12,2->13,4->17,5->3,5->8] | 0.50 | 0.0 | 11 | 33 | 0 | 11 |
| Path 314 | C00031->C00002:[4->10,4->11,5->13,7->17,7->3,7->5,9->12,9->2,9->8] | 0.90 | 0.0 | 31 | 162 | 0 | 31 |
| Path 315 | C00031->C00002:[1->12,2->13,4->10,4->11,4->17,7->3,7->5,9->2,9->8] | 0.90 | 0.0 | 31 | 160 | 0 | 31 |
| Path 316 | C00031->C00002:[5->17,7->13,7->3,9->12,9->8] | 0.50 | 0.0 | 16 | 153 | 0 | 16 |
| Path 317 | C00031->C00002:[1->12,2->13,4->10,4->17,4->4,5->3,5->8] | 0.70 | 0.0 | 25 | 150 | 0 | 25 |
| Path 318 | C00031->C00002:[4->10,4->11,5->17,7->13,7->3,7->5,9->12,9->2,9->8] | 0.90 | 0.0 | 32 | 69 | 0 | 32 |
| Path 319 | C00031->C00002:[4->11,5->17,7->13,7->3,9->12,9->8] | 0.60 | 0.0 | 26 | 161 | 0 | 26 |
| Path 320 | C00031->C00002:[1->12,2->13,4->10,4->11,4->17,5->3,5->8,7->5,9->2] | 0.90 | 0.0 | 28 | 155 | 0 | 28 |
| Path 321 | C00031->C00002:[1->12,2->13,4->10,4->11,4->17,5->3,5->8] | 0.70 | 0.0 | 22 | 52 | 0 | 22 |
| Path 322 | C00031->C00002:[4->10,4->11,5->13,7->17,7->3,9->12,9->8] | 0.70 | 0.0 | 24 | 155 | 0 | 24 |
| Path 323 | C00031->C00002:[5->13,7->17,7->3,9->12,9->8] | 0.50 | 0.0 | 16 | 363 | 0 | 16 |
| Path 324 | C00031->C00002:[1->12,2->13,4->11,4->17,7->3,9->8] | 0.60 | 0.0 | 22 | 372 | 0 | 22 |
| Path 325 | C00031->C00002:[4->11,5->17,7->13,7->3,9->12,9->8] | 0.60 | 0.0 | 25 | 378 | 0 | 25 |
| Path 326 | C00031->C00002:[4->11,5->13,7->17,7->3,9->12,9->8] | 0.60 | 0.0 | 22 | 64 | 0 | 22 |
| Path 327 | C00031->C00002:[5->17,7->13,7->3,9->12,9->8] | 0.50 | 0.0 | 15 | 152 | 0 | 15 |
| Path 328 | C00031->C00002:[5->13,7->17,7->3,9->12,9->8] | 0.50 | 0.0 | 16 | 143 | 0 | 16 |
| Path 329 | C00031->C00002:[5->13,7->17,7->3,9->12,9->8] | 0.50 | 0.0 | 16 | 138 | 0 | 16 |
| Path 330 | C00031->C00002:[5->3,7->11] | 0.20 | 0.0 | 18 | 137 | 0 | 18 |
| Path 331 | C00031->C00002:[5->13,7->17,7->3,9->12,9->8] | 0.50 | 0.0 | 17 | 138 | 0 | 17 |
| Path 332 | C00031->C00002:[4->10,4->11,5->13,7->17,7->3,9->12,9->8] | 0.70 | 0.0 | 25 | 154 | 0 | 25 |
| Path 333 | C00031->C00002:[4->11,5->13,7->17,7->3,9->12,9->8] | 0.60 | 0.0 | 22 | 149 | 0 | 22 |
| Path 334 | C00031->C00002:[5->17,7->13,7->3,9->12,9->8] | 0.50 | 0.0 | 16 | 148 | 0 | 16 |
| Path 335 | C00031->C00002:[4->11,5->13,7->17,7->3,9->12,9->8] | 0.60 | 0.0 | 23 | 43 | 0 | 23 |
| Path 336 | C00031->C00002:[4->10,4->11,5->13,7->17,7->3,9->12,9->8] | 0.70 | 0.0 | 24 | 55 | 0 | 24 |
| Path 337 | C00031->C00002:[4->11,5->13,7->17,7->3,9->12,9->8] | 0.60 | 0.0 | 22 | 158 | 0 | 22 |
| Path 338 | C00031->C00002:[4->11,5->13,7->17,7->3,9->12,9->8] | 0.60 | 0.0 | 24 | 164 | 0 | 24 |
| Path 339 | C00031->C00002:[5->13,7->17,7->3,9->12,9->8] | 0.50 | 0.0 | 14 | 142 | 0 | 14 |
| Path 340 | C00031->C00002:[5->13,7->17,7->3,9->12,9->8] | 0.50 | 0.0 | 15 | 137 | 0 | 15 |
| Path 341 | C00031->C00002:[1->12,2->13,4->10,4->11,4->17,5->3,5->8,7->5,9->2] | 0.90 | 0.0 | 32 | 161 | 0 | 32 |
| Path 342 | C00031->C00002:[1->12,2->13,4->10,4->11,4->17,4->4,5->3,5->8] | 0.80 | 0.0 | 26 | 152 | 0 | 26 |
| Path 343 | C00031->C00002:[4->10,4->11,5->17,7->13,7->3,9->12,9->8] | 0.70 | 0.0 | 28 | 166 | 0 | 28 |
| Path 344 | C00031->C00002:[1->12,2->13,4->11,4->17,5->3,5->8] | 0.60 | 0.0 | 24 | 60 | 0 | 24 |
| Path 345 | C00031->C00002:[4->11,5->13,7->17,7->3,9->12,9->8] | 0.60 | 0.0 | 22 | 158 | 0 | 22 |
| Path 346 | C00031->C00002:[4->11,5->13,7->17,7->3,9->12,9->8] | 0.60 | 0.0 | 22 | 154 | 0 | 22 |
| Path 347 | C00031->C00002:[4->10,4->11,5->17,7->13,7->3,9->12,9->8] | 0.70 | 0.0 | 27 | 57 | 0 | 27 |
| Path 348 | C00031->C00002:[4->10,4->11,5->17,7->13,7->3,7->5,9->12,9->2,9->8] | 0.90 | 0.0 | 34 | 65 | 0 | 34 |
| Path 349 | C00031->C00002:[4->11,5->13,7->17,7->3,9->12,9->8] | 0.60 | 0.0 | 22 | 368 | 0 | 22 |
| Path 350 | C00031->C00002:[5->13,7->17,7->3,9->12,9->8] | 0.50 | 0.0 | 14 | 138 | 0 | 14 |
| Path 351 | C00031->C00002:[4->10,4->11,5->13,7->17,7->3,9->12,9->8] | 0.70 | 0.0 | 25 | 165 | 0 | 25 |
| Path 352 | C00031->C00002:[1->12,2->13,4->10,4->17,5->8,9->3] | 0.60 | 0.0 | 21 | 153 | 0 | 21 |
| Path 353 | C00031->C00002:[1->12,2->13,4->10,4->11,4->17,5->8,9->3] | 0.70 | 0.0 | 24 | 155 | 0 | 24 |
| Path 354 | C00031->C00002:[4->10,4->11,5->13,7->17,7->3,9->12,9->8] | 0.70 | 0.0 | 25 | 154 | 0 | 25 |
| Path 355 | C00031->C00002:[4->11,5->17,7->13,7->3,9->12,9->8] | 0.60 | 0.0 | 25 | 57 | 0 | 25 |
| Path 356 | C00031->C00002:[5->3,7->11] | 0.20 | 0.0 | 19 | 138 | 0 | 19 |
| Path 357 | C00031->C00002:[4->10,4->11,5->13,7->17,7->3,9->12,9->8] | 0.70 | 0.0 | 25 | 160 | 0 | 25 |
| Path 358 | C00031->C00002:[4->10,4->11,5->13,7->17,7->3,9->12,9->8] | 0.70 | 0.0 | 24 | 159 | 0 | 24 |
| Path 359 | C00031->C00002:[1->12,2->13,4->11,4->17,7->3,9->8] | 0.60 | 0.0 | 22 | 148 | 0 | 22 |
| Path 360 | C00031->C00002:[4->10,4->11,5->17,7->13,7->3,9->12,9->8] | 0.70 | 0.0 | 28 | 176 | 0 | 28 |
| Path 361 | C00031->C00002:[5->13,7->17,7->3,9->12,9->8] | 0.50 | 0.0 | 12 | 42 | 0 | 12 |
| Path 362 | C00031->C00002:[4->10,4->11,5->13,7->17,7->3,9->12,9->8] | 0.70 | 0.0 | 26 | 156 | 0 | 26 |
| Path 363 | C00031->C00002:[1->12,2->13,4->17,7->3,9->8] | 0.50 | 0.0 | 13 | 134 | 0 | 13 |
| Path 364 | C00031->C00002:[4->11,5->13,7->17,7->3,9->12,9->8] | 0.60 | 0.0 | 24 | 155 | 0 | 24 |
| Path 365 | C00031->C00002:[5->13,7->17,7->3,9->12,9->8] | 0.50 | 0.0 | 13 | 133 | 0 | 13 |
| Path 366 | C00031->C00002:[1->12,2->13,4->10,4->11,4->17,5->8,7->10,7->11,9->3] | 0.70 | 0.0 | 22 | 150 | 0 | 22 |
| Path 367 | C00031->C00002:[1->12,2->13,4->10,4->17,4->4,5->8,9->3] | 0.70 | 0.0 | 27 | 163 | 0 | 27 |
| Path 368 | C00031->C00002:[5->13,7->17,7->3,9->12,9->8] | 0.50 | 0.0 | 16 | 38 | 0 | 16 |
| Path 369 | C00031->C00002:[4->11,5->13,7->17,7->3,9->12,9->8] | 0.60 | 0.0 | 25 | 156 | 0 | 25 |
| Path 370 | C00031->C00002:[4->11,5->13,7->17,7->3,9->12,9->8] | 0.60 | 0.0 | 23 | 156 | 0 | 23 |
| Path 371 | C00031->C00002:[5->13,7->17,7->3,9->12,9->8] | 0.50 | 0.0 | 18 | 139 | 0 | 18 |
| Path 372 | C00031->C00002:[5->3,7->11] | 0.20 | 0.0 | 18 | 137 | 0 | 18 |
| Path 373 | C00031->C00002:[5->13,7->17,7->3,9->12,9->8] | 0.50 | 0.0 | 15 | 135 | 0 | 15 |
| Path 374 | C00031->C00002:[5->17,7->13,7->3,9->12,9->8] | 0.50 | 0.0 | 16 | 31 | 0 | 16 |
| Path 375 | C00031->C00002:[1->12,2->13,4->17,5->8,7->10,9->3] | 0.60 | 0.0 | 18 | 141 | 0 | 18 |
| Path 376 | C00031->C00002:[2->8,4->10,4->11,5->13,5->3,7->17,9->12] | 0.70 | 0.0 | 25 | 372 | 0 | 25 |
| Path 377 | C00031->C00002:[4->10,4->11,5->13,7->17,7->3,9->12,9->8] | 0.70 | 0.0 | 24 | 151 | 0 | 24 |
| Path 378 | C00031->C00002:[1->12,2->13,4->10,4->11,4->17,5->8,9->3] | 0.70 | 0.0 | 21 | 148 | 0 | 21 |
| Path 379 | C00031->C00002:[1->12,2->13,4->10,4->11,4->17,4->4,5->8,9->3] | 0.80 | 0.0 | 30 | 167 | 0 | 30 |
| Path 380 | C00031->C00002:[1->12,2->13,4->10,4->17,5->8] | 0.50 | 0.0 | 20 | 149 | 0 | 20 |
| Path 381 | C00031->C00002:[4->10,4->11,5->13,7->17,7->3,7->5,9->12,9->2,9->8] | 0.90 | 0.0 | 31 | 58 | 0 | 31 |
| Path 382 | C00031->C00002:[4->11,5->13,7->17,7->3,9->12,9->8] | 0.60 | 0.0 | 25 | 373 | 0 | 25 |
| Path 383 | C00031->C00002:[5->13,7->17,7->3,9->12,9->8] | 0.50 | 0.0 | 15 | 146 | 0 | 15 |
| Path 384 | C00031->C00002:[1->12,2->13,4->17,5->3,5->8] | 0.50 | 0.0 | 11 | 29 | 0 | 11 |
| Path 385 | C00031->C00002:[1->12,2->13,4->17,5->8,7->11] | 0.50 | 0.0 | 19 | 146 | 0 | 19 |
| Path 386 | C00031->C00002:[1->12,2->13,4->10,4->11,4->17,5->3,5->8] | 0.70 | 0.0 | 26 | 62 | 0 | 26 |
| Path 387 | C00031->C00002:[4->10,4->11,5->13,7->17,7->3,7->5,9->12,9->2,9->8] | 0.90 | 0.0 | 32 | 55 | 0 | 32 |
| Path 388 | C00031->C00002:[1->12,2->13,4->10,4->17,4->4,5->3,5->8] | 0.70 | 0.0 | 24 | 153 | 0 | 24 |
| Path 389 | C00031->C00002:[4->11,5->13,7->17,7->3,9->12,9->8] | 0.60 | 0.0 | 24 | 151 | 0 | 24 |
| Path 390 | C00031->C00002:[4->11,5->13,7->17,7->3,9->12,9->8] | 0.60 | 0.0 | 25 | 159 | 0 | 25 |
| Path 391 | C00031->C00002:[4->10,4->11,5->13,7->17,7->3,9->12,9->8] | 0.70 | 0.0 | 25 | 157 | 0 | 25 |
| Path 392 | C00031->C00002:[4->11,5->13,7->17,7->3,9->12,9->8] | 0.60 | 0.0 | 23 | 151 | 0 | 23 |
| Path 393 | C00031->C00002:[4->11,5->13,7->17,7->3,9->12,9->8] | 0.60 | 0.0 | 24 | 167 | 0 | 24 |
| Path 394 | C00031->C00002:[4->10,4->11,5->13,7->17,7->3,7->5,9->12,9->2,9->8] | 0.90 | 0.0 | 31 | 61 | 0 | 31 |
| Path 395 | C00031->C00002:[1->12,2->13,4->10,4->11,4->17,7->3,7->5,9->2,9->8] | 0.90 | 0.0 | 28 | 157 | 0 | 28 |
| Path 396 | C00031->C00002:[1->12,2->13,4->11,4->17,5->3,5->8] | 0.60 | 0.0 | 20 | 360 | 0 | 20 |
| Path 397 | C00031->C00002:[4->10,4->11,5->13,7->17,7->3,9->12,9->8] | 0.70 | 0.0 | 24 | 151 | 0 | 24 |
| Path 398 | C00031->C00002:[1->12,2->13,4->10,4->11,4->17,5->3,5->8] | 0.70 | 0.0 | 22 | 48 | 0 | 22 |
| Path 399 | C00031->C00002:[5->17,7->13,7->3,9->12,9->8] | 0.50 | 0.0 | 17 | 53 | 0 | 17 |
| Path 400 | C00031->C00002:[4->11,5->13,7->17,7->3,9->12,9->8] | 0.60 | 0.0 | 24 | 380 | 0 | 24 |
| Path 401 | C00031->C00002:[4->10,4->11,5->13,7->17,7->3,7->5,9->12,9->2,9->8] | 0.90 | 0.0 | 33 | 59 | 0 | 33 |
| Path 402 | C00031->C00002:[4->10,4->11,5->17,7->13,7->3,9->12,9->8] | 0.70 | 0.0 | 28 | 170 | 0 | 28 |
| Path 403 | C00031->C00002:[1->12,2->13,4->10,4->11,4->17,7->3,9->8] | 0.70 | 0.0 | 22 | 148 | 0 | 22 |
| Path 404 | C00031->C00002:[1->12,2->13,2->3,4->17,5->8] | 0.50 | 0.0 | 12 | 134 | 0 | 12 |
| Path 405 | C00031->C00002:[4->11,5->13,7->17,7->3,9->12,9->8] | 0.60 | 0.0 | 24 | 53 | 0 | 24 |
| Path 406 | C00031->C00002:[4->10,4->11,5->13,7->17,7->3,7->5,9->12,9->2,9->8] | 0.90 | 0.0 | 30 | 162 | 0 | 30 |
| Path 407 | C00031->C00002:[7->11,7->3,9->8] | 0.30 | 0.0 | 19 | 145 | 0 | 19 |
| Path 408 | C00031->C00002:[4->10,4->11,5->17,7->13,7->3,9->12,9->8] | 0.70 | 0.0 | 27 | 160 | 0 | 27 |
| Path 409 | C00031->C00002:[5->13,7->17,7->3,9->12,9->8] | 0.50 | 0.0 | 13 | 36 | 0 | 13 |
| Path 410 | C00031->C00002:[4->11,5->13,7->17,7->3,9->12,9->8] | 0.60 | 0.0 | 21 | 55 | 0 | 21 |
| Path 411 | C00031->C00002:[5->13,7->17,7->3,9->12,9->8] | 0.50 | 0.0 | 16 | 151 | 0 | 16 |
| Path 412 | C00031->C00002:[5->17,7->13,7->3,9->12,9->8] | 0.50 | 0.0 | 17 | 143 | 0 | 17 |
| Path 413 | C00031->C00002:[4->10,4->11,5->17,7->13,7->3,9->12,9->8] | 0.70 | 0.0 | 26 | 70 | 0 | 26 |
| Path 414 | C00031->C00002:[1->12,2->13,4->10,4->11,4->17,4->4,5->8,9->3] | 0.80 | 0.0 | 25 | 152 | 0 | 25 |
| Path 415 | C00031->C00002:[5->3,7->11] | 0.20 | 0.0 | 19 | 141 | 0 | 19 |
| Path 416 | C00031->C00002:[1->12,2->13,4->11,4->17,5->8,9->3] | 0.60 | 0.0 | 17 | 138 | 0 | 17 |
| Path 417 | C00031->C00002:[5->13,7->17,7->3,9->12,9->8] | 0.50 | 0.0 | 22 | 148 | 0 | 22 |
| Path 418 | C00031->C00002:[5->13,7->17,7->3,9->12,9->8] | 0.50 | 0.0 | 15 | 38 | 0 | 15 |
| Path 419 | C00031->C00002:[1->12,2->13,4->17,5->8,7->11] | 0.50 | 0.0 | 19 | 142 | 0 | 19 |
| Path 420 | C00031->C00002:[5->13,7->17,7->3,9->12,9->8] | 0.50 | 0.0 | 23 | 151 | 0 | 23 |
| Path 421 | C00031->C00002:[4->10,4->11,5->13,7->17,7->3,9->12,9->8] | 0.70 | 0.0 | 26 | 68 | 0 | 26 |
| Path 422 | C00031->C00002:[5->17,7->13,7->3,9->12,9->8] | 0.50 | 0.0 | 17 | 142 | 0 | 17 |
| Path 423 | C00031->C00002:[1->12,2->13,4->11,4->17,5->3,5->8] | 0.60 | 0.0 | 22 | 362 | 0 | 22 |
| Path 424 | C00031->C00002:[5->17,7->13,7->3,9->12,9->8] | 0.50 | 0.0 | 17 | 149 | 0 | 17 |
| Path 425 | C00031->C00002:[1->12,2->13,4->17,5->3,5->8,7->11] | 0.60 | 0.0 | 22 | 145 | 0 | 22 |
| Path 426 | C00031->C00002:[5->13,7->17,7->3,9->12,9->8] | 0.50 | 0.0 | 14 | 141 | 0 | 14 |
| Path 427 | C00031->C00002:[4->11,5->13,7->17,7->3,9->12,9->8] | 0.60 | 0.0 | 22 | 41 | 0 | 22 |
| Path 428 | C00031->C00002:[4->10,4->11,5->13,7->17,7->3,7->5,9->12,9->2,9->8] | 0.90 | 0.0 | 32 | 162 | 0 | 32 |
| Path 429 | C00031->C00002:[4->10,4->11,5->13,7->17,7->3,7->5,9->12,9->2,9->8] | 0.90 | 0.0 | 31 | 50 | 0 | 31 |
| Path 430 | C00031->C00002:[1->12,2->13,4->17,7->3,9->8] | 0.50 | 0.0 | 12 | 130 | 0 | 12 |
| Path 431 | C00031->C00002:[4->10,4->11,5->13,7->17,7->3,7->5,9->12,9->2,9->8] | 0.90 | 0.0 | 32 | 178 | 0 | 32 |
| Path 432 | C00031->C00002:[1->12,2->13,4->17,5->3,5->8] | 0.50 | 0.0 | 16 | 140 | 0 | 16 |
| Path 433 | C00031->C00002:[4->10,4->11,5->17,7->13,7->3,7->5,9->12,9->2,9->8] | 0.90 | 0.0 | 33 | 59 | 0 | 33 |
| Path 434 | C00031->C00002:[1->12,2->13,4->17,7->11,7->3,9->8] | 0.60 | 0.0 | 20 | 142 | 0 | 20 |
| Path 435 | C00031->C00002:[4->10,4->11,5->13,7->17,7->3,9->12,9->8] | 0.70 | 0.0 | 25 | 64 | 0 | 25 |
| Path 436 | C00031->C00002:[4->11,5->13,7->17,7->3,9->12,9->8] | 0.60 | 0.0 | 23 | 47 | 0 | 23 |
| Path 437 | C00031->C00002:[4->10,4->11,5->17,7->13,7->3,7->5,9->12,9->2,9->8] | 0.90 | 0.0 | 34 | 170 | 0 | 34 |
| Path 438 | C00031->C00002:[4->10,4->11,5->13,7->17,7->3,7->5,9->12,9->2,9->8] | 0.90 | 0.0 | 33 | 392 | 0 | 33 |
| Path 439 | C00031->C00002:[4->10,4->11,5->13,7->17,7->3,9->12,9->8] | 0.70 | 0.0 | 24 | 373 | 0 | 24 |
| Path 440 | C00031->C00002:[4->10,4->11,5->13,7->17,7->3,7->5,9->12,9->2,9->8] | 0.90 | 0.0 | 31 | 162 | 0 | 31 |
| Path 441 | C00031->C00002:[5->13,7->17,7->3,9->12,9->8] | 0.50 | 0.0 | 16 | 139 | 0 | 16 |
| Path 442 | C00031->C00002:[4->11,5->17,7->13,7->3,9->12,9->8] | 0.60 | 0.0 | 26 | 385 | 0 | 26 |
| Path 443 | C00031->C00002:[5->13,7->17,7->3,9->12,9->8] | 0.50 | 0.0 | 16 | 151 | 0 | 16 |
| Path 444 | C00031->C00002:[4->10,4->11,5->13,7->17,7->3,7->5,9->12,9->2,9->8] | 0.90 | 0.0 | 32 | 163 | 0 | 32 |
| Path 445 | C00031->C00002:[5->13,7->17,7->3,9->12,9->8] | 0.50 | 0.0 | 13 | 28 | 0 | 13 |
| Path 446 | C00031->C00002:[4->10,4->11,5->13,7->17,7->3,7->5,9->12,9->2,9->8] | 0.90 | 0.0 | 31 | 55 | 0 | 31 |
| Path 447 | C00031->C00002:[5->13,7->17,7->3,9->12,9->8] | 0.50 | 0.0 | 15 | 34 | 0 | 15 |
| Path 448 | C00031->C00002:[4->10,4->11,5->13,7->17,7->3,9->12,9->8] | 0.70 | 0.0 | 26 | 161 | 0 | 26 |
| Path 449 | C00031->C00002:[1->12,2->13,4->17,5->8,7->10,9->3] | 0.60 | 0.0 | 16 | 139 | 0 | 16 |
| Path 450 | C00031->C00002:[4->10,4->11,5->17,7->13,7->3,9->12,9->8] | 0.70 | 0.0 | 27 | 167 | 0 | 27 |
| Path 451 | C00031->C00002:[4->10,4->11,5->17,7->13,7->3,9->12,9->8] | 0.70 | 0.0 | 27 | 53 | 0 | 27 |
| Path 452 | C00031->C00002:[4->10,4->11,5->13,7->17,7->3,7->5,9->12,9->2,9->8] | 0.90 | 0.0 | 32 | 62 | 0 | 32 |
| Path 453 | C00031->C00002:[5->13,7->17,7->3,9->12,9->8] | 0.50 | 0.0 | 16 | 136 | 0 | 16 |
| Path 454 | C00031->C00002:[4->10,4->11,5->17,7->13,7->3,7->5,9->12,9->2,9->8] | 0.90 | 0.0 | 35 | 64 | 0 | 35 |
| Path 455 | C00031->C00002:[4->10,4->11,5->17,7->13,7->3,9->12,9->8] | 0.70 | 0.0 | 28 | 168 | 0 | 28 |
| Path 456 | C00031->C00002:[4->10,4->11,5->13,7->17,7->3,7->5,9->12,9->2,9->8] | 0.90 | 0.0 | 33 | 78 | 0 | 33 |
| Path 457 | C00031->C00002:[4->11,5->13,7->17,7->3,9->12,9->8] | 0.60 | 0.0 | 25 | 157 | 0 | 25 |
| Path 458 | C00031->C00002:[1->12,2->13,4->10,4->11,4->17,7->3,9->8] | 0.70 | 0.0 | 23 | 156 | 0 | 23 |
| Path 459 | C00031->C00002:[4->10,4->11,5->13,7->17,7->3,7->5,9->12,9->2,9->8] | 0.90 | 0.0 | 35 | 390 | 0 | 35 |
| Path 460 | C00031->C00002:[4->10,4->11,5->13,7->17,7->3,9->12,9->8] | 0.70 | 0.0 | 26 | 154 | 0 | 26 |
| Path 461 | C00031->C00002:[5->13,7->17,7->3,9->12,9->8] | 0.50 | 0.0 | 16 | 140 | 0 | 16 |
| Path 462 | C00031->C00002:[5->13,7->17,7->3,9->12,9->8] | 0.50 | 0.0 | 15 | 52 | 0 | 15 |
| Path 463 | C00031->C00002:[4->10,4->11,5->13,7->17,7->3,9->12,9->8] | 0.70 | 0.0 | 26 | 56 | 0 | 26 |
| Path 464 | C00031->C00002:[1->12,2->13,4->10,4->11,4->17,4->4,5->8,7->10,7->11,7->4,9->3] | 0.80 | 0.0 | 28 | 159 | 0 | 28 |
| Path 465 | C00031->C00002:[5->3] | 0.10 | 0.0 | 8 | 22 | 0 | 8 |
| Path 466 | C00031->C00002:[1->12,2->13,4->17,7->3,9->8] | 0.50 | 0.0 | 11 | 31 | 0 | 11 |
| Path 467 | C00031->C00002:[4->11,5->17,7->13,7->3,9->12,9->8] | 0.60 | 0.0 | 25 | 62 | 0 | 25 |
| Path 468 | C00031->C00002:[4->10,4->11,5->13,7->17,7->3,9->12,9->8] | 0.70 | 0.0 | 26 | 378 | 0 | 26 |
| Path 469 | C00031->C00002:[5->13,7->17,7->3,9->12,9->8] | 0.50 | 0.0 | 16 | 30 | 0 | 16 |
| Path 470 | C00031->C00002:[1->12,2->13,4->10,4->11,4->17,4->4,5->8,9->3] | 0.80 | 0.0 | 27 | 159 | 0 | 27 |
| Path 471 | C00031->C00002:[1->12,2->13,4->10,4->11,4->17,5->3,5->8,7->5,9->2] | 0.90 | 0.0 | 33 | 169 | 0 | 33 |
| Path 472 | C00031->C00002:[4->10,4->11,5->17,7->13,7->3,7->5,9->12,9->2,9->8] | 0.90 | 0.0 | 33 | 388 | 0 | 33 |
| Path 473 | C00031->C00002:[4->10,4->11,5->13,7->17,7->3,7->5,9->12,9->2,9->8] | 0.90 | 0.0 | 31 | 163 | 0 | 31 |
| Path 474 | C00031->C00002:[4->10,4->11,5->13,7->17,7->3,7->5,9->12,9->2,9->8] | 0.90 | 0.0 | 32 | 164 | 0 | 32 |
| Path 475 | C00031->C00002:[1->12,2->13,4->10,4->11,4->17,5->3,5->8] | 0.70 | 0.0 | 23 | 371 | 0 | 23 |
| Path 476 | C00031->C00002:[4->10,4->11,5->13,7->17,7->3,7->5,9->12,9->2,9->8] | 0.90 | 0.0 | 33 | 163 | 0 | 33 |
| Path 477 | C00031->C00002:[4->11,5->13,7->17,7->3,9->12,9->8] | 0.60 | 0.0 | 24 | 167 | 0 | 24 |
| Path 478 | C00031->C00002:[1->12,2->13,4->10,4->11,4->17,5->3,5->8] | 0.70 | 0.0 | 22 | 153 | 0 | 22 |
| Path 479 | C00031->C00002:[4->10,4->11,5->13,7->17,7->3,9->12,9->8] | 0.70 | 0.0 | 25 | 158 | 0 | 25 |
| Path 480 | C00031->C00002:[4->11,5->17,7->13,7->3,9->12,9->8] | 0.60 | 0.0 | 27 | 175 | 0 | 27 |
| Path 481 | C00031->C00002:[1->12,2->13,4->10,4->11,4->17,5->3,5->8,7->5,9->2] | 0.90 | 0.0 | 34 | 170 | 0 | 34 |
| Path 482 | C00031->C00002:[4->11,5->17,7->13,7->3,9->12,9->8] | 0.60 | 0.0 | 26 | 59 | 0 | 26 |
| Path 483 | C00031->C00002:[4->11,5->13,7->17,7->3,9->12,9->8] | 0.60 | 0.0 | 23 | 165 | 0 | 23 |
| Path 484 | C00031->C00002:[4->10,4->11,5->17,7->13,7->3,9->12,9->8] | 0.70 | 0.0 | 27 | 165 | 0 | 27 |
| Path 485 | C00031->C00002:[1->12,2->13,4->10,4->11,4->17,4->4,5->8,7->4,7->5,9->2,9->3] | 1.00 | 0.0 | 34 | 168 | 0 | 34 |
| Path 486 | C00031->C00002:[1->12,2->13,4->17,5->3,5->8] | 0.50 | 0.0 | 14 | 135 | 0 | 14 |
| Path 487 | C00031->C00002:[4->10,4->11,5->13,7->17,7->3,7->5,9->12,9->2,9->8] | 0.90 | 0.0 | 29 | 63 | 0 | 29 |
| Path 488 | C00031->C00002:[1->12,2->13,4->11,4->17,5->3,5->8] | 0.60 | 0.0 | 20 | 151 | 0 | 20 |
| Path 489 | C00031->C00002:[1->12,2->13,4->11,4->17,7->3,9->8] | 0.60 | 0.0 | 20 | 37 | 0 | 20 |
| Path 490 | C00031->C00002:[4->10,4->11,5->13,7->17,7->3,9->12,9->8] | 0.70 | 0.0 | 27 | 375 | 0 | 27 |
| Path 491 | C00031->C00002:[4->10,4->11,5->17,7->13,7->3,9->12,9->8] | 0.70 | 0.0 | 26 | 162 | 0 | 26 |
| Path 492 | C00031->C00002:[1->12,2->13,4->17,5->3,5->8] | 0.50 | 0.0 | 13 | 134 | 0 | 13 |
| Path 493 | C00031->C00002:[5->13,7->17,7->3,9->12,9->8] | 0.50 | 0.0 | 14 | 149 | 0 | 14 |
| Path 494 | C00031->C00002:[4->10,4->11,5->13,7->17,7->3,9->12,9->8] | 0.70 | 0.0 | 27 | 390 | 0 | 27 |
| Path 495 | C00031->C00002:[1->12,2->13,4->10,4->11,4->17,5->3,5->8,7->10,7->11] | 0.70 | 0.0 | 24 | 151 | 0 | 24 |
| Path 496 | C00031->C00002:[1->12,2->13,2->3,4->10,4->11,4->17,5->8] | 0.70 | 0.0 | 25 | 158 | 0 | 25 |
| Path 497 | C00031->C00002:[4->10,4->11,5->13,7->17,7->3,7->5,9->12,9->2,9->8] | 0.90 | 0.0 | 33 | 67 | 0 | 33 |
| Path 498 | C00031->C00002:[4->11,5->17,7->13,7->3,9->12,9->8] | 0.60 | 0.0 | 25 | 165 | 0 | 25 |
| Path 499 | C00031->C00002:[1->12,2->13,4->10,4->11,4->17,5->3,5->8,7->5,9->2] | 0.90 | 0.0 | 31 | 158 | 0 | 31 |
| Path 500 | C00031->C00002:[5->13,5->3,7->17,7->3,9->12,9->8] | 0.50 | 0.0 | 18 | 141 | 0 | 18 |
| Path 501 | C00031->C00002:[4->10,4->11,5->13,7->17,7->3,9->12,9->8] | 0.70 | 0.0 | 25 | 161 | 0 | 25 |
| Path 502 | C00031->C00002:[5->3,7->11] | 0.20 | 0.0 | 18 | 140 | 0 | 18 |
| Path 503 | C00031->C00002:[4->10,4->11,5->13,7->17,7->3,9->12,9->8] | 0.70 | 0.0 | 25 | 59 | 0 | 25 |
| Path 504 | C00031->C00002:[1->12,2->13,4->11,4->17,5->3,5->8] | 0.60 | 0.0 | 24 | 161 | 0 | 24 |
| Path 505 | C00031->C00002:[5->17,7->13,7->3,9->12,9->8] | 0.50 | 0.0 | 16 | 43 | 0 | 16 |
| Path 506 | C00031->C00002:[1->12,2->13,4->10,4->17,5->8,9->3] | 0.60 | 0.0 | 19 | 145 | 0 | 19 |
| Path 507 | C00031->C00002:[4->10,4->11,5->13,7->17,7->3,7->5,9->12,9->2,9->8] | 0.90 | 0.0 | 34 | 180 | 0 | 34 |
| Path 508 | C00031->C00002:[4->11,5->13,7->17,7->3,9->12,9->8] | 0.60 | 0.0 | 24 | 159 | 0 | 24 |
| Path 509 | C00031->C00002:[4->11,5->17,7->13,7->3,9->12,9->8] | 0.60 | 0.0 | 26 | 160 | 0 | 26 |
| Path 510 | C00031->C00002:[4->10,4->11,5->13,7->17,7->3,9->12,9->8] | 0.70 | 0.0 | 25 | 156 | 0 | 25 |
| Path 511 | C00031->C00002:[4->10,4->11,5->17,7->13,7->3,7->5,9->12,9->2,9->8] | 0.90 | 0.0 | 34 | 170 | 0 | 34 |
| Path 512 | C00031->C00002:[5->13,7->17,7->3,9->12,9->8] | 0.50 | 0.0 | 14 | 141 | 0 | 14 |
| Path 513 | C00031->C00002:[4->10,4->11,5->13,7->17,7->3,9->12,9->8] | 0.70 | 0.0 | 25 | 45 | 0 | 25 |
| Path 514 | C00031->C00002:[4->10,4->11,5->13,7->17,7->3,7->5,9->12,9->2,9->8] | 0.90 | 0.0 | 32 | 62 | 0 | 32 |
| Path 515 | C00031->C00002:[1->12,2->13,4->10,4->11,4->17,7->3,9->8] | 0.70 | 0.0 | 23 | 369 | 0 | 23 |
| Path 516 | C00031->C00002:[4->10,4->11,5->17,7->13,7->3,9->12,9->8] | 0.70 | 0.0 | 26 | 159 | 0 | 26 |
| Path 517 | C00031->C00002:[1->12,2->13,4->10,4->11,4->17,7->3,7->5,9->2,9->8] | 0.90 | 0.0 | 28 | 56 | 0 | 28 |
| Path 518 | C00031->C00002:[5->13,7->17,7->3,9->12,9->8] | 0.50 | 0.0 | 16 | 31 | 0 | 16 |
| Path 519 | C00031->C00002:[1->12,2->13,4->11,4->17,7->3,9->8] | 0.60 | 0.0 | 23 | 152 | 0 | 23 |
| Path 520 | C00031->C00002:[4->10,4->11,5->17,7->13,7->3,9->12,9->8] | 0.70 | 0.0 | 28 | 385 | 0 | 28 |
| Path 521 | C00031->C00002:[4->10,4->11,5->13,7->17,7->3,9->12,9->8] | 0.70 | 0.0 | 27 | 162 | 0 | 27 |
| Path 522 | C00031->C00002:[5->3,7->11] | 0.20 | 0.0 | 19 | 138 | 0 | 19 |
| Path 523 | C00031->C00002:[5->13,7->17,9->12] | 0.30 | 0.0 | 16 | 137 | 0 | 16 |
| Path 524 | C00031->C00002:[1->12,2->13,4->17,5->8,9->10,9->3] | 0.60 | 0.0 | 20 | 149 | 0 | 20 |
| Path 525 | C00031->C00002:[1->12,2->13,4->11,4->17,7->3,9->8] | 0.60 | 0.0 | 22 | 155 | 0 | 22 |
| Path 526 | C00031->C00002:[4->10,4->11,5->13,7->17,7->3,9->12,9->8] | 0.70 | 0.0 | 27 | 157 | 0 | 27 |
| Path 527 | C00031->C00002:[2->8,4->11,5->13,5->3,7->17,9->12] | 0.60 | 0.0 | 23 | 48 | 0 | 23 |
| Path 528 | C00031->C00002:[5->17,7->13,7->3,9->12,9->8] | 0.50 | 0.0 | 15 | 51 | 0 | 15 |
| Path 529 | C00031->C00002:[1->12,2->13,4->10,4->11,4->17,7->3,7->5,9->2,9->8] | 0.90 | 0.0 | 29 | 57 | 0 | 29 |
| Path 530 | C00031->C00002:[4->10,4->11,5->13,7->17,7->3,9->12,9->8] | 0.70 | 0.0 | 27 | 383 | 0 | 27 |
| Path 531 | C00031->C00002:[1->12,2->13,4->10,4->11,4->17,5->3,5->8,7->5,9->2] | 0.90 | 0.0 | 32 | 61 | 0 | 32 |
| Path 532 | C00031->C00002:[1->12,2->13,4->10,4->11,4->17,7->3,9->8] | 0.70 | 0.0 | 23 | 149 | 0 | 23 |
| Path 533 | C00031->C00002:[7->3,9->8] | 0.20 | 0.0 | 9 | 127 | 0 | 9 |
| Path 534 | C00031->C00002:[4->10,4->11,5->13,7->17,7->3,9->12,9->8] | 0.70 | 0.0 | 26 | 154 | 0 | 26 |
| Path 535 | C00031->C00002:[5->17,7->13,7->3,9->12,9->8] | 0.50 | 0.0 | 16 | 153 | 0 | 16 |
| Path 536 | C00031->C00002:[1->12,2->13,4->11,4->17,7->3,9->8] | 0.60 | 0.0 | 22 | 50 | 0 | 22 |
| Path 537 | C00031->C00002:[4->11,5->13,7->17,7->3,9->12,9->8] | 0.60 | 0.0 | 24 | 54 | 0 | 24 |
| Path 538 | C00031->C00002:[1->12,2->13,4->10,4->11,4->17,5->3,5->8,7->5,9->2] | 0.90 | 0.0 | 29 | 155 | 0 | 29 |
| Path 539 | C00031->C00002:[4->10,4->11,5->13,7->17,7->3,7->5,9->12,9->2,9->8] | 0.90 | 0.0 | 34 | 381 | 0 | 34 |
| Path 540 | C00031->C00002:[5->17,7->13,7->3,9->12,9->8] | 0.50 | 0.0 | 18 | 155 | 0 | 18 |
| Path 541 | C00031->C00002:[4->11,5->13,5->3,5->8,7->17,9->12] | 0.60 | 0.0 | 25 | 57 | 0 | 25 |
| Path 542 | C00031->C00002:[5->13,7->17,7->3,9->12,9->8] | 0.50 | 0.0 | 23 | 149 | 0 | 23 |
| Path 543 | C00031->C00002:[1->12,2->13,4->17,7->3,9->8] | 0.50 | 0.0 | 11 | 129 | 0 | 11 |
| Path 544 | C00031->C00002:[4->11,5->13,7->17,7->3,9->12,9->8] | 0.60 | 0.0 | 23 | 158 | 0 | 23 |
| Path 545 | C00031->C00002:[7->11,7->3,9->8] | 0.30 | 0.0 | 19 | 147 | 0 | 19 |
| Path 546 | C00031->C00002:[4->10,4->11,5->13,7->17,7->3,7->5,9->12,9->2,9->8] | 0.90 | 0.0 | 34 | 171 | 0 | 34 |
| Path 547 | C00031->C00002:[1->12,2->13,4->10,4->11,4->17,5->3,5->8] | 0.70 | 0.0 | 24 | 150 | 0 | 24 |
| Path 548 | C00031->C00002:[4->11,5->13,7->17,7->3,9->12,9->8] | 0.60 | 0.0 | 22 | 52 | 0 | 22 |
| Path 549 | C00031->C00002:[4->10,4->11,5->17,7->13,7->3,9->12,9->8] | 0.70 | 0.0 | 29 | 174 | 0 | 29 |
| Path 550 | C00031->C00002:[4->11,5->13,7->17,7->3,9->12,9->8] | 0.60 | 0.0 | 22 | 154 | 0 | 22 |
| Path 551 | C00031->C00002:[4->11,5->13,7->17,7->3,9->12,9->8] | 0.60 | 0.0 | 24 | 65 | 0 | 24 |
| Path 552 | C00031->C00002:[4->10,4->11,5->13,7->17,7->3,7->5,9->12,9->2,9->8] | 0.90 | 0.0 | 33 | 168 | 0 | 33 |
| Path 553 | C00031->C00002:[4->10,4->11,5->13,7->17,7->3,9->12,9->8] | 0.70 | 0.0 | 27 | 155 | 0 | 27 |
| Path 554 | C00031->C00002:[4->11,5->13,7->17,7->3,9->12,9->8] | 0.60 | 0.0 | 23 | 42 | 0 | 23 |
| Path 555 | C00031->C00002:[5->13,7->17,7->3,9->12,9->8] | 0.50 | 0.0 | 18 | 139 | 0 | 18 |
| Path 556 | C00031->C00002:[4->10,4->11,5->13,7->17,7->3,9->12,9->8] | 0.70 | 0.0 | 26 | 161 | 0 | 26 |
| Path 557 | C00031->C00002:[4->10,4->11,5->13,7->17,7->3,9->12,9->8] | 0.70 | 0.0 | 25 | 156 | 0 | 25 |
| Path 558 | C00031->C00002:[4->11,5->13,7->17,7->3,9->12,9->8] | 0.60 | 0.0 | 24 | 170 | 0 | 24 |
| Path 559 | C00031->C00002:[4->11,5->13,7->17,7->3,9->12,9->8] | 0.60 | 0.0 | 23 | 159 | 0 | 23 |
| Path 560 | C00031->C00002:[1->12,2->13,4->17,5->3,5->8] | 0.50 | 0.0 | 9 | 124 | 0 | 9 |
| Path 561 | C00031->C00002:[4->10,4->11,5->13,7->17,7->3,7->5,9->12,9->2,9->8] | 0.90 | 0.0 | 30 | 61 | 0 | 30 |
| Path 562 | C00031->C00002:[1->12,2->13,4->11,4->17,5->3,5->8] | 0.60 | 0.0 | 23 | 366 | 0 | 23 |
| Path 563 | C00031->C00002:[5->13,7->17,7->3,9->12,9->8] | 0.50 | 0.0 | 15 | 147 | 0 | 15 |
| Path 564 | C00031->C00002:[5->13,7->17,7->3,9->12,9->8] | 0.50 | 0.0 | 15 | 37 | 0 | 15 |
| Path 565 | C00031->C00002:[4->10,4->11,5->13,7->17,7->3,7->5,9->12,9->2,9->8] | 0.90 | 0.0 | 31 | 170 | 0 | 31 |
| Path 566 | C00031->C00002:[4->10,4->11,5->13,7->17,7->3,9->12,9->8] | 0.70 | 0.0 | 26 | 154 | 0 | 26 |
| Path 567 | C00031->C00002:[1->12,2->13,4->11,4->17,5->3,5->8] | 0.60 | 0.0 | 24 | 374 | 0 | 24 |
| Path 568 | C00031->C00002:[1->12,2->13,4->10,4->11,4->17,7->3,7->5,9->2,9->8] | 0.90 | 0.0 | 29 | 375 | 0 | 29 |
| Path 569 | C00031->C00002:[5->17,7->13,7->3,9->12,9->8] | 0.50 | 0.0 | 17 | 151 | 0 | 17 |
| Path 570 | C00031->C00002:[4->10,4->11,5->13,7->17,7->3,9->12,9->8] | 0.70 | 0.0 | 25 | 152 | 0 | 25 |
| Path 571 | C00031->C00002:[5->13,7->17,7->3,9->12,9->8] | 0.50 | 0.0 | 16 | 41 | 0 | 16 |
| Path 572 | C00031->C00002:[4->10,4->11,5->13,7->17,7->3,7->5,9->12,9->2,9->8] | 0.90 | 0.0 | 32 | 163 | 0 | 32 |
| Path 573 | C00031->C00002:[5->13,7->17,7->3,9->12,9->8] | 0.50 | 0.0 | 13 | 35 | 0 | 13 |
| Path 574 | C00031->C00002:[5->3,7->11] | 0.20 | 0.0 | 18 | 140 | 0 | 18 |
| Path 575 | C00031->C00002:[5->17,7->13,7->3,9->12,9->8] | 0.50 | 0.0 | 16 | 363 | 0 | 16 |
| Path 576 | C00031->C00002:[4->11,5->13,7->17,7->3,9->12,9->8] | 0.60 | 0.0 | 23 | 156 | 0 | 23 |
| Path 577 | C00031->C00002:[5->13,5->3,7->17,9->12,9->8] | 0.50 | 0.0 | 21 | 146 | 0 | 21 |
| Path 578 | C00031->C00002:[4->11,5->13,7->17,7->3,9->12,9->8] | 0.60 | 0.0 | 25 | 153 | 0 | 25 |
| Path 579 | C00031->C00002:[4->11,5->13,7->17,7->3,9->12,9->8] | 0.60 | 0.0 | 25 | 55 | 0 | 25 |
| Path 580 | C00031->C00002:[4->10,4->11,5->13,7->17,7->3,9->12,9->8] | 0.70 | 0.0 | 25 | 52 | 0 | 25 |
| Path 581 | C00031->C00002:[4->10,4->11,5->13,7->17,7->3,7->5,9->12,9->2,9->8] | 0.90 | 0.0 | 31 | 66 | 0 | 31 |
| Path 582 | C00031->C00002:[5->17,7->13,7->3,9->12,9->8] | 0.50 | 0.0 | 17 | 366 | 0 | 17 |
| Path 583 | C00031->C00002:[4->10,4->11,5->13,7->17,7->3,7->5,9->12,9->2,9->8] | 0.90 | 0.0 | 33 | 169 | 0 | 33 |
| Path 584 | C00031->C00002:[1->12,2->13,4->10,4->11,4->17,5->8,9->3] | 0.70 | 0.0 | 22 | 147 | 0 | 22 |
| Path 585 | C00031->C00002:[1->12,2->13,4->10,4->17,5->3,5->8] | 0.60 | 0.0 | 20 | 145 | 0 | 20 |
| Path 586 | C00031->C00002:[4->10,4->11,5->17,7->13,7->3,7->5,9->12,9->2,9->8] | 0.90 | 0.0 | 35 | 386 | 0 | 35 |
| Path 587 | C00031->C00002:[4->10,4->11,5->13,7->17,7->3,9->12,9->8] | 0.70 | 0.0 | 26 | 154 | 0 | 26 |
| Path 588 | C00031->C00002:[1->12,2->13,4->10,4->11,4->17,5->3,5->8,7->5,9->2] | 0.90 | 0.0 | 34 | 170 | 0 | 34 |
| Path 589 | C00031->C00002:[4->11,5->17,7->13,7->3,9->12,9->8] | 0.60 | 0.0 | 26 | 70 | 0 | 26 |
| Path 590 | C00031->C00002:[4->10,4->11,5->13,7->17,7->3,9->12,9->8] | 0.70 | 0.0 | 26 | 158 | 0 | 26 |
| Path 591 | C00031->C00002:[5->17,7->13,7->3,9->12,9->8] | 0.50 | 0.0 | 17 | 143 | 0 | 17 |
| Path 592 | C00031->C00002:[4->10,4->11,5->13,7->17,7->3,7->5,9->12,9->2,9->8] | 0.90 | 0.0 | 30 | 65 | 0 | 30 |
| Path 593 | C00031->C00002:[4->10,4->11,5->13,7->17,7->3,9->12,9->8] | 0.70 | 0.0 | 27 | 173 | 0 | 27 |
| Path 594 | C00031->C00002:[1->12,2->13,4->11,4->17,5->8,9->3] | 0.60 | 0.0 | 17 | 137 | 0 | 17 |
| Path 595 | C00031->C00002:[4->11,5->13,7->17,7->3,9->12,9->8] | 0.60 | 0.0 | 23 | 150 | 0 | 23 |
| Path 596 | C00031->C00002:[4->10,4->11,5->13,7->17,7->3,7->5,9->12,9->2,9->8] | 0.90 | 0.0 | 33 | 393 | 0 | 33 |
| Path 597 | C00031->C00002:[4->10,4->11,5->13,7->17,7->3,9->12,9->8] | 0.70 | 0.0 | 26 | 46 | 0 | 26 |
| Path 598 | C00031->C00002:[1->12,2->13,4->11,4->17,5->3,5->8] | 0.60 | 0.0 | 24 | 56 | 0 | 24 |
| Path 599 | C00031->C00002:[4->10,4->11,5->13,7->17,7->3,9->12,9->8] | 0.70 | 0.0 | 23 | 50 | 0 | 23 |
| Path 600 | C00031->C00002:[4->10,4->11,5->17,7->13,7->3,7->5,9->12,9->2,9->8] | 0.90 | 0.0 | 33 | 171 | 0 | 33 |
| Path 601 | C00031->C00002:[1->12,2->13,4->10,4->11,4->17,7->3,7->5,9->2,9->8] | 0.90 | 0.0 | 31 | 55 | 0 | 31 |
| Path 602 | C00031->C00002:[1->12,2->13,4->10,4->11,4->17,7->3,9->8] | 0.70 | 0.0 | 25 | 154 | 0 | 25 |
| Path 603 | C00031->C00002:[4->10,4->11,5->13,7->17,7->3,9->12,9->8] | 0.70 | 0.0 | 25 | 160 | 0 | 25 |
| Path 604 | C00031->C00002:[1->12,2->13,4->17,5->3,5->8] | 0.50 | 0.0 | 15 | 140 | 0 | 15 |
| Path 605 | C00031->C00002:[5->13,7->17,7->3,9->12,9->8] | 0.50 | 0.0 | 14 | 359 | 0 | 14 |
| Path 606 | C00031->C00002:[4->11,5->13,7->17,7->3,9->12,9->8] | 0.60 | 0.0 | 22 | 151 | 0 | 22 |
| Path 607 | C00031->C00002:[1->12,2->13,4->11,4->17,5->3,5->8] | 0.60 | 0.0 | 22 | 148 | 0 | 22 |
| Path 608 | C00031->C00002:[1->12,2->13,4->10,4->11,4->17,7->3,7->5,9->2,9->8] | 0.90 | 0.0 | 32 | 168 | 0 | 32 |
| Path 609 | C00031->C00002:[4->10,4->11,5->17,7->13,7->3,7->5,9->12,9->2,9->8] | 0.90 | 0.0 | 33 | 66 | 0 | 33 |
| Path 610 | C00031->C00002:[4->11,5->17,7->13,7->3,9->12,9->8] | 0.60 | 0.0 | 24 | 68 | 0 | 24 |
| Path 611 | C00031->C00002:[1->12,2->13,4->11,4->17,5->3,5->8] | 0.60 | 0.0 | 25 | 157 | 0 | 25 |
| Path 612 | C00031->C00002:[1->12,2->13,4->17,5->3,5->8] | 0.50 | 0.0 | 15 | 144 | 0 | 15 |
| Path 613 | C00031->C00002:[5->13,7->17,7->3,9->12,9->8] | 0.50 | 0.0 | 19 | 147 | 0 | 19 |
| Path 614 | C00031->C00002:[4->11,5->13,7->17,7->3,9->12,9->8] | 0.60 | 0.0 | 23 | 375 | 0 | 23 |
| Path 615 | C00031->C00002:[4->11,5->13,7->17,7->3,9->12,9->8] | 0.60 | 0.0 | 21 | 150 | 0 | 21 |
| Path 616 | C00031->C00002:[1->12,2->13,4->17,5->3,5->8] | 0.50 | 0.0 | 11 | 28 | 0 | 11 |
| Path 617 | C00031->C00002:[1->12,1->8,2->13,4->10,4->11,4->17,4->3,7->3,9->8] | 0.70 | 0.0 | 26 | 155 | 0 | 26 |
| Path 618 | C00031->C00002:[5->13,7->17,7->3,9->12,9->8] | 0.50 | 0.0 | 15 | 142 | 0 | 15 |
| Path 619 | C00031->C00002:[5->13,7->17,7->3,9->12,9->8] | 0.50 | 0.0 | 14 | 27 | 0 | 14 |
| Path 620 | C00031->C00002:[5->13,7->17,7->3,9->12,9->8] | 0.50 | 0.0 | 16 | 139 | 0 | 16 |
| Path 621 | C00031->C00002:[4->11,5->13,7->17,7->3,9->12,9->8] | 0.60 | 0.0 | 22 | 42 | 0 | 22 |
| Path 622 | C00031->C00002:[5->13,7->17,7->3,9->12,9->8] | 0.50 | 0.0 | 12 | 38 | 0 | 12 |
| Path 623 | C00031->C00002:[1->12,2->13,4->10,4->11,4->17,7->3,7->5,9->2,9->8] | 0.90 | 0.0 | 32 | 164 | 0 | 32 |
| Path 624 | C00031->C00002:[4->10,4->11,5->13,7->17,7->3,7->5,9->12,9->2,9->8] | 0.90 | 0.0 | 31 | 72 | 0 | 31 |
| Path 625 | C00031->C00002:[4->11,5->17,7->13,7->3,9->12,9->8] | 0.60 | 0.0 | 24 | 56 | 0 | 24 |
| Path 626 | C00031->C00002:[1->12,2->13,4->10,4->11,4->17,4->4,5->8,7->2,9->3,9->5] | 1.00 | 0.0 | 30 | 173 | 0 | 30 |
| Path 627 | C00031->C00002:[1->12,2->13,4->11,4->17,5->3,5->8] | 0.60 | 0.0 | 22 | 51 | 0 | 22 |
| Path 628 | C00031->C00002:[5->13,7->17,7->3,9->12,9->8] | 0.50 | 0.0 | 15 | 135 | 0 | 15 |
| Path 629 | C00031->C00002:[1->12,2->13,4->10,4->11,4->17,7->3,7->5,9->2,9->8] | 0.90 | 0.0 | 31 | 167 | 0 | 31 |
| Path 630 | C00031->C00002:[4->10,4->11,5->17,7->13,7->3,9->12,9->8] | 0.70 | 0.0 | 27 | 160 | 0 | 27 |
| Path 631 | C00031->C00002:[1->12,2->13,4->17,7->3,9->8] | 0.50 | 0.0 | 13 | 26 | 0 | 13 |
| Path 632 | C00031->C00002:[4->10,4->11,5->13,7->17,7->3,7->5,9->12,9->2,9->8] | 0.90 | 0.0 | 32 | 170 | 0 | 32 |
| Path 633 | C00031->C00002:[1->12,2->13,4->10,4->11,4->17,5->3,5->8] | 0.70 | 0.0 | 26 | 55 | 0 | 26 |
| Path 634 | C00031->C00002:[4->10,4->11,5->17,7->13,7->3,7->5,9->12,9->2,9->8] | 0.90 | 0.0 | 35 | 71 | 0 | 35 |
| Path 635 | C00031->C00002:[1->12,2->13,4->10,4->11,4->17,5->8,9->3] | 0.70 | 0.0 | 22 | 151 | 0 | 22 |
| Path 636 | C00031->C00002:[4->11,5->13,7->17,7->3,9->12,9->8] | 0.60 | 0.0 | 21 | 55 | 0 | 21 |
| Path 637 | C00031->C00002:[4->10,4->11,5->17,7->13,7->3,7->5,9->12,9->2,9->8] | 0.90 | 0.0 | 35 | 169 | 0 | 35 |
| Path 638 | C00031->C00002:[4->10,4->11,5->13,7->17,7->3,9->12,9->8] | 0.70 | 0.0 | 26 | 166 | 0 | 26 |
| Path 639 | C00031->C00002:[5->13,7->17,7->3,9->12,9->8] | 0.50 | 0.0 | 13 | 133 | 0 | 13 |
| Path 640 | C00031->C00002:[4->11,5->13,7->17,7->3,9->12,9->8] | 0.60 | 0.0 | 23 | 162 | 0 | 23 |
| Path 641 | C00031->C00002:[4->10,4->11,5->13,7->17,7->3,7->5,9->12,9->2,9->8] | 0.90 | 0.0 | 32 | 51 | 0 | 32 |
| Path 642 | C00031->C00002:[1->12,2->13,4->11,4->17,7->3,9->8] | 0.60 | 0.0 | 24 | 153 | 0 | 24 |
| Path 643 | C00031->C00002:[7->3,9->8] | 0.20 | 0.0 | 10 | 352 | 0 | 10 |
| Path 644 | C00031->C00002:[5->13,7->17,7->3,9->12,9->8] | 0.50 | 0.0 | 15 | 142 | 0 | 15 |
| Path 645 | C00031->C00002:[4->10,4->11,5->17,7->13,7->3,7->5,9->12,9->2,9->8] | 0.90 | 0.0 | 34 | 389 | 0 | 34 |
| Path 646 | C00031->C00002:[1->12,2->13,4->11,4->17,7->3,9->8] | 0.60 | 0.0 | 23 | 152 | 0 | 23 |
| Path 647 | C00031->C00002:[4->10,4->11,5->17,7->13,7->3,9->12,9->8] | 0.70 | 0.0 | 28 | 387 | 0 | 28 |
| Path 648 | C00031->C00002:[5->17,7->13,7->3,9->12,9->8] | 0.50 | 0.0 | 18 | 155 | 0 | 18 |
| Path 649 | C00031->C00002:[4->11,5->13,7->17,7->3,9->12,9->8] | 0.60 | 0.0 | 23 | 158 | 0 | 23 |
| Path 650 | C00031->C00002:[4->10,4->11,5->13,7->17,7->3,9->12,9->8] | 0.70 | 0.0 | 24 | 152 | 0 | 24 |
| Path 651 | C00031->C00002:[4->10,4->11,5->13,7->17,7->3,9->12,9->8] | 0.70 | 0.0 | 26 | 169 | 0 | 26 |
| Path 652 | C00031->C00002:[5->13,7->17,7->3,9->12,9->8] | 0.50 | 0.0 | 13 | 140 | 0 | 13 |
| Path 653 | C00031->C00002:[4->11,5->13,7->17,7->3,9->12,9->8] | 0.60 | 0.0 | 22 | 150 | 0 | 22 |
| Path 654 | C00031->C00002:[4->10,4->11,5->13,7->17,7->3,9->12,9->8] | 0.70 | 0.0 | 25 | 156 | 0 | 25 |
| Path 655 | C00031->C00002:[4->11,5->17,7->13,7->3,9->12,9->8] | 0.60 | 0.0 | 24 | 159 | 0 | 24 |
| Path 656 | C00031->C00002:[5->13,5->3,5->8,7->17,9->12] | 0.50 | 0.0 | 16 | 141 | 0 | 16 |
| Path 657 | C00031->C00002:[4->10,4->11,5->17,7->13,7->3,9->12,9->8] | 0.70 | 0.0 | 28 | 173 | 0 | 28 |
| Path 658 | C00031->C00002:[5->3,7->11] | 0.20 | 0.0 | 18 | 141 | 0 | 18 |
| Path 659 | C00031->C00002:[5->13,7->17,7->3,9->12,9->8] | 0.50 | 0.0 | 15 | 37 | 0 | 15 |
| Path 660 | C00031->C00002:[1->12,2->13,4->11,4->17,5->3,5->8] | 0.60 | 0.0 | 24 | 53 | 0 | 24 |
| Path 661 | C00031->C00002:[4->11,5->17,7->13,7->3,9->12,9->8] | 0.60 | 0.0 | 26 | 164 | 0 | 26 |
| Path 662 | C00031->C00002:[5->13,7->17,7->3,9->12,9->8] | 0.50 | 0.0 | 15 | 153 | 0 | 15 |
| Path 663 | C00031->C00002:[4->11,5->13,7->17,7->3,9->12,9->8] | 0.60 | 0.0 | 22 | 53 | 0 | 22 |
| Path 664 | C00031->C00002:[4->10,4->11,5->13,7->17,7->3,7->5,9->12,9->2,9->8] | 0.90 | 0.0 | 32 | 62 | 0 | 32 |
| Path 665 | C00031->C00002:[1->12,2->13,4->17,7->3,9->8] | 0.50 | 0.0 | 11 | 129 | 0 | 11 |
| Path 666 | C00031->C00002:[4->11,5->13,7->17,7->3,9->12,9->8] | 0.60 | 0.0 | 24 | 167 | 0 | 24 |
| Path 667 | C00031->C00002:[4->11,5->13,7->17,7->3,9->12,9->8] | 0.60 | 0.0 | 21 | 149 | 0 | 21 |
| Path 668 | C00031->C00002:[1->12,2->13,4->11,4->17,5->3,5->8] | 0.60 | 0.0 | 25 | 379 | 0 | 25 |
| Path 669 | C00031->C00002:[4->10,4->11,5->17,7->13,7->3,7->5,9->12,9->2,9->8] | 0.90 | 0.0 | 34 | 56 | 0 | 34 |
| Path 670 | C00031->C00002:[4->10,4->11,5->13,7->17,7->3,7->5,9->12,9->2,9->8] | 0.90 | 0.0 | 32 | 174 | 0 | 32 |
| Path 671 | C00031->C00002:[1->12,2->13,4->10,4->11,4->17,5->8,7->5,9->2,9->3] | 0.90 | 0.0 | 27 | 154 | 0 | 27 |
| Path 672 | C00031->C00002:[1->12,2->13,4->11,4->17,7->3,9->8] | 0.60 | 0.0 | 19 | 145 | 0 | 19 |
| Path 673 | C00031->C00002:[4->10,4->11,5->13,7->17,7->3,7->5,9->12,9->2,9->8] | 0.90 | 0.0 | 31 | 61 | 0 | 31 |
| Path 674 | C00031->C00002:[4->10,4->11,5->13,7->17,7->3,7->5,9->12,9->2,9->8] | 0.90 | 0.0 | 30 | 60 | 0 | 30 |
| Path 675 | C00031->C00002:[1->12,2->13,4->17,5->3,5->8,7->11] | 0.60 | 0.0 | 21 | 147 | 0 | 21 |
| Path 676 | C00031->C00002:[4->10,4->11,5->13,7->17,7->3,9->12,9->8] | 0.70 | 0.0 | 23 | 50 | 0 | 23 |
| Path 677 | C00031->C00002:[5->13,7->17,7->3,9->12,9->8] | 0.50 | 0.0 | 22 | 149 | 0 | 22 |
| Path 678 | C00031->C00002:[5->17,7->13,7->3,9->12,9->8] | 0.50 | 0.0 | 16 | 47 | 0 | 16 |
| Path 679 | C00031->C00002:[4->11,5->17,7->13,7->3,9->12,9->8] | 0.60 | 0.0 | 24 | 57 | 0 | 24 |
| Path 680 | C00031->C00002:[4->10,4->11,5->13,7->17,7->3,9->12,9->8] | 0.70 | 0.0 | 26 | 44 | 0 | 26 |
| Path 681 | C00031->C00002:[4->11,5->13,7->17,7->3,9->12,9->8] | 0.60 | 0.0 | 22 | 157 | 0 | 22 |
| Path 682 | C00031->C00002:[1->12,2->13,4->10,4->11,4->17,5->3,5->8] | 0.70 | 0.0 | 25 | 372 | 0 | 25 |
| Path 683 | C00031->C00002:[4->11,5->17,7->13,7->3,9->12,9->8] | 0.60 | 0.0 | 26 | 164 | 0 | 26 |
| Path 684 | C00031->C00002:[1->12,2->13,4->10,4->11,4->17,5->3,5->8] | 0.70 | 0.0 | 22 | 52 | 0 | 22 |
| Path 685 | C00031->C00002:[5->13,7->17,7->3,9->12,9->8] | 0.50 | 0.0 | 16 | 352 | 0 | 16 |
| Path 686 | C00031->C00002:[2->8,4->11,5->13,5->3,7->17,9->12] | 0.60 | 0.0 | 21 | 148 | 0 | 21 |
| Path 687 | C00031->C00002:[1->12,2->13,4->10,4->11,4->17,5->8,9->3] | 0.70 | 0.0 | 21 | 152 | 0 | 21 |
| Path 688 | C00031->C00002:[4->10,4->11,5->13,7->17,7->3,7->5,9->12,9->2,9->8] | 0.90 | 0.0 | 32 | 174 | 0 | 32 |
| Path 689 | C00031->C00002:[4->10,4->11,5->13,7->17,7->3,9->12,9->8] | 0.70 | 0.0 | 26 | 169 | 0 | 26 |
| Path 690 | C00031->C00002:[5->13,7->17,7->3,9->12,9->8] | 0.50 | 0.0 | 13 | 134 | 0 | 13 |
| Path 691 | C00031->C00002:[4->10,4->11,5->17,7->13,7->3,9->12,9->8] | 0.70 | 0.0 | 28 | 61 | 0 | 28 |
| Path 692 | C00031->C00002:[1->12,2->13,4->10,4->11,4->17,4->2,5->8,7->5,9->2,9->3] | 0.90 | 0.0 | 28 | 162 | 0 | 28 |
| Path 693 | C00031->C00002:[1->12,2->13,4->10,4->11,4->17,4->4,5->8,9->3] | 0.80 | 0.0 | 30 | 167 | 0 | 30 |
| Path 694 | C00031->C00002:[4->11,5->17,7->13,7->3,9->12,9->8] | 0.60 | 0.0 | 25 | 160 | 0 | 25 |
| Path 695 | C00031->C00002:[4->10,4->11,5->17,7->13,7->3,7->5,9->12,9->2,9->8] | 0.90 | 0.0 | 34 | 172 | 0 | 34 |
| Path 696 | C00031->C00002:[4->10,4->11,5->13,7->17,7->3,9->12,9->8] | 0.70 | 0.0 | 26 | 60 | 0 | 26 |
| Path 697 | C00031->C00002:[4->10,4->11,5->13,7->17,7->3,7->5,9->12,9->2,9->8] | 0.90 | 0.0 | 31 | 72 | 0 | 31 |
| Path 698 | C00031->C00002:[1->12,2->13,4->17,5->8,7->11,9->3] | 0.60 | 0.0 | 18 | 142 | 0 | 18 |
| Path 699 | C00031->C00002:[4->11,5->13,7->17,7->3,9->12,9->8] | 0.60 | 0.0 | 24 | 158 | 0 | 24 |
| Path 700 | C00031->C00002:[4->11,5->13,7->17,7->3,9->12,9->8] | 0.60 | 0.0 | 24 | 167 | 0 | 24 |
| Path 701 | C00031->C00002:[5->17,7->13,7->3,9->12,9->8] | 0.50 | 0.0 | 17 | 140 | 0 | 17 |
| Path 702 | C00031->C00002:[1->12,2->13,4->11,4->17,5->3,5->8] | 0.60 | 0.0 | 25 | 372 | 0 | 25 |
| Path 703 | C00031->C00002:[1->12,2->13,4->10,4->11,4->17,5->3,5->8] | 0.70 | 0.0 | 24 | 367 | 0 | 24 |
| Path 704 | C00031->C00002:[4->10,4->11,5->13,7->17,7->3,7->5,9->12,9->2,9->8] | 0.90 | 0.0 | 32 | 66 | 0 | 32 |
| Path 705 | C00031->C00002:[4->11,5->17,7->13,7->3,9->12,9->8] | 0.60 | 0.0 | 25 | 170 | 0 | 25 |
| Path 706 | C00031->C00002:[4->10,4->11,5->13,7->17,7->3,9->12,9->8] | 0.70 | 0.0 | 25 | 153 | 0 | 25 |
| Path 707 | C00031->C00002:[4->10,4->11,5->17,7->13,7->3,9->12,9->8] | 0.70 | 0.0 | 27 | 169 | 0 | 27 |
| Path 708 | C00031->C00002:[1->12,2->13,4->17,7->3,9->8] | 0.50 | 0.0 | 13 | 131 | 0 | 13 |
| Path 709 | C00031->C00002:[4->10,4->11,5->13,7->17,7->3,7->5,9->12,9->2,9->8] | 0.90 | 0.0 | 31 | 61 | 0 | 31 |
| Path 710 | C00031->C00002:[4->10,4->11,5->17,7->13,7->3,9->12,9->8] | 0.70 | 0.0 | 29 | 76 | 0 | 29 |
| Path 711 | C00031->C00002:[5->17,7->13,7->3,9->12,9->8] | 0.50 | 0.0 | 17 | 42 | 0 | 17 |
| Path 712 | C00031->C00002:[5->13,7->17,7->3,9->12,9->8] | 0.50 | 0.0 | 15 | 366 | 0 | 15 |
| Path 713 | C00031->C00002:[1->12,2->13,4->11,4->17,7->3,9->8] | 0.60 | 0.0 | 21 | 147 | 0 | 21 |
| Path 714 | C00031->C00002:[1->12,2->13,4->10,4->11,4->17,7->3,9->8] | 0.70 | 0.0 | 24 | 370 | 0 | 24 |
| Path 715 | C00031->C00002:[4->10,4->11,5->13,7->17,7->3,7->5,9->12,9->2,9->8] | 0.90 | 0.0 | 34 | 180 | 0 | 34 |
| Path 716 | C00031->C00002:[4->10,4->11,5->13,7->17,7->3,7->5,9->12,9->2,9->8] | 0.90 | 0.0 | 30 | 49 | 0 | 30 |
| Path 717 | C00031->C00002:[4->11,5->17,7->13,7->3,9->12,9->8] | 0.60 | 0.0 | 25 | 58 | 0 | 25 |
| Path 718 | C00031->C00002:[4->10,4->11,5->17,7->13,7->3,9->12,9->8] | 0.70 | 0.0 | 28 | 168 | 0 | 28 |
| Path 719 | C00031->C00002:[1->12,2->13,4->10,4->11,4->17,5->3,5->8] | 0.70 | 0.0 | 24 | 364 | 0 | 24 |
| Path 720 | C00031->C00002:[1->12,2->13,4->10,4->11,4->17,5->3,5->8] | 0.70 | 0.0 | 24 | 149 | 0 | 24 |
| Path 721 | C00031->C00002:[5->13,7->17,7->3,9->12,9->8] | 0.50 | 0.0 | 15 | 142 | 0 | 15 |
| Path 722 | C00031->C00002:[4->10,4->11,5->13,7->17,7->3,7->5,9->12,9->2,9->8] | 0.90 | 0.0 | 31 | 51 | 0 | 31 |
| Path 723 | C00031->C00002:[1->12,2->13,4->17,7->3,9->8] | 0.50 | 0.0 | 13 | 348 | 0 | 13 |
| Path 724 | C00031->C00002:[1->12,2->13,4->10,4->11,4->17,7->3,7->5,9->2,9->8] | 0.90 | 0.0 | 31 | 167 | 0 | 31 |
| Path 725 | C00031->C00002:[4->10,4->11,5->17,7->13,7->3,7->5,9->12,9->2,9->8] | 0.90 | 0.0 | 36 | 184 | 0 | 36 |
| Path 726 | C00031->C00002:[4->11,5->17,7->13,7->3,9->12,9->8] | 0.60 | 0.0 | 25 | 44 | 0 | 25 |
| Path 727 | C00031->C00002:[4->10,4->11,5->13,7->17,7->3,7->5,9->12,9->2,9->8] | 0.90 | 0.0 | 34 | 59 | 0 | 34 |
| Path 728 | C00031->C00002:[5->3,7->11] | 0.20 | 0.0 | 18 | 141 | 0 | 18 |
| Path 729 | C00031->C00002:[1->12,2->13,4->10,4->11,4->17,5->3,5->8,7->10,7->11,7->5,9->2] | 0.90 | 0.0 | 33 | 166 | 0 | 33 |
| Path 730 | C00031->C00002:[4->10,4->11,5->13,7->17,7->3,7->5,9->12,9->2,9->8] | 0.90 | 0.0 | 32 | 168 | 0 | 32 |
| Path 731 | C00031->C00002:[1->12,2->13,4->11,4->17,5->3,5->8] | 0.60 | 0.0 | 20 | 46 | 0 | 20 |
| Path 732 | C00031->C00002:[4->10,4->11,5->17,7->13,7->3,7->5,9->12,9->2,9->8] | 0.90 | 0.0 | 34 | 170 | 0 | 34 |
| Path 733 | C00031->C00002:[1->12,2->13,4->10,4->11,4->17,5->3,5->8,7->5,9->2] | 0.90 | 0.0 | 31 | 166 | 0 | 31 |
| Path 734 | C00031->C00002:[1->12,2->13,4->11,4->17,5->3,5->8] | 0.60 | 0.0 | 25 | 379 | 0 | 25 |
| Path 735 | C00031->C00002:[1->12,2->13,4->17,7->11,7->3,9->8] | 0.60 | 0.0 | 20 | 146 | 0 | 20 |
| Path 736 | C00031->C00002:[4->10,4->11,5->13,7->17,7->3,7->5,9->12,9->2,9->8] | 0.90 | 0.0 | 32 | 170 | 0 | 32 |
| Path 737 | C00031->C00002:[1->12,2->13,4->10,4->17,4->4,5->8,7->10,7->4,9->3] | 0.70 | 0.0 | 23 | 153 | 0 | 23 |
| Path 738 | C00031->C00002:[1->12,2->13,4->10,4->11,4->17,5->3,5->8] | 0.70 | 0.0 | 27 | 159 | 0 | 27 |
| Path 739 | C00031->C00002:[4->10,4->11,5->13,7->17,7->3,7->5,9->12,9->2,9->8] | 0.90 | 0.0 | 31 | 69 | 0 | 31 |
| Path 740 | C00031->C00002:[4->11,5->17,7->13,7->3,9->12,9->8] | 0.60 | 0.0 | 25 | 156 | 0 | 25 |
| Path 741 | C00031->C00002:[1->12,2->13,4->10,4->11,4->17,5->3,5->8] | 0.70 | 0.0 | 20 | 42 | 0 | 20 |
| Path 742 | C00031->C00002:[4->10,4->11,5->13,7->17,7->3,7->5,9->12,9->2,9->8] | 0.90 | 0.0 | 34 | 164 | 0 | 34 |
| Path 743 | C00031->C00002:[1->12,2->13,4->11,4->17,7->3,9->8] | 0.60 | 0.0 | 22 | 155 | 0 | 22 |
| Path 744 | C00031->C00002:[4->10,4->11,5->13,7->17,7->3,7->5,9->12,9->2,9->8] | 0.90 | 0.0 | 33 | 52 | 0 | 33 |
| Path 745 | C00031->C00002:[4->10,4->11,5->13,7->17,7->3,9->12,9->8] | 0.70 | 0.0 | 25 | 164 | 0 | 25 |
| Path 746 | C00031->C00002:[1->12,2->13,4->11,4->17,5->3,5->8] | 0.60 | 0.0 | 22 | 147 | 0 | 22 |
| Path 747 | C00031->C00002:[1->12,2->13,4->10,4->11,4->17,7->3,9->8] | 0.70 | 0.0 | 22 | 39 | 0 | 22 |
| Path 748 | C00031->C00002:[4->10,4->11,5->13,7->17,7->3,7->5,9->12,9->2,9->8] | 0.90 | 0.0 | 33 | 168 | 0 | 33 |
| Path 749 | C00031->C00002:[5->13,7->17,7->3,9->12,9->8] | 0.50 | 0.0 | 19 | 143 | 0 | 19 |
| Path 750 | C00031->C00002:[1->12,2->13,4->17,7->3,9->8] | 0.50 | 0.0 | 10 | 128 | 0 | 10 |
| Path 751 | C00031->C00002:[4->11,5->13,7->17,7->3,9->12,9->8] | 0.60 | 0.0 | 24 | 156 | 0 | 24 |
| Path 752 | C00031->C00002:[4->11,5->13,7->17,7->3,9->12,9->8] | 0.60 | 0.0 | 25 | 70 | 0 | 25 |
| Path 753 | C00031->C00002:[1->12,2->13,4->11,4->17,5->3,5->8] | 0.60 | 0.0 | 24 | 157 | 0 | 24 |
| Path 754 | C00031->C00002:[5->13,7->17,7->3,9->12,9->8] | 0.50 | 0.0 | 14 | 356 | 0 | 14 |
| Path 755 | C00031->C00002:[2->8,5->13,5->3,7->17,9->12] | 0.50 | 0.0 | 14 | 353 | 0 | 14 |
| Path 756 | C00031->C00002:[4->11,5->13,7->17,7->3,9->12,9->8] | 0.60 | 0.0 | 23 | 42 | 0 | 23 |
| Path 757 | C00031->C00002:[1->12,2->13,4->17,5->3,5->8,7->11] | 0.60 | 0.0 | 18 | 137 | 0 | 18 |
| Path 758 | C00031->C00002:[5->13,7->17,7->3,9->12,9->8] | 0.50 | 0.0 | 14 | 149 | 0 | 14 |
| Path 759 | C00031->C00002:[4->11,5->13,7->17,7->3,9->12,9->8] | 0.60 | 0.0 | 25 | 155 | 0 | 25 |
| Path 760 | C00031->C00002:[4->10,4->11,5->13,7->17,7->3,7->5,9->12,9->2,9->8] | 0.90 | 0.0 | 31 | 162 | 0 | 31 |
| Path 761 | C00031->C00002:[5->13,7->17,7->3,9->12,9->8] | 0.50 | 0.0 | 15 | 49 | 0 | 15 |
| Path 762 | C00031->C00002:[1->12,2->13,4->11,4->17,5->3,5->8] | 0.60 | 0.0 | 21 | 152 | 0 | 21 |
| Path 763 | C00031->C00002:[1->12,2->13,4->17,5->3,5->8,7->11] | 0.60 | 0.0 | 18 | 138 | 0 | 18 |
| Path 764 | C00031->C00002:[5->13,7->17,7->3,9->12,9->8] | 0.50 | 0.0 | 16 | 30 | 0 | 16 |
| Path 765 | C00031->C00002:[4->10,4->11,5->17,7->13,7->3,9->12,9->8] | 0.70 | 0.0 | 28 | 72 | 0 | 28 |
| Path 766 | C00031->C00002:[1->12,2->13,4->11,4->17,5->3,5->8] | 0.60 | 0.0 | 19 | 146 | 0 | 19 |
| Path 767 | C00031->C00002:[1->12,2->13,4->17,5->8,9->3] | 0.50 | 0.0 | 8 | 124 | 0 | 8 |
| Path 768 | C00031->C00002:[4->10,4->11,5->13,7->17,7->3,7->5,9->12,9->2,9->8] | 0.90 | 0.0 | 30 | 382 | 0 | 30 |
| Path 769 | C00031->C00002:[4->10,4->11,5->13,7->17,7->3,9->12,9->8] | 0.70 | 0.0 | 25 | 49 | 0 | 25 |
| Path 770 | C00031->C00002:[4->11,5->13,7->17,7->3,9->12,9->8] | 0.60 | 0.0 | 22 | 56 | 0 | 22 |
| Path 771 | C00031->C00002:[2->8,4->11,5->13,5->3,7->17,9->12] | 0.60 | 0.0 | 23 | 370 | 0 | 23 |
| Path 772 | C00031->C00002:[5->13,7->17,7->3,9->12,9->8] | 0.50 | 0.0 | 14 | 37 | 0 | 14 |
| Path 773 | C00031->C00002:[1->12,2->13,4->10,4->11,4->17,7->3,7->5,9->2,9->8] | 0.90 | 0.0 | 29 | 158 | 0 | 29 |
| Path 774 | C00031->C00002:[1->12,2->13,4->10,4->17,5->8,9->3] | 0.60 | 0.0 | 21 | 149 | 0 | 21 |
| Path 775 | C00031->C00002:[4->10,4->11,5->17,7->13,7->3,7->5,9->12,9->2,9->8] | 0.90 | 0.0 | 33 | 68 | 0 | 33 |
| Path 776 | C00031->C00002:[4->11,5->13,7->17,7->3,9->12,9->8] | 0.60 | 0.0 | 25 | 370 | 0 | 25 |
| Path 777 | C00031->C00002:[1->12,2->13,4->10,4->11,4->17,5->3,5->8,7->5,9->2] | 0.90 | 0.0 | 31 | 60 | 0 | 31 |
| Path 778 | C00031->C00002:[1->12,2->13,4->17,5->3,5->8] | 0.50 | 0.0 | 9 | 124 | 0 | 9 |
| Path 779 | C00031->C00002:[1->12,2->13,4->11,4->17,5->3,5->8] | 0.60 | 0.0 | 23 | 153 | 0 | 23 |
| Path 780 | C00031->C00002:[4->11,5->13,7->17,7->3,9->12,9->8] | 0.60 | 0.0 | 25 | 372 | 0 | 25 |
| Path 781 | C00031->C00002:[5->3,7->11] | 0.20 | 0.0 | 18 | 137 | 0 | 18 |
| Path 782 | C00031->C00002:[4->10,4->11,5->17,7->13,7->3,9->12,9->8] | 0.70 | 0.0 | 27 | 160 | 0 | 27 |
| Path 783 | C00031->C00002:[1->12,2->13,4->17,7->3,9->8] | 0.50 | 0.0 | 13 | 26 | 0 | 13 |
| Path 784 | C00031->C00002:[1->12,2->13,4->17,5->3,5->8,7->11] | 0.60 | 0.0 | 21 | 148 | 0 | 21 |
| Path 785 | C00031->C00002:[4->11,5->13,7->17,7->3,9->12,9->8] | 0.60 | 0.0 | 25 | 54 | 0 | 25 |
| Path 786 | C00031->C00002:[4->10,4->11,5->13,7->17,7->3,7->5,9->12,9->2,9->8] | 0.90 | 0.0 | 33 | 171 | 0 | 33 |
| Path 787 | C00031->C00002:[4->11,5->13,7->17,7->3,9->12,9->8] | 0.60 | 0.0 | 22 | 42 | 0 | 22 |
| Path 788 | C00031->C00002:[4->11,5->13,7->17,7->3,9->12,9->8] | 0.60 | 0.0 | 25 | 171 | 0 | 25 |
| Path 789 | C00031->C00002:[5->13,7->17,7->3,9->12,9->8] | 0.50 | 0.0 | 14 | 145 | 0 | 14 |
| Path 790 | C00031->C00002:[4->10,4->11,5->13,7->17,7->3,9->12,9->8] | 0.70 | 0.0 | 26 | 162 | 0 | 26 |
| Path 791 | C00031->C00002:[1->12,2->13,4->17,5->8,7->11] | 0.50 | 0.0 | 18 | 145 | 0 | 18 |
| Path 792 | C00031->C00002:[1->12,2->13,4->10,4->11,4->17,5->3,5->8] | 0.70 | 0.0 | 25 | 158 | 0 | 25 |
| Path 793 | C00031->C00002:[4->11,5->17,7->13,7->3,9->12,9->8] | 0.60 | 0.0 | 25 | 156 | 0 | 25 |
| Path 794 | C00031->C00002:[4->10,4->11,5->13,7->17,7->3,9->12,9->8] | 0.70 | 0.0 | 25 | 164 | 0 | 25 |
| Path 795 | C00031->C00002:[4->10,4->11,5->13,7->17,7->3,9->12,9->8] | 0.70 | 0.0 | 26 | 165 | 0 | 26 |
| Path 796 | C00031->C00002:[1->12,2->13,4->10,4->17,5->3,5->8] | 0.60 | 0.0 | 22 | 147 | 0 | 22 |
| Path 797 | C00031->C00002:[1->12,2->13,4->17,7->3,9->8] | 0.50 | 0.0 | 13 | 134 | 0 | 13 |
| Path 798 | C00031->C00002:[1->12,2->13,4->10,4->11,4->17,5->8,9->3] | 0.70 | 0.0 | 24 | 158 | 0 | 24 |
| Path 799 | C00031->C00002:[4->10,4->11,5->13,7->17,7->3,9->12,9->8] | 0.70 | 0.0 | 24 | 48 | 0 | 24 |
| Path 800 | C00031->C00002:[4->10,4->11,5->13,7->17,7->3,7->5,9->12,9->2,9->8] | 0.90 | 0.0 | 33 | 67 | 0 | 33 |
| Path 801 | C00031->C00002:[4->10,4->11,5->13,7->17,7->3,7->5,9->12,9->2,9->8] | 0.90 | 0.0 | 34 | 382 | 0 | 34 |
| Path 802 | C00031->C00002:[4->10,4->11,5->13,7->17,7->3,7->5,9->12,9->2,9->8] | 0.90 | 0.0 | 31 | 163 | 0 | 31 |
| Path 803 | C00031->C00002:[4->10,4->11,5->17,7->13,7->3,9->12,9->8] | 0.70 | 0.0 | 27 | 160 | 0 | 27 |
| Path 804 | C00031->C00002:[1->12,2->13,4->10,4->11,4->17,4->4,5->8,7->10,7->11,7->4,9->3] | 0.80 | 0.0 | 26 | 157 | 0 | 26 |
| Path 805 | C00031->C00002:[5->17,7->13,7->3,9->12,9->8] | 0.50 | 0.0 | 16 | 361 | 0 | 16 |
| Path 806 | C00031->C00002:[5->13,7->17,7->3,9->12,9->8] | 0.50 | 0.0 | 14 | 137 | 0 | 14 |
| Path 807 | C00031->C00002:[4->10,4->11,5->13,7->17,7->3,9->12,9->8] | 0.70 | 0.0 | 24 | 55 | 0 | 24 |
| Path 808 | C00031->C00002:[4->11,5->13,7->17,7->3,9->12,9->8] | 0.60 | 0.0 | 24 | 163 | 0 | 24 |
| Path 809 | C00031->C00002:[1->12,2->13,4->10,4->11,4->17,7->3,7->5,9->2,9->8] | 0.90 | 0.0 | 28 | 157 | 0 | 28 |
| Path 810 | C00031->C00002:[1->12,2->13,4->17,5->8,7->11] | 0.50 | 0.0 | 18 | 142 | 0 | 18 |
| Path 811 | C00031->C00002:[4->10,4->11,5->13,7->17,7->3,7->5,9->12,9->2,9->8] | 0.90 | 0.0 | 32 | 385 | 0 | 32 |
| Path 812 | C00031->C00002:[4->10,4->11,5->13,7->17,7->3,7->5,9->12,9->2,9->8] | 0.90 | 0.0 | 31 | 173 | 0 | 31 |
| Path 813 | C00031->C00002:[1->12,2->13,4->10,4->17,5->3,5->8] | 0.60 | 0.0 | 21 | 150 | 0 | 21 |
| Path 814 | C00031->C00002:[1->12,2->13,4->10,4->11,4->17,5->8,9->3] | 0.70 | 0.0 | 21 | 152 | 0 | 21 |
| Path 815 | C00031->C00002:[1->12,2->13,4->10,4->11,4->17,5->3,5->8,7->5,9->2] | 0.90 | 0.0 | 31 | 160 | 0 | 31 |
| Path 816 | C00031->C00002:[4->10,4->11,5->13,7->17,7->3,7->5,9->12,9->2,9->8] | 0.90 | 0.0 | 31 | 163 | 0 | 31 |
| Path 817 | C00031->C00002:[1->12,2->13,4->10,4->11,4->17,7->3,7->5,9->2,9->8] | 0.90 | 0.0 | 28 | 56 | 0 | 28 |
| Path 818 | C00031->C00002:[4->10,4->11,5->13,7->17,7->3,9->12,9->8] | 0.70 | 0.0 | 25 | 55 | 0 | 25 |
| Path 819 | C00031->C00002:[1->12,2->13,4->10,4->11,4->17,5->3,5->8] | 0.70 | 0.0 | 23 | 371 | 0 | 23 |
| Path 820 | C00031->C00002:[4->10,4->11,5->13,7->17,7->3,7->5,9->12,9->2,9->8] | 0.90 | 0.0 | 30 | 50 | 0 | 30 |
| Path 821 | C00031->C00002:[2->8,4->11,5->13,5->3,7->17,9->12] | 0.60 | 0.0 | 22 | 47 | 0 | 22 |
| Path 822 | C00031->C00002:[4->10,4->11,5->17,7->13,7->3,9->12,9->8] | 0.70 | 0.0 | 29 | 65 | 0 | 29 |
| Path 823 | C00031->C00002:[5->17,7->13,7->3,9->12,9->8] | 0.50 | 0.0 | 17 | 151 | 0 | 17 |
| Path 824 | C00031->C00002:[4->10,4->11,5->13,7->17,7->3,7->5,9->12,9->2,9->8] | 0.90 | 0.0 | 31 | 173 | 0 | 31 |
| Path 825 | C00031->C00002:[1->12,2->13,4->10,4->11,4->17,7->3,9->8] | 0.70 | 0.0 | 23 | 152 | 0 | 23 |
| Path 826 | C00031->C00002:[5->13,7->17,7->3,9->12,9->8] | 0.50 | 0.0 | 14 | 135 | 0 | 14 |
| Path 827 | C00031->C00002:[1->12,2->13,4->17,5->3,5->8,7->11] | 0.60 | 0.0 | 19 | 143 | 0 | 19 |
| Path 828 | C00031->C00002:[1->12,2->13,4->10,4->11,4->17,7->3,9->8] | 0.70 | 0.0 | 24 | 157 | 0 | 24 |
| Path 829 | C00031->C00002:[1->12,2->13,4->17,5->8,9->11,9->3] | 0.60 | 0.0 | 16 | 136 | 0 | 16 |
| Path 830 | C00031->C00002:[1->12,2->13,4->10,4->11,4->17,4->4,5->8,9->3] | 0.80 | 0.0 | 27 | 157 | 0 | 27 |
| Path 831 | C00031->C00002:[5->13,7->17,7->3,9->12,9->8] | 0.50 | 0.0 | 16 | 42 | 0 | 16 |
| Path 832 | C00031->C00002:[5->17,7->13,7->3,9->12,9->8] | 0.50 | 0.0 | 17 | 140 | 0 | 17 |
| Path 833 | C00031->C00002:[5->13,7->17,7->3,9->12,9->8] | 0.50 | 0.0 | 15 | 141 | 0 | 15 |
| Path 834 | C00031->C00002:[1->12,2->13,4->10,4->11,4->17,5->3,5->8,7->5,9->2] | 0.90 | 0.0 | 31 | 58 | 0 | 31 |
| Path 835 | C00031->C00002:[4->11,5->13,7->17,7->3,9->12,9->8] | 0.60 | 0.0 | 22 | 53 | 0 | 22 |
| Path 836 | C00031->C00002:[4->10,4->11,5->13,7->17,7->3,9->12,9->8] | 0.70 | 0.0 | 25 | 59 | 0 | 25 |
| Path 837 | C00031->C00002:[5->17,7->13,7->3,9->12,9->8] | 0.50 | 0.0 | 17 | 145 | 0 | 17 |
| Path 838 | C00031->C00002:[1->12,2->13,4->10,4->11,4->17,5->3,5->8,7->5,9->2] | 0.90 | 0.0 | 33 | 161 | 0 | 33 |
| Path 839 | C00031->C00002:[5->13,7->17,7->3,9->12,9->8] | 0.50 | 0.0 | 13 | 31 | 0 | 13 |
| Path 840 | C00031->C00002:[4->10,4->11,5->13,7->17,7->3,9->12,9->8] | 0.70 | 0.0 | 25 | 48 | 0 | 25 |
| Path 841 | C00031->C00002:[1->12,2->13,4->10,4->11,4->17,5->3,5->8,7->5,9->2] | 0.90 | 0.0 | 31 | 160 | 0 | 31 |
| Path 842 | C00031->C00002:[4->10,4->11,5->13,7->17,7->3,7->5,9->12,9->2,9->8] | 0.90 | 0.0 | 32 | 62 | 0 | 32 |
| Path 843 | C00031->C00002:[5->17,7->13,7->3,9->12,9->8] | 0.50 | 0.0 | 16 | 146 | 0 | 16 |
| Path 844 | C00031->C00002:[4->10,4->11,5->13,7->17,7->3,9->12,9->8] | 0.70 | 0.0 | 26 | 55 | 0 | 26 |
| Path 845 | C00031->C00002:[4->11,5->13,7->17,7->3,9->12,9->8] | 0.60 | 0.0 | 22 | 165 | 0 | 22 |
| Path 846 | C00031->C00002:[7->11,7->3,9->8] | 0.30 | 0.0 | 19 | 148 | 0 | 19 |
| Path 847 | C00031->C00002:[4->11,5->13,7->17,7->3,9->12,9->8] | 0.60 | 0.0 | 21 | 37 | 0 | 21 |
| Path 848 | C00031->C00002:[7->3,9->8] | 0.20 | 0.0 | 10 | 135 | 0 | 10 |
| Path 849 | C00031->C00002:[4->10,4->11,5->17,7->13,7->3,7->5,9->12,9->2,9->8] | 0.90 | 0.0 | 33 | 167 | 0 | 33 |
| Path 850 | C00031->C00002:[1->12,2->13,4->10,4->11,4->17,5->3,5->8,7->5,9->2] | 0.90 | 0.0 | 29 | 155 | 0 | 29 |
| Path 851 | C00031->C00002:[4->11,5->13,7->17,7->3,9->12,9->8] | 0.60 | 0.0 | 24 | 159 | 0 | 24 |
| Path 852 | C00031->C00002:[1->12,2->13,4->17,5->8,7->11,9->3] | 0.60 | 0.0 | 17 | 142 | 0 | 17 |
| Path 853 | C00031->C00002:[4->11,5->17,7->13,7->3,9->12,9->8] | 0.60 | 0.0 | 27 | 172 | 0 | 27 |
| Path 854 | C00031->C00002:[1->12,2->13,4->10,4->11,4->17,7->3,9->8] | 0.70 | 0.0 | 24 | 52 | 0 | 24 |
| Path 855 | C00031->C00002:[5->13,7->17,7->3,9->12,9->8] | 0.50 | 0.0 | 14 | 38 | 0 | 14 |
| Path 856 | C00031->C00002:[1->12,1->8,2->13,4->11,4->17,4->3,7->3,9->8] | 0.60 | 0.0 | 23 | 152 | 0 | 23 |
| Path 857 | C00031->C00002:[1->12,2->13,4->17,5->3,5->8,7->11] | 0.60 | 0.0 | 20 | 143 | 0 | 20 |
| Path 858 | C00031->C00002:[4->11,5->13,7->17,7->3,9->12,9->8] | 0.60 | 0.0 | 22 | 57 | 0 | 22 |
| Path 859 | C00031->C00002:[1->12,2->13,4->10,4->11,4->17,5->3,5->8,7->5,9->2] | 0.90 | 0.0 | 27 | 153 | 0 | 27 |
| Path 860 | C00031->C00002:[1->12,2->13,4->17,5->3,5->8,7->11] | 0.60 | 0.0 | 22 | 149 | 0 | 22 |
| Path 861 | C00031->C00002:[5->13,7->17,7->3,9->12,9->8] | 0.50 | 0.0 | 15 | 141 | 0 | 15 |
| Path 862 | C00031->C00002:[1->12,2->13,4->17,7->3,9->8] | 0.50 | 0.0 | 11 | 346 | 0 | 11 |
| Path 863 | C00031->C00002:[7->12] | 0.10 | 0.0 | 11 | 131 | 0 | 11 |
| Path 864 | C00031->C00002:[1->12,2->13,4->10,4->11,4->17,4->4,5->8,7->2,9->3,9->5] | 1.00 | 0.0 | 30 | 170 | 0 | 30 |
| Path 865 | C00031->C00002:[4->10,4->11,5->17,7->13,7->3,7->5,9->12,9->2,9->8] | 0.90 | 0.0 | 34 | 172 | 0 | 34 |
| Path 866 | C00031->C00002:[4->10,4->11,5->17,7->13,7->3,7->5,9->12,9->2,9->8] | 0.90 | 0.0 | 35 | 183 | 0 | 35 |
| Path 867 | C00031->C00002:[4->10,4->11,5->17,7->13,7->3,9->12,9->8] | 0.70 | 0.0 | 27 | 60 | 0 | 27 |
| Path 868 | C00031->C00002:[4->10,4->11,5->13,7->17,7->3,7->5,9->12,9->2,9->8] | 0.90 | 0.0 | 33 | 63 | 0 | 33 |
| Path 869 | C00031->C00002:[4->10,4->11,5->13,5->3,5->8,7->17,9->12] | 0.70 | 0.0 | 27 | 377 | 0 | 27 |
| Path 870 | C00031->C00002:[4->10,4->11,5->17,7->13,7->3,7->5,9->12,9->2,9->8] | 0.90 | 0.0 | 35 | 169 | 0 | 35 |
| Path 871 | C00031->C00002:[1->12,2->13,4->10,4->11,4->17,5->3,5->8,7->5,9->2] | 0.90 | 0.0 | 27 | 154 | 0 | 27 |
| Path 872 | C00031->C00002:[4->10,4->11,5->17,7->13,7->3,7->5,9->12,9->2,9->8] | 0.90 | 0.0 | 33 | 169 | 0 | 33 |
| Path 873 | C00031->C00002:[5->13,7->17,7->3,9->12,9->8] | 0.50 | 0.0 | 23 | 150 | 0 | 23 |
| Path 874 | C00031->C00002:[1->12,2->13,4->10,4->11,4->17,4->4,5->8,7->10,7->11,7->4,9->3] | 0.80 | 0.0 | 29 | 164 | 0 | 29 |
| Path 875 | C00031->C00002:[1->12,2->13,4->17,5->3,5->8,7->11] | 0.60 | 0.0 | 20 | 140 | 0 | 20 |
| Path 876 | C00031->C00002:[5->17,7->13,7->3,9->12,9->8] | 0.50 | 0.0 | 16 | 153 | 0 | 16 |
| Path 877 | C00031->C00002:[1->12,2->13,4->11,4->17,7->3,9->8] | 0.60 | 0.0 | 21 | 46 | 0 | 21 |
| Path 878 | C00031->C00002:[4->11,5->17,7->13,7->3,9->12,9->8] | 0.60 | 0.0 | 24 | 58 | 0 | 24 |
| Path 879 | C00031->C00002:[1->12,2->13,4->10,4->11,4->17,7->3,9->8] | 0.70 | 0.0 | 22 | 151 | 0 | 22 |
| Path 880 | C00031->C00002:[5->13,7->17,7->3,9->12,9->8] | 0.50 | 0.0 | 14 | 25 | 0 | 14 |
| Path 881 | C00031->C00002:[4->10,4->11,5->13,7->17,7->3,7->5,9->12,9->2,9->8] | 0.90 | 0.0 | 30 | 64 | 0 | 30 |
| Path 882 | C00031->C00002:[1->12,2->13,4->10,4->11,4->17,4->4,5->8,7->2,9->3,9->5] | 1.00 | 0.0 | 32 | 176 | 0 | 32 |
| Path 883 | C00031->C00002:[5->13,7->17,7->3,9->12,9->8] | 0.50 | 0.0 | 19 | 147 | 0 | 19 |
| Path 884 | C00031->C00002:[5->13,7->17,7->3,9->12,9->8] | 0.50 | 0.0 | 12 | 132 | 0 | 12 |
| Path 885 | C00031->C00002:[5->13,7->17,7->3,9->12,9->8] | 0.50 | 0.0 | 14 | 47 | 0 | 14 |
| Path 886 | C00031->C00002:[5->13,7->17,7->3,9->12,9->8] | 0.50 | 0.0 | 15 | 135 | 0 | 15 |
| Path 887 | C00031->C00002:[4->10,4->11,5->13,7->17,7->3,7->5,9->12,9->2,9->8] | 0.90 | 0.0 | 32 | 171 | 0 | 32 |
| Path 888 | C00031->C00002:[4->10,4->11,5->13,7->17,7->3,7->5,9->12,9->2,9->8] | 0.90 | 0.0 | 33 | 70 | 0 | 33 |
| Path 889 | C00031->C00002:[4->11,5->13,7->17,7->3,9->12,9->8] | 0.60 | 0.0 | 24 | 152 | 0 | 24 |
| Path 890 | C00031->C00002:[4->11,5->13,7->17,7->3,9->12,9->8] | 0.60 | 0.0 | 24 | 155 | 0 | 24 |
| Path 891 | C00031->C00002:[1->12,2->13,4->10,4->11,4->17,5->3,5->8] | 0.70 | 0.0 | 21 | 148 | 0 | 21 |
| Path 892 | C00031->C00002:[4->10,4->11,5->13,7->17,7->3,7->5,9->12,9->2,9->8] | 0.90 | 0.0 | 32 | 62 | 0 | 32 |
| Path 893 | C00031->C00002:[4->11,5->13,7->17,7->3,9->12,9->8] | 0.60 | 0.0 | 25 | 168 | 0 | 25 |
| Path 894 | C00031->C00002:[4->11,5->17,7->13,7->3,9->12,9->8] | 0.60 | 0.0 | 25 | 161 | 0 | 25 |
| Path 895 | C00031->C00002:[1->12,2->13,4->10,4->11,4->17,5->3,5->8] | 0.70 | 0.0 | 23 | 157 | 0 | 23 |
| Path 896 | C00031->C00002:[4->10,4->11,5->13,7->17,7->3,7->5,9->12,9->2,9->8] | 0.90 | 0.0 | 32 | 69 | 0 | 32 |
| Path 897 | C00031->C00002:[5->13,7->17,7->3,9->12,9->8] | 0.50 | 0.0 | 20 | 144 | 0 | 20 |
| Path 898 | C00031->C00002:[4->10,4->11,5->17,7->13,7->3,9->12,9->8] | 0.70 | 0.0 | 26 | 162 | 0 | 26 |
| Path 899 | C00031->C00002:[5->13,7->17,7->3,9->12,9->8] | 0.50 | 0.0 | 13 | 25 | 0 | 13 |
| Path 900 | C00031->C00002:[5->13,7->17,7->3,9->12,9->8] | 0.50 | 0.0 | 16 | 139 | 0 | 16 |
| Path 901 | C00031->C00002:[1->12,2->13,4->11,4->17,7->3,9->8] | 0.60 | 0.0 | 22 | 148 | 0 | 22 |
| Path 902 | C00031->C00002:[4->10,4->11,5->13,7->17,7->3,9->12,9->8] | 0.70 | 0.0 | 25 | 60 | 0 | 25 |
| Path 903 | C00031->C00002:[4->10,4->11,5->13,7->17,7->3,9->12,9->8] | 0.70 | 0.0 | 24 | 156 | 0 | 24 |
| Path 904 | C00031->C00002:[5->13,7->17,7->3,9->12,9->8] | 0.50 | 0.0 | 16 | 26 | 0 | 16 |
| Path 905 | C00031->C00002:[1->12,2->13,4->10,4->11,4->17,7->3,7->5,9->2,9->8] | 0.90 | 0.0 | 30 | 159 | 0 | 30 |
| Path 906 | C00031->C00002:[4->10,4->11,5->13,7->17,7->3,7->5,9->12,9->2,9->8] | 0.90 | 0.0 | 33 | 171 | 0 | 33 |
| Path 907 | C00031->C00002:[1->12,2->13,4->10,4->11,4->17,7->3,9->8] | 0.70 | 0.0 | 23 | 40 | 0 | 23 |
| Path 908 | C00031->C00002:[5->13,7->17,7->3,9->12,9->8] | 0.50 | 0.0 | 12 | 35 | 0 | 12 |
| Path 909 | C00031->C00002:[5->17,7->13,7->3,9->12,9->8] | 0.50 | 0.0 | 17 | 357 | 0 | 17 |
| Path 910 | C00031->C00002:[4->10,4->11,5->13,7->17,7->3,9->12,9->8] | 0.70 | 0.0 | 27 | 155 | 0 | 27 |
| Path 911 | C00031->C00002:[1->12,2->13,4->11,4->17,5->8,9->3] | 0.60 | 0.0 | 18 | 147 | 0 | 18 |
| Path 912 | C00031->C00002:[5->13,7->17,7->3,9->12,9->8] | 0.50 | 0.0 | 15 | 146 | 0 | 15 |
| Path 913 | C00031->C00002:[5->17,7->13,7->3,9->12,9->8] | 0.50 | 0.0 | 16 | 146 | 0 | 16 |
| Path 914 | C00031->C00002:[2->8,5->13,5->3,7->17,9->12] | 0.50 | 0.0 | 14 | 31 | 0 | 14 |
| Path 915 | C00031->C00002:[1->12,1->8,2->13,4->17,4->3,7->3,9->8] | 0.50 | 0.0 | 15 | 139 | 0 | 15 |
| Path 916 | C00031->C00002:[2->8,4->11,5->13,5->3,7->17,9->12] | 0.60 | 0.0 | 22 | 148 | 0 | 22 |
| Path 917 | C00031->C00002:[4->10,4->11,5->17,7->13,7->3,9->12,9->8] | 0.70 | 0.0 | 27 | 158 | 0 | 27 |
| Path 918 | C00031->C00002:[4->10,4->11,5->13,7->17,7->3,9->12,9->8] | 0.70 | 0.0 | 25 | 160 | 0 | 25 |
| Path 919 | C00031->C00002:[1->12,2->13,4->11,4->17,7->3,9->8] | 0.60 | 0.0 | 21 | 49 | 0 | 21 |
| Path 920 | C00031->C00002:[5->17,7->13,7->3,9->12,9->8] | 0.50 | 0.0 | 18 | 158 | 0 | 18 |
| Path 921 | C00031->C00002:[5->17,7->13,7->3,9->12,9->8] | 0.50 | 0.0 | 16 | 41 | 0 | 16 |
| Path 922 | C00031->C00002:[1->12,2->13,4->10,4->11,4->17,5->3,5->8,7->5,9->2] | 0.90 | 0.0 | 32 | 161 | 0 | 32 |
| Path 923 | C00031->C00002:[1->12,2->13,4->10,4->11,4->17,7->3,7->5,9->2,9->8] | 0.90 | 0.0 | 31 | 167 | 0 | 31 |
| Path 924 | C00031->C00002:[4->11,5->13,7->17,7->3,9->12,9->8] | 0.60 | 0.0 | 23 | 154 | 0 | 23 |
| Path 925 | C00031->C00002:[4->11,5->13,7->17,7->3,9->12,9->8] | 0.60 | 0.0 | 23 | 152 | 0 | 23 |
| Path 926 | C00031->C00002:[1->12,2->13,4->11,4->17,7->3,9->8] | 0.60 | 0.0 | 19 | 44 | 0 | 19 |
| Path 927 | C00031->C00002:[1->12,2->13,4->10,4->11,4->17,7->3,9->8] | 0.70 | 0.0 | 25 | 154 | 0 | 25 |
| Path 928 | C00031->C00002:[4->10,4->11,5->13,7->17,7->3,9->12,9->8] | 0.70 | 0.0 | 24 | 167 | 0 | 24 |
| Path 929 | C00031->C00002:[7->3,9->8] | 0.20 | 0.0 | 10 | 128 | 0 | 10 |
| Path 930 | C00031->C00002:[4->11,5->13,7->17,7->3,9->12,9->8] | 0.60 | 0.0 | 24 | 65 | 0 | 24 |
| Path 931 | C00031->C00002:[5->13,7->17,7->3,9->12,9->8] | 0.50 | 0.0 | 16 | 31 | 0 | 16 |
| Path 932 | C00031->C00002:[1->12,2->13,4->10,4->11,4->17,7->3,9->8] | 0.70 | 0.0 | 24 | 157 | 0 | 24 |
| Path 933 | C00031->C00002:[4->10,4->11,5->13,7->17,7->3,7->5,9->12,9->2,9->8] | 0.90 | 0.0 | 33 | 67 | 0 | 33 |
| Path 934 | C00031->C00002:[4->11,5->17,7->13,7->3,9->12,9->8] | 0.60 | 0.0 | 27 | 74 | 0 | 27 |
| Path 935 | C00031->C00002:[1->12,2->13,4->11,4->17,5->3,5->8] | 0.60 | 0.0 | 25 | 161 | 0 | 25 |
| Path 936 | C00031->C00002:[4->11,5->13,7->17,7->3,9->12,9->8] | 0.60 | 0.0 | 25 | 168 | 0 | 25 |
| Path 937 | C00031->C00002:[5->13,7->17,7->3,9->12,9->8] | 0.50 | 0.0 | 13 | 37 | 0 | 13 |
| Path 938 | C00031->C00002:[5->3,7->11] | 0.20 | 0.0 | 19 | 142 | 0 | 19 |
| Path 939 | C00031->C00002:[5->13,7->17,7->3,9->12,9->8] | 0.50 | 0.0 | 16 | 143 | 0 | 16 |
| Path 940 | C00031->C00002:[4->10,4->11,5->13,7->17,7->3,7->5,9->12,9->2,9->8] | 0.90 | 0.0 | 33 | 168 | 0 | 33 |
| Path 941 | C00031->C00002:[4->10,4->11,5->13,7->17,7->3,7->5,9->12,9->2,9->8] | 0.90 | 0.0 | 30 | 161 | 0 | 30 |
| Path 942 | C00031->C00002:[4->10,4->11,5->13,7->17,7->3,7->5,9->12,9->2,9->8] | 0.90 | 0.0 | 34 | 70 | 0 | 34 |
| Path 943 | C00031->C00002:[4->10,4->11,5->13,7->17,7->3,9->12,9->8] | 0.70 | 0.0 | 26 | 48 | 0 | 26 |
| Path 944 | C00031->C00002:[1->12,2->13,4->17,5->8,7->11,9->3] | 0.60 | 0.0 | 18 | 139 | 0 | 18 |
| Path 945 | C00031->C00002:[4->10,4->11,5->13,7->17,7->3,7->5,9->12,9->2,9->8] | 0.90 | 0.0 | 30 | 165 | 0 | 30 |
| Path 946 | C00031->C00002:[5->13,7->17,7->3,9->12,9->8] | 0.50 | 0.0 | 23 | 151 | 0 | 23 |
| Path 947 | C00031->C00002:[5->13,7->17,7->3,9->12,9->8] | 0.50 | 0.0 | 16 | 143 | 0 | 16 |
| Path 948 | C00031->C00002:[1->12,2->13,4->10,4->11,4->17,5->3,5->8,7->10,7->11] | 0.70 | 0.0 | 25 | 159 | 0 | 25 |
| Path 949 | C00031->C00002:[4->11,5->13,7->17,7->3,9->12,9->8] | 0.60 | 0.0 | 21 | 48 | 0 | 21 |
| Path 950 | C00031->C00002:[1->12,2->13,4->10,4->11,4->17,5->3,5->8,7->10,7->11] | 0.70 | 0.0 | 26 | 153 | 0 | 26 |
| Path 951 | C00031->C00002:[5->13,7->17,7->3,9->12,9->8] | 0.50 | 0.0 | 16 | 53 | 0 | 16 |
| Path 952 | C00031->C00002:[5->13,7->17,7->3,9->12,9->8] | 0.50 | 0.0 | 14 | 359 | 0 | 14 |
| Path 953 | C00031->C00002:[4->10,4->11,5->13,7->17,7->3,9->12,9->8] | 0.70 | 0.0 | 26 | 385 | 0 | 26 |
| Path 954 | C00031->C00002:[4->11,5->17,7->13,7->3,9->12,9->8] | 0.60 | 0.0 | 27 | 172 | 0 | 27 |
| Path 955 | C00031->C00002:[5->13,7->17,7->3,9->12,9->8] | 0.50 | 0.0 | 14 | 41 | 0 | 14 |
| Path 956 | C00031->C00002:[4->10,4->11,5->13,7->17,7->3,7->5,9->12,9->2,9->8] | 0.90 | 0.0 | 30 | 64 | 0 | 30 |
| Path 957 | C00031->C00002:[4->11,5->13,7->17,7->3,9->12,9->8] | 0.60 | 0.0 | 23 | 154 | 0 | 23 |
| Path 958 | C00031->C00002:[1->12,2->13,4->10,4->11,4->17,4->4,5->8,9->3] | 0.80 | 0.0 | 27 | 156 | 0 | 27 |
| Path 959 | C00031->C00002:[5->17,7->13,7->3,9->12,9->8] | 0.50 | 0.0 | 15 | 42 | 0 | 15 |
| Path 960 | C00031->C00002:[5->13,7->17,7->3,9->12,9->8] | 0.50 | 0.0 | 15 | 145 | 0 | 15 |
| Path 961 | C00031->C00002:[4->10,4->11,5->13,7->17,7->3,9->12,9->8] | 0.70 | 0.0 | 26 | 56 | 0 | 26 |
| Path 962 | C00031->C00002:[4->11,5->17,7->13,7->3,9->12,9->8] | 0.60 | 0.0 | 26 | 171 | 0 | 26 |
| Path 963 | C00031->C00002:[1->12,2->13,4->17,5->3,5->8] | 0.50 | 0.0 | 12 | 29 | 0 | 12 |
| Path 964 | C00031->C00002:[2->8,4->10,4->11,5->13,5->3,7->17,9->12] | 0.70 | 0.0 | 24 | 368 | 0 | 24 |
| Path 965 | C00031->C00002:[1->12,2->13,4->10,4->11,4->17,5->3,5->8] | 0.70 | 0.0 | 27 | 381 | 0 | 27 |
| Path 966 | C00031->C00002:[1->12,2->13,4->17,7->3,9->8] | 0.50 | 0.0 | 13 | 138 | 0 | 13 |
| Path 967 | C00031->C00002:[4->10,4->11,5->17,7->13,7->3,7->5,9->12,9->2,9->8] | 0.90 | 0.0 | 34 | 172 | 0 | 34 |
| Path 968 | C00031->C00002:[5->13,7->17,7->3,9->12,9->8] | 0.50 | 0.0 | 14 | 138 | 0 | 14 |
| Path 969 | C00031->C00002:[4->10,4->11,5->13,7->17,7->3,7->5,9->12,9->2,9->8] | 0.90 | 0.0 | 29 | 164 | 0 | 29 |
| Path 970 | C00031->C00002:[4->10,4->11,5->13,7->17,7->3,7->5,9->12,9->2,9->8] | 0.90 | 0.0 | 31 | 61 | 0 | 31 |
| Path 971 | C00031->C00002:[4->11,5->13,7->17,7->3,9->12,9->8] | 0.60 | 0.0 | 23 | 154 | 0 | 23 |
| Path 972 | C00031->C00002:[1->12,2->13,4->10,4->11,4->17,5->3,5->8,7->5,9->2] | 0.90 | 0.0 | 30 | 59 | 0 | 30 |
| Path 973 | C00031->C00002:[1->12,2->13,4->10,4->11,4->17,7->3,9->8] | 0.70 | 0.0 | 24 | 150 | 0 | 24 |
| Path 974 | C00031->C00002:[2->8,5->13,5->3,7->17,9->12] | 0.50 | 0.0 | 13 | 349 | 0 | 13 |
| Path 975 | C00031->C00002:[4->10,4->11,5->13,7->17,7->3,9->12,9->8] | 0.70 | 0.0 | 27 | 376 | 0 | 27 |
| Path 976 | C00031->C00002:[4->11,5->13,7->17,7->3,9->12,9->8] | 0.60 | 0.0 | 24 | 159 | 0 | 24 |
| Path 977 | C00031->C00002:[4->10,4->11,5->13,7->17,7->3,7->5,9->12,9->2,9->8] | 0.90 | 0.0 | 35 | 177 | 0 | 35 |
| Path 978 | C00031->C00002:[7->3,9->8] | 0.20 | 0.0 | 10 | 23 | 0 | 10 |
| Path 979 | C00031->C00002:[4->10,4->11,5->13,7->17,7->3,9->12,9->8] | 0.70 | 0.0 | 23 | 46 | 0 | 23 |
| Path 980 | C00031->C00002:[1->12,2->13,4->10,4->11,4->17,5->3,5->8,7->5,9->2] | 0.90 | 0.0 | 30 | 373 | 0 | 30 |
| Path 981 | C00031->C00002:[1->12,2->13,4->10,4->11,4->17,5->3,5->8,7->5,9->2] | 0.90 | 0.0 | 27 | 53 | 0 | 27 |
| Path 982 | C00031->C00002:[5->13,7->17,7->3,9->12,9->8] | 0.50 | 0.0 | 20 | 148 | 0 | 20 |
| Path 983 | C00031->C00002:[4->10,4->11,5->13,7->17,7->3,9->12,9->8] | 0.70 | 0.0 | 24 | 58 | 0 | 24 |
| Path 984 | C00031->C00002:[4->10,4->11,5->17,7->13,7->3,7->5,9->12,9->2,9->8] | 0.90 | 0.0 | 32 | 170 | 0 | 32 |
| Path 985 | C00031->C00002:[4->10,4->11,5->13,7->17,7->3,7->5,9->12,9->2,9->8] | 0.90 | 0.0 | 30 | 65 | 0 | 30 |
| Path 986 | C00031->C00002:[4->10,4->11,5->13,7->17,7->3,7->5,9->12,9->2,9->8] | 0.90 | 0.0 | 34 | 172 | 0 | 34 |
| Path 987 | C00031->C00002:[4->10,4->11,5->13,7->17,7->3,9->12,9->8] | 0.70 | 0.0 | 26 | 64 | 0 | 26 |
| Path 988 | C00031->C00002:[4->10,4->11,5->17,7->13,7->3,9->12,9->8] | 0.70 | 0.0 | 27 | 162 | 0 | 27 |
| Path 989 | C00031->C00002:[5->13,7->17,7->3,9->12,9->8] | 0.50 | 0.0 | 14 | 37 | 0 | 14 |
| Path 990 | C00031->C00002:[4->10,4->11,5->13,7->17,7->3,9->12,9->8] | 0.70 | 0.0 | 25 | 63 | 0 | 25 |
| Path 991 | C00031->C00002:[4->10,4->11,5->13,7->17,7->3,7->5,9->12,9->2,9->8] | 0.90 | 0.0 | 34 | 176 | 0 | 34 |
| Path 992 | C00031->C00002:[5->17,7->13,7->3,9->12,9->8] | 0.50 | 0.0 | 17 | 53 | 0 | 17 |
| Path 993 | C00031->C00002:[1->12,2->13,4->10,4->11,4->17,7->3,9->8] | 0.70 | 0.0 | 23 | 55 | 0 | 23 |
| Path 994 | C00031->C00002:[4->11,5->13,7->17,7->3,9->12,9->8] | 0.60 | 0.0 | 22 | 151 | 0 | 22 |
| Path 995 | C00031->C00002:[5->13,7->17,7->3,9->12,9->8] | 0.50 | 0.0 | 20 | 144 | 0 | 20 |
| Path 996 | C00031->C00002:[4->10,4->11,5->13,7->17,7->3,7->5,9->12,9->2,9->8] | 0.90 | 0.0 | 32 | 171 | 0 | 32 |
| Path 997 | C00031->C00002:[4->11,5->13,7->17,7->3,9->12,9->8] | 0.60 | 0.0 | 25 | 160 | 0 | 25 |
| Path 998 | C00031->C00002:[4->11,5->13,7->17,7->3,9->12,9->8] | 0.60 | 0.0 | 22 | 50 | 0 | 22 |
| Path 999 | C00031->C00002:[1->12,2->13,4->10,4->11,4->17,4->4,5->8,7->4,7->5,9->2,9->3] | 1.00 | 0.0 | 32 | 159 | 0 | 32 |
| Path 1000 | C00031->C00002:[1->12,2->13,4->10,4->11,4->17,7->3,7->5,9->2,9->8] | 0.90 | 0.0 | 31 | 51 | 0 | 31 |
| Path 1001 | C00031->C00002:[1->12,2->13,4->10,4->11,4->17,7->3,7->5,9->2,9->8] | 0.90 | 0.0 | 31 | 62 | 0 | 31 |
| Path 1002 | C00031->C00002:[4->10,4->11,5->13,7->17,7->3,7->5,9->12,9->2,9->8] | 0.90 | 0.0 | 33 | 171 | 0 | 33 |
| Path 1003 | C00031->C00002:[1->12,2->13,4->17,5->3,5->8,7->11] | 0.60 | 0.0 | 20 | 140 | 0 | 20 |
| Path 1004 | C00031->C00002:[1->12,2->13,4->10,4->11,4->17,5->8,7->5,9->2,9->3] | 0.90 | 0.0 | 29 | 161 | 0 | 29 |
| Path 1005 | C00031->C00002:[1->12,2->13,4->10,4->11,4->17,5->8,9->3] | 0.70 | 0.0 | 19 | 143 | 0 | 19 |
| Path 1006 | C00031->C00002:[4->10,4->11,5->13,7->17,7->3,7->5,9->12,9->2,9->8] | 0.90 | 0.0 | 31 | 65 | 0 | 31 |
| Path 1007 | C00031->C00002:[1->12,2->13,4->17,5->8,9->10,9->3] | 0.60 | 0.0 | 20 | 149 | 0 | 20 |
| Path 1008 | C00031->C00002:[5->13,7->17,7->3,9->12,9->8] | 0.50 | 0.0 | 13 | 39 | 0 | 13 |
| Path 1009 | C00031->C00002:[5->17,7->13,7->3,9->12,9->8] | 0.50 | 0.0 | 18 | 155 | 0 | 18 |
| Path 1010 | C00031->C00002:[1->12,2->13,4->11,4->17,5->3,5->8] | 0.60 | 0.0 | 21 | 43 | 0 | 21 |
| Path 1011 | C00031->C00002:[1->12,2->13,4->17,5->3,5->8] | 0.50 | 0.0 | 16 | 355 | 0 | 16 |
| Path 1012 | C00031->C00002:[1->12,2->13,4->10,4->11,4->17,5->3,5->8,7->10,7->11] | 0.70 | 0.0 | 25 | 156 | 0 | 25 |
| Path 1013 | C00031->C00002:[4->11,5->13,7->17,7->3,9->12,9->8] | 0.60 | 0.0 | 21 | 52 | 0 | 21 |
| Path 1014 | C00031->C00002:[5->13,7->17,7->3,9->12,9->8] | 0.50 | 0.0 | 14 | 366 | 0 | 14 |
| Path 1015 | C00031->C00002:[5->13,7->17,7->3,9->12,9->8] | 0.50 | 0.0 | 15 | 146 | 0 | 15 |
| Path 1016 | C00031->C00002:[1->12,2->13,2->3,4->17,5->8] | 0.50 | 0.0 | 13 | 139 | 0 | 13 |
| Path 1017 | C00031->C00002:[4->10,4->11,5->17,7->13,7->3,7->5,9->12,9->2,9->8] | 0.90 | 0.0 | 35 | 183 | 0 | 35 |
| Path 1018 | C00031->C00002:[5->13,7->17,7->3,9->12,9->8] | 0.50 | 0.0 | 13 | 137 | 0 | 13 |
| Path 1019 | C00031->C00002:[4->10,4->11,5->13,7->17,7->3,9->12,9->8] | 0.70 | 0.0 | 27 | 56 | 0 | 27 |
| Path 1020 | C00031->C00002:[1->12,2->13,4->10,4->11,4->17,5->3,5->8,7->5,9->2] | 0.90 | 0.0 | 32 | 161 | 0 | 32 |
| Path 1021 | C00031->C00002:[1->12,2->13,4->10,4->11,4->17,4->4,5->8,7->2,9->3,9->5] | 1.00 | 0.0 | 32 | 173 | 0 | 32 |
| Path 1022 | C00031->C00002:[4->10,4->11,5->13,7->17,7->3,9->12,9->8] | 0.70 | 0.0 | 27 | 46 | 0 | 27 |
| Path 1023 | C00031->C00002:[5->13,7->17,7->3,9->12,9->8] | 0.50 | 0.0 | 16 | 142 | 0 | 16 |
| Path 1024 | C00031->C00002:[4->11,5->13,7->17,7->3,9->12,9->8] | 0.60 | 0.0 | 24 | 163 | 0 | 24 |
| Path 1025 | C00031->C00002:[2->8,4->10,4->11,5->13,5->3,7->17,9->12] | 0.70 | 0.0 | 25 | 151 | 0 | 25 |
| Path 1026 | C00031->C00002:[1->12,2->13,4->10,4->11,4->17,7->3,7->5,9->2,9->8] | 0.90 | 0.0 | 28 | 157 | 0 | 28 |
| Path 1027 | C00031->C00002:[5->13,7->17,7->3,9->12,9->8] | 0.50 | 0.0 | 13 | 140 | 0 | 13 |
| Path 1028 | C00031->C00002:[4->10,4->11,5->13,7->17,7->3,7->5,9->12,9->2,9->8] | 0.90 | 0.0 | 30 | 165 | 0 | 30 |
| Path 1029 | C00031->C00002:[4->10,4->11,5->13,7->17,7->3,9->12,9->8] | 0.70 | 0.0 | 26 | 165 | 0 | 26 |
| Path 1030 | C00031->C00002:[4->10,4->11,5->13,7->17,7->3,7->5,9->12,9->2,9->8] | 0.90 | 0.0 | 33 | 164 | 0 | 33 |
| Path 1031 | C00031->C00002:[2->8,4->11,5->13,5->3,7->17,9->12] | 0.60 | 0.0 | 23 | 366 | 0 | 23 |
| Path 1032 | C00031->C00002:[4->10,4->11,5->13,7->17,7->3,7->5,9->12,9->2,9->8] | 0.90 | 0.0 | 34 | 71 | 0 | 34 |
| Path 1033 | C00031->C00002:[5->13,7->17,7->3,9->12,9->8] | 0.50 | 0.0 | 16 | 138 | 0 | 16 |
| Path 1034 | C00031->C00002:[4->10,4->11,5->13,7->17,7->3,7->5,9->12,9->2,9->8] | 0.90 | 0.0 | 32 | 178 | 0 | 32 |
| Path 1035 | C00031->C00002:[1->12,2->13,4->11,4->17,5->3,5->8] | 0.60 | 0.0 | 21 | 151 | 0 | 21 |
| Path 1036 | C00031->C00002:[4->10,4->11,5->13,7->17,7->3,9->12,9->8] | 0.70 | 0.0 | 26 | 371 | 0 | 26 |
| Path 1037 | C00031->C00002:[4->10,4->11,5->13,7->17,7->3,7->5,9->12,9->2,9->8] | 0.90 | 0.0 | 32 | 167 | 0 | 32 |
| Path 1038 | C00031->C00002:[1->12,2->13,4->10,4->11,4->17,5->3,5->8,7->5,9->2] | 0.90 | 0.0 | 29 | 372 | 0 | 29 |
| Path 1039 | C00031->C00002:[5->13,7->17,7->3,9->12,9->8] | 0.50 | 0.0 | 15 | 31 | 0 | 15 |
| Path 1040 | C00031->C00002:[4->10,4->11,5->13,7->17,7->3,7->5,9->12,9->2,9->8] | 0.90 | 0.0 | 33 | 171 | 0 | 33 |
| Path 1041 | C00031->C00002:[4->10,4->11,5->13,5->3,5->8,7->17,9->12] | 0.70 | 0.0 | 26 | 159 | 0 | 26 |
| Path 1042 | C00031->C00002:[4->10,4->11,5->13,7->17,7->3,9->12,9->8] | 0.70 | 0.0 | 25 | 60 | 0 | 25 |
| Path 1043 | C00031->C00002:[4->11,5->17,7->13,7->3,9->12,9->8] | 0.60 | 0.0 | 24 | 157 | 0 | 24 |
| Path 1044 | C00031->C00002:[4->11,5->13,7->17,7->3,9->12,9->8] | 0.60 | 0.0 | 24 | 152 | 0 | 24 |
| Path 1045 | C00031->C00002:[4->10,4->11,5->13,7->17,7->3,7->5,9->12,9->2,9->8] | 0.90 | 0.0 | 32 | 167 | 0 | 32 |
| Path 1046 | C00031->C00002:[5->13,7->17,7->3,9->12,9->8] | 0.50 | 0.0 | 13 | 141 | 0 | 13 |
| Path 1047 | C00031->C00002:[7->11] | 0.10 | 0.0 | 10 | 131 | 0 | 10 |
| Path 1048 | C00031->C00002:[5->13,7->17,7->3,9->12,9->8] | 0.50 | 0.0 | 14 | 142 | 0 | 14 |
| Path 1049 | C00031->C00002:[4->10,4->11,5->17,7->13,7->3,9->12,9->8] | 0.70 | 0.0 | 27 | 158 | 0 | 27 |
| Path 1050 | C00031->C00002:[5->13,5->3,5->8,7->17,9->12] | 0.50 | 0.0 | 16 | 40 | 0 | 16 |
| Path 1051 | C00031->C00002:[1->12,2->13,4->11,4->17,5->3,5->8] | 0.60 | 0.0 | 20 | 364 | 0 | 20 |
| Path 1052 | C00031->C00002:[4->11,5->17,7->13,7->3,9->12,9->8] | 0.60 | 0.0 | 26 | 157 | 0 | 26 |
| Path 1053 | C00031->C00002:[5->13,7->17,7->3,9->12,9->8] | 0.50 | 0.0 | 14 | 142 | 0 | 14 |
| Path 1054 | C00031->C00002:[4->10,4->11,5->13,7->17,7->3,9->12,9->8] | 0.70 | 0.0 | 24 | 369 | 0 | 24 |
| Path 1055 | C00031->C00002:[5->3,7->11] | 0.20 | 0.0 | 19 | 142 | 0 | 19 |
| Path 1056 | C00031->C00002:[1->12,2->13,4->10,4->11,4->17,5->8,7->10,7->11,7->5,9->2,9->3] | 0.90 | 0.0 | 29 | 160 | 0 | 29 |
| Path 1057 | C00031->C00002:[1->12,2->13,4->10,4->11,4->17,7->3,7->5,9->2,9->8] | 0.90 | 0.0 | 28 | 45 | 0 | 28 |
| Path 1058 | C00031->C00002:[5->17,7->13,7->3,9->12,9->8] | 0.50 | 0.0 | 18 | 158 | 0 | 18 |
| Path 1059 | C00031->C00002:[1->12,2->13,4->10,4->11,4->17,7->3,7->5,9->2,9->8] | 0.90 | 0.0 | 31 | 160 | 0 | 31 |
| Path 1060 | C00031->C00002:[1->12,2->13,4->10,4->11,4->17,5->3,5->8,7->10,7->11,7->5,9->2] | 0.90 | 0.0 | 32 | 166 | 0 | 32 |
| Path 1061 | C00031->C00002:[4->11,5->13,7->17,7->3,9->12,9->8] | 0.60 | 0.0 | 25 | 55 | 0 | 25 |
| Path 1062 | C00031->C00002:[1->12,2->13,4->17,5->3,5->8,7->11] | 0.60 | 0.0 | 20 | 143 | 0 | 20 |
| Path 1063 | C00031->C00002:[5->13,7->17,7->3,9->12,9->8] | 0.50 | 0.0 | 16 | 142 | 0 | 16 |
| Path 1064 | C00031->C00002:[4->10,4->11,5->13,7->17,7->3,7->5,9->12,9->2,9->8] | 0.90 | 0.0 | 31 | 167 | 0 | 31 |
| Path 1065 | C00031->C00002:[1->12,2->13,4->17,5->8,7->10,9->3] | 0.60 | 0.0 | 19 | 146 | 0 | 19 |
| Path 1066 | C00031->C00002:[1->12,2->13,4->11,4->17,7->3,9->8] | 0.60 | 0.0 | 21 | 150 | 0 | 21 |
| Path 1067 | C00031->C00002:[1->12,2->13,4->10,4->11,4->17,4->4,5->8,7->10,7->11,7->4,9->3] | 0.80 | 0.0 | 26 | 154 | 0 | 26 |
| Path 1068 | C00031->C00002:[7->3,9->8] | 0.20 | 0.0 | 10 | 128 | 0 | 10 |
| Path 1069 | C00031->C00002:[4->10,4->11,5->13,7->17,7->3,7->5,9->12,9->2,9->8] | 0.90 | 0.0 | 31 | 163 | 0 | 31 |
| Path 1070 | C00031->C00002:[4->10,4->11,5->13,7->17,7->3,7->5,9->12,9->2,9->8] | 0.90 | 0.0 | 33 | 175 | 0 | 33 |
| Path 1071 | C00031->C00002:[5->13,7->17,7->3,9->12,9->8] | 0.50 | 0.0 | 14 | 37 | 0 | 14 |
| Path 1072 | C00031->C00002:[4->10,4->11,5->13,7->17,7->3,9->12,9->8] | 0.70 | 0.0 | 25 | 53 | 0 | 25 |
| Path 1073 | C00031->C00002:[4->10,4->11,5->13,7->17,7->3,7->5,9->12,9->2,9->8] | 0.90 | 0.0 | 30 | 50 | 0 | 30 |
| Path 1074 | C00031->C00002:[4->10,4->11,5->13,7->17,7->3,7->5,9->12,9->2,9->8] | 0.90 | 0.0 | 31 | 162 | 0 | 31 |
| Path 1075 | C00031->C00002:[4->10,4->11,5->13,7->17,7->3,7->5,9->12,9->2,9->8] | 0.90 | 0.0 | 30 | 61 | 0 | 30 |
| Path 1076 | C00031->C00002:[5->13,7->17,7->3,9->12,9->8] | 0.50 | 0.0 | 23 | 150 | 0 | 23 |
| Path 1077 | C00031->C00002:[5->17,7->13,7->3,9->12,9->8] | 0.50 | 0.0 | 17 | 147 | 0 | 17 |
| Path 1078 | C00031->C00002:[1->12,2->13,4->10,4->17,4->4,5->8,9->3] | 0.70 | 0.0 | 27 | 160 | 0 | 27 |
| Path 1079 | C00031->C00002:[7->3,9->8] | 0.20 | 0.0 | 9 | 26 | 0 | 9 |
| Path 1080 | C00031->C00002:[4->10,4->11,5->17,7->13,7->3,9->12,9->8] | 0.70 | 0.0 | 27 | 68 | 0 | 27 |
| Path 1081 | C00031->C00002:[4->10,4->11,5->13,7->17,7->3,7->5,9->12,9->2,9->8] | 0.90 | 0.0 | 35 | 394 | 0 | 35 |
| Path 1082 | C00031->C00002:[1->12,2->13,4->10,4->11,4->17,5->3,5->8] | 0.70 | 0.0 | 22 | 145 | 0 | 22 |
| Path 1083 | C00031->C00002:[1->12,2->13,4->17,5->8,7->11,9->3] | 0.60 | 0.0 | 14 | 132 | 0 | 14 |
| Path 1084 | C00031->C00002:[5->13,7->17,7->3,9->12,9->8] | 0.50 | 0.0 | 13 | 137 | 0 | 13 |
| Path 1085 | C00031->C00002:[4->11,5->13,7->17,7->3,9->12,9->8] | 0.60 | 0.0 | 25 | 157 | 0 | 25 |
| Path 1086 | C00031->C00002:[1->12,2->13,4->10,4->11,4->17,5->3,5->8,7->5,9->2] | 0.90 | 0.0 | 28 | 371 | 0 | 28 |
| Path 1087 | C00031->C00002:[4->10,4->11,5->13,7->17,7->3,9->12,9->8] | 0.70 | 0.0 | 26 | 71 | 0 | 26 |
| Path 1088 | C00031->C00002:[1->12,2->13,4->11,4->17,7->3,9->8] | 0.60 | 0.0 | 22 | 155 | 0 | 22 |
| Path 1089 | C00031->C00002:[4->11,5->17,7->13,7->3,9->12,9->8] | 0.60 | 0.0 | 27 | 63 | 0 | 27 |
| Path 1090 | C00031->C00002:[5->17,7->13,7->3,9->12,9->8] | 0.50 | 0.0 | 18 | 43 | 0 | 18 |
| Path 1091 | C00031->C00002:[4->10,4->11,5->13,7->17,7->3,7->5,9->12,9->2,9->8] | 0.90 | 0.0 | 31 | 383 | 0 | 31 |
| Path 1092 | C00031->C00002:[4->10,4->11,5->13,7->17,7->3,7->5,9->12,9->2,9->8] | 0.90 | 0.0 | 33 | 163 | 0 | 33 |
| Path 1093 | C00031->C00002:[4->11,5->13,5->3,5->8,7->17,9->12] | 0.60 | 0.0 | 24 | 157 | 0 | 24 |
| Path 1094 | C00031->C00002:[4->11,5->13,7->17,7->3,9->12,9->8] | 0.60 | 0.0 | 24 | 159 | 0 | 24 |
| Path 1095 | C00031->C00002:[4->10,4->11,5->17,7->13,7->3,7->5,9->12,9->2,9->8] | 0.90 | 0.0 | 35 | 71 | 0 | 35 |
| Path 1096 | C00031->C00002:[4->10,4->11,5->13,7->17,7->3,9->12,9->8] | 0.70 | 0.0 | 26 | 153 | 0 | 26 |
| Path 1097 | C00031->C00002:[5->17,7->13,7->3,9->12,9->8] | 0.50 | 0.0 | 17 | 39 | 0 | 17 |
| Path 1098 | C00031->C00002:[1->12,2->13,4->10,4->11,4->17,4->4,5->8,7->10,7->11,7->4,9->3] | 0.80 | 0.0 | 29 | 164 | 0 | 29 |
| Path 1099 | C00031->C00002:[1->12,2->13,4->11,4->17,5->3,5->8] | 0.60 | 0.0 | 22 | 147 | 0 | 22 |
| Path 1100 | C00031->C00002:[1->12,2->13,4->10,4->11,4->17,7->3,7->5,9->2,9->8] | 0.90 | 0.0 | 31 | 66 | 0 | 31 |
| Path 1101 | C00031->C00002:[1->12,2->13,4->10,4->11,4->17,5->3,5->8,7->5,9->2] | 0.90 | 0.0 | 32 | 59 | 0 | 32 |
| Path 1102 | C00031->C00002:[5->13,7->17,7->3,9->12,9->8] | 0.50 | 0.0 | 13 | 133 | 0 | 13 |
| Path 1103 | C00031->C00002:[1->12,2->13,4->11,4->17,5->3,5->8] | 0.60 | 0.0 | 23 | 47 | 0 | 23 |
| Path 1104 | C00031->C00002:[5->13,7->17,7->3,9->12,9->8] | 0.50 | 0.0 | 16 | 27 | 0 | 16 |
| Path 1105 | C00031->C00002:[1->12,2->13,4->17,5->8,7->11,9->3] | 0.60 | 0.0 | 18 | 138 | 0 | 18 |
| Path 1106 | C00031->C00002:[5->17,7->13,7->3,9->12,9->8] | 0.50 | 0.0 | 16 | 43 | 0 | 16 |
| Path 1107 | C00031->C00002:[4->10,4->11,5->13,7->17,7->3,9->12,9->8] | 0.70 | 0.0 | 25 | 63 | 0 | 25 |
| Path 1108 | C00031->C00002:[4->10,4->11,5->17,7->13,7->3,7->5,9->12,9->2,9->8] | 0.90 | 0.0 | 36 | 170 | 0 | 36 |
| Path 1109 | C00031->C00002:[1->12,2->13,4->10,4->11,4->17,7->3,9->8] | 0.70 | 0.0 | 22 | 50 | 0 | 22 |
| Path 1110 | C00031->C00002:[4->11,5->17,7->13,7->3,9->12,9->8] | 0.60 | 0.0 | 26 | 174 | 0 | 26 |
| Path 1111 | C00031->C00002:[4->10,4->11,5->17,7->13,7->3,9->12,9->8] | 0.70 | 0.0 | 26 | 161 | 0 | 26 |
| Path 1112 | C00031->C00002:[2->8,4->10,4->11,5->13,5->3,7->17,9->12] | 0.70 | 0.0 | 25 | 368 | 0 | 25 |
| Path 1113 | C00031->C00002:[4->10,4->11,5->13,7->17,7->3,7->5,9->12,9->2,9->8] | 0.90 | 0.0 | 33 | 380 | 0 | 33 |
| Path 1114 | C00031->C00002:[5->13,7->17,7->3,9->12,9->8] | 0.50 | 0.0 | 22 | 150 | 0 | 22 |
| Path 1115 | C00031->C00002:[5->13,7->17,7->3,9->12,9->8] | 0.50 | 0.0 | 16 | 364 | 0 | 16 |
| Path 1116 | C00031->C00002:[4->10,4->11,5->17,7->13,7->3,9->12,9->8] | 0.70 | 0.0 | 29 | 76 | 0 | 29 |
| Path 1117 | C00031->C00002:[4->10,4->11,5->13,7->17,7->3,9->12,9->8] | 0.70 | 0.0 | 24 | 159 | 0 | 24 |
| Path 1118 | C00031->C00002:[1->12,2->13,4->10,4->17,4->4,5->8,7->10,7->4,9->3] | 0.70 | 0.0 | 23 | 150 | 0 | 23 |
| Path 1119 | C00031->C00002:[4->10,4->11,5->13,7->17,7->3,9->12,9->8] | 0.70 | 0.0 | 24 | 160 | 0 | 24 |
| Path 1120 | C00031->C00002:[4->11,5->13,7->17,7->3,9->12,9->8] | 0.60 | 0.0 | 23 | 50 | 0 | 23 |
| Path 1121 | C00031->C00002:[5->17,7->13,7->3,9->12,9->8] | 0.50 | 0.0 | 18 | 155 | 0 | 18 |
| Path 1122 | C00031->C00002:[4->10,4->11,5->17,7->13,7->3,7->5,9->12,9->2,9->8] | 0.90 | 0.0 | 34 | 170 | 0 | 34 |
| Path 1123 | C00031->C00002:[1->12,2->13,4->10,4->11,4->17,5->3,5->8,7->5,9->2] | 0.90 | 0.0 | 30 | 160 | 0 | 30 |
| Path 1124 | C00031->C00002:[1->12,2->13,4->17,5->8,7->11,9->3] | 0.60 | 0.0 | 18 | 138 | 0 | 18 |
| Path 1125 | C00031->C00002:[4->10,4->11,5->13,7->17,7->3,7->5,9->12,9->2,9->8] | 0.90 | 0.0 | 32 | 73 | 0 | 32 |
| Path 1126 | C00031->C00002:[4->10,4->11,5->13,7->17,7->3,9->12,9->8] | 0.70 | 0.0 | 23 | 151 | 0 | 23 |
| Path 1127 | C00031->C00002:[5->13,7->17,7->3,9->12,9->8] | 0.50 | 0.0 | 15 | 142 | 0 | 15 |
| Path 1128 | C00031->C00002:[4->10,4->11,5->17,7->13,7->3,7->5,9->12,9->2,9->8] | 0.90 | 0.0 | 34 | 71 | 0 | 34 |
| Path 1129 | C00031->C00002:[1->12,2->13,4->17,7->11,7->3,9->8] | 0.60 | 0.0 | 21 | 149 | 0 | 21 |
| Path 1130 | C00031->C00002:[1->12,2->13,4->10,4->11,4->17,7->3,9->8] | 0.70 | 0.0 | 24 | 150 | 0 | 24 |
| Path 1131 | C00031->C00002:[4->10,4->11,5->13,7->17,7->3,7->5,9->12,9->2,9->8] | 0.90 | 0.0 | 31 | 166 | 0 | 31 |
| Path 1132 | C00031->C00002:[4->10,4->11,5->13,7->17,7->3,9->12,9->8] | 0.70 | 0.0 | 25 | 156 | 0 | 25 |
| Path 1133 | C00031->C00002:[4->11,5->17,7->13,7->3,9->12,9->8] | 0.60 | 0.0 | 25 | 158 | 0 | 25 |
| Path 1134 | C00031->C00002:[1->12,2->13,4->17,5->3,5->8,7->11] | 0.60 | 0.0 | 19 | 139 | 0 | 19 |
| Path 1135 | C00031->C00002:[4->10,4->11,5->13,7->17,7->3,9->12,9->8] | 0.70 | 0.0 | 26 | 60 | 0 | 26 |
| Path 1136 | C00031->C00002:[1->12,2->13,4->10,4->11,4->17,4->4,5->8,7->2,9->3,9->5] | 1.00 | 0.0 | 32 | 177 | 0 | 32 |
| Path 1137 | C00031->C00002:[1->12,2->13,4->10,4->11,4->17,4->4,5->8,7->2,9->3,9->5] | 1.00 | 0.0 | 32 | 174 | 0 | 32 |
| Path 1138 | C00031->C00002:[1->12,2->13,4->10,4->17,4->4,5->8,9->3] | 0.70 | 0.0 | 27 | 163 | 0 | 27 |
| Path 1139 | C00031->C00002:[1->12,2->13,4->17,5->8] | 0.40 | 0.0 | 9 | 128 | 0 | 9 |
| Path 1140 | C00031->C00002:[4->11,5->13,7->17,7->3,9->12,9->8] | 0.60 | 0.0 | 21 | 156 | 0 | 21 |
| Path 1141 | C00031->C00002:[4->10,4->11,5->13,7->17,7->3,9->12,9->8] | 0.70 | 0.0 | 27 | 72 | 0 | 27 |
| Path 1142 | C00031->C00002:[5->13,7->17,7->3,9->12,9->8] | 0.50 | 0.0 | 13 | 351 | 0 | 13 |
| Path 1143 | C00031->C00002:[1->12,2->13,4->10,4->11,4->17,4->4,5->8,7->5,9->2,9->3] | 1.00 | 0.0 | 32 | 165 | 0 | 32 |
| Path 1144 | C00031->C00002:[4->11,5->13,7->17,7->3,9->12,9->8] | 0.60 | 0.0 | 22 | 150 | 0 | 22 |
| Path 1145 | C00031->C00002:[4->11,5->13,7->17,7->3,9->12,9->8] | 0.60 | 0.0 | 24 | 57 | 0 | 24 |
| Path 1146 | C00031->C00002:[2->8,4->10,4->11,5->13,5->3,7->17,9->12] | 0.70 | 0.0 | 24 | 150 | 0 | 24 |
| Path 1147 | C00031->C00002:[4->10,4->11,5->13,7->17,7->3,9->12,9->8] | 0.70 | 0.0 | 26 | 60 | 0 | 26 |
| Path 1148 | C00031->C00002:[1->12,2->13,4->10,4->11,4->17,5->8,7->10,7->11,9->3] | 0.70 | 0.0 | 23 | 154 | 0 | 23 |
| Path 1149 | C00031->C00002:[1->12,2->13,4->17,7->3,9->8] | 0.50 | 0.0 | 13 | 138 | 0 | 13 |
| Path 1150 | C00031->C00002:[1->12,2->13,4->10,4->11,4->17,5->3,5->8] | 0.70 | 0.0 | 25 | 368 | 0 | 25 |
| Path 1151 | C00031->C00002:[4->10,4->11,5->13,7->17,7->3,9->12,9->8] | 0.70 | 0.0 | 25 | 160 | 0 | 25 |
| Path 1152 | C00031->C00002:[1->12,2->13,4->17,5->8,7->11,9->3] | 0.60 | 0.0 | 18 | 142 | 0 | 18 |
| Path 1153 | C00031->C00002:[4->10,4->11,5->13,7->17,7->3,7->5,9->12,9->2,9->8] | 0.90 | 0.0 | 32 | 51 | 0 | 32 |
| Path 1154 | C00031->C00002:[5->13,5->3,5->8,7->17,9->12] | 0.50 | 0.0 | 16 | 358 | 0 | 16 |
| Path 1155 | C00031->C00002:[1->12,2->13,4->10,4->17,4->4,5->3,5->8] | 0.70 | 0.0 | 25 | 158 | 0 | 25 |
| Path 1156 | C00031->C00002:[4->10,4->11,5->13,7->17,7->3,9->12,9->8] | 0.70 | 0.0 | 24 | 44 | 0 | 24 |
| Path 1157 | C00031->C00002:[5->13,7->17,7->3,9->12,9->8] | 0.50 | 0.0 | 19 | 140 | 0 | 19 |
| Path 1158 | C00031->C00002:[4->10,4->11,5->13,7->17,7->3,9->12,9->8] | 0.70 | 0.0 | 26 | 161 | 0 | 26 |
| Path 1159 | C00031->C00002:[4->11,5->17,7->13,7->3,9->12,9->8] | 0.60 | 0.0 | 27 | 172 | 0 | 27 |
| Path 1160 | C00031->C00002:[1->12,2->13,4->17,7->3,9->8] | 0.50 | 0.0 | 12 | 350 | 0 | 12 |
| Path 1161 | C00031->C00002:[5->13,7->17,7->3,9->12,9->8] | 0.50 | 0.0 | 14 | 135 | 0 | 14 |
| Path 1162 | C00031->C00002:[4->10,4->11,5->17,7->13,7->3,9->12,9->8] | 0.70 | 0.0 | 27 | 172 | 0 | 27 |
| Path 1163 | C00031->C00002:[4->10,4->11,5->17,7->13,7->3,7->5,9->12,9->2,9->8] | 0.90 | 0.0 | 33 | 169 | 0 | 33 |
| Path 1164 | C00031->C00002:[4->10,4->11,5->13,7->17,7->3,7->5,9->12,9->2,9->8] | 0.90 | 0.0 | 31 | 166 | 0 | 31 |
| Path 1165 | C00031->C00002:[1->12,2->13,4->11,4->17,5->3,5->8] | 0.60 | 0.0 | 25 | 157 | 0 | 25 |
| Path 1166 | C00031->C00002:[1->12,2->13,4->10,4->11,4->17,7->3,7->5,9->2,9->8] | 0.90 | 0.0 | 29 | 158 | 0 | 29 |
| Path 1167 | C00031->C00002:[1->12,2->13,4->10,4->11,4->17,7->3,9->8] | 0.70 | 0.0 | 22 | 148 | 0 | 22 |
| Path 1168 | C00031->C00002:[4->10,4->11,5->13,7->17,7->3,9->12,9->8] | 0.70 | 0.0 | 27 | 173 | 0 | 27 |
| Path 1169 | C00031->C00002:[4->10,4->11,5->13,7->17,7->3,7->5,9->12,9->2,9->8] | 0.90 | 0.0 | 33 | 171 | 0 | 33 |
| Path 1170 | C00031->C00002:[4->11,5->13,7->17,7->3,9->12,9->8] | 0.60 | 0.0 | 23 | 158 | 0 | 23 |
| Path 1171 | C00031->C00002:[4->10,4->11,5->13,7->17,7->3,9->12,9->8] | 0.70 | 0.0 | 25 | 160 | 0 | 25 |
| Path 1172 | C00031->C00002:[4->10,4->11,5->17,7->13,7->3,7->5,9->12,9->2,9->8] | 0.90 | 0.0 | 35 | 183 | 0 | 35 |
| Path 1173 | C00031->C00002:[1->12,2->13,4->10,4->11,4->17,5->3,5->8,7->5,9->2] | 0.90 | 0.0 | 33 | 380 | 0 | 33 |
| Path 1174 | C00031->C00002:[5->17,7->13,7->3,9->12,9->8] | 0.50 | 0.0 | 17 | 37 | 0 | 17 |
| Path 1175 | C00031->C00002:[4->11,5->13,7->17,7->3,9->12,9->8] | 0.60 | 0.0 | 22 | 53 | 0 | 22 |
| Path 1176 | C00031->C00002:[1->12,2->13,4->17,5->3,5->8,7->11] | 0.60 | 0.0 | 18 | 141 | 0 | 18 |
| Path 1177 | C00031->C00002:[4->11,5->13,7->17,7->3,9->12,9->8] | 0.60 | 0.0 | 24 | 152 | 0 | 24 |
| Path 1178 | C00031->C00002:[5->17,7->13,7->3,9->12,9->8] | 0.50 | 0.0 | 18 | 57 | 0 | 18 |
| Path 1179 | C00031->C00002:[1->12,2->13,4->17,5->3,5->8] | 0.50 | 0.0 | 11 | 33 | 0 | 11 |
| Path 1180 | C00031->C00002:[4->11,5->13,7->17,7->3,9->12,9->8] | 0.60 | 0.0 | 23 | 46 | 0 | 23 |
| Path 1181 | C00031->C00002:[1->12,2->13,4->17,5->8,9->11,9->3] | 0.60 | 0.0 | 18 | 145 | 0 | 18 |
| Path 1182 | C00031->C00002:[1->12,2->13,4->10,4->11,4->17,7->3,9->8] | 0.70 | 0.0 | 23 | 51 | 0 | 23 |
| Path 1183 | C00031->C00002:[4->10,4->11,5->13,7->17,7->3,7->5,9->12,9->2,9->8] | 0.90 | 0.0 | 32 | 167 | 0 | 32 |
| Path 1184 | C00031->C00002:[4->10,4->11,5->13,7->17,7->3,7->5,9->12,9->2,9->8] | 0.90 | 0.0 | 31 | 170 | 0 | 31 |
| Path 1185 | C00031->C00002:[1->12,2->13,4->10,4->11,4->17,5->8,7->10,7->11,9->3] | 0.70 | 0.0 | 20 | 145 | 0 | 20 |
| Path 1186 | C00031->C00002:[4->10,4->11,5->13,7->17,7->3,9->12,9->8] | 0.70 | 0.0 | 25 | 375 | 0 | 25 |
| Path 1187 | C00031->C00002:[5->13,7->17,7->3,9->12,9->8] | 0.50 | 0.0 | 15 | 44 | 0 | 15 |
| Path 1188 | C00031->C00002:[1->12,2->13,4->11,4->17,5->3,5->8] | 0.60 | 0.0 | 20 | 147 | 0 | 20 |
| Path 1189 | C00031->C00002:[4->10,4->11,5->17,7->13,7->3,9->12,9->8] | 0.70 | 0.0 | 28 | 159 | 0 | 28 |
| Path 1190 | C00031->C00002:[1->12,2->13,4->17,5->3,5->8] | 0.50 | 0.0 | 13 | 30 | 0 | 13 |
| Path 1191 | C00031->C00002:[5->3] | 0.10 | 0.0 | 9 | 341 | 0 | 9 |
| Path 1192 | C00031->C00002:[4->11,5->17,7->13,7->3,9->12,9->8] | 0.60 | 0.0 | 25 | 380 | 0 | 25 |
| Path 1193 | C00031->C00002:[4->11,5->13,7->17,7->3,9->12,9->8] | 0.60 | 0.0 | 24 | 369 | 0 | 24 |
| Path 1194 | C00031->C00002:[1->12,2->13,4->17,5->3,5->8] | 0.50 | 0.0 | 15 | 136 | 0 | 15 |
| Path 1195 | C00031->C00002:[1->12,2->13,4->10,4->17,5->8,9->3] | 0.60 | 0.0 | 21 | 150 | 0 | 21 |
| Path 1196 | C00031->C00002:[4->10,4->11,5->13,7->17,7->3,7->5,9->12,9->2,9->8] | 0.90 | 0.0 | 32 | 66 | 0 | 32 |
| Path 1197 | C00031->C00002:[1->12,2->13,4->17,5->3,5->8] | 0.50 | 0.0 | 12 | 131 | 0 | 12 |
| Path 1198 | C00031->C00002:[4->10,4->11,5->13,7->17,7->3,9->12,9->8] | 0.70 | 0.0 | 24 | 370 | 0 | 24 |
| Path 1199 | C00031->C00002:[4->11,5->13,7->17,7->3,9->12,9->8] | 0.60 | 0.0 | 25 | 59 | 0 | 25 |
| Path 1200 | C00031->C00002:[1->12,2->13,4->10,4->11,4->17,5->3,5->8,7->5,9->2] | 0.90 | 0.0 | 32 | 160 | 0 | 32 |
| Path 1201 | C00031->C00002:[1->12,2->13,4->10,4->11,4->17,7->3,9->8] | 0.70 | 0.0 | 24 | 153 | 0 | 24 |
| Path 1202 | C00031->C00002:[1->12,2->13,4->10,4->11,4->17,7->3,9->8] | 0.70 | 0.0 | 24 | 153 | 0 | 24 |
| Path 1203 | C00031->C00002:[5->17,7->13,7->3,9->12,9->8] | 0.50 | 0.0 | 16 | 45 | 0 | 16 |
| Path 1204 | C00031->C00002:[1->12,2->13,4->10,4->11,4->17,5->3,5->8,7->5,9->2] | 0.90 | 0.0 | 33 | 170 | 0 | 33 |
| Path 1205 | C00031->C00002:[4->10,4->11,5->17,7->13,7->3,9->12,9->8] | 0.70 | 0.0 | 27 | 64 | 0 | 27 |
| Path 1206 | C00031->C00002:[1->12,2->13,4->17,5->8,7->11] | 0.50 | 0.0 | 17 | 141 | 0 | 17 |
| Path 1207 | C00031->C00002:[1->12,2->13,4->17,5->3,5->8,7->11] | 0.60 | 0.0 | 19 | 141 | 0 | 19 |
| Path 1208 | C00031->C00002:[1->12,2->13,4->17,5->3,5->8,7->11] | 0.60 | 0.0 | 18 | 138 | 0 | 18 |
| Path 1209 | C00031->C00002:[5->3,7->11] | 0.20 | 0.0 | 17 | 136 | 0 | 17 |
| Path 1210 | C00031->C00002:[2->8,4->10,4->11,5->13,5->3,7->17,9->12] | 0.70 | 0.0 | 23 | 49 | 0 | 23 |
| Path 1211 | C00031->C00002:[4->11,5->17,7->13,7->3,9->12,9->8] | 0.60 | 0.0 | 26 | 374 | 0 | 26 |
| Path 1212 | C00031->C00002:[4->10,4->11,5->17,7->13,7->3,7->5,9->12,9->2,9->8] | 0.90 | 0.0 | 32 | 168 | 0 | 32 |
| Path 1213 | C00031->C00002:[4->10,4->11,5->13,7->17,7->3,9->12,9->8] | 0.70 | 0.0 | 27 | 371 | 0 | 27 |
| Path 1214 | C00031->C00002:[4->10,4->11,5->13,7->17,7->3,9->12,9->8] | 0.70 | 0.0 | 25 | 160 | 0 | 25 |
| Path 1215 | C00031->C00002:[5->17,7->13,7->3,9->12,9->8] | 0.50 | 0.0 | 17 | 42 | 0 | 17 |
| Path 1216 | C00031->C00002:[5->13,7->17,7->3,9->12,9->8] | 0.50 | 0.0 | 15 | 352 | 0 | 15 |
| Path 1217 | C00031->C00002:[4->11,5->17,7->13,7->3,9->12,9->8] | 0.60 | 0.0 | 26 | 157 | 0 | 26 |
| Path 1218 | C00031->C00002:[1->12,2->13,4->11,4->17,5->3,5->8] | 0.60 | 0.0 | 21 | 147 | 0 | 21 |
| Path 1219 | C00031->C00002:[4->11,5->13,7->17,7->3,9->12,9->8] | 0.60 | 0.0 | 24 | 167 | 0 | 24 |
| Path 1220 | C00031->C00002:[4->11,5->13,7->17,7->3,9->12,9->8] | 0.60 | 0.0 | 24 | 54 | 0 | 24 |
| Path 1221 | C00031->C00002:[5->13,7->17,7->3,9->12,9->8] | 0.50 | 0.0 | 13 | 22 | 0 | 13 |
| Path 1222 | C00031->C00002:[4->10,4->11,5->13,7->17,7->3,9->12,9->8] | 0.70 | 0.0 | 27 | 162 | 0 | 27 |
| Path 1223 | C00031->C00002:[4->11,5->13,7->17,7->3,9->12,9->8] | 0.60 | 0.0 | 21 | 149 | 0 | 21 |
| Path 1224 | C00031->C00002:[4->10,4->11,5->13,7->17,7->3,7->5,9->12,9->2,9->8] | 0.90 | 0.0 | 34 | 164 | 0 | 34 |
| Path 1225 | C00031->C00002:[1->12,2->13,4->10,4->11,4->17,5->3,5->8] | 0.70 | 0.0 | 22 | 362 | 0 | 22 |
| Path 1226 | C00031->C00002:[5->3,7->11] | 0.20 | 0.0 | 19 | 142 | 0 | 19 |
| Path 1227 | C00031->C00002:[5->13,7->17,7->3,9->12,9->8] | 0.50 | 0.0 | 16 | 140 | 0 | 16 |
| Path 1228 | C00031->C00002:[5->13,7->17,7->3,9->12,9->8] | 0.50 | 0.0 | 14 | 40 | 0 | 14 |
| Path 1229 | C00031->C00002:[1->12,2->13,4->17,7->3,9->8] | 0.50 | 0.0 | 13 | 131 | 0 | 13 |
| Path 1230 | C00031->C00002:[5->13,7->17,7->3,9->12,9->8] | 0.50 | 0.0 | 16 | 359 | 0 | 16 |
| Path 1231 | C00031->C00002:[1->12,2->13,4->10,4->11,4->17,7->3,7->5,9->2,9->8] | 0.90 | 0.0 | 30 | 376 | 0 | 30 |
| Path 1232 | C00031->C00002:[1->12,2->13,4->10,4->11,4->17,7->3,7->5,9->2,9->8] | 0.90 | 0.0 | 29 | 158 | 0 | 29 |
| Path 1233 | C00031->C00002:[1->12,2->13,4->17,7->3,9->8] | 0.50 | 0.0 | 22 | 150 | 0 | 22 |
| Path 1234 | C00031->C00002:[4->11,5->13,7->17,7->3,9->12,9->8] | 0.60 | 0.0 | 25 | 48 | 0 | 25 |
| Path 1235 | C00031->C00002:[4->10,4->11,5->17,7->13,7->3,9->12,9->8] | 0.70 | 0.0 | 28 | 163 | 0 | 28 |
| Path 1236 | C00031->C00002:[1->12,2->13,4->10,4->11,4->17,7->3,7->5,9->2,9->8] | 0.90 | 0.0 | 29 | 57 | 0 | 29 |
| Path 1237 | C00031->C00002:[1->12,2->13,4->11,4->17,5->8,9->3] | 0.60 | 0.0 | 17 | 142 | 0 | 17 |
| Path 1238 | C00031->C00002:[4->11,5->17,7->13,7->3,9->12,9->8] | 0.60 | 0.0 | 25 | 47 | 0 | 25 |
| Path 1239 | C00031->C00002:[4->10,4->11,5->17,7->13,7->3,7->5,9->12,9->2,9->8] | 0.90 | 0.0 | 32 | 170 | 0 | 32 |
| Path 1240 | C00031->C00002:[4->11,5->17,7->13,7->3,9->12,9->8] | 0.60 | 0.0 | 26 | 377 | 0 | 26 |
| Path 1241 | C00031->C00002:[4->11,5->17,7->13,7->3,9->12,9->8] | 0.60 | 0.0 | 24 | 59 | 0 | 24 |
| Path 1242 | C00031->C00002:[4->10,4->11,5->13,7->17,7->3,7->5,9->12,9->2,9->8] | 0.90 | 0.0 | 33 | 389 | 0 | 33 |
| Path 1243 | C00031->C00002:[4->11,5->17,7->13,7->3,9->12,9->8] | 0.60 | 0.0 | 25 | 158 | 0 | 25 |
| Path 1244 | C00031->C00002:[4->10,4->11,5->13,7->17,7->3,9->12,9->8] | 0.70 | 0.0 | 26 | 53 | 0 | 26 |
| Path 1245 | C00031->C00002:[4->10,4->11,5->13,7->17,7->3,7->5,9->12,9->2,9->8] | 0.90 | 0.0 | 30 | 60 | 0 | 30 |
| Path 1246 | C00031->C00002:[1->12,2->13,2->3,4->10,4->11,4->17,5->8] | 0.70 | 0.0 | 24 | 153 | 0 | 24 |
| Path 1247 | C00031->C00002:[5->13,7->17,7->3,9->12,9->8] | 0.50 | 0.0 | 14 | 354 | 0 | 14 |
| Path 1248 | C00031->C00002:[1->12,2->13,4->17,5->3,5->8,7->11] | 0.60 | 0.0 | 21 | 144 | 0 | 21 |
| Path 1249 | C00031->C00002:[4->10,4->11,5->17,7->13,7->3,7->5,9->12,9->2,9->8] | 0.90 | 0.0 | 34 | 76 | 0 | 34 |
| Path 1250 | C00031->C00002:[4->11,5->13,7->17,7->3,9->12,9->8] | 0.60 | 0.0 | 21 | 49 | 0 | 21 |
| Path 1251 | C00031->C00002:[4->10,4->11,5->17,7->13,7->3,9->12,9->8] | 0.70 | 0.0 | 26 | 61 | 0 | 26 |
| Path 1252 | C00031->C00002:[1->12,2->13,4->10,4->11,4->17,5->3,5->8] | 0.70 | 0.0 | 23 | 45 | 0 | 23 |
| Path 1253 | C00031->C00002:[4->10,4->11,5->17,7->13,7->3,9->12,9->8] | 0.70 | 0.0 | 27 | 161 | 0 | 27 |
| Path 1254 | C00031->C00002:[1->12,2->13,4->10,4->11,4->17,7->3,9->8] | 0.70 | 0.0 | 22 | 148 | 0 | 22 |
| Path 1255 | C00031->C00002:[1->12,2->13,4->10,4->11,4->17,5->3,5->8] | 0.70 | 0.0 | 22 | 149 | 0 | 22 |
| Path 1256 | C00031->C00002:[4->10,4->11,5->13,7->17,7->3,9->12,9->8] | 0.70 | 0.0 | 23 | 50 | 0 | 23 |
| Path 1257 | C00031->C00002:[4->10,4->11,5->13,7->17,7->3,9->12,9->8] | 0.70 | 0.0 | 26 | 71 | 0 | 26 |
| Path 1258 | C00031->C00002:[4->10,4->11,5->13,5->3,5->8,7->17,9->12] | 0.70 | 0.0 | 27 | 160 | 0 | 27 |
| Path 1259 | C00031->C00002:[4->10,4->11,5->13,7->17,7->3,7->5,9->12,9->2,9->8] | 0.90 | 0.0 | 34 | 165 | 0 | 34 |
| Path 1260 | C00031->C00002:[5->13,7->17,7->3,9->12,9->8] | 0.50 | 0.0 | 16 | 147 | 0 | 16 |
| Path 1261 | C00031->C00002:[4->10,4->11,5->17,7->13,7->3,9->12,9->8] | 0.70 | 0.0 | 27 | 162 | 0 | 27 |
| Path 1262 | C00031->C00002:[4->11,5->13,7->17,7->3,9->12,9->8] | 0.60 | 0.0 | 25 | 160 | 0 | 25 |
| Path 1263 | C00031->C00002:[1->12,2->13,4->10,4->11,4->17,7->3,7->5,9->2,9->8] | 0.90 | 0.0 | 30 | 166 | 0 | 30 |
| Path 1264 | C00031->C00002:[1->12,2->13,4->11,4->17,7->3,9->8] | 0.60 | 0.0 | 21 | 53 | 0 | 21 |
| Path 1265 | C00031->C00002:[4->11,5->13,7->17,7->3,9->12,9->8] | 0.60 | 0.0 | 25 | 54 | 0 | 25 |
| Path 1266 | C00031->C00002:[5->13,7->17,7->3,9->12,9->8] | 0.50 | 0.0 | 15 | 134 | 0 | 15 |
| Path 1267 | C00031->C00002:[1->12,2->13,4->10,4->11,4->17,4->4,5->8,9->3] | 0.80 | 0.0 | 25 | 156 | 0 | 25 |
| Path 1268 | C00031->C00002:[1->12,2->13,4->10,4->11,4->17,4->4,5->8,7->5,9->2,9->3] | 1.00 | 0.0 | 32 | 165 | 0 | 32 |
| Path 1269 | C00031->C00002:[1->12,2->13,4->17,7->3,9->8] | 0.50 | 0.0 | 12 | 36 | 0 | 12 |
| Path 1270 | C00031->C00002:[5->13,7->17,7->3,9->12,9->8] | 0.50 | 0.0 | 19 | 143 | 0 | 19 |
| Path 1271 | C00031->C00002:[1->12,2->13,4->17,5->8,7->10,9->3] | 0.60 | 0.0 | 19 | 146 | 0 | 19 |
| Path 1272 | C00031->C00002:[1->12,2->13,4->17,7->3,9->8] | 0.50 | 0.0 | 13 | 138 | 0 | 13 |
| Path 1273 | C00031->C00002:[4->10,4->11,5->13,7->17,7->3,9->12,9->8] | 0.70 | 0.0 | 26 | 374 | 0 | 26 |
| Path 1274 | C00031->C00002:[1->12,2->13,4->17,5->3,5->8,7->11] | 0.60 | 0.0 | 21 | 144 | 0 | 21 |
| Path 1275 | C00031->C00002:[1->12,2->13,4->11,4->17,5->3,5->8] | 0.60 | 0.0 | 19 | 359 | 0 | 19 |
| Path 1276 | C00031->C00002:[1->12,2->13,4->10,4->11,4->17,5->3,5->8,7->5,9->2] | 0.90 | 0.0 | 28 | 372 | 0 | 28 |
| Path 1277 | C00031->C00002:[4->10,4->11,5->13,7->17,7->3,7->5,9->12,9->2,9->8] | 0.90 | 0.0 | 30 | 60 | 0 | 30 |
| Path 1278 | C00031->C00002:[5->17,7->13,7->3,9->12,9->8] | 0.50 | 0.0 | 16 | 144 | 0 | 16 |
| Path 1279 | C00031->C00002:[4->10,4->11,5->17,7->13,7->3,9->12,9->8] | 0.70 | 0.0 | 27 | 161 | 0 | 27 |
| Path 1280 | C00031->C00002:[4->10,4->11,5->17,7->13,7->3,9->12,9->8] | 0.70 | 0.0 | 27 | 55 | 0 | 27 |
| Path 1281 | C00031->C00002:[1->12,2->13,4->10,4->11,4->17,7->3,9->8] | 0.70 | 0.0 | 21 | 147 | 0 | 21 |
| Path 1282 | C00031->C00002:[1->12,2->13,4->17,7->3,9->8] | 0.50 | 0.0 | 12 | 21 | 0 | 12 |
| Path 1283 | C00031->C00002:[5->13,7->17,7->3,9->12,9->8] | 0.50 | 0.0 | 15 | 138 | 0 | 15 |
| Path 1284 | C00031->C00002:[1->12,2->13,4->17,5->3,5->8,7->11] | 0.60 | 0.0 | 22 | 145 | 0 | 22 |
| Path 1285 | C00031->C00002:[1->12,2->13,4->11,4->17,5->8,9->3] | 0.60 | 0.0 | 17 | 143 | 0 | 17 |
| Path 1286 | C00031->C00002:[5->13,7->17,7->3,9->12,9->8] | 0.50 | 0.0 | 16 | 356 | 0 | 16 |
| Path 1287 | C00031->C00002:[4->10,4->11,5->13,7->17,7->3,7->5,9->12,9->2,9->8] | 0.90 | 0.0 | 31 | 162 | 0 | 31 |
| Path 1288 | C00031->C00002:[1->12,2->13,4->10,4->11,4->17,5->3,5->8,7->5,9->2] | 0.90 | 0.0 | 30 | 63 | 0 | 30 |
| Path 1289 | C00031->C00002:[4->11,5->13,7->17,7->3,9->12,9->8] | 0.60 | 0.0 | 22 | 154 | 0 | 22 |
| Path 1290 | C00031->C00002:[1->12,2->13,4->10,4->11,4->17,5->8,9->3] | 0.70 | 0.0 | 19 | 144 | 0 | 19 |
| Path 1291 | C00031->C00002:[4->10,4->11,5->13,7->17,7->3,9->12,9->8] | 0.70 | 0.0 | 26 | 168 | 0 | 26 |
| Path 1292 | C00031->C00002:[1->12,2->13,4->11,4->17,5->3,5->8] | 0.60 | 0.0 | 20 | 151 | 0 | 20 |
| Path 1293 | C00031->C00002:[4->10,4->11,5->13,7->17,7->3,7->5,9->12,9->2,9->8] | 0.90 | 0.0 | 31 | 50 | 0 | 31 |
| Path 1294 | C00031->C00002:[4->10,4->11,5->13,7->17,7->3,9->12,9->8] | 0.70 | 0.0 | 27 | 157 | 0 | 27 |
| Path 1295 | C00031->C00002:[4->10,4->11,5->13,7->17,7->3,7->5,9->12,9->2,9->8] | 0.90 | 0.0 | 33 | 56 | 0 | 33 |
| Path 1296 | C00031->C00002:[1->12,2->13,4->11,4->17,5->8,9->3] | 0.60 | 0.0 | 17 | 139 | 0 | 17 |
| Path 1297 | C00031->C00002:[4->10,4->11,5->17,7->13,7->3,7->5,9->12,9->2,9->8] | 0.90 | 0.0 | 33 | 169 | 0 | 33 |
| Path 1298 | C00031->C00002:[1->12,2->13,4->10,4->11,4->17,7->3,7->5,9->2,9->8] | 0.90 | 0.0 | 31 | 160 | 0 | 31 |
| Path 1299 | C00031->C00002:[4->10,4->11,5->13,7->17,7->3,7->5,9->12,9->2,9->8] | 0.90 | 0.0 | 30 | 165 | 0 | 30 |
| Path 1300 | C00031->C00002:[1->12,2->13,4->17,5->3,5->8] | 0.50 | 0.0 | 12 | 352 | 0 | 12 |
| Path 1301 | C00031->C00002:[5->13,7->17,7->3,9->12,9->8] | 0.50 | 0.0 | 14 | 355 | 0 | 14 |
| Path 1302 | C00031->C00002:[2->8,4->10,4->11,5->13,5->3,7->17,9->12] | 0.70 | 0.0 | 24 | 50 | 0 | 24 |
| Path 1303 | C00031->C00002:[4->10,4->11,5->13,7->17,7->3,9->12,9->8] | 0.70 | 0.0 | 25 | 156 | 0 | 25 |
| Path 1304 | C00031->C00002:[4->10,4->11,5->13,7->17,7->3,7->5,9->12,9->2,9->8] | 0.90 | 0.0 | 32 | 162 | 0 | 32 |
| Path 1305 | C00031->C00002:[5->13,7->17,7->3,9->12,9->8] | 0.50 | 0.0 | 14 | 139 | 0 | 14 |
| Path 1306 | C00031->C00002:[4->10,4->11,5->17,7->13,7->3,7->5,9->12,9->2,9->8] | 0.90 | 0.0 | 33 | 171 | 0 | 33 |
| Path 1307 | C00031->C00002:[4->10,4->11,5->13,7->17,7->3,9->12,9->8] | 0.70 | 0.0 | 25 | 378 | 0 | 25 |
| Path 1308 | C00031->C00002:[4->10,4->11,5->13,7->17,7->3,9->12,9->8] | 0.70 | 0.0 | 24 | 167 | 0 | 24 |
| Path 1309 | C00031->C00002:[1->12,2->13,4->10,4->11,4->17,5->8,7->10,7->11,7->5,9->2,9->3] | 0.90 | 0.0 | 28 | 159 | 0 | 28 |
| Path 1310 | C00031->C00002:[4->10,4->11,5->13,7->17,7->3,9->12,9->8] | 0.70 | 0.0 | 24 | 58 | 0 | 24 |
| Path 1311 | C00031->C00002:[4->11,5->13,7->17,7->3,9->12,9->8] | 0.60 | 0.0 | 25 | 164 | 0 | 25 |
| Path 1312 | C00031->C00002:[4->10,4->11,5->17,7->13,7->3,7->5,9->12,9->2,9->8] | 0.90 | 0.0 | 36 | 184 | 0 | 36 |
| Path 1313 | C00031->C00002:[1->12,2->13,4->10,4->11,4->17,5->3,5->8,7->5,9->2] | 0.90 | 0.0 | 33 | 69 | 0 | 33 |
| Path 1314 | C00031->C00002:[1->12,2->13,2->3,4->10,4->11,4->17,5->8] | 0.70 | 0.0 | 24 | 154 | 0 | 24 |
| Path 1315 | C00031->C00002:[5->13,7->17,7->3,9->12,9->8] | 0.50 | 0.0 | 16 | 151 | 0 | 16 |
| Path 1316 | C00031->C00002:[7->11,7->3,9->8] | 0.30 | 0.0 | 17 | 139 | 0 | 17 |
| Path 1317 | C00031->C00002:[4->10,4->11,5->13,7->17,7->3,7->5,9->12,9->2,9->8] | 0.90 | 0.0 | 30 | 161 | 0 | 30 |
| Path 1318 | C00031->C00002:[5->17,7->13,7->3,9->12,9->8] | 0.50 | 0.0 | 18 | 57 | 0 | 18 |
| Path 1319 | C00031->C00002:[4->11,5->13,7->17,7->3,9->12,9->8] | 0.60 | 0.0 | 21 | 160 | 0 | 21 |
| Path 1320 | C00031->C00002:[2->8,4->11,5->13,5->3,7->17,9->12] | 0.60 | 0.0 | 22 | 48 | 0 | 22 |
| Path 1321 | C00031->C00002:[1->12,2->13,4->11,4->17,5->3,5->8] | 0.60 | 0.0 | 20 | 147 | 0 | 20 |
| Path 1322 | C00031->C00002:[1->12,2->13,4->10,4->11,4->17,7->3,9->8] | 0.70 | 0.0 | 23 | 156 | 0 | 23 |
| Path 1323 | C00031->C00002:[5->13,7->17,7->3,9->12,9->8] | 0.50 | 0.0 | 14 | 145 | 0 | 14 |
| Path 1324 | C00031->C00002:[4->11,5->13,7->17,7->3,9->12,9->8] | 0.60 | 0.0 | 22 | 52 | 0 | 22 |
| Path 1325 | C00031->C00002:[4->11,5->13,7->17,7->3,9->12,9->8] | 0.60 | 0.0 | 24 | 166 | 0 | 24 |
| Path 1326 | C00031->C00002:[1->12,2->13,4->10,4->11,4->17,5->3,5->8,7->5,9->2] | 0.90 | 0.0 | 31 | 165 | 0 | 31 |
| Path 1327 | C00031->C00002:[7->3,9->8] | 0.20 | 0.0 | 10 | 131 | 0 | 10 |
| Path 1328 | C00031->C00002:[4->11,5->17,7->13,7->3,9->12,9->8] | 0.60 | 0.0 | 25 | 158 | 0 | 25 |
| Path 1329 | C00031->C00002:[4->10,4->11,5->13,7->17,7->3,9->12,9->8] | 0.70 | 0.0 | 26 | 156 | 0 | 26 |
| Path 1330 | C00031->C00002:[4->11,5->13,7->17,7->3,9->12,9->8] | 0.60 | 0.0 | 24 | 54 | 0 | 24 |
| Path 1331 | C00031->C00002:[1->12,2->13,4->10,4->11,4->17,5->3,5->8] | 0.70 | 0.0 | 26 | 62 | 0 | 26 |
| Path 1332 | C00031->C00002:[1->12,2->13,4->10,4->11,4->17,7->3,9->8] | 0.70 | 0.0 | 24 | 52 | 0 | 24 |
| Path 1333 | C00031->C00002:[4->11,5->13,7->17,7->3,9->12,9->8] | 0.60 | 0.0 | 23 | 158 | 0 | 23 |
| Path 1334 | C00031->C00002:[4->11,5->13,7->17,7->3,9->12,9->8] | 0.60 | 0.0 | 23 | 57 | 0 | 23 |
| Path 1335 | C00031->C00002:[1->12,2->13,4->10,4->11,4->17,7->3,7->5,9->2,9->8] | 0.90 | 0.0 | 32 | 63 | 0 | 32 |
| Path 1336 | C00031->C00002:[5->13,7->17,7->3,9->12,9->8] | 0.50 | 0.0 | 15 | 142 | 0 | 15 |
| Path 1337 | C00031->C00002:[5->13,7->17,7->3,9->12,9->8] | 0.50 | 0.0 | 16 | 139 | 0 | 16 |
| Path 1338 | C00031->C00002:[4->10,4->11,5->17,7->13,7->3,7->5,9->12,9->2,9->8] | 0.90 | 0.0 | 32 | 69 | 0 | 32 |
| Path 1339 | C00031->C00002:[5->13,7->17,7->3,9->12,9->8] | 0.50 | 0.0 | 16 | 34 | 0 | 16 |
| Path 1340 | C00031->C00002:[4->10,4->11,5->17,7->13,7->3,9->12,9->8] | 0.70 | 0.0 | 28 | 159 | 0 | 28 |
| Path 1341 | C00031->C00002:[4->10,4->11,5->13,7->17,7->3,7->5,9->12,9->2,9->8] | 0.90 | 0.0 | 32 | 163 | 0 | 32 |
| Path 1342 | C00031->C00002:[5->13,7->17,7->3,9->12,9->8] | 0.50 | 0.0 | 16 | 135 | 0 | 16 |
| Path 1343 | C00031->C00002:[4->10,4->11,5->17,7->13,7->3,9->12,9->8] | 0.70 | 0.0 | 29 | 174 | 0 | 29 |
| Path 1344 | C00031->C00002:[4->10,4->11,5->13,7->17,7->3,9->12,9->8] | 0.70 | 0.0 | 24 | 55 | 0 | 24 |
| Path 1345 | C00031->C00002:[5->17,7->13,7->3,9->12,9->8] | 0.50 | 0.0 | 17 | 147 | 0 | 17 |
| Path 1346 | C00031->C00002:[4->11,5->13,7->17,7->3,9->12,9->8] | 0.60 | 0.0 | 23 | 61 | 0 | 23 |
| Path 1347 | C00031->C00002:[4->11,5->17,7->13,7->3,9->12,9->8] | 0.60 | 0.0 | 27 | 172 | 0 | 27 |
| Path 1348 | C00031->C00002:[1->12,2->13,4->17,7->3,9->8] | 0.50 | 0.0 | 12 | 133 | 0 | 12 |
| Path 1349 | C00031->C00002:[1->12,2->13,4->17,5->3,5->8] | 0.50 | 0.0 | 11 | 130 | 0 | 11 |
| Path 1350 | C00031->C00002:[1->12,2->13,4->10,4->11,4->17,5->8,7->5,9->2,9->3] | 0.90 | 0.0 | 29 | 158 | 0 | 29 |
| Path 1351 | C00031->C00002:[4->10,4->11,5->13,7->17,7->3,9->12,9->8] | 0.70 | 0.0 | 23 | 155 | 0 | 23 |
| Path 1352 | C00031->C00002:[1->12,2->13,4->17,5->3,5->8,7->11] | 0.60 | 0.0 | 21 | 144 | 0 | 21 |
| Path 1353 | C00031->C00002:[4->10,4->11,5->13,7->17,7->3,9->12,9->8] | 0.70 | 0.0 | 24 | 50 | 0 | 24 |
| Path 1354 | C00031->C00002:[5->13,7->17,7->3,9->12,9->8] | 0.50 | 0.0 | 13 | 36 | 0 | 13 |
| Path 1355 | C00031->C00002:[4->11,5->13,7->17,7->3,9->12,9->8] | 0.60 | 0.0 | 22 | 158 | 0 | 22 |
| Path 1356 | C00031->C00002:[4->10,4->11,5->17,7->13,7->3,7->5,9->12,9->2,9->8] | 0.90 | 0.0 | 36 | 170 | 0 | 36 |
| Path 1357 | C00031->C00002:[4->11,5->13,7->17,7->3,9->12,9->8] | 0.60 | 0.0 | 21 | 48 | 0 | 21 |
| Path 1358 | C00031->C00002:[1->12,2->13,4->17,5->3,5->8,7->11] | 0.60 | 0.0 | 21 | 147 | 0 | 21 |
| Path 1359 | C00031->C00002:[4->11,5->13,7->17,7->3,9->12,9->8] | 0.60 | 0.0 | 24 | 69 | 0 | 24 |
| Path 1360 | C00031->C00002:[1->12,2->13,4->17,5->3,5->8] | 0.50 | 0.0 | 15 | 349 | 0 | 15 |
| Path 1361 | C00031->C00002:[4->11,5->13,7->17,7->3,9->12,9->8] | 0.60 | 0.0 | 23 | 158 | 0 | 23 |
| Path 1362 | C00031->C00002:[1->12,2->13,4->10,4->11,4->17,5->3,5->8,7->5,9->2] | 0.90 | 0.0 | 30 | 55 | 0 | 30 |
| Path 1363 | C00031->C00002:[5->13,7->17,7->3,9->12,9->8] | 0.50 | 0.0 | 15 | 142 | 0 | 15 |
| Path 1364 | C00031->C00002:[4->10,4->11,5->13,7->17,7->3,9->12,9->8] | 0.70 | 0.0 | 27 | 61 | 0 | 27 |
| Path 1365 | C00031->C00002:[1->8,4->3,5->13,7->17,9->12] | 0.50 | 0.0 | 19 | 143 | 0 | 19 |
| Path 1366 | C00031->C00002:[1->8,4->3,5->13,7->17,9->12] | 0.50 | 0.0 | 19 | 143 | 0 | 19 |
| Path 1367 | C00031->C00002:[4->10,4->11,5->13,7->17,7->3,7->5,9->12,9->2,9->8] | 0.90 | 0.0 | 32 | 66 | 0 | 32 |
| Path 1368 | C00031->C00002:[1->12,2->13,4->17,5->3,5->8] | 0.50 | 0.0 | 14 | 348 | 0 | 14 |
| Path 1369 | C00031->C00002:[4->11,5->13,7->17,7->3,9->12,9->8] | 0.60 | 0.0 | 21 | 48 | 0 | 21 |
| Path 1370 | C00031->C00002:[4->11,5->13,7->17,7->3,9->12,9->8] | 0.60 | 0.0 | 25 | 377 | 0 | 25 |
| Path 1371 | C00031->C00002:[4->11,5->17,7->13,7->3,9->12,9->8] | 0.60 | 0.0 | 26 | 166 | 0 | 26 |
| Path 1372 | C00031->C00002:[4->10,4->11,5->13,7->17,7->3,9->12,9->8] | 0.70 | 0.0 | 26 | 383 | 0 | 26 |
| Path 1373 | C00031->C00002:[5->13,7->17,7->3,9->12,9->8] | 0.50 | 0.0 | 12 | 31 | 0 | 12 |
| Path 1374 | C00031->C00002:[1->12,2->13,4->10,4->11,4->17,5->3,5->8,7->5,9->2] | 0.90 | 0.0 | 28 | 154 | 0 | 28 |
| Path 1375 | C00031->C00002:[1->12,2->13,4->17,5->3,5->8,7->11] | 0.60 | 0.0 | 20 | 143 | 0 | 20 |
| Path 1376 | C00031->C00002:[5->13,7->17,7->3,9->12,9->8] | 0.50 | 0.0 | 14 | 30 | 0 | 14 |
| Path 1377 | C00031->C00002:[5->13,7->17,7->3,9->12,9->8] | 0.50 | 0.0 | 12 | 143 | 0 | 12 |
| Path 1378 | C00031->C00002:[4->10,4->11,5->13,7->17,7->3,7->5,9->12,9->2,9->8] | 0.90 | 0.0 | 33 | 163 | 0 | 33 |
| Path 1379 | C00031->C00002:[5->17,7->13,7->3,9->12,9->8] | 0.50 | 0.0 | 17 | 56 | 0 | 17 |
| Path 1380 | C00031->C00002:[1->12,2->13,4->17,5->3,5->8,7->11] | 0.60 | 0.0 | 19 | 139 | 0 | 19 |
| Path 1381 | C00031->C00002:[1->12,2->13,4->17,5->3,5->8,7->11] | 0.60 | 0.0 | 20 | 140 | 0 | 20 |
| Path 1382 | C00031->C00002:[5->13,7->17,7->3,9->12,9->8] | 0.50 | 0.0 | 15 | 359 | 0 | 15 |
| Path 1383 | C00031->C00002:[4->10,4->11,5->13,7->17,7->3,9->12,9->8] | 0.70 | 0.0 | 27 | 45 | 0 | 27 |
| Path 1384 | C00031->C00002:[4->10,4->11,5->17,7->13,7->3,7->5,9->12,9->2,9->8] | 0.90 | 0.0 | 35 | 397 | 0 | 35 |
| Path 1385 | C00031->C00002:[5->13,7->17,7->3,9->12,9->8] | 0.50 | 0.0 | 16 | 154 | 0 | 16 |
| Path 1386 | C00031->C00002:[5->17,7->13,7->3,9->12,9->8] | 0.50 | 0.0 | 17 | 149 | 0 | 17 |
| Path 1387 | C00031->C00002:[1->12,2->13,4->11,4->17,5->3,5->8] | 0.60 | 0.0 | 22 | 365 | 0 | 22 |
| Path 1388 | C00031->C00002:[4->10,4->11,5->13,7->17,7->3,7->5,9->12,9->2,9->8] | 0.90 | 0.0 | 34 | 169 | 0 | 34 |
| Path 1389 | C00031->C00002:[7->3,9->8] | 0.20 | 0.0 | 10 | 135 | 0 | 10 |
| Path 1390 | C00031->C00002:[1->12,2->13,4->10,4->17,4->4,5->8,9->3] | 0.70 | 0.0 | 22 | 152 | 0 | 22 |
| Path 1391 | C00031->C00002:[1->12,2->13,4->11,4->17,5->3,5->8] | 0.60 | 0.0 | 20 | 139 | 0 | 20 |
| Path 1392 | C00031->C00002:[5->13,7->17,7->3,9->12,9->8] | 0.50 | 0.0 | 14 | 138 | 0 | 14 |
| Path 1393 | C00031->C00002:[5->13,7->17,7->3,9->12,9->8] | 0.50 | 0.0 | 23 | 147 | 0 | 23 |
| Path 1394 | C00031->C00002:[4->10,4->11,5->13,7->17,7->3,7->5,9->12,9->2,9->8] | 0.90 | 0.0 | 32 | 51 | 0 | 32 |
| Path 1395 | C00031->C00002:[4->10,4->11,5->13,7->17,7->3,7->5,9->12,9->2,9->8] | 0.90 | 0.0 | 34 | 180 | 0 | 34 |
| Path 1396 | C00031->C00002:[1->12,2->13,4->10,4->11,4->17,5->3,5->8,7->5,9->2] | 0.90 | 0.0 | 30 | 373 | 0 | 30 |
| Path 1397 | C00031->C00002:[4->11,5->13,7->17,7->3,9->12,9->8] | 0.60 | 0.0 | 24 | 47 | 0 | 24 |
| Path 1398 | C00031->C00002:[4->10,4->11,5->13,7->17,7->3,9->12,9->8] | 0.70 | 0.0 | 25 | 164 | 0 | 25 |
| Path 1399 | C00031->C00002:[4->11,5->13,7->17,7->3,9->12,9->8] | 0.60 | 0.0 | 25 | 164 | 0 | 25 |
| Path 1400 | C00031->C00002:[4->11,5->13,7->17,7->3,9->12,9->8] | 0.60 | 0.0 | 23 | 156 | 0 | 23 |
| Path 1401 | C00031->C00002:[5->13,7->17,7->3,9->12,9->8] | 0.50 | 0.0 | 13 | 144 | 0 | 13 |
| Path 1402 | C00031->C00002:[1->12,2->13,4->17,5->3,5->8] | 0.50 | 0.0 | 14 | 136 | 0 | 14 |
| Path 1403 | C00031->C00002:[1->12,2->13,4->11,4->17,5->3,5->8] | 0.60 | 0.0 | 22 | 47 | 0 | 22 |
| Path 1404 | C00031->C00002:[4->10,4->11,5->17,7->13,7->3,9->12,9->8] | 0.70 | 0.0 | 28 | 61 | 0 | 28 |
| Path 1405 | C00031->C00002:[1->12,2->13,4->10,4->11,4->17,5->3,5->8,7->5,9->2] | 0.90 | 0.0 | 29 | 163 | 0 | 29 |
| Path 1406 | C00031->C00002:[4->10,4->11,5->13,7->17,7->3,9->12,9->8] | 0.70 | 0.0 | 24 | 157 | 0 | 24 |
| Path 1407 | C00031->C00002:[4->10,4->11,5->17,7->13,7->3,7->5,9->12,9->2,9->8] | 0.90 | 0.0 | 32 | 56 | 0 | 32 |
| Path 1408 | C00031->C00002:[4->11,5->17,7->13,7->3,9->12,9->8] | 0.60 | 0.0 | 24 | 169 | 0 | 24 |
| Path 1409 | C00031->C00002:[1->12,1->8,2->13,4->10,4->11,4->17,4->3,7->3,7->5,9->2,9->8] | 0.90 | 0.0 | 33 | 165 | 0 | 33 |
| Path 1410 | C00031->C00002:[4->10,4->11,5->13,7->17,7->3,9->12,9->8] | 0.70 | 0.0 | 25 | 52 | 0 | 25 |
| Path 1411 | C00031->C00002:[4->10,4->11,5->13,7->17,7->3,7->5,9->12,9->2,9->8] | 0.90 | 0.0 | 33 | 381 | 0 | 33 |
| Path 1412 | C00031->C00002:[4->11,5->13,7->17,7->3,9->12,9->8] | 0.60 | 0.0 | 23 | 158 | 0 | 23 |
| Path 1413 | C00031->C00002:[4->10,4->11,5->13,7->17,7->3,9->12,9->8] | 0.70 | 0.0 | 27 | 56 | 0 | 27 |
| Path 1414 | C00031->C00002:[5->13,7->17,7->3,9->12,9->8] | 0.50 | 0.0 | 16 | 35 | 0 | 16 |
| Path 1415 | C00031->C00002:[1->12,2->13,4->11,4->17,5->3,5->8] | 0.60 | 0.0 | 19 | 142 | 0 | 19 |
| Path 1416 | C00031->C00002:[7->3,9->8] | 0.20 | 0.0 | 10 | 19 | 0 | 10 |
| Path 1417 | C00031->C00002:[1->12,2->13,4->10,4->17,4->4,5->8,9->3] | 0.70 | 0.0 | 24 | 155 | 0 | 24 |
| Path 1418 | C00031->C00002:[4->11,5->17,7->13,7->3,9->12,9->8] | 0.60 | 0.0 | 24 | 157 | 0 | 24 |
| Path 1419 | C00031->C00002:[1->12,2->13,4->17,7->3,9->8] | 0.50 | 0.0 | 13 | 33 | 0 | 13 |
| Path 1420 | C00031->C00002:[4->10,4->11,5->13,7->17,7->3,9->12,9->8] | 0.70 | 0.0 | 27 | 387 | 0 | 27 |
| Path 1421 | C00031->C00002:[5->13,7->17,7->3,9->12,9->8] | 0.50 | 0.0 | 16 | 37 | 0 | 16 |
| Path 1422 | C00031->C00002:[4->11,5->13,7->17,7->3,9->12,9->8] | 0.60 | 0.0 | 23 | 151 | 0 | 23 |
| Path 1423 | C00031->C00002:[4->10,4->11,5->13,7->17,7->3,7->5,9->12,9->2,9->8] | 0.90 | 0.0 | 32 | 163 | 0 | 32 |
| Path 1424 | C00031->C00002:[1->12,2->13,4->10,4->11,4->17,7->3,9->8] | 0.70 | 0.0 | 21 | 46 | 0 | 21 |
| Path 1425 | C00031->C00002:[4->11,5->17,7->13,7->3,9->12,9->8] | 0.60 | 0.0 | 25 | 49 | 0 | 25 |
| Path 1426 | C00031->C00002:[4->10,4->11,5->17,7->13,7->3,9->12,9->8] | 0.70 | 0.0 | 28 | 159 | 0 | 28 |
| Path 1427 | C00031->C00002:[4->10,4->11,5->13,7->17,7->3,7->5,9->12,9->2,9->8] | 0.90 | 0.0 | 33 | 175 | 0 | 33 |
| Path 1428 | C00031->C00002:[1->12,2->13,4->10,4->11,4->17,5->3,5->8,7->5,9->2] | 0.90 | 0.0 | 32 | 378 | 0 | 32 |
| Path 1429 | C00031->C00002:[1->12,2->13,4->17,7->3,9->8] | 0.50 | 0.0 | 12 | 133 | 0 | 12 |
| Path 1430 | C00031->C00002:[5->13,7->17,7->3,9->12,9->8] | 0.50 | 0.0 | 16 | 154 | 0 | 16 |
| Path 1431 | C00031->C00002:[4->11,5->13,7->17,7->3,9->12,9->8] | 0.60 | 0.0 | 24 | 381 | 0 | 24 |
| Path 1432 | C00031->C00002:[5->13,7->17,7->3,9->12,9->8] | 0.50 | 0.0 | 14 | 26 | 0 | 14 |
| Path 1433 | C00031->C00002:[4->11,5->13,7->17,7->3,9->12,9->8] | 0.60 | 0.0 | 22 | 54 | 0 | 22 |
| Path 1434 | C00031->C00002:[1->12,2->13,4->17,5->8,7->11] | 0.50 | 0.0 | 18 | 141 | 0 | 18 |
| Path 1435 | C00031->C00002:[5->3,7->11] | 0.20 | 0.0 | 19 | 141 | 0 | 19 |
| Path 1436 | C00031->C00002:[4->10,4->11,5->17,7->13,7->3,9->12,9->8] | 0.70 | 0.0 | 28 | 75 | 0 | 28 |
| Path 1437 | C00031->C00002:[4->11,5->13,7->17,7->3,9->12,9->8] | 0.60 | 0.0 | 23 | 159 | 0 | 23 |
| Path 1438 | C00031->C00002:[4->10,4->11,5->13,7->17,7->3,7->5,9->12,9->2,9->8] | 0.90 | 0.0 | 33 | 385 | 0 | 33 |
| Path 1439 | C00031->C00002:[4->10,4->11,5->17,7->13,7->3,9->12,9->8] | 0.70 | 0.0 | 27 | 379 | 0 | 27 |
| Path 1440 | C00031->C00002:[4->11,5->13,7->17,7->3,9->12,9->8] | 0.60 | 0.0 | 23 | 54 | 0 | 23 |
| Path 1441 | C00031->C00002:[4->10,4->11,5->17,7->13,7->3,7->5,9->12,9->2,9->8] | 0.90 | 0.0 | 35 | 82 | 0 | 35 |
| Path 1442 | C00031->C00002:[4->10,4->11,5->13,7->17,7->3,9->12,9->8] | 0.70 | 0.0 | 25 | 55 | 0 | 25 |
| Path 1443 | C00031->C00002:[1->12,2->13,4->17,7->3,9->8] | 0.50 | 0.0 | 13 | 22 | 0 | 13 |
| Path 1444 | C00031->C00002:[4->10,4->11,5->17,7->13,7->3,9->12,9->8] | 0.70 | 0.0 | 29 | 177 | 0 | 29 |
| Path 1445 | C00031->C00002:[5->17,7->13,7->3,9->12,9->8] | 0.50 | 0.0 | 16 | 45 | 0 | 16 |
| Path 1446 | C00031->C00002:[4->10,4->11,5->13,7->17,7->3,9->12,9->8] | 0.70 | 0.0 | 25 | 48 | 0 | 25 |
| Path 1447 | C00031->C00002:[5->13,7->17,7->3,9->12,9->8] | 0.50 | 0.0 | 19 | 143 | 0 | 19 |
| Path 1448 | C00031->C00002:[1->12,2->13,4->11,4->17,5->3,5->8] | 0.60 | 0.0 | 22 | 152 | 0 | 22 |
| Path 1449 | C00031->C00002:[1->12,2->13,4->10,4->11,4->17,5->3,5->8,7->5,9->2] | 0.90 | 0.0 | 32 | 378 | 0 | 32 |
| Path 1450 | C00031->C00002:[4->10,4->11,5->13,7->17,7->3,7->5,9->12,9->2,9->8] | 0.90 | 0.0 | 32 | 162 | 0 | 32 |
| Path 1451 | C00031->C00002:[1->12,2->13,4->17,7->3,9->8] | 0.50 | 0.0 | 12 | 137 | 0 | 12 |
| Path 1452 | C00031->C00002:[1->12,2->13,4->10,4->11,4->17,5->3,5->8] | 0.70 | 0.0 | 26 | 155 | 0 | 26 |
| Path 1453 | C00031->C00002:[1->12,2->13,4->10,4->11,4->17,5->3,5->8,7->5,9->2] | 0.90 | 0.0 | 32 | 379 | 0 | 32 |
| Path 1454 | C00031->C00002:[1->12,2->13,4->11,4->17,5->3,5->8] | 0.60 | 0.0 | 23 | 149 | 0 | 23 |
| Path 1455 | C00031->C00002:[4->10,4->11,5->13,7->17,7->3,7->5,9->12,9->2,9->8] | 0.90 | 0.0 | 33 | 381 | 0 | 33 |
| Path 1456 | C00031->C00002:[1->12,2->13,4->17,5->8] | 0.40 | 0.0 | 9 | 132 | 0 | 9 |
| Path 1457 | C00031->C00002:[4->10,4->11,5->13,7->17,7->3,7->5,9->12,9->2,9->8] | 0.90 | 0.0 | 32 | 62 | 0 | 32 |
| Path 1458 | C00031->C00002:[1->12,2->13,4->17,5->8,7->11,9->3] | 0.60 | 0.0 | 15 | 135 | 0 | 15 |
| Path 1459 | C00031->C00002:[4->10,4->11,5->17,7->13,7->3,9->12,9->8] | 0.70 | 0.0 | 27 | 165 | 0 | 27 |
| Path 1460 | C00031->C00002:[1->12,2->13,4->17,5->8,7->11,9->3] | 0.60 | 0.0 | 15 | 132 | 0 | 15 |
| Path 1461 | C00031->C00002:[4->11,5->13,7->17,7->3,9->12,9->8] | 0.60 | 0.0 | 24 | 152 | 0 | 24 |
| Path 1462 | C00031->C00002:[4->10,4->11,5->13,7->17,7->3,7->5,9->12,9->2,9->8] | 0.90 | 0.0 | 30 | 162 | 0 | 30 |
| Path 1463 | C00031->C00002:[4->10,4->11,5->13,7->17,7->3,9->12,9->8] | 0.70 | 0.0 | 27 | 170 | 0 | 27 |
| Path 1464 | C00031->C00002:[1->12,2->13,4->11,4->17,5->3,5->8] | 0.60 | 0.0 | 24 | 157 | 0 | 24 |
| Path 1465 | C00031->C00002:[4->10,4->11,5->13,7->17,7->3,9->12,9->8] | 0.70 | 0.0 | 25 | 44 | 0 | 25 |
| Path 1466 | C00031->C00002:[7->11] | 0.10 | 0.0 | 10 | 127 | 0 | 10 |
| Path 1467 | C00031->C00002:[1->12,2->13,4->10,4->11,4->17,5->3,5->8] | 0.70 | 0.0 | 22 | 153 | 0 | 22 |
| Path 1468 | C00031->C00002:[4->10,4->11,5->13,7->17,7->3,9->12,9->8] | 0.70 | 0.0 | 26 | 57 | 0 | 26 |
| Path 1469 | C00031->C00002:[1->12,2->13,4->10,4->11,4->17,7->3,7->5,9->2,9->8] | 0.90 | 0.0 | 31 | 377 | 0 | 31 |
| Path 1470 | C00031->C00002:[1->12,2->13,4->17,5->3,5->8] | 0.50 | 0.0 | 14 | 131 | 0 | 14 |
| Path 1471 | C00031->C00002:[4->10,4->11,5->13,7->17,7->3,7->5,9->12,9->2,9->8] | 0.90 | 0.0 | 34 | 165 | 0 | 34 |
| Path 1472 | C00031->C00002:[5->13,7->17,7->3,9->12,9->8] | 0.50 | 0.0 | 16 | 147 | 0 | 16 |
| Path 1473 | C00031->C00002:[4->10,4->11,5->13,7->17,7->3,9->12,9->8] | 0.70 | 0.0 | 26 | 154 | 0 | 26 |
| Path 1474 | C00031->C00002:[4->11,5->17,7->13,7->3,9->12,9->8] | 0.60 | 0.0 | 24 | 50 | 0 | 24 |
| Path 1475 | C00031->C00002:[4->10,4->11,5->13,7->17,7->3,7->5,9->12,9->2,9->8] | 0.90 | 0.0 | 32 | 162 | 0 | 32 |
| Path 1476 | C00031->C00002:[5->13,7->17,7->3,9->12,9->8] | 0.50 | 0.0 | 14 | 141 | 0 | 14 |
| Path 1477 | C00031->C00002:[4->10,4->11,5->13,7->17,7->3,9->12,9->8] | 0.70 | 0.0 | 26 | 53 | 0 | 26 |
| Path 1478 | C00031->C00002:[4->10,4->11,5->17,7->13,7->3,9->12,9->8] | 0.70 | 0.0 | 27 | 162 | 0 | 27 |
| Path 1479 | C00031->C00002:[4->10,4->11,5->17,7->13,7->3,9->12,9->8] | 0.70 | 0.0 | 26 | 50 | 0 | 26 |
| Path 1480 | C00031->C00002:[1->12,2->13,4->17,7->3,9->8] | 0.50 | 0.0 | 11 | 129 | 0 | 11 |
| Path 1481 | C00031->C00002:[5->13,7->17,7->3,9->12,9->8] | 0.50 | 0.0 | 14 | 135 | 0 | 14 |
| Path 1482 | C00031->C00002:[1->12,2->13,2->3,4->10,4->11,4->17,5->8,7->5,9->2] | 0.90 | 0.0 | 29 | 159 | 0 | 29 |
| Path 1483 | C00031->C00002:[4->10,4->11,5->17,7->13,7->3,9->12,9->8] | 0.70 | 0.0 | 27 | 163 | 0 | 27 |
| Path 1484 | C00031->C00002:[4->10,4->11,5->13,7->17,7->3,7->5,9->12,9->2,9->8] | 0.90 | 0.0 | 31 | 167 | 0 | 31 |
| Path 1485 | C00031->C00002:[5->13,7->17,7->3,9->12,9->8] | 0.50 | 0.0 | 15 | 135 | 0 | 15 |
| Path 1486 | C00031->C00002:[5->17,7->13,7->3,9->12,9->8] | 0.50 | 0.0 | 17 | 28 | 0 | 17 |
| Path 1487 | C00031->C00002:[1->12,2->13,4->10,4->11,4->17,5->3,5->8] | 0.70 | 0.0 | 25 | 49 | 0 | 25 |
| Path 1488 | C00031->C00002:[1->12,2->13,4->17,5->8,7->11] | 0.50 | 0.0 | 19 | 145 | 0 | 19 |
| Path 1489 | C00031->C00002:[1->12,2->13,4->10,4->11,4->17,5->3,5->8,7->5,9->2] | 0.90 | 0.0 | 30 | 163 | 0 | 30 |
| Path 1490 | C00031->C00002:[5->13,7->17,7->3,9->12,9->8] | 0.50 | 0.0 | 15 | 149 | 0 | 15 |
| Path 1491 | C00031->C00002:[4->11,5->13,7->17,7->3,9->12,9->8] | 0.60 | 0.0 | 23 | 51 | 0 | 23 |
| Path 1492 | C00031->C00002:[5->13,7->17,7->3,9->12,9->8] | 0.50 | 0.0 | 15 | 37 | 0 | 15 |
| Path 1493 | C00031->C00002:[4->10,4->11,5->13,5->3,5->8,7->17,9->12] | 0.70 | 0.0 | 28 | 378 | 0 | 28 |
| Path 1494 | C00031->C00002:[1->12,2->13,2->3,4->17,5->8] | 0.50 | 0.0 | 14 | 140 | 0 | 14 |
| Path 1495 | C00031->C00002:[4->10,4->11,5->13,7->17,7->3,9->12,9->8] | 0.70 | 0.0 | 24 | 151 | 0 | 24 |
| Path 1496 | C00031->C00002:[4->10,4->11,5->13,7->17,7->3,9->12,9->8] | 0.70 | 0.0 | 26 | 371 | 0 | 26 |
| Path 1497 | C00031->C00002:[4->10,4->11,5->13,7->17,7->3,7->5,9->12,9->2,9->8] | 0.90 | 0.0 | 31 | 162 | 0 | 31 |
| Path 1498 | C00031->C00002:[1->12,2->13,4->10,4->11,4->17,5->3,5->8] | 0.70 | 0.0 | 26 | 62 | 0 | 26 |
| Path 1499 | C00031->C00002:[1->12,2->13,4->10,4->11,4->17,7->3,7->5,9->2,9->8] | 0.90 | 0.0 | 29 | 158 | 0 | 29 |
| Path 1500 | C00031->C00002:[4->10,4->11,5->17,7->13,7->3,9->12,9->8] | 0.70 | 0.0 | 28 | 61 | 0 | 28 |
| Path 1501 | C00031->C00002:[5->13,7->17,7->3,9->12,9->8] | 0.50 | 0.0 | 16 | 23 | 0 | 16 |
| Path 1502 | C00031->C00002:[1->12,2->13,2->3,4->10,4->11,4->17,5->8] | 0.70 | 0.0 | 24 | 158 | 0 | 24 |
| Path 1503 | C00031->C00002:[5->13,7->17,7->3,9->12,9->8] | 0.50 | 0.0 | 16 | 27 | 0 | 16 |
| Path 1504 | C00031->C00002:[1->12,2->13,4->10,4->11,4->17,7->3,7->5,9->2,9->8] | 0.90 | 0.0 | 29 | 46 | 0 | 29 |
| Path 1505 | C00031->C00002:[4->10,4->11,5->13,7->17,7->3,7->5,9->12,9->2,9->8] | 0.90 | 0.0 | 32 | 167 | 0 | 32 |
| Path 1506 | C00031->C00002:[1->12,2->13,4->17,5->3,5->8,7->11] | 0.60 | 0.0 | 22 | 145 | 0 | 22 |
| Path 1507 | C00031->C00002:[4->10,4->11,5->13,7->17,7->3,7->5,9->12,9->2,9->8] | 0.90 | 0.0 | 29 | 49 | 0 | 29 |
| Path 1508 | C00031->C00002:[4->11,5->13,7->17,7->3,9->12,9->8] | 0.60 | 0.0 | 23 | 372 | 0 | 23 |
| Path 1509 | C00031->C00002:[5->13,7->17,7->3,9->12,9->8] | 0.50 | 0.0 | 20 | 144 | 0 | 20 |
| Path 1510 | C00031->C00002:[4->10,4->11,5->13,7->17,7->3,7->5,9->12,9->2,9->8] | 0.90 | 0.0 | 33 | 67 | 0 | 33 |
| Path 1511 | C00031->C00002:[4->10,4->11,5->13,7->17,7->3,7->5,9->12,9->2,9->8] | 0.90 | 0.0 | 32 | 162 | 0 | 32 |
| Path 1512 | C00031->C00002:[1->12,2->13,4->17,5->8,9->11,9->3] | 0.60 | 0.0 | 18 | 145 | 0 | 18 |
| Path 1513 | C00031->C00002:[2->8,4->10,4->11,5->13,5->3,7->17,9->12] | 0.70 | 0.0 | 26 | 369 | 0 | 26 |
| Path 1514 | C00031->C00002:[1->12,2->13,4->11,4->17,5->8,9->3] | 0.60 | 0.0 | 18 | 147 | 0 | 18 |
| Path 1515 | C00031->C00002:[4->10,4->11,5->17,7->13,7->3,7->5,9->12,9->2,9->8] | 0.90 | 0.0 | 34 | 168 | 0 | 34 |
| Path 1516 | C00031->C00002:[4->10,4->11,5->13,7->17,7->3,9->12,9->8] | 0.70 | 0.0 | 24 | 59 | 0 | 24 |
| Path 1517 | C00031->C00002:[4->10,4->11,5->17,7->13,7->3,9->12,9->8] | 0.70 | 0.0 | 27 | 62 | 0 | 27 |
| Path 1518 | C00031->C00002:[5->13,7->17,7->3,9->12,9->8] | 0.50 | 0.0 | 21 | 148 | 0 | 21 |
| Path 1519 | C00031->C00002:[4->10,4->11,5->13,7->17,7->3,9->12,9->8] | 0.70 | 0.0 | 25 | 158 | 0 | 25 |
| Path 1520 | C00031->C00002:[4->10,4->11,5->13,7->17,7->3,9->12,9->8] | 0.70 | 0.0 | 26 | 67 | 0 | 26 |
| Path 1521 | C00031->C00002:[2->8,4->10,4->11,5->13,5->3,7->17,9->12] | 0.70 | 0.0 | 25 | 50 | 0 | 25 |
| Path 1522 | C00031->C00002:[1->12,2->13,4->10,4->11,4->17,4->4,5->8,9->3] | 0.80 | 0.0 | 27 | 155 | 0 | 27 |
| Path 1523 | C00031->C00002:[4->10,4->11,5->17,7->13,7->3,7->5,9->12,9->2,9->8] | 0.90 | 0.0 | 32 | 168 | 0 | 32 |
| Path 1524 | C00031->C00002:[1->12,2->13,4->10,4->11,4->17,5->3,5->8] | 0.70 | 0.0 | 22 | 145 | 0 | 22 |
| Path 1525 | C00031->C00002:[4->10,4->11,5->13,7->17,7->3,9->12,9->8] | 0.70 | 0.0 | 26 | 59 | 0 | 26 |
| Path 1526 | C00031->C00002:[4->10,4->11,5->13,7->17,7->3,7->5,9->12,9->2,9->8] | 0.90 | 0.0 | 32 | 163 | 0 | 32 |
| Path 1527 | C00031->C00002:[4->10,4->11,5->13,7->17,7->3,7->5,9->12,9->2,9->8] | 0.90 | 0.0 | 32 | 62 | 0 | 32 |
| Path 1528 | C00031->C00002:[1->12,2->13,4->17,7->3,9->8] | 0.50 | 0.0 | 12 | 32 | 0 | 12 |
| Path 1529 | C00031->C00002:[5->17,7->13,7->3,9->12,9->8] | 0.50 | 0.0 | 15 | 140 | 0 | 15 |
| Path 1530 | C00031->C00002:[4->11,5->13,7->17,7->3,9->12,9->8] | 0.60 | 0.0 | 24 | 154 | 0 | 24 |
| Path 1531 | C00031->C00002:[4->11,5->17,7->13,7->3,9->12,9->8] | 0.60 | 0.0 | 25 | 161 | 0 | 25 |
| Path 1532 | C00031->C00002:[4->11,5->17,7->13,7->3,9->12,9->8] | 0.60 | 0.0 | 25 | 55 | 0 | 25 |
| Path 1533 | C00031->C00002:[4->10,4->11,5->17,7->13,7->3,7->5,9->12,9->2,9->8] | 0.90 | 0.0 | 36 | 184 | 0 | 36 |
| Path 1534 | C00031->C00002:[4->11,5->13,7->17,7->3,9->12,9->8] | 0.60 | 0.0 | 24 | 62 | 0 | 24 |
| Path 1535 | C00031->C00002:[4->11,5->13,7->17,7->3,9->12,9->8] | 0.60 | 0.0 | 24 | 155 | 0 | 24 |
| Path 1536 | C00031->C00002:[1->12,2->13,4->10,4->11,4->17,5->3,5->8,7->10,7->11,7->5,9->2] | 0.90 | 0.0 | 31 | 162 | 0 | 31 |
| Path 1537 | C00031->C00002:[4->11,5->13,7->17,7->3,9->12,9->8] | 0.60 | 0.0 | 25 | 369 | 0 | 25 |
| Path 1538 | C00031->C00002:[1->12,2->13,4->11,4->17,5->3,5->8] | 0.60 | 0.0 | 23 | 365 | 0 | 23 |
| Path 1539 | C00031->C00002:[4->11,5->13,7->17,7->3,9->12,9->8] | 0.60 | 0.0 | 21 | 44 | 0 | 21 |
| Path 1540 | C00031->C00002:[4->10,4->11,5->13,7->17,7->3,9->12,9->8] | 0.70 | 0.0 | 26 | 161 | 0 | 26 |
| Path 1541 | C00031->C00002:[4->10,4->11,5->13,7->17,7->3,9->12,9->8] | 0.70 | 0.0 | 25 | 161 | 0 | 25 |
| Path 1542 | C00031->C00002:[4->10,4->11,5->13,7->17,7->3,9->12,9->8] | 0.70 | 0.0 | 24 | 159 | 0 | 24 |
| Path 1543 | C00031->C00002:[4->10,4->11,5->17,7->13,7->3,9->12,9->8] | 0.70 | 0.0 | 27 | 389 | 0 | 27 |
| Path 1544 | C00031->C00002:[4->11,5->13,7->17,7->3,9->12,9->8] | 0.60 | 0.0 | 23 | 159 | 0 | 23 |
| Path 1545 | C00031->C00002:[1->12,2->13,4->17,5->3,5->8] | 0.50 | 0.0 | 11 | 126 | 0 | 11 |
| Path 1546 | C00031->C00002:[1->12,2->13,4->17,5->8,7->11] | 0.50 | 0.0 | 19 | 146 | 0 | 19 |
| Path 1547 | C00031->C00002:[4->10,4->11,5->17,7->13,7->3,7->5,9->12,9->2,9->8] | 0.90 | 0.0 | 36 | 184 | 0 | 36 |
| Path 1548 | C00031->C00002:[2->8,4->11,5->13,5->3,7->17,9->12] | 0.60 | 0.0 | 22 | 51 | 0 | 22 |
| Path 1549 | C00031->C00002:[1->12,2->13,4->10,4->11,4->17,5->3,5->8,7->5,9->2] | 0.90 | 0.0 | 29 | 373 | 0 | 29 |
| Path 1550 | C00031->C00002:[4->11,5->17,7->13,7->3,9->12,9->8] | 0.60 | 0.0 | 26 | 59 | 0 | 26 |
| Path 1551 | C00031->C00002:[4->10,4->11,5->13,7->17,7->3,9->12,9->8] | 0.70 | 0.0 | 26 | 153 | 0 | 26 |
| Path 1552 | C00031->C00002:[1->12,2->13,4->11,4->17,7->3,9->8] | 0.60 | 0.0 | 21 | 154 | 0 | 21 |
| Path 1553 | C00031->C00002:[5->13,7->17,7->3,9->12,9->8] | 0.50 | 0.0 | 13 | 357 | 0 | 13 |
| Path 1554 | C00031->C00002:[4->11,5->13,7->17,7->3,9->12,9->8] | 0.60 | 0.0 | 24 | 383 | 0 | 24 |
| Path 1555 | C00031->C00002:[4->11,5->13,7->17,7->3,9->12,9->8] | 0.60 | 0.0 | 23 | 155 | 0 | 23 |
| Path 1556 | C00031->C00002:[4->10,4->11,5->13,7->17,7->3,7->5,9->12,9->2,9->8] | 0.90 | 0.0 | 32 | 167 | 0 | 32 |
| Path 1557 | C00031->C00002:[1->12,2->13,4->17,5->3,5->8,7->11] | 0.60 | 0.0 | 21 | 148 | 0 | 21 |
| Path 1558 | C00031->C00002:[4->10,4->11,5->13,7->17,7->3,7->5,9->12,9->2,9->8] | 0.90 | 0.0 | 34 | 79 | 0 | 34 |
| Path 1559 | C00031->C00002:[4->10,4->11,5->13,7->17,7->3,7->5,9->12,9->2,9->8] | 0.90 | 0.0 | 32 | 51 | 0 | 32 |
| Path 1560 | C00031->C00002:[4->10,4->11,5->13,7->17,7->3,9->12,9->8] | 0.70 | 0.0 | 25 | 160 | 0 | 25 |
| Path 1561 | C00031->C00002:[4->10,4->11,5->17,7->13,7->3,9->12,9->8] | 0.70 | 0.0 | 27 | 66 | 0 | 27 |
| Path 1562 | C00031->C00002:[4->10,4->11,5->13,7->17,7->3,9->12,9->8] | 0.70 | 0.0 | 24 | 59 | 0 | 24 |
| Path 1563 | C00031->C00002:[1->12,2->13,4->10,4->11,4->17,7->3,7->5,9->2,9->8] | 0.90 | 0.0 | 30 | 58 | 0 | 30 |
| Path 1564 | C00031->C00002:[5->13,7->17,7->3,9->12,9->8] | 0.50 | 0.0 | 16 | 142 | 0 | 16 |
