# Supplementary material for: Inferring branching pathways in genome-scale metabolic networks
Source: BMC Syst Biol. 2009 Oct 29;3:103. doi: 10.1186/1752-0509-3-103 (PMC2791103; doi:10.1186/1752-0509-3-103)
Supplement: Additional file 2 — ReTrace results from experiments. Summary data and html output from ReTrace runs performed for the queries discussed in the section Results. A self-contained web site: unpack archive and open index.html in a web browser. [file 1752-0509-3-103-S2.zip › retrace-AF2/index.html]

ReTrace - Additional file 2


This document is the Additional file 2 of the manuscript

```
Esa Pitk�nen, Paula Jouhten and Juho Rousu:
Inferring branching pathways in genome-scale metabolic networks. 
Submitted, 2009.
```

You can find Additional file 1 here.

ReTrace
is a computational method and software for inferring branching
pathways in genome-scale networks.

This document contains data from experiments described in the manuscript.

1. Atom graph
   construction: atomgraph.txt contains the
   following columns:
   - 1 - atom graph node identifier (KEGG compound identifier -
     atom number)
   - 2 - number of nodes connected to this node
   - 3 - average shortest path distance from this atom node to other nodes
   - 4 - variance of shortest path distances
   - 5 - standard deviation of shortest path distances
   - 6 - atom type
2. Performance testing: performance.txt
   is a summary file written by ReTrace during the experiment, which
   reports ReTrace parameters used and information on result
   pathways.
3. Glucose - IMP
